# Novel “GaEl Antigenic Patches” Identified by a “Reverse Epitomics” Approach to Design Multipatch Vaccines against NIPAH Infection, a Silent Threat to Global Human Health

**DOI:** 10.1021/acsomega.3c01909

**Published:** 2023-08-22

**Authors:** Sukrit Srivastava, Michael Kolbe

**Affiliations:** † Infection Biology Group, Indian Foundation for Fundamental Research Trust, Raebareli, Uttar Pradesh 229316, India; ‡ Department for Structural Infection Biology, Centre for Structural Systems Biology (CSSB) & Helmholtz-Centre for Infection Research, Notkestraße 85, 22607 Hamburg, Germany; § Faculty of Mathematics, Informatics and Natural Sciences, University of Hamburg, Rothenbaumchaussee 19, 20148 Hamburg, Germany

## Abstract

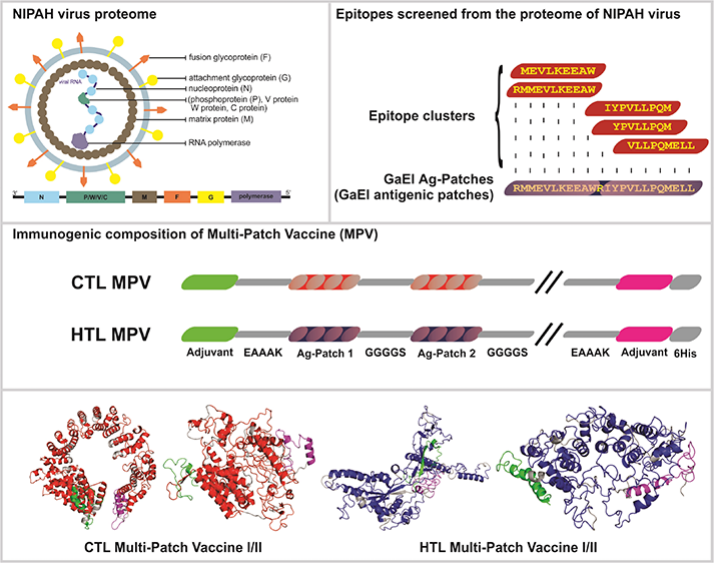

Nipah virus (NiV) is a zoonotic virus that causes lethal encephalitis and respiratory disease with the symptom of endothelial cell–cell fusion. Several NiV outbreaks have been reported since 1999 with nearly annual occurrences in Bangladesh. The outbreaks had high mortality rates ranging from 40 to 90%. No specific vaccine has yet been reported against NiV. Recently, several vaccine candidates and different designs of vaccines composed of epitopes against NiV were proposed. Most of the vaccines target single protein or protein complex subunits of the pathogen. The multiepitope vaccines proposed also cover a largely limited number of epitopes, and hence, their efficiency is still uncertain. To address the urgent need for a specific and effective vaccine against NiV infection, in the present study, we have utilized the “reverse epitomics” approach (“overlapping-epitope-clusters-to-patches” method) to identify “antigenic patches” (Ag-Patches) and utilize them as immunogenic composition for multipatch vaccine (MPV) design. The designed MPVs were analyzed for immunologically crucial parameters, physiochemical properties, and interaction with Toll-like receptor 3 ectodomain. In total, 30 CTL (cytotoxic T lymphocyte) and 27 HTL (helper T lymphocyte) antigenic patches were identified from the entire NiV proteome based on the clusters of overlapping epitopes. These identified Ag-Patches cover a total of discrete 362 CTL and 414 HTL epitopes from the entire proteome of NiV. The antigenic patches were utilized as immunogenic composition for the design of two CTL and two HTL multipatch vaccines. The 57 antigenic patches utilized here cover 776 overlapping epitopes targeting 52 different HLA class I and II alleles, providing a global ethnically distributed human population coverage of 99.71%. Such large number of epitope coverage resulting in large human population coverage cannot be reached with single-protein/subunit or multiepitope based vaccines. The reported antigenic patches also provide potential immunogenic composition for early detection diagnostic kits for NiV infection. Further, all the MPVs and Toll-like receptor ectodomain complexes show a stable nature of molecular interaction with numerous hydrogen bonds, salt bridges, and nonbounded contact formation and acceptable root mean square deviation and fluctuation. The cDNA analysis shows a favorable large-scale expression of the MPV constructs in a human cell line. By utilizing the novel “reverse epitomics” approach, highly immunogenic novel “GaEl antigenic patches” (GaEl Ag-Patches), a synonym term for “antigenic patches”, were identified and utilized as immunogenic composition to design four MPVs against NiV. We conclude that the novel multipatch vaccines are potential candidates to combat NiV, with greater effectiveness, high specificity, and large human population coverage worldwide.

## Introduction

Nipah virus (NiV) is a zoonotic virus of the genus *Henipavirus* and family Paramyxoviridae.^1^ The first NiV outbreak was reported among pigs in Malaysia in 1999.^2^ Later, another NiV outbreak was reported in Meherpur, Bangladesh, in year 2001, this time in humans. The transmission of NiV infection in Bangladesh and India was associated with both the contaminated date palm sap and the human-to-human contact.^3^ Bats were identified as the main reservoir for NiV, and they are responsible for the transmission of the infection to both humans and animals.^4^ Since 2001, NiV outbreaks have been reported for Bangladesh almost every year (2003–2005, 2007–2012). In India, two NiV outbreaks were reported in the state of West Bengal in 2001 and 2007.^5^ Afterward, another NiV outbreak was reported in the state of Kerala in India during the period of May to June in 2018. The Kerala outbreak claimed 17 human lives, leaving only 2 survivors out of 19 confirmed cases.^6^ Hitherto, no efficient vaccine against NiV has been reported. Vaccines targeting multiple proteins of NiV might provide efficient protection, and their potential needs to be explored in the future.

The essential proteins involved in NiV pathogenesis to humans include C protein, glycoproteins (G), matrix proteins (M), fusion glycoproteins (F), nucleocapsid protein, phosphoprotein, polymerase, V protein, and W protein.^7−21^ The C protein regulates the early host proinflammatory response as well as the pathogen virulence; the glycoprotein (G), the matrix protein (M), and the fusion protein (F) together form a cluster on the host human cell membrane and facilitate virus particle assembly formation.^22,23^ The NiV polymerase is essentially responsible for the initiation of RNA synthesis, primer extension, and transition to elongation mode. The phosphoprotein and glycoprotein are also essentially involved in viral replication, whereas the V protein is responsible for the host interferon signaling evasion.^17−20^ The identical N-terminal region of the V and W proteins was found to be sufficient to exert the IFN-antagonist activity.^21^ Hence, all the above-mentioned nine NiV proteins play an essential role in NiV proliferation and pathogenesis and so provide important drug and vaccine target candidates.

In recent studies, a number of T-cell and B-cell epitope candidates from different NiV proteins have been reported. Additionally, a number of vaccine design approaches and vaccine candidates including multiepitope vaccines were reported.^22,24−37^ However, using a single or a small number of epitopes might be limiting the vaccine potential. The long-term adaptive immunity essentially involves the presentation of the antigen as short peptides (epitope) on the surface of antigen presenting cells (APCs). To achieve this presentation, an intracellular proteolytic chop down process is orchestrated by the proteasome and lysosome. The “transporter associated with antigen processing” (TAP) and further the binding HLA allele (human leukocyte antigen) molecules facilitate the epitope presentation.^38,39^

Here, we introduce the term “GaEl antigenic patch” (GaEl Ag-Patch) as a synonym for “antigenic patch”. This is to acknowledge the inventors of “reverse epitomics”, and the prefix GaEl is derived from river Ganga and Elna of the inventors’ home countries. The novel “reverse epitomics” approach to identify “GaEl Ag-Patch” has been introduced in our earlier study on SARS-CoV-2.^40−43^ In the present study, we have utilized the “reverse epitomics” approach to identify “GaEl Ag-Patch” from NiV proteins. The reverse epitomics approach applies the novel “overlapping-epitope-clusters-to-patches” method to identify the “GaEl Ag-Patches”. Here, we first identify the overlapping epitopes that arise from a particular region of the protein. This particular region is a consensus peptide sequence of all the overlapping epitopes, and we identify this region of protein as “GaEl Ag-Patches”. From the proteome of NiV, here we report a total of 57 “GaEl Ag-Patches” identified from overlapping 776 epitopes. Next, we utilized these “GaEl Ag-Patches” from the NiV proteome as immunogenic composition to design multipatch vaccines.

## Methodology

In the present study, we have designed two CTL and two HTL multipatch vaccines (MPVs). These vaccines are composed of “GaEL antigenic patches” (GaEl Ag-Patches) identified from the proteome of NiV. The “GaEl Ag-Patches” were identified by the reverse epitomics approach as introduced in our earlier studies.^40−43^ All nine proteins of the NiV proteome (https://www.uniprot.org/proteomes/UP000120177) were used in this study, viz., C protein (gi-1859635642), glycoprotein (gi-253559848), fusion protein (gi-13559813), nucleoprotein (gi-1679387250), matrix protein (gi-13559811), phosphoprotein (gi-1802790259), W protein (gi-374256971), V protein (gi-1802790260), and RNA polymerase (gi-15487370). The full-length amino acid sequences of NiV proteins were retrieved from the National Center for Biotechnology Information (NCBI). Up to 96 full-length protein sequences belonging to different strains and origins of NiV were retrieved. The MPVs designed with antigenic patches as immunogenic composition carry potential discontinuous B-cell epitopes as well as IFN-γ inducing epitopes in their tertiary structure models. Hence, the designed MPVs carry the potential to elicit cell-mediated as well as humoral immune response. Furthermore, both MPVs were designed with the adjuvants human β defensin 2 and human β defensin 3 at N and C termini.^44,45^ The β-defensins have considerable immunological adjuvant activity and generate potent humoral immune responses.^45−48^ Tertiary structure models of the MPVs were generated, refined, and further analyzed for molecular interaction with the ectodomain of the human Toll-like receptor 3 (TLR3) by molecular docking. TLR3 is an essential immunoreceptor and acts as a sentinel to bind and process antigens causing activation of the IFN response against foreign antigens.^49,50^ With these functions, TLR3 plays an important role as immune-receptor during the NiV infection and hence was chosen to examine the candidate MPVs.^51,52^ The ability of CTL or HTL MPVs forming a complex with the ectodomain of human TLR3 was further investigated by molecular dynamics simulation studies. The cDNA of the designed MPVs was also analyzed for its high expression potential in the human (mammalian) host cell line. The corresponding workflow for the vaccine design and utility of “GaEl Ag-Patches” in diagnostics are shown in Figure S1.

### Screening of Potential Epitopes

#### T-Cell Epitope Prediction

##### Screening for Cytotoxic T Lymphocyte (CTL) Epitope

Identification of cytotoxic T lymphocyte epitopes was performed by the IEDB (Immune Epitope Database) tool “Proteasomal cleavage/TAP transport/MHC class I combined predictor” (http://tools.iedb.org/processing/) and “MHC-I Binding Predictions” (http://tools.iedb.org/mhci/).^53,54^ The tool provides a “Total Score” that is a combined score including results from the proteasome, MHC, TAP (N-terminal interaction), and processing analysis. A combination of six different methods, viz., Consensus, NN-align, SMM-align, Combinatorial library, Sturniolo, and the NetMHCIIpan, was applied.^55^ Further, immunogenicity of all the shortlisted CTL epitopes was obtained by using the “MHC I Immunogenicity” tool of IEDB (http://tools.iedb.org/immunogenicity/)^55^ on the basis of physiochemical properties of constituting amino acid of the peptide sequence.

##### Screening for Helper T Lymphocyte (HTL) Epitopes

The IEDB tool “MHC-II Binding Predictions” (http://tools.iedb.org/mhcii/) was used to identify helper T lymphocyte (HTL) epitopes from NiV proteins.^56−59^ Three different methods, viz., combinatorial library, SMM_align, and Sturniolo, were used; further, a comparison of score of the peptide against the scores of other known five million 15-mer peptides of the Swiss-Prot database was performed to screen HTL epitopes.^56−59^ A percentile rank for each peptide is generated, of which a lower value indicates a higher immunogenic potential of the HTL epitope.

##### CTL and HTL Epitope Toxicity Prediction

The ToxinPred tool was used to characterize the toxic potential of shortlisted CTL and HTL epitopes. The tool facilitates the identification of the highly toxic or nontoxic peptides.^60^ The “SVM (Swiss-Prot) based” (support vector machine) method was used here. The method utilizes a data set of 1805 sequences as positive (toxic) and 3593 sequences as negative (nontoxic) peptides from Swiss-Prot as well as 1805 positive and 12,541 negative peptide sequences from TrEMBLE.

### Overlapping CTL and HTL Epitope Clusters to “GaEl Antigenic Patches”

#### CTL and HTL Overlapping Epitope Clusters Based GaEl Antigenic Patch Identification

All the shortlisted high-scoring epitopes from NiV proteome were aligned using their amino acid sequences with the multiple sequence alignment (MSA) tool of Clustal Omega.^61^ The consensus amino acid sequence of overlapping epitopes was identified as “GaEl Ag-Patches”. This novel approach of search and identification of “GaEl antigenic patches” from a source protein is named as “reverse epitomics” as introduced and explained in our earlier studies.^40−43^ This approach is applicable to proteins/antigens of any pathogen and not only to SARS-CoV-2 or NiV.

#### Population Coverage by CTL and HTL Epitopes Covered by the “GaEl Ag-Patches”

The world population coverage by the overlapping CTL and HTL epitopes that were utilized to identify the GaEl Ag-Patches was studied by the “population coverage” tool of IEDB.^62^ T cells recognize complexes of MHC molecules with a given epitope. The epitope can elicit an immune response in individuals who express the binding MHC molecule.^59^ The MHC molecules are expressed differentially in the human population in different ethnically distributed populations. This MHC restricted epitope binding provides an opportunity to analyze worldwide population coverage by the given epitope.

#### Conservation Analysis of Antigenic Patches

The amino acid sequence conservation of the shortlisted CTL and HTL “GaEl antigenic patches” was analyzed with the “Epitope Conservancy Analysis” tool of IEDB. Epitope conservancy was performed against up to 96 protein sequences of NiV of different strains and origins collected from NCBI.^63^

### Multipatch Vaccines against NiV

The identified and shortlisted “GaEl Ag-Patches” from the NiV proteins were further used as immunogenic composition to design two CTL and two HTL multipatch vaccine constructs as explained in the Results section.

#### Physicochemical Property Analysis of the Designed MPVs

Two CTL and two HTL MPVs were analyzed with the ProtParam tool.^64^ The tool performs an empirical investigation of various physicochemical parameters, viz., amino acid length, theoretical pI, molecular weight, aliphatic index, expected half-life (in *Escherichia coli*, yeast, and mammalian cell), grand average of hydropathicity (GRAVY), and the instability index score. The aliphatic index and the grand average of hydropathicity (GRAVY) indicate the globular and hydrophilic nature of proteins. Further, the instability index score indicates the stable nature of proteins.

#### Interferon-Gamma Inducing Epitope Prediction from the MPVs

The two CTL and two HTL MPVs were screened for potential interferon-gamma (IFN-γ) inducing epitopes (from CD4+ T cells) utilizing the “IFN epitope” tool implementing the “Motif and SVM hybrid” method, i.e., MERCI: Motif-EmeRging and with Classes-Identification, and the SVM: support vector machine hybrid. The prediction is based on the IEDB database of 3705 IFN-gamma inducing and 6728 noninducing epitopes.^65,66^

#### MPV Allergenicity and Antigenicity Prediction

The CTL and HTL MPVs were analyzed for allergenicity and antigenicity with the AllergenFP and VaxiJen tools, respectively.^67,68^ The AllergenFP prediction is based on hydrophobicity, their size, their helix-forming propensity, the relative abundance of amino acids, and β-strand forming propensity. The VaxiJen tool utilizes an alignment-free approach based on physicochemical properties of query protein sequence.

#### Tertiary Structure Modeling and Refinement of MPVs

The tertiary structures of the MPVs were calculated by homology modeling utilizing I-TASSER, which utilizes the sequence-to-structure-to-function paradigm for protein structure prediction.^69^

The refinement of all calculated structural models (two CTL and two HTL MPVs) was performed with the GalaxyRefine tools.^71,72^ The GalaxyRefine tool refines the input tertiary structure by repeated structure perturbation followed by subsequent structural relaxation and molecular dynamics simulation.^73,74^

#### Validation of CTL and HTL MPV Refined Models

Both the refined two CTL and two HTL MPV tertiary models were further validated by the Ramachandran Plot Server (https://zlab.umassmed.edu/bu/rama/index.pl).^75,76^ The Ramachandran plots show sterically allowed and disallowed residues along with dihedral psi (ψ) and phi (φ) angles.

#### Linear and Discontinuous B-Cell Epitope Prediction from the MPVs

The IEDB tool Ellipro (ElliPro: Antibody Epitope Prediction tool) was used to screen the linear and discontinuous B-cell epitopes from all the CTL and HTL MPV tertiary models. Here, the farthest residue to be considered was limited to 6 Å, the residues lying outside of an ellipsoid covering 90% of the inner core residues of the protein score highest protrusion index (PI) of 0.9, and so on. The discontinuous epitopes were predicted based on the distance “*R*” in Å between the center of mass of two residues lying outside of the largest possible ellipsoid. The larger the value of *R* is, the greater is the distance between the residues (residue discontinuity) of the discontinuous epitopes.^77,78^

#### Molecular Interaction Analysis of MPVs and Immune Receptor Complexes

The molecular interaction of the CTL and HTL MPVs with the ectodomain of Toll-like receptor 3 (TLR3) was analyzed by molecular docking followed by a molecular dynamics simulation study. The protein–protein molecular docking was performed by the GRAMM-X Protein–Protein Docking v.1.2.0 tool.^79^ The GRAMM-X tool utilizes the GRAMM Fast Fourier Transformation (FFT) methodology by employing smoothed potentials, refinement stage, and knowledge-based scoring.

#### Molecular Dynamics (MD) Simulation Study of MPVs–TLR3(ECD) Complexes

The molecular interactions of MPVs–TLR3(ECD) complexes were further evaluated using molecular dynamics (MD) simulation analysis. The MD simulation studies were performed for 150 ns using the GROMACS tool.^80,81^ MD simulation studies were carried out in an explicit water environment in a cubic box as the unit cell simulation box at a stabilized temperature of 300 K and pressure of 1 atm with periodic cell boundary conditions. The solvated systems were neutralized with counter Cl^–^ ions. Only the ions necessary to neutralize the net charge on the protein are added by *gmx genion*. The OPLS all-atom force field was used on the systems during MD simulation.^82^ The solvated structures were energy minimized by steepest descent. Further, the complexes were equilibrated for a period of 100 ps. After equilibration, the MD simulation was run for 150 ns. The RMSD and RMSF values for Cα, backbone, and all the atoms of CTL and HTL MPVs in complex with TLR3 were analyzed.

#### Analysis of MPV cDNA for Expression in the Human Host Cell Line

A codon-optimized complementary DNA (cDNA) of the two CTL and two HTL MPVs was generated and analyzed for favored expression in the mammalian cell line (human) by the Java Codon Adaptation Tool. The cDNA of MPVs was further analyzed by the GenScript Rare Codon Analysis Tool for the large-scale expression potential. The tool analyzes the GC content, codon adaptation index (CAI), and the tandem rare codon frequency.^83,84^ The CAI indicates the possibility of expression in the chosen human cell line expression system. The tandem rare codon frequency indicates the presence of low-frequency codons in the cDNA construct.

## Results

### Screening of Potential Epitopes

#### T-Cell Epitope Prediction

##### Screening of Cytotoxic T Lymphocyte (CTL) Epitope

The cytotoxic T lymphocyte (CTL) epitopes were screened and shortlisted according to their high “Total Score”, low IC(50) (nM) value for epitope-HLA class I allele binding, and larger number of the HLA class I allele binders. Additional epitopes were screened from the protein sequences of NiV on the basis of their percentile rank. A smaller value of percentile rank indicates a higher affinity of peptide with its respective HLA class I allele binders. A total of 1811 (579 + 1232) CD8+ T-cell epitopes/HLA class I allele pairs were screened. These included the top-scoring epitopes and the highest-percentile-ranking epitopes from the NiV proteome. The immunogenicity of the shortlisted CTL epitopes was also determined. All the screened and shortlisted CTL epitopes are assessed as highly immunogenic in humans (Tables S1 and S2).

##### Screening of Helper T Lymphocyte (HTL) Epitopes

The screening of helper T lymphocyte (HTL) epitopes was performed on the basis of “percentile rank”. A smaller value of percentile rank suggests a higher affinity of the peptide with its respective HLA allele binders. We screened in total 773 potential CD4+ T-cell epitopes/allele pairs showing the highest percentile rank. The epitopes with the largest number of HLA class II allele binders were also included (Table S3).

##### CTL and HTL Epitope Toxicity Prediction

All the screened and shortlisted CTL and HTL epitopes were evaluated as nontoxic (Tables S1–S3).

Further, all the 1811 CTL and 773 HTL epitopes were evaluated to be highly conserved (IEDB: “Epitope Conservancy Analysis”) with 100% amino acid sequence of epitope being present in most of the NiV strains as shown in Tables S1–S3.

### Overlapping CTL and HTL Epitope Clusters to Antigenic Patches

#### CTL and HTL Overlapping Epitope Clusters Based Antigenic Patch Identification

**Figure 1:**
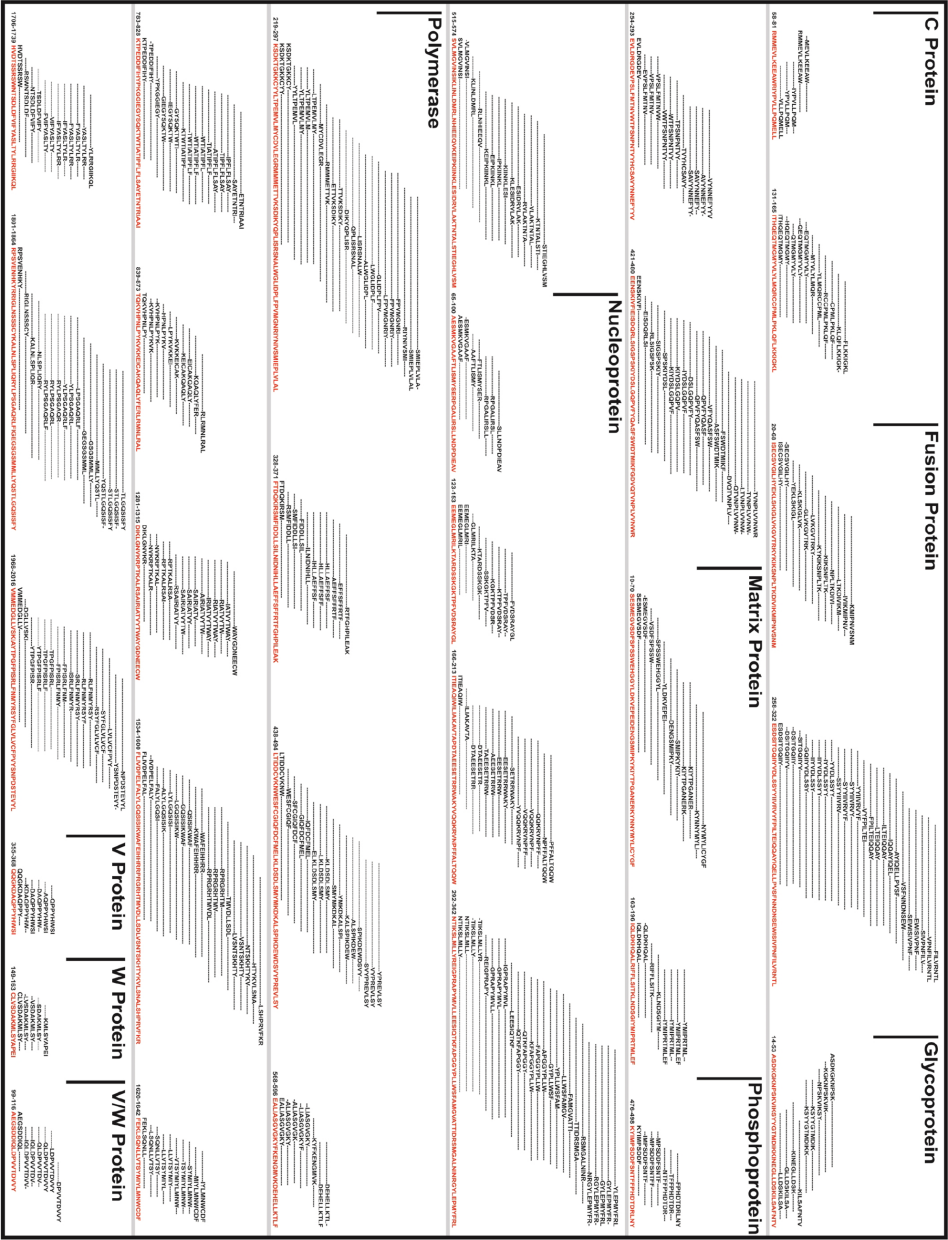
Graphical representation of the identified 30 CTL Ag-Patches from overlapping 362 CTL epitopes screened from NiV proteins. The CTL GaEl Ag-Patches amino acid consensus sequences are highlighted in red.

**Figure 2:**
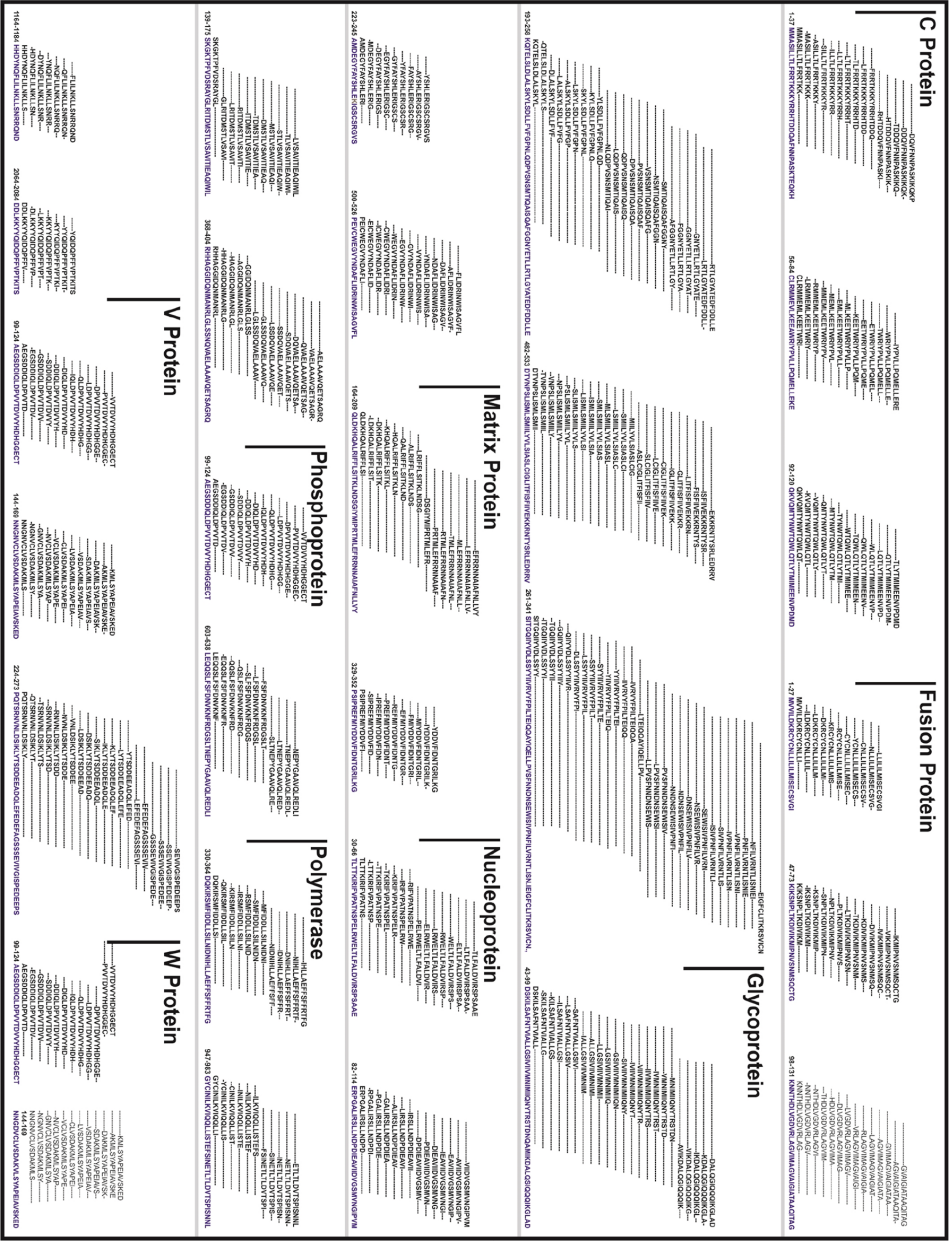
Graphical representation of the identified 27 HTL GaEl Ag-Patches from overlapping 414 HTL epitope clusters of NiV proteins. The HTL GaEl Ag-Patches are shown in blue amino acid sequence consensus.

**Figure 3:**
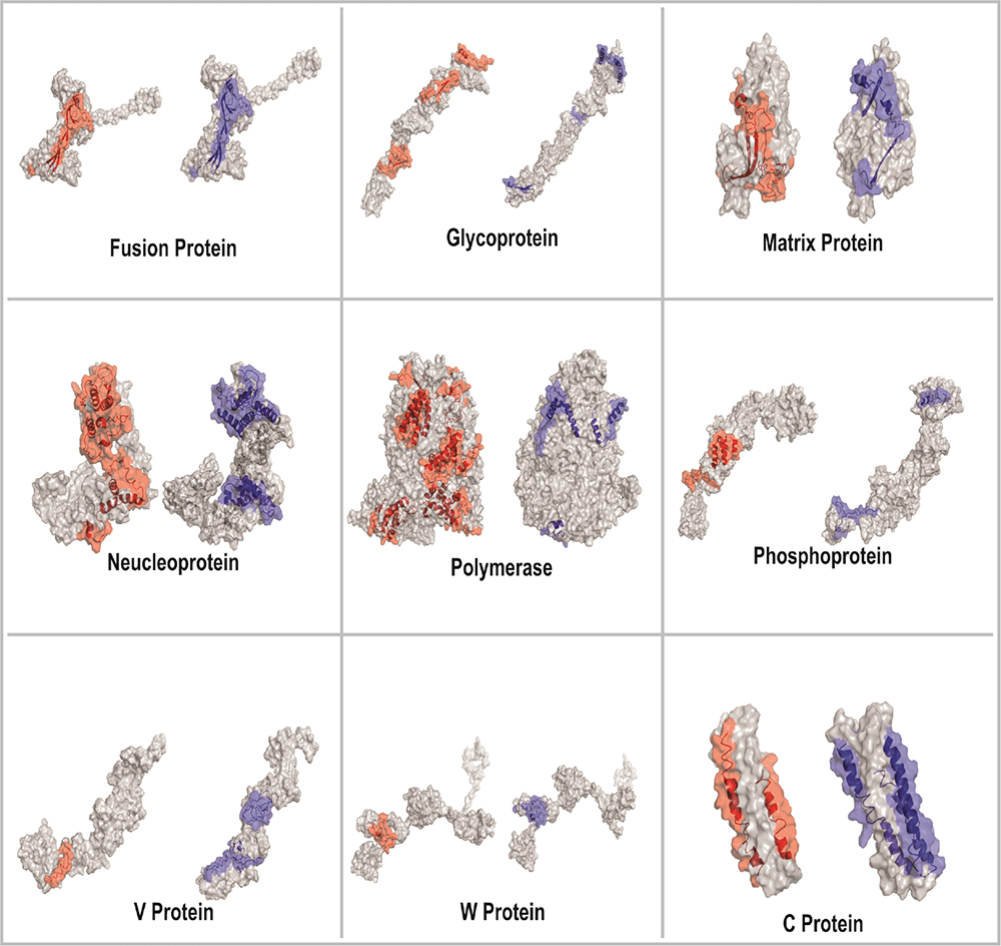
The identified 57 “GaEl Ag-Patches” are shown in tertiary structure models of the NiV proteins. The 30 CTL GaEl Ag-Patches are shown in red, and the 27 HTL GaEl Ag-Patches are shown in blue color. Most of the GaEl Ag-Patches identified are observed on the exposed surface of the NiV proteins. Structural modeling details are given in Table S4.

A total of 30 GaEl Ag-Patches from 362 CTL and 27 GaEl Ag-Patches from 414 HTL overlapping epitopes were identified. To identify the GaEl Ag-Patches, a novel “reverse epitomics” approach involving the “overlapping-epitope-clusters-to-patches” method was utilized^40−43^ (Tables 1 and 2). The herewith suggested GaEl Ag-Patches are expected to result in up to 776 overlapping epitopes upon intercellular proteolytic chop down by antigen presenting cells (APCs). Such large numbers of epitopes cannot be accommodated by multiepitope vaccines (MEV) candidates.^27,32,36,37,85,86^ Furthermore, lagging behind MPVs in respect of encoding a high number of epitopes, the MEVs with a short peptide of epitopes might also result in wrong or missfoled peptide output upon intracellular proteolytic chop down by APC. Structural analysis showed that all the identified GaEl Ag-Patches locate at the surface of NiV proteins, providing a more accessible target for immunogenic response. Therefore, the multipatch vaccine (MPV) candidates are expected to be superior by utilizing “GaEl Ag-Patches” as immunogenic composition (Figures 1–3). The tertiary structure models of NiV proteins are generated by SwissModel and I-TASSER homology modeling.^69,70^

**Table 1:** CTL “GaEl Ag-Patches” from NiV Proteome Were Identified by the Novel Reverse Epitomics Approach Involving the “Overlapping-Epitope-Clusters-to-Patches” Method^a^

#	CTL-MPVs	CTL overlapping epitope based GaEl Ag-Patches	epitopes in cluster	conservancy	source NIPAH proteins
CTL-MPV-1					
1	CTL-MPV-1	RMMEVLKEEAWRIYPVLLPQMELL	9	78.33% (47/60)	C protein/58–81
2	CTL-MPV-1	ITHQEQTMGMYVLYLMQRCCPMLPKLQFLKKIGKL	11	98.33% (59/60)	C protein/131–165
3	CTL-MPV-1	ISECSVGILHYEKLSKIGLVKGVTRKYKIKSNPLTKDIVIKMIP NVSNM	12	40.54% (15/37)	fusion protein/20–68
4	CTL-MPV-1	ESDSITGQIIYVDLSSYYIIVRVYFPILTEIQQAYIQELLPVSF NNDNSEWISIVPNFILVRNTL	25	45.95% (17/37)	fusion protein/258–322
5	CTL-MPV-1	ASDKGKNPSKVIKSYYGTMDIKKINEGLLDSKILSAFNTV	10	74.24% (49/66)	glycoprotein/14–53
6	CTL-MPV-1	EVLDRGDEVPSLFMTNVWTPSNPNTVYHCSAVYNNEFYYV	12	66.67% (44/66)	glycoprotein/254–293
7	CTL-MPV-1	EENSKIVFIEISDQRLSIGSPSKIYDSLGQPVFYQASFSWDT MIKFGDVQTVNPLVVNWR	19	60.61% (40/66)	glycoprotein/421–480
8	CTL-MPV-1	SESMEGVSDFSPSSWEHGGYLDKVEPEIDENGSMIPKYKIY TPGANERKYNNYMYLICYGF	11	43.59%	matrix protein/10–70
9	CTL-MPV-1	IQLDKHQALRIFFLSITKLNDSGIYMIPRTMLEF	8	97.44%	matrix protein/163–196
10	CTL-MPV-1	AESMKVGAAFTLISMYSERPGALIRSLLNDPDIEAV	9	100% (13/13)	nucleoprotein/65–100
11	CTL-MPV-1	EEMEGLMRILKTARDSSKGKTPFVDSRAYGL	9	100% (13/13)	nucleoprotein/123–153
12	CTL-MPV-1	ITIEAQIWILIAKAVTAPDTAEESETRRWAKYVQQKRVNPFFA LTQQW	15	100% (13/13)	nucleoprotein/166–213
13	CTL-MPV-1	NTIKSLMLLYREIGPRAPYMVLLEESIQTKFAPGGYPLLWSFAM GVATTIDRSMGALNINRGYLEPMYFRL	25	100% (13/13)	nucleoprotein/292–362
14	CTL-MPV-1	KYIMPSDDFSNTFFPHDTDRLNY	7	96.30% (26/27)	phosphoprotein/476–498
15	CTL-MPV-1	SVLMGVINSIKLINLDMRLNHIEEQVKEIPKIINKLESIDRVLAKT NTALSTIEGHLVSM	14	100% (27/27)	phosphoprotein/515–574
CTL-MPV-2					
16	CTL-MPV-2	KSDKTGKKCYYLTPEMVLMYCDVLEGRMMMETTVKSDIKYQPL ISRSNALWGLIDPLFPVMGNRIYNIVSMIEPLVLAL	22	70% (7/10)	polymerase/219–297
17	CTL-MPV-2	FTDQKIRSMFIDDLLSILNIDNIHLLAEFFSFFRTFGHPILEAK	11	100% (10/10)	polymerase/328–371
18	CTL-MPV-2	LTIDDCVKNWESFCGIQFDCFMELKLDSDLSMYMKDKALSPIKD EWDSVYPREVLSY	16	100% (10/10)	polymerase/438–494
19	CTL-MPV-2	EALIASGVGKYFKENGMVKDEHELLKTLF	10	100% (10/10)	polymerase/568–596
20	CTL-MPV-2	KTPEDDIFIHYPKGGIEGYSQKTWTIATIPFLFLSAYETNTRIAAI	16	90% (9/10)	polymerase/783–828
21	CTL-MPV-2	TQKVHPNLPYKVKKEICAKQAQLYFERLRMNLRAL	12	100% (10/10)	polymerase/839–873
22	CTL-MPV-2	DIKLGNVKRPTKALRSAIRIATVYTWAYGDNEECW	15	100% (10/10)	polymerase/1281–1315
23	CTL-MPV-2	FLIVDPELFALYLGQSISIKWAFEIHHRRPRGRHTMVDLLSDLVS NTSKHTYKVLSNALSHPRVFKR	24	100% (10/10)	polymerase/1534–1600
24	CTL-MPV-2	FEKLSQNLLVTSYMIYLMNWCDF	10	100% (10/10)	polymerase/1620–1642
25	CTL-MPV-2	HVDTSSRSWNTSDLDFVIFYASLTYLRRGIIKQL	12	100% (10/10)	polymerase/1706–1739
26	CTL-MPV-2	RPSVENHKYRRIGLNSSSCYKALNLSPLIQRYLPSGAQRLFIGE GSGSMMLLYQSTLGQSISFY	17	100% (10/10)	polymerase/1801–1864
27	CTL-MPV-2	VMMEDGLLVSKIAYTPGFPISRLFNMYRSYFGLVLVCFPVYSN PDSTEVYL	17	100% (10/10)	polymerase/1966–2016
28	CTL-MPV-2	AEGSDDIQLDPVVTDVVY	7	94.79% (91/96)	V and W protein/99–116
29	CTL-MPV-2	CLVSDAKVLSYAPEI	9	78.12% (75/96)	W protein/149–163
30	CTL-MPV-2	QQGKDAQPPYHWSI	6	19.30% (11/57)	V protein/355–368

a The highly immunogenic 30 Ag-Patches were utilized to design two CTL (CTL-MPV-1 and CTL-MPV-2) multipatch vaccine candidates. The amino acid sequences of the identified “GaEl Ag-Patches” were highly conserved. CTL “GaEl Ag-Patches” from NIPAH: Supplementary txt 1 and Table S7.

**Table 2:** HTL GaEl Ag-Patches from NiV Proteome Were Identified by the Novel Reverse Epitomics Approach Involving the “Overlapping-Epitope-Clusters-to-Patches” Method^a^

#	HTL-MPVs	HTL overlapping epitope based GaEl Ag-Patches	epitopes in cluster	conservancy	source NIPAH proteins
HTL-MPV-1					
1	HTL-MPV-1	MMASILLTLFRRTKKKYRRHTDDQAFNNPASKTEQKH	15	68.33% (41/60)	C protein/1–37
2	HTL-MPV-1	CLRMMEVLKEEAWRIYPVLLPQMELLEKE	13	78.33% (47/60)	C protein/56–84
3	HTL-MPV-1	QKVQMTYNWTQWLQTLYTMIMEENVPDMD	13	95.00% (57/60)	C protein/92–120
4	HTL-MPV-1	MVVILDKRCYCNLLILILMISECSVGI	11	40.54% (15/37)	fusion protein/1–27
5	HTL-MPV-1	KIKSNPLTKDIVIKMIPNVSNMSQCTG	13	97.30% (36/37)	fusion protein/47–73
6	HTL-MPV-1	KNNTHDLVGDVRLAGVIMAGVAIGIATAAQITAG	14	97.30% (36/37)	fusion protein/98–131
7	HTL-MPV-1	KQTELSLDLALSKYLSDLLFVFGPNLQDPVSNSMTIQ AISQAFGGNYETLLRTLGYATEDFDDLLE	23	94.59% (35/37)	fusion protein/193–258
8	HTL-MPV-1	SIT.GQIIYVDLSSYYIIVRVYFPILTEIQQAYIQELLPVSF NNDNSEWISIVPNFILVRNTLISNIEIGFCLITKRSVICN	29	45.95% (17/37)	fusion protein/261–341
9	HTL-MPV-1	DTVNPSLISMLSMIILYVLSIASLCIGLITFISFIIVEKKR NTYSRLEDRRV	23	100% (37/37)	fusion protein/482–533
10	HTL-MPV-1	DSKILSAFNTVIALLGSIVIIVMNIMIIQNYTRSTDNQA MIKDALQSIQQQIKGLAD	23	71.21% (47/66)	glycoprotein/43–99
11	HTL-MPV-1	AMDEGYFAYSHLEKIGSCSRGVS	9	65.15% (43/66)	glycoprotein/223–245
12	HTL-MPV-1	PEVCWEGVYNDAFLIDRINWISAGVFL	13	74.24% (49/66)	glycoprotein/500–526
#	HTL-MPVs	HTL overlapping epitope based GaEl Ag-Patches	epitopes in cluster	conservancy	source NIPAH proteins
HTL-MPV-2					
13	HTL-MPV-2	QLDKHQALRIFFLSITKLNDSGIYMIPRTMLEFRRNN AIAFNLLVY	15	84.62% (38/39)	matrix/164–209
14	HTL-MPV-2	PSIPREFMIYDDVFIDNTGRILKG	10	38.46% (15/39)	matrix/329–352
15	HTL-MPV-2	TLTTKIRIFVPATNSPELRWELTLFALDVIRSPSAAE	15	100% (13/13)	nucleoprotein/30–66
16	HTL-MPV-2	ERPGALIRSLLNDPDIEAVIIDVGSMVNGIPVM	14	100% (13/13)	nucleoprotein/82–114
17	HTL-MPV-2	SKGKTPFVDSRAYGLRITDMSTLVSAVITIEAQIWIL	11	100% (13/13)	nucleoprotein/139–175
18	HTL-MPV-2	RHHAGGIDQNMANRLGLSSNQVAELAAAVQETS AGRQ	14	84.62% (11/13)	nucleoprotein/368–404
19	HTL-MPV-2	AEGSDDIQLDPVVTDVVYHDHGGECT	12	100% (27/27)	phosphoprotein/99–124
20	HTL-MPV-2	LEQQSLFSFDNVKNFRDGSLTNEPYGAAVQLREDLI	11	62.96%(17/27)	phosphoprotein/603–638
21	HTL-MPV-2	DQKIRSMFIDDLLSILNIDNIHLLAEFFSFFRTFG	12	100%(10/10)	polymerase/330–364
22	HTL-MPV-2	GYCINILKVIQQLLISTEFSINETLTLDVTSPISNNL	11	100%(10/10)	polymerase/947–983
23	HTL-MPV-2	HHDYNQFLILNKLLSNRRQND	7	100%(10/10)	polymerase/1164–1184
24	HTL-MPV-2	DDLKKYYQIDQPFFVPTKITS	7	100%(10/10)	polymerase/2064–2084
25	HTL-MPV-2	AEGSDDIQLDPVVTDVVYHDHGGECT	12	94.79% (91/96)	V and W protein/99–124
26	HTL-MPV-2	NNGNVCLVSDAKMLSYAPEIAVSKED	12	19.79% (19/96)	V and W protein/144–169
27	HTL-MPV-2	PQTSRNVNLDSIKLYTSDDEEADQLEFEDEFAGSS SEVIVGISPEDEEPS	20	19.30% (11/57)	V protein/224–273

a The highly immunogenic 27 Ag-Patches were utilized to design two HTL (HTL-MPV-1 and HTL-MPV-2) multipatch vaccine candidates. The amino acid sequences of the identified GaEl Ag-Patches were highly conserved. HTL “GaEl Ag-Patches” from NIPAH: Supplementary txt 2 and Table S7.

#### Population Coverage by Antigenic Patches

The population coverage by the GaEl antigenic patches was analyzed by the overlapping epitopes and their HLA allele binding pairs using the “Population Coverage” tool of IEDB. The 57 GaEL Ag-Patches constructed in this study from 362 CTL and 414 HTL overlapping epitopes target a total of discrete 27 HLA class I and 25 HLA class II alleles (Table S5). Because HLA alleles are differentially expressed in the ethnically distributed global population, they provide us with the opportunity to analyze population coverage. The convincing 99.71% (average: 85.79 and standard deviation: 20.73) of the global human population is predicted to be covered by the CTL and HTL multipatch vaccine candidates proposed in this study. The countries most affected by NiV infections showed a significant coverage like India: 97.17%, Malaysia: 91.87%, etc. (Table S6). The epitope–HLA allele pairs are summarized in Supplementary txt 3.

#### Conservation Analysis of Antigenic Patches

The GaEl Ag-Patches were further analyzed for conservancy of their amino acid sequence. Up to 96 full-length NiV protein sequences of different strains and origins retrieved from NCBI protein database were analyzed with the “Epitope Conservancy Analysis” tool of IEDB. The CTL GaEl Ag-Patches were in the range of 43.59 to 100% (mostly above 90%), and HTL GaEl Ag-Patches were between 40.54 and 100% conservation (mostly above 90%) (Tables 1 and 2). Some of the assigned patches also have lower conservancy of around 19%; nevertheless, the variation in their amino acid sequences is limited to only a few residues.

### Multipatch Vaccines

#### Design of Multipatch Vaccines

**Figure 4:**
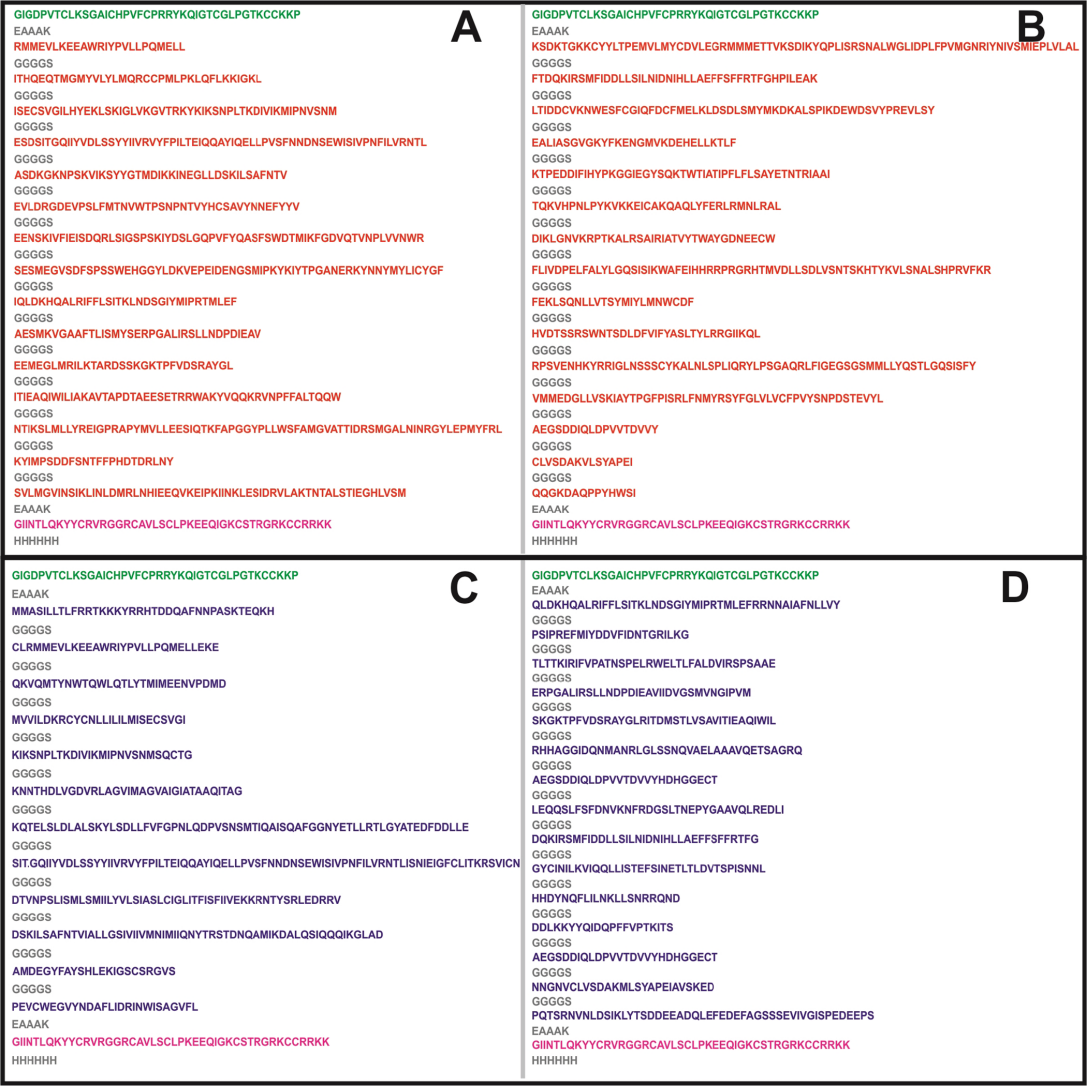
The CTL and HTL GaEl Ag-Patches are utilized as immunogenic composition to design multipatch vaccines. Short peptide linkers GGGGS and EAAK are used to fuse the GaEl Ag-Patches. The CTL MPV constructs include (A) CTL-MPV-1 and (B) CTL-MPV-2. The HTL MPV constructs include (C) HTL-MPV-1 and (D) HTL-MPV-2 (Table S7).

The GaEl Ag-Patches identified from the proteome of NiV were utilized as immunogenic composition for the design of two CTL and two HTL MULTIPatch vaccines (Figure 4, Table S7). Short amino acid linkers EAAAK and GGGGS provide a covalent and flexible connection between the constituting peptide, respectively. The EAAAK linker facilitates domain formation, and the GGGGS linker provides conformational flexibility; hence, together, they favor stable protein folding. The EAAAK linker was used to fuse the human β defensin 2 and 3 (hBD-2 and hBD-3) at N and C terminal ends of the MPVs, respectively. The constructs include hBD-2 with sequence: GIGDPVTCLKSGAICHPVFCPRRYKQIGTCGLPGTKCCKKP and 3D structure deposited in the Protein Data Bank (PDB code 1FD3) and hBD-3 with sequence: GIINTLQKYYCRVRGGRCAVLSCLPKEEQIGKCSTRGRKCCRRKK and 3D structure (PDB code 1KJ6). hBD 2 and 3 are utilized here as adjuvants to enhance the immunogenic response.^44−46,48,87−89^ The multipatch vaccine design and the method thereof are included in the field patents 202011037939, 202011037939, PCT/IN2021/050841, and previous publications.^40−43^

#### Physicochemical Property Analysis of the MPVs

ProtParam analysis was performed to analyze the physiochemical properties of all the suggested CTL and HTL MPVs. The empirical physiochemical properties of CTL and HTL MPVs are summarized in Table 3. Molecular weights of all MPVs range from 70.04 to 93.27 kDa. The expected half-life of the MPVs is predicted to be around ∼30 h in mammalian cells, suggesting stable expression products. The aliphatic index (81.80 to 96.13) and grand average of hydropathicity (GRAVY) (0.044 to −0.251) of the MPVs indicate a globular and hydrophilic nature. The instability index score of the MPVs (42.75 to 52.49) indicate the stable nature of the MPV protein molecules. Taken together, the physiochemical parameters of MPV candidates indicate a favorable expression of the designed MPVs in human cells (Table 3).

**Table 3:** Empirical Physicochemical Properties of Multipatch Vaccine Candidates

MPVs	length	molecular weight	theoretical pI	expected half-life	aliphatic index	grand average of hydropathicity (GRAVY)	instability index score
CTL-MPV-1	849 aa	93.27 kDa	8.83	*E. coli*: >10 h	84.26	–0.197	42.75
				yeast: >20 h			
				mammalian cell: 30 h			
CTL-MPV-2	783 aa	86.13 kDa	8.94	*E. coli*: >10 h	81.80	–0.177	44.11
				yeast: >20 h			
				mammalian cell: 30 h			
HTL-MPV-1	646 aa	70.04 kDa	8.64	*E. coli*: >10 h	96.13	0.044	46.45
				yeast: >20 h			
				mammalian cell: 30 h			
HTL-MPV-2	663 aa	70.63 kDa	5.45	*E. coli*: >10 h	83.09	–0.251	52.49
				yeast: >20 h			
				mammalian cell: 30 h			

#### Interferon-Gamma Inducing Epitope Prediction from the MPVs

Further, the MPV candidate molecules were also analyzed for the presence of potential interferon-gamma (IFN-γ) inducing epitopes. The amino acid sequences of all the MPV candidates were screened for IFN-γ inducing 15-mer peptide epitopes by utilizing the IFNepitope tool. A total of 75 IFN-γ inducing epitopes were screened from two CTL MPVs and two HTL MPVs (Table S8 and S9).

#### Allergenicity and Antigenicity of MPV Candidates

The characterization of the immunogenic properties of the suggested MPV is a crucial step in selecting appropriate vaccine candidates. We investigated both the *allergenicity and* the *antigenicity* using the bionformatic tools AllergenFP and Vaxijen, respectively. Note that all the MPV candidates were found to be good potential antigens and nonallergens (Table S10).

#### Tertiary Structure Modeling and Refinement of MPVs

**Figure 5:**
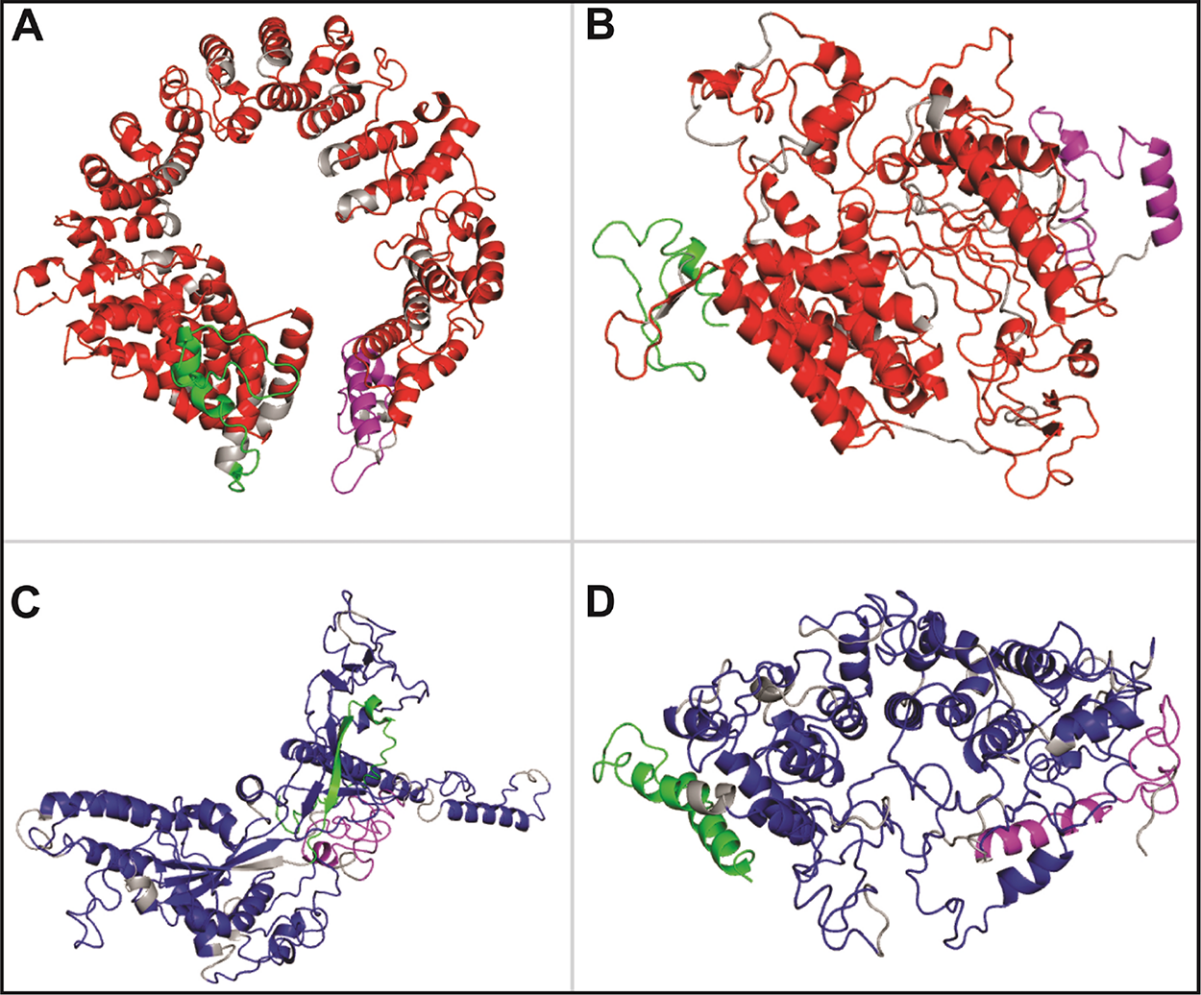
Tertiary structure modeling of multipatch vaccine candidates. The tertiary structural models of CTL and HTL MPVs are shown: (A) CTL-MPV-1, (B) CTL-MPV-2, (C) HTL-MPV-1, and (D) HTL-MPV-2. The GaEl Ag-Patches are shown in red (CTL GaEl Ag-Patches) and blue (HTL GaEl Ag-Patches). Linkers are shown in gray, and adjuvants are in green and magenta.

Tertiary structure homology models of MPV candidates were generated by using the I-TASSER modeling tool (Figure 5). All the parameters, viz., C-score, TM-Score, and RMSD of the homology modeling show acceptable values for all the MPV models (Table S11). The C-score is a confidence score indicating the significance of template alignments and the convergence parameters of structure assembly simulations. The C-score typically ranges from −5 to 2, with higher values indicating a model with higher confidence and vice versa. The C-scores of all the four MPV models are acceptable, indicating high confidence on the generated models. The TM-score indicates structural alignment between the query structure and template structure. The RMSD (root mean square deviation) is the deviation between the residues that are structurally aligned (by TM-align) to the template structure.

All the generated MPV tertiary models were refined with the GalaxyRefine tools to improve the sterical conformation parameters of the calculated structures. We used for further analysis only the top-scoring models. All selected MPV refinement output models have good Rama favored, GDT-HA, RMSD value, and MolProbity scores (Table S12). The Rama favored criterion indicates the percentage of residues that are in the favored region of the Ramachandran plot, the GDT-HA (global distance test-high accuracy) indicates the accuracy of the model structure backbone, and the RMSD (root mean square deviation) value indicates the deviation from the initial model. Further, the MolProbity score indicates the log-weighted combination score of the clash score, the percentage of Ramachandran not favored residues, and the percentage of bad side-chain rotamers.

Overall, after structural refinement, the MPV candidates had sterical parameters in the acceptable range; and hence, all the MPV models were carried forward for further analysis.

#### Validation of CTL and HTL MPV Refined Models

We next analyzed the sterical acceptable conformation of all MPV models using Ramachandran plots. The Ramachandran Plot analysis indicates that the MPV models (CTL-MPV-1 and CTL-MPV-2) have 95.5 and 94.9% residues in the favored region. Likewise, the HTL MPV models (HTL-MPV-1 and HTL-MPV-2) were found to have 90.9 and 87.3% residues in the favored region (Figure S2). Hence, all the MPV models show acceptable sterical conformations.

#### Linear and Discontinuous B-Cell Epitope Prediction from the MPVs

The tertiary models of MPV candidates were tested for the presence of potential linear and discontinuous B-cell epitopes with the ElliPro tool of IEDB. The screening revealed that the CTL MPVs carry a total of 39 linear epitopes [CTL-MPV-1 (21 epitopes) and CTL-MPV-2 (18 epitopes)]. Likewise, the HTL MPVs carry a total of 26 linear epitopes [HTL-MPV-1 (10 epitopes) and HTL-MPV-2 (16 epitopes)] (Table S13). Further, the CTL MPVs also carry 14 discontinuous B-cell epitopes [CTL-MPV-1 (3 epitopes) and CTL-MPV-2 (11 epitopes)]. Likewise, the HTL MPVs carry 15 potential discontinuous epitopes [HTL-MPV-1 (7 epitopes) and HTL-MPV-2 (8 epitopes)] (Table S14). The ElliPro associates its prediction with a PI (protrusion index) value. The high PI value of the linear and discontinuous epitopes suggests MPVs to have high potential to cause humoral immune response.

#### Molecular Interaction Analysis of MPVs with the Immune Receptor

**Figure 6:**
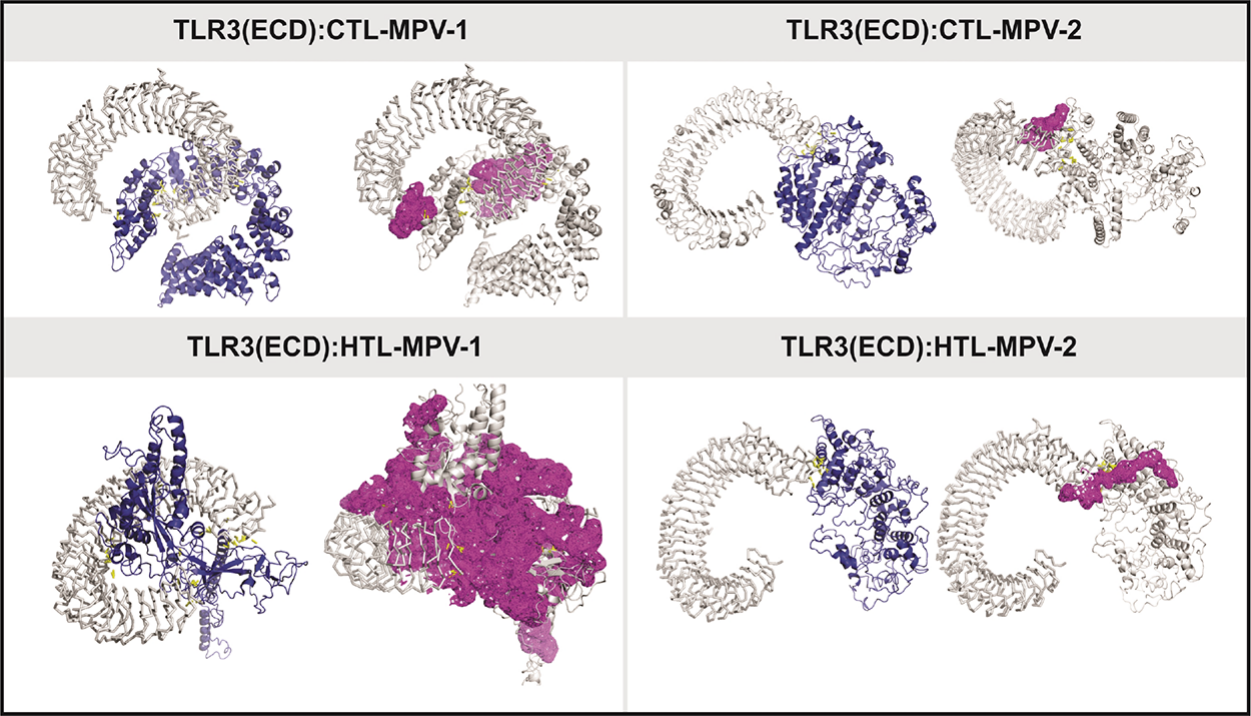
Molecular docking study of MPVs with TLR3-ECD. Complex formation by the MPVs (CTL-MPV-1, CTL-MPV-2, HTL-MPV-1, HTL-MPV-2) and TLR3-ECD is shown in different panels. In each panel, the B-factor analysis for the MPVs in complex with TLR3-ECD is shown in VIBGYOR color, with violet showing low and red showing high B-factor. Here, most of the MPV regions are in blue, indicating a low B-factor, hence suggesting a stable complex formation. The hydrogen bonds and the polar contacts are shown by yellow dots. Further, in each panel, the binding site clefts between MPVs and TLR3-ECD are shown by the magenta surface. The binding site clefts are generated by PDB*sum*. Detailed molecular interaction between MPVs and TLR3-ECD is shown in Figure S3.

The innate immune system Toll-like receptor 3 (TLR3) acts as a sentinel against foreign antigens. For those reasons, the molecular interaction of MPVs with the ectodomain (ECD) of the human TLR3 is an important aspect to study for a potential vaccine candidate. Therefore, the MPV candidates were docked with the ectodomain of human TLR3. The molecular docking study for the MPV models using a TLR3-ECD crystal structure (PDB ID: 2A0Z) was performed with the GRAMM-X Protein–Protein Docking tool of the Vakser Lab, Center for Bioinformatics at KU (Figure 6).

The B-factor analysis of the MPV and TLR3-ECD complexes indicates displacement of atomic positions from an average (mean) value of the structure (Figure 6). The B-factor analysis of MPV and TLR3-ECD complexes shows that most of the antigenic regions bound to TLR3-ECD are stable. The B-factor analysis was performed using the VIBGYOR color presentation with violet representing low and red representing high B-factor. Most of the regions of MPVs and TLR3-ECD complex are in blue, suggesting stable complex formation.

The docking studies of MPVs with TLR3-ECD indicated multiple binding sites as represented by the different complex structures in Figure 6 and Figure S3. These complexes form multiple hydrogen bonds in the interaction interface (Figure 6). The residues forming hydrogen bonds, salt bridges, disulfide bonds, and nonbonded contacts are shown in Figure S3.

#### Molecular Dynamics (MD) Simulations Study of MPV and TLR3 Ectodomain Complexes

The MPV/TLR3-ECD complexes were subjected to molecular dynamics (MD) simulation analysis with the program GROMACS version 2021.4 to investigate their stability. The complexes have very convincing root mean square deviation (RMSD) values for the backbone that gradually stabilize toward the end of 150 ns of MD simulation (CTL-MPV-1:TLR3-ECD, RMSD: ∼1 Å; CTL-MPV-2:TLR3-ECD, RMSD: ∼1 Å; HTL-MPV-1:TLR3-ECD, RMSD: ∼0.4 to ∼1.2 Å; HTL-MPV-2:TLR3-ECD, RMSD: ∼0.4 to ∼1.4 Å) (Figure S4). In summary, all the four simulated complexes were found to form very stable interactions with TLR3-ECD at constant temperature (∼300 K) and pressure (∼1 atm). Further, the variation in the radius of gyration (Rg) for all the MPV and TLR3-ECD complexes remains in the acceptable range throughout the MD simulation (CTL-MPV-1:TLR3-ECD, Rg: ∼4.5 to ∼7.5 Å; CTL-MPV-2:TLR3-ECD, Rg: ∼4.5 to ∼7.5 Å; HTL-MPV-1:TLR3-ECD, Rg: ∼3.55 to ∼3.85 Å; HTL-MPV-2:TLR3-ECD, Rg: ∼4.0 to ∼4.8 Å) (Figure S4). The stable Rg indicates the presence of structurally compact complexes throughout the MD simulation. Furthermore, the amino acid residues of the complexes show acceptable root mean square fluctuation (RMSF) from their respective initial confirmation (CTL-MPV-1:TLR3-ECD, RMSF: ∼2 to ∼14 Å; CTL-MPV-2:TLR3-ECD, RMSF: ∼1 to ∼6 Å; HTL-MPV-1:TLR3-ECD, RMSF: ∼0.25 to ∼2.25 Å; HTL-MPV-2:TLR3-ECD, RMSF: ∼0.2 to ∼1.2 Å) (Figure S4). Overall, the molecular docking and the molecular dynamics simulation study of all the MPV and TLR3-ECD complexes indicate stable complexes formed.

#### Analysis of Complementary DNA of MPV Candidates

The MPV construct was analyzed for expression feasibility in the human cell line. Codon-optimized *complementary* DNA (cDNA) of all the MPVs was generated for expression in the human cell line by the Java Codon Adaptation Tool. The optimized cDNA was further analyzed for its expression feasibility by the GenScript Rare Codon Analysis Tool. The GC content, the CAI score, and the tandem rare codon frequency optimized MPV cDNA were observed to be in the acceptable rage (Table S15). The CAI score indicates a high propensity of cDNA expression in the human cell expression system for PMV constructs. The tandem rare codons that may hinder the proper expression of the cDNA in the chosen expression system were observed to be 0% in all the MPVs. Therefore, as per the GenScript Rare Codon analysis, the cDNA of all the MPVs is predicted to have a high potential for large-scale expression in the human cell line.

## Discussion

Majority of the vaccines designed against NiV are focused on the single protein like G and F proteins, protein subunits, or the epitopes from NiV proteins.^27,32,36,37,84,85^ The recent strategies for the design and development of vaccines to combat NiV involve subunit vaccines or multiepitope vaccines. The subunit vaccine involves the use of a single protein or multiple subunits of NiV proteins. The major limitation with those vaccine candidates is their limited efficiency being composed of single/limited number of protein/subunits. The more recent multiepitope vaccine approach provides an opportunity to target multiple proteins of NiV. However, the low probability of MEV epitopes to be successfully presented by APC after the intercellular chop down process limits the efficacy of the MEV candidates. Moreover, the multiepitope vaccines can accommodate only a limited number of epitopes, causing them to face the challenge of frequent mutations in the proteome of the NiV.

In the present study, we have utilized the novel “reverse epitomics” approach to design multipatch vaccines.^40−43,90^ The consensus amino acid sequence of overlapping epitopes is identified as the GaEl Ag-Patch. This method is termed as the “overlapping-epitope-clusters-to-patches” method. We have identified potential GaEl Ag-Patches from the entire proteome of NiV. All the identified GaEl Ag-Patches are observed to arise from the surface of the NiV proteins, providing a potential target for immunogenic response. Further, we utilized the GaEl Ag-Patch as immunogenic composition of the multipatch vaccine against NiV. Hence, these GaEl Ag-Patches cover a large number of overlapping epitopes that they could produce upon the proteolytic intracellular chop down process by APC. These shortlisted GaEl Ag-Patches originate from all the proteins encoded by NiV, thus targeting its entire proteome.^89^ These advantages of the multipatch strategy would enhance the vaccine efficiency and lead to vaccines that are more targeted/specific and effective. Because the GaEl Ag-Patches cover a large number of epitopes that in turn target a large number of different numbers of HLA alleles, the MPV would also provide a larger ethnically distributed human population coverage. Here, the designed MPVs cover 99.71% of the world population, targeting 52 different HLA class I and class II alleles, a coverage that is much higher in comparison to vaccines composed of single/limited number of protein/subunits/epitopes. In addition to potential immunogenic composition for vaccine candidates, the reported GaEl Ag-Patches also provide potential immunogenic composition for early detection diagnostic kits against NiV infection. Further, the MPVs show stable binding with TLR3-ECD, which is one of the essential criteria for an antigen to be recognized and processed by the human immune system. The physicochemical properties and cDNA analysis of MPVs favor their overexpression in human cell lines.

## Conclusions

In the present study, we have identified highly immunogenic novel GaEl antigenic patches (GaEl Ag-Patches) (30 CTL and 27 HTL) from the entire proteome of NiV. We have utilized the novel “reverse epitomics” approach involving the “overlapping-epitope-clusters-to-patches” method. These GaEl Ag-Patches are highly evolutionarily conserved in nature. Further, for the first time, we have identified the GaEl Ag-Patches and used them as immunogenic composition to design a multipatch vaccine (MPV) against NiV. The MPVs designed against NiV in our study have potential to give rise to a total of 776 discrete CTL and HTL epitopes targeting a total of discrete 52 HLA alleles and hence covering a convincing 99.71% of the world human population. Such large number of epitopes is not possible to accommodate in multiepitope vaccines. We conclude that the multipatch vaccine designed by the novel “reverse epitomics” approach utilizing GaEl Ag-Patches as immunogenic composition could be highly potent with greater effectiveness and high specificity with large human population coverage worldwide and protect the human population against NiV infection in an effective manner. The reported GaEl Ag-Patches also provide potential candidates for early detection diagnostic kits for NiV infection. The future prospects would involve production and animal model based validation of our MPVs to be potential vaccine candidates for NiV.

## Supporting Information Available

Figure S1. Schematic representation of workflow and methodology. Figure S2. Ramachandran plot analysis for all the MPVs: (A) CTL-MPV-1, (B) CTL-MPV-2, (C) HTL-MPV-1, (D) HTL-MPV-2. Figure S3. Molecular interaction between MPVs and TLR3 ectodomain: (A) CTL-MPV-1, (B) CTL-MPV-2, (C) HTL-MPV-1 and (D) HTL-MPV-2. Figure S4. Molecular dynamics simulation study of the MPVs and TLR3-ECD complexes. The root mean square deviation (RMSD) for Cα, backbone, radius of gyration for all the MPVs and TLR3-ECD complexes, and the root mean square fluctuation (RMSF) in the conformation of residues of the MPVs in complex with TLR3-ECD are shown. Table S1: High percentile ranking CTL epitopes–HLA allele pairs screened from the entire proteome of SARS-CoV-2 by the “MHC-I Processing Predictions” tool of IEDB. These epitopes were further utilized to identify the potentially immunogenic multiple epitope cluster based CTL Ag-Patches from the entire proteome of the SRAS-CoV-2. The screened epitopes are in consensus with previous studies.^37^ Table S2: High-scoring CTL epitopes–HLA allele pairs screened from the entire proteome of SARS-CoV-2 by the “MHC-I Binding Predictions” tool of IEDB. These epitopes were further utilized to identify the potentially immunogenic multiple epitope cluster based CTL Ag-Patches from the entire proteome of the SRAS-CoV-2. The screened epitopes are in consensus with previous studies.^37^ Table S3: High percentile ranking HTL epitopes–HLA allele pairs screened from the entire proteome of SARS-CoV-2 by the “MHC-II Binding Predictions” tool of IEDB. These epitopes were further utilized to identify the potentially immunogenic multiple epitope cluster based HTL Ag-Patches from the entire proteome of the SRAS-CoV-2. The screened epitopes are in consensus with previous studies.^37^ Table S4. Homology models of the full-length protein tertiary structure of NIPAH virus. Nipah protein sequences were retrieved from NCBI. The homology modeling tools SwissModel and I-TASSER were used. Table S5: HLA alleles covered by the overlapping CTL and HTL epitopes. Table S6: Population coverage by all the overlapping CTL and HTL epitopes forming epitope clusters. Table S7: Construct of CTL-MPV-1, CTL-MPV-2, CTL-MPV-3, HTL-MPV-1, and HTL-MPV-2. Physicochemical property analysis based on the amino acid sequences of all the designed three CTL and two HTL multipatch vaccines. Table S8: INF-γ inducing positive epitopes with a score of 1 or more than 1, screened from the CTL MPVs. Table S9: INF-γ inducing positive epitopes with a score of 1 or more than 1, screened from the HTL MPVs. Table S10: AllergenFP and Vaxijen analysis of MPVs. For the Vaxijen, the default threshold is 0.4, and here, all the MPVs have scored above 0.4, indicating a potential antigenic nature. Table S11: Parameters for the tertiary structure homology modeling of all the CTL and HTL MPVs by the I-TASSER tool. Table S12: Refinement parameter values for CTL and HTL MPV models after refinement by the GalaxyRefine tool. The RMSD value in Å indicates deviation from the initial model. GDT-HA (global distance test-high accuracy): backbone structure accuracy measured by GDT-HA. Table S13: B-cell linear epitopes screened from CTL and HTL MPVs. Table S14: B-cell discontinuous epitopes screened from CTL MPVs. Table S15: Analysis of codon-optimized cDNA of all the MPVs. Supplementary txt 1: CTL “GaEl Ag-Patches” from NIPAH proteome. Supplementary txt 2: HTL “GaEl Ag-Patches” from NIPAH Proteome. Supplementary txt 3: Overlapping epitopes and HLA allele pairs (PDF)

## Notes

The novel term “GaEl antigenic patch” is introduced. Three patents are used with due permissions. The study is part of patent proceedings. All rights reserved.
