# Supplementary material for: Novel “GaEl Antigenic Patches” Identified by a “Reverse Epitomics” Approach to Design Multipatch Vaccines against NIPAH Infection, a Silent Threat to Global Human Health
Source: ACS Omega. 2023 Aug 22;8(35):31698–713. doi: 10.1021/acsomega.3c01909 (PMC10483669; doi:10.1021/acsomega.3c01909)
Supplement: Supplementary file 1 — ao3c01909_si_001.pdf [file ao3c01909_si_001.pdf]

Novel 'GaEl antigenic patches' identified by 'reverse epitomics' approach to design multi-patch vaccines against NIPAH infection, a silent threat to global human health

Sukrit Srivastava*[a,b], Michael Kolbe*[b,c]

a Infection Biology Group, Indian Foundation for Fundamental Research Trust, RaeBareli, Uttar Pradesh, India

b Department for Structural Infection Biology, Centre for Structural Systems Biology (CSSB) & Helmholtz-Centre for Infection Research, Notkestraße 85, 22607 Hamburg, Germany.

c Faculty of Mathematics, Informatics and Natural Sciences, University of Hamburg, Rothenbaumchaussee 19, 20148 Hamburg, Germany.

*Correspondence should be addressed to S.S. (srivastava.sukrit@iffr.in) or M.K. (Michael.kolbe@helmholtz-hzi.de)

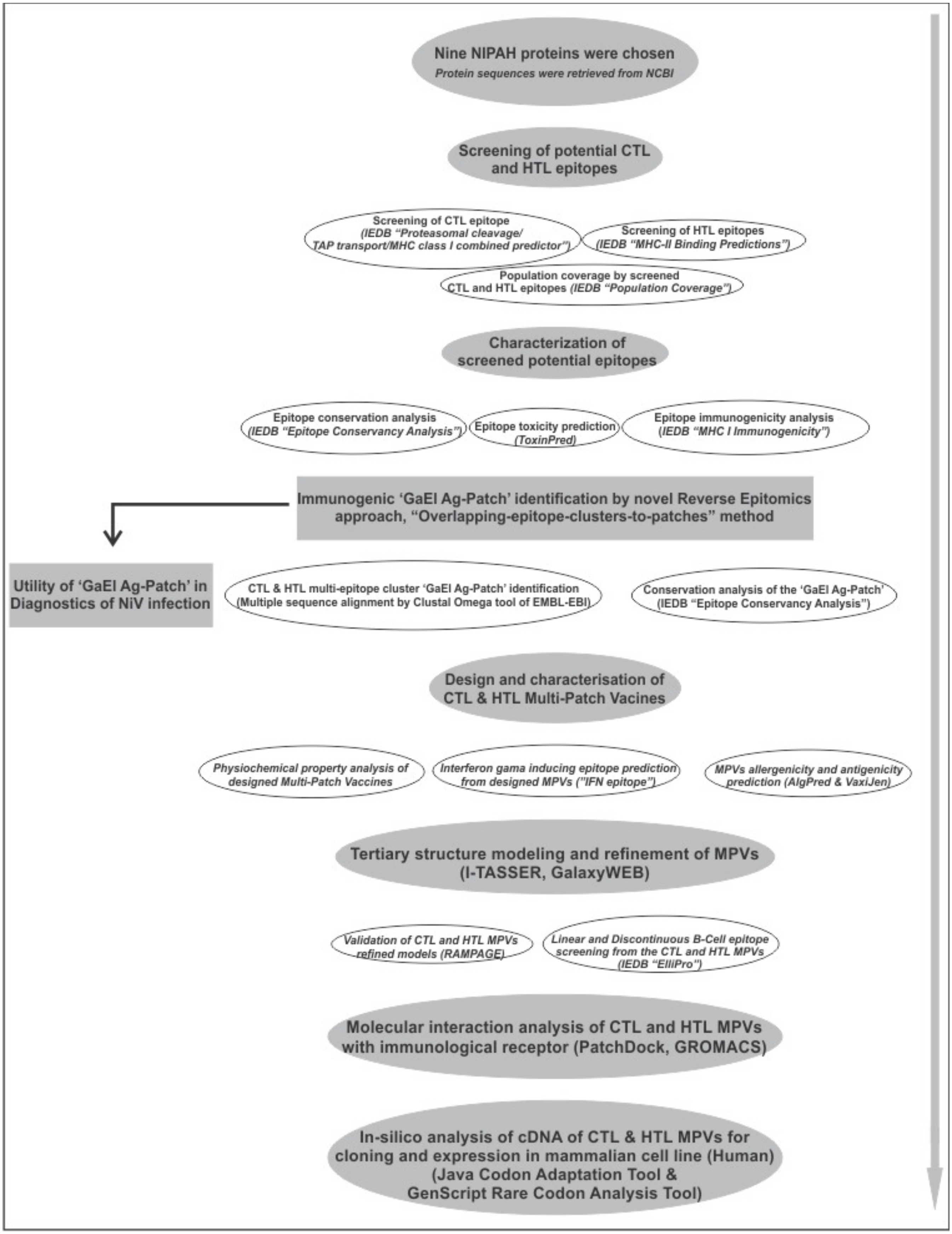

Figure S1. Schematic representation of workflow and methodology.

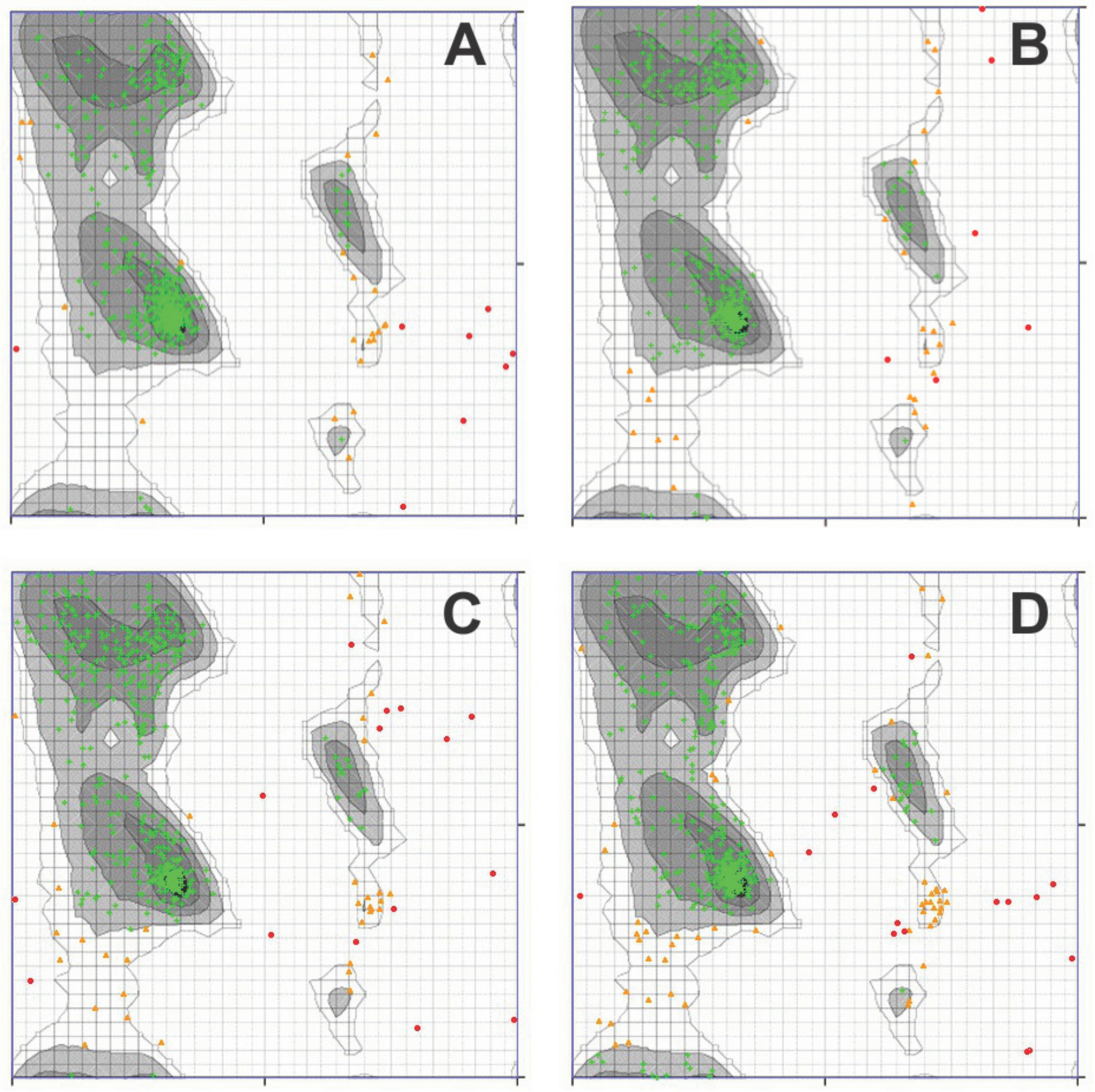

#	Ramachandran Plot	(A) CTL-MPV-1	(B) CTL-MPV-2	(C) HTL-MPV-1	(D) HTL-MPV-2
1	Highly Preferred (GREEN)	676 (95.5%)	620 (94.9%)	500 (90.9%)	476 (87.3%)
2	Preferred (BROWN)	24 (3.4%)	27 (4.1%)	35 (6.4%)	54 (9.9%)
3	Questionable (RED)	8 (1.1%)	6 (0.9%)	15 (2.7%)	15 (2.8%)

Figure S2. Ramachandran Plot analysis for all the MPVs **(A)** CTL-MPV-1, **(B)** CTL-MPV-2, **(C)** HTL-MPV-1, **(D)** HTL-MPV-2.

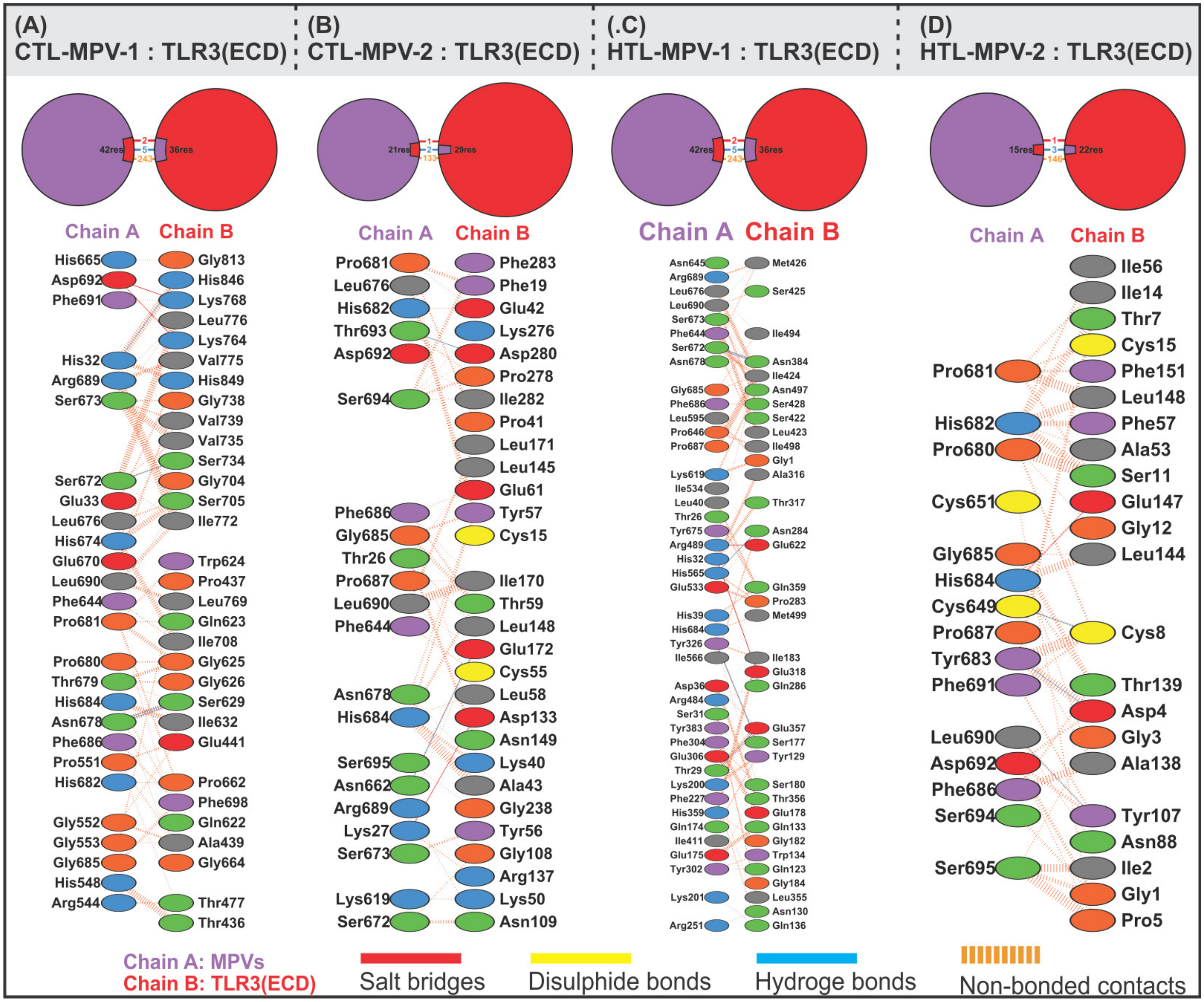

#	MPV:TLR3(ECD) Bonds	(A) CTL-MPV-1	(B) CTL-MPV-2	(C) HTL-MPV-1	(D) HTL-MPV-2
1	Hydrogen Bonds	5	2	5	3
2	Salt Bridge	2	1	2	1
3	Disulphide bonds	0	0	0	0
4	Non-Bonded contacts	243	133	243	146

Figure S3. Molecular interaction between MPVs (chain A) and TLR3 Ectodomain **(A)** CTL-MPV-1, **(B)** CTL-MPV-2, **(C)** HTL-MPV-1 and **(D)** HTL-MPV-2 (chain B).

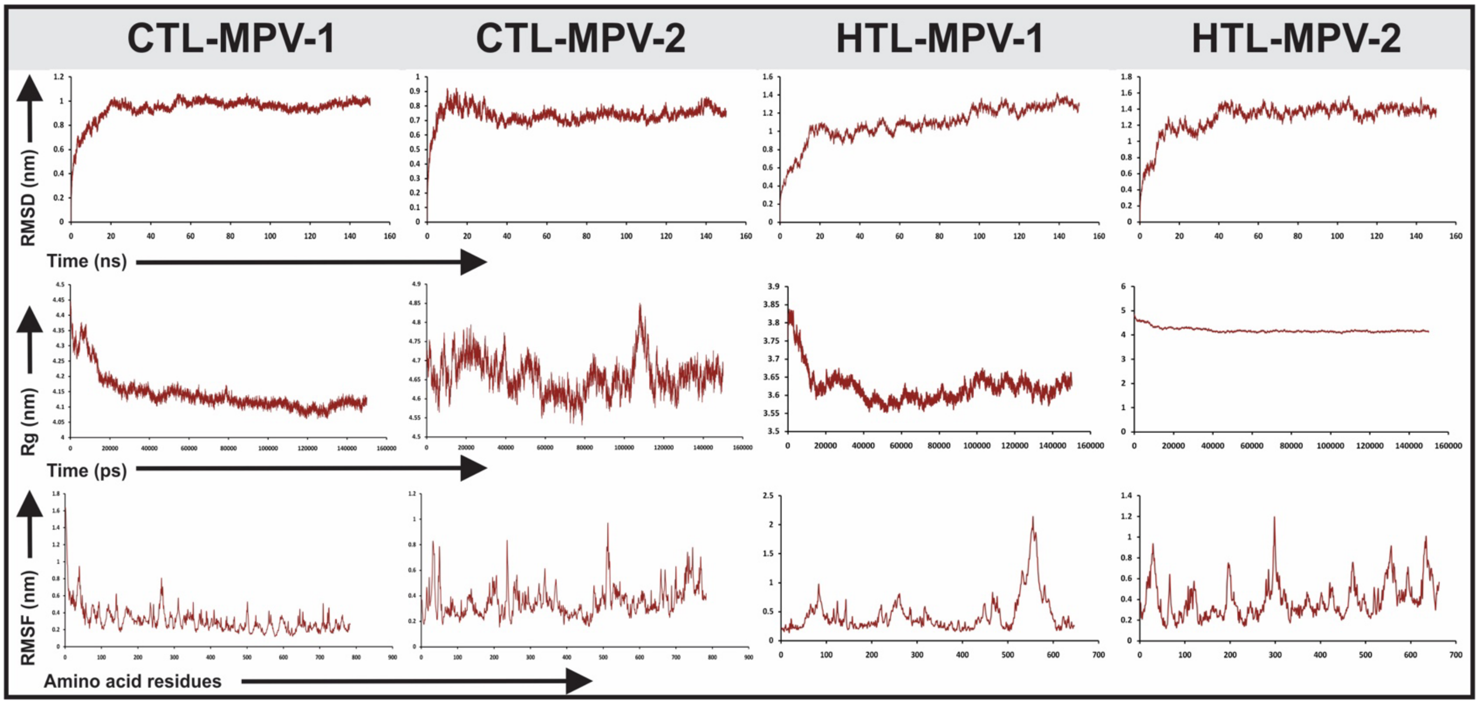

Figure S4. Molecular Dynamics simulation study of the MPVs and TLR3-ECD complexes. The Root Mean Square Deviation (RMSD) for Cα, Backbone, the Radius of gyration for all the MPVs and TLR3-ECD complexes and the Root Mean Square Fluctuation (RMSF) in the conformation of residues of the MPVs in complex with TLR3-ECD are shown.

Table S1: High Percentile Ranking CTL epitopes-HLA allele pairs screened from entire proteome of SARS-CoV-2 by the "MHC-I Processing Predictions" tool of IEDB. These epitopes were further utilized to identify the potentially immunogenic multiple epitope cluster based CTL Ag-Patches from the entire proteome of the SRAS-CoV-2. The screened epitopes are in consensus with the previous studies[38].

#	Protein	Peptide	Allele	Length	Immunogenicity	Conservancy	Toxicity	Proteasome Score	TAP Score	MHC Score	Processing Score	Total Score	MHC IC50[nM]
1	c protein	ASILLTLFR	HLA-A*11:01	9	0.11682	100.00% (60/60)	Non-Toxin	1.27	0.77	-1.33	2.04	0.71	21.4
2	c protein	ASILLTLFR	HLA-A*68:01	9	0.11682	100.00% (60/60)	Non-Toxin	1.27	0.77	-1.48	2.04	0.55	30.5
3	c protein	ASILLTLFR	HLA-A*31:01	9	0.11682	100.00% (60/60)	Non-Toxin	1.27	0.77	-1.5	2.04	0.53	31.8
4	c protein	EQTMGMYVLY	HLA-A*26:01	10	-0.27722	100.00% (60/60)	Non-Toxin	1.3	1.18	-1.34	2.47	1.14	21.8
5	c protein	EQTMGMYVLY	HLA-A*68:01	10	-0.27722	100.00% (60/60)	Non-Toxin	1.3	1.18	-1.74	2.47	0.74	54.6
6	c protein	MASILLTLF	HLA-B*58:01	9	0.08526	100.00% (60/60)	Non-Toxin	1.2	1.16	-1.04	2.35	1.31	11
7	c protein	MASILLTLF	HLA-B*35:01	9	0.08526	100.00% (60/60)	Non-Toxin	1.2	1.16	-1.26	2.35	1.1	18.1
8	c protein	MASILLTLF	HLA-B*53:01	9	0.08526	100.00% (60/60)	Non-Toxin	1.2	1.16	-1.56	2.35	0.8	35.9
9	c protein	MASILLTLF	HLA-B*15:01	9	0.08526	100.00% (60/60)	Non-Toxin	1.2	1.16	-1.78	2.35	0.57	60.9
10	c protein	MASILLTLFR	HLA-A*68:01	10	0.1542	100.00% (60/60)	Non-Toxin	1.27	0.7	-0.72	1.97	1.25	5.2
11	c protein	MASILLTLFR	HLA-A*31:01	10	0.1542	100.00% (60/60)	Non-Toxin	1.27	0.7	-1.4	1.97	0.57	25.1
12	c protein	MEMLKEETW	HLA-B*44:02	9	-0.07673	21.67% (13/60)	Non-Toxin	1.82	0.39	-1.4	2.21	0.8	25.2
13	c protein	MEMLKEETW	HLA-B*44:03	9	-0.07673	21.67% (13/60)	Non-Toxin	1.82	0.39	-1.54	2.21	0.67	34.7
14	c protein	MLKEETWRIY	HLA-B*15:01	10	0.496	21.67% (13/60)	Non-Toxin	1.29	1.23	-1.72	2.51	0.79	52.4
15	c protein	MMASILLTL	HLA-A*02:03	9	-0.02129	100.00% (60/60)	Non-Toxin	1.45	0.45	-0.61	1.9	1.29	4.1
16	c protein	MMASILLTL	HLA-A*02:01	9	-0.02129	100.00% (60/60)	Non-Toxin	1.45	0.45	-0.75	1.9	1.15	5.6
17	c protein	MMASILLTL	HLA-A*02:06	9	-0.02129	100.00% (60/60)	Non-Toxin	1.45	0.45	-0.9	1.9	1	7.9
18	c protein	MMASILLTL	HLA-A*32:01	9	-0.02129	100.00% (60/60)	Non-Toxin	1.45	0.45	-1.11	1.9	0.79	12.9
19	c protein	MMASILLTL	HLA-B*15:01	9	-0.02129	100.00% (60/60)	Non-Toxin	1.45	0.45	-1.38	1.9	0.52	23.9
20	c protein	MMASILLTLF	HLA-B*15:01	10	-0.01913	100.00% (60/60)	Non-Toxin	1.2	1.13	-0.76	2.33	1.57	5.8
21	c protein	MMASILLTLF	HLA-A*02:03	10	-0.01913	100.00% (60/60)	Non-Toxin	1.2	1.13	-1.37	2.33	0.96	23.6
22	c protein	MMASILLTLF	HLA-B*58:01	10	-0.01913	100.00% (60/60)	Non-Toxin	1.2	1.13	-1.53	2.33	0.8	33.7
23	c protein	MMASILLTLF	HLA-A*02:01	10	-0.01913	100.00% (60/60)	Non-Toxin	1.2	1.13	-1.71	2.33	0.62	51.5
24	c protein	MMASILLTLF	HLA-A*32:01	10	-0.01913	100.00% (60/60)	Non-Toxin	1.2	1.13	-1.77	2.33	0.56	59.5
25	c protein	MMASILLTLF	HLA-A*23:01	10	-0.01913	100.00% (60/60)	Non-Toxin	1.2	1.13	-1.8	2.33	0.53	62.6
26	c protein	MMASILLTLFR	HLA-A*68:01	11	0.04981	100.00% (60/60)	Non-Toxin	1.27	0.68	-1.25	1.95	0.7	17.6
27	c protein	MMEMLKEETW	HLA-B*58:01	10	-0.16357	21.67% (13/60)	Non-Toxin	1.82	0.45	-1.73	2.27	0.53	54.1
28	c protein	MTYNWTQW	HLA-B*58:01	8	0.14677	95.00% (57/60)	Non-Toxin	1.24	0.45	-1.17	1.7	0.53	14.8
29	c protein	MTYNWTQWL	HLA-A*68:02	9	0.27619	95.00% (57/60)	Non-Toxin	1.5	0.49	-1.16	1.99	0.83	14.4
30	c protein	MTYNWTQWL	HLA-A*32:01	9	0.27619	95.00% (57/60)	Non-Toxin	1.5	0.49	-1.24	1.99	0.75	17.5
31	c protein	MYVLYLMQR	HLA-A*33:01	9	-0.22768	100.00% (60/60)	Non-Toxin	1	0.8	-1.08	1.8	0.72	12
32	c protein	NWTQWLQTLY	HLA-A*01:01	10	0.01818	100.00% (60/60)	Non-Toxin	1.29	1.35	-2.15	2.64	0.5	139.8
33	c protein	QEQTMGMYVLY	HLA-B*44:03	11	-0.28266	100.00% (60/60)	Non-Toxin	1.3	1.19	-1.95	2.49	0.54	88.5
34	c protein	QTMGMYVLY	HLA-A*11:01	9	-0.16902	100.00% (60/60)	Non-Toxin	1.3	1.22	-1.4	2.52	1.12	25
35	c protein	QTMGMYVLY	HLA-A*68:01	9	-0.16902	100.00% (60/60)	Non-Toxin	1.3	1.22	-1.42	2.52	1.1	26.4
36	c protein	QTMGMYVLY	HLA-A*30:02	9	-0.16902	100.00% (60/60)	Non-Toxin	1.3	1.22	-1.45	2.52	1.06	28.5
37	c protein	QTMGMYVLY	HLA-A*26:01	9	-0.16902	100.00% (60/60)	Non-Toxin	1.3	1.22	-1.52	2.52	1	32.9
38	c protein	QTMGMYVLY	HLA-A*32:01	9	-0.16902	100.00% (60/60)	Non-Toxin	1.3	1.22	-1.76	2.52	0.76	57.2
39	c protein	RMMEMLKEETW	HLA-B*58:01	11	-0.14662	21.67% (13/60)	Non-Toxin	1.82	0.59	-1.46	2.41	0.95	28.7
40	c protein	WTQWLQTLY	HLA-A*01:01	9	0.09173	100.00% (60/60)	Non-Toxin	1.29	1.3	-1.24	2.59	1.34	17.5
41	c protein	WTQWLQTLY	HLA-A*30:02	9	0.09173	100.00% (60/60)	Non-Toxin	1.29	1.3	-1.61	2.59	0.98	40.3
42	c protein	YLMQRCCPML	HLA-B*08:01	10	-0.33837	100.00% (60/60)	Non-Toxin	1.41	0.47	-1.1	1.87	0.77	12.6
43	c protein	YLMQRCCPML	HLA-A*02:03	10	-0.33837	100.00% (60/60)	Non-Toxin	1.41	0.47	-1.23	1.87	0.65	16.9

#	Protein	Peptide	Allele	Length	Immunogenicity	Conservancy	Toxicity	Proteasome Score	TAP Score	MHC Score	Processing Score	Total Score	MHC IC50[nM]
44	c protein	YLMQRCCPML	HLA-A*02:01	10	-0.33837	100.00% (60/60)	Non-Toxin	1.41	0.47	-1.29	1.87	0.59	19.3
45	fusion protein	AISQAFGGNY	HLA-A*30:02	10	0.03856	97.30% (36/37)	Non-Toxin	1.35	1.37	-1.72	2.71	0.99	52.6
46	fusion protein	AQITAGVALY	HLA-B*15:01	10	0.21876	97.30% (36/37)	Non-Toxin	1.18	1.46	-1	2.64	1.64	10
47	fusion protein	ETLLRTLGY	HLA-A*26:01	9	0.08262	97.30% (36/37)	Non-Toxin	1.61	1.16	-1.56	2.76	1.2	36.7
48	fusion protein	FALSNGVLF	HLA-B*35:01	9	-0.11611	100.00% (37/37)	Non-Toxin	1.49	1.15	-0.77	2.64	1.86	5.9
49	fusion protein	FALSNGVLF	HLA-B*53:01	9	-0.11611	100.00% (37/37)	Non-Toxin	1.49	1.15	-1.53	2.64	1.11	33.9
50	fusion protein	FPILTEIQQAY	HLA-B*35:01	11	0.113	100.00% (37/37)	Non-Toxin	1.17	1.1	-0.96	2.28	1.32	9.1
51	fusion protein	IIYVDLSSY	HLA-B*15:01	9	-0.18478	45.95% (17/37)	Non-Toxin	1.41	1.4	-1.63	2.8	1.17	42.8
52	fusion protein	IIYVDLSSY	HLA-A*30:02	9	-0.18478	45.95% (17/37)	Non-Toxin	1.41	1.4	-1.8	2.8	1.01	62.8
53	fusion protein	IIYVDLSSYY	HLA-A*30:02	10	-0.24637	45.95% (17/37)	Non-Toxin	1.24	1.4	-1.61	2.64	1.03	40.3
54	fusion protein	ISQAFGGNY	HLA-A*30:02	9	0.17249	97.30% (36/37)	Non-Toxin	1.35	1.35	-1.44	2.7	1.25	27.7
55	fusion protein	KYLSDLLFVF	HLA-A*23:01	10	-0.04679	97.30% (36/37)	Non-Toxin	1.55	1.29	-0.74	2.84	2.1	5.5
56	fusion protein	KYLSDLLFVF	HLA-A*24:02	10	-0.04679	97.30% (36/37)	Non-Toxin	1.55	1.29	-0.98	2.84	1.86	9.5
57	fusion protein	LVVSSHVPRF	HLA-B*15:01	10	-0.22293	100.00% (37/37)	Non-Toxin	1.49	1.12	-1.35	2.62	1.26	22.6
58	fusion protein	MTIQAISQAF	HLA-B*15:01	10	-0.13629	97.30% (36/37)	Non-Toxin	1.4	1.16	-0.92	2.57	1.64	8.4
59	fusion protein	MTIQAISQAF	HLA-A*26:01	10	-0.13629	97.30% (36/37)	Non-Toxin	1.4	1.16	-1.55	2.57	1.01	35.7
60	fusion protein	RFALSNGVLF	HLA-A*23:01	10	-0.1056	100.00% (37/37)	Non-Toxin	1.49	1.22	-1.29	2.71	1.43	19.3
61	fusion protein	RFALSNGVLF	HLA-A*24:02	10	-0.1056	100.00% (37/37)	Non-Toxin	1.49	1.22	-1.41	2.71	1.3	25.9
62	fusion protein	RVRPTSSGDLY	HLA-A*30:02	11	-0.23462	100.00% (37/37)	Non-Toxin	1.42	1.51	-1.67	2.94	1.26	47
63	fusion protein	SEWISIVPNF	HLA-B*44:03	10	0.20004	97.30% (36/37)	Non-Toxin	1.25	1.2	-1.23	2.45	1.22	17
64	fusion protein	SEWISIVPNF	HLA-A*23:01	10	0.20004	97.30% (36/37)	Non-Toxin	1.25	1.2	-1.29	2.45	1.15	19.6
65	fusion protein	SEWISIVPNF	HLA-B*44:02	10	0.20004	97.30% (36/37)	Non-Toxin	1.25	1.2	-1.44	2.45	1	27.7
66	fusion protein	SKYLSDLLFVF	HLA-A*23:01	11	-0.05018	97.30% (36/37)	Non-Toxin	1.55	1.25	-1.79	2.79	1	61.9
67	fusion protein	SYYIIVRVYF	HLA-A*23:01	10	0.38392	45.95% (17/37)	Non-Toxin	1.46	1.34	-1.45	2.8	1.35	28.2
68	fusion protein	YLSDLLFVF	HLA-A*02:01	9	0.0703	97.30% (36/37)	Non-Toxin	1.55	1.14	-1.52	2.69	1.16	33.3
69	fusion protein	YLSDLLFVF	HLA-A*02:06	9	0.0703	97.30% (36/37)	Non-Toxin	1.55	1.14	-1.54	2.69	1.15	34.6
70	fusion protein	YLSDLLFVF	HLA-B*15:01	9	0.0703	97.30% (36/37)	Non-Toxin	1.55	1.14	-1.62	2.69	1.07	41.6
71	fusion protein	YYIIVRVYF	HLA-A*23:01	9	0.29872	100.00% (37/37)	Non-Toxin	1.46	1.26	-1.63	2.72	1.08	43
72	Glycoprotein	AENPVFTVF	HLA-B*44:03	9	0.19402	100.00% (66/66)	Non-Toxin	1.71	1.07	-1.09	2.77	1.69	12.3
73	Glycoprotein	AENPVFTVF	HLA-B*44:02	9	0.19402	100.00% (66/66)	Non-Toxin	1.71	1.07	-1.21	2.77	1.56	16.3
74	Glycoprotein	AENPVFTVF	HLA-B*40:01	9	0.19402	100.00% (66/66)	Non-Toxin	1.71	1.07	-1.31	2.77	1.46	20.6
75	Glycoprotein	AMDEGYFAY	HLA-A*01:01	9	0.25913	90.91% (60/66)	Non-Toxin	1.43	1.33	-1.61	2.76	1.15	40.7
76	Glycoprotein	AVYNNEFYY	HLA-A*11:01	9	0.17688	90.91% (60/66)	Non-Toxin	1.21	1.5	-1.07	2.7	1.63	11.8
77	Glycoprotein	AVYNNEFYY	HLA-A*30:02	9	0.17688	90.91% (60/66)	Non-Toxin	1.21	1.5	-1.11	2.7	1.59	13
78	Glycoprotein	FLIDRINWI	HLA-A*02:03	9	0.36516	100.00% (66/66)	Non-Toxin	1.3	0.3	-0.28	1.6	1.32	1.9
79	Glycoprotein	FLIDRINWI	HLA-A*02:01	9	0.36516	100.00% (66/66)	Non-Toxin	1.3	0.3	-0.48	1.6	1.12	3
80	Glycoprotein	FLIDRINWI	HLA-A*02:06	9	0.36516	100.00% (66/66)	Non-Toxin	1.3	0.3	-0.49	1.6	1.1	3.1
81	Glycoprotein	ILKPKLISY	HLA-B*15:01	9	-0.28594	96.97% (64/66)	Non-Toxin	1.43	1.27	-1.52	2.7	1.18	33.4
82	Glycoprotein	ILRSGLLKY	HLA-B*15:01	9	-0.26247	98.48% (65/66)	Non-Toxin	1.44	1.29	-1.56	2.73	1.17	36.2
83	Glycoprotein	IPSKVIKSY	HLA-B*35:01	9	-0.38388	18.18% (12/66)	Non-Toxin	1.44	1.19	-1.34	2.63	1.29	21.7
84	Glycoprotein	LAMDEGYFAY	HLA-B*35:01	10	0.214	90.91% (60/66)	Non-Toxin	1.43	1.34	-0.85	2.77	1.93	7
85	Glycoprotein	LAMDEGYFAY	HLA-B*15:01	10	0.214	90.91% (60/66)	Non-Toxin	1.43	1.34	-1.55	2.77	1.22	35.7
86	Glycoprotein	LAMDEGYFAY	HLA-A*01:01	10	0.214	90.91% (60/66)	Non-Toxin	1.43	1.34	-1.72	2.77	1.05	52.9
87	Glycoprotein	LTVNPLVVNW	HLA-B*58:01	10	0.05521	19.70% (13/66)	Non-Toxin	1.73	0.43	-0.9	2.16	1.26	8
88	Glycoprotein	NIMIIQNYTR	HLA-A*68:01	10	0.10719	98.48% (65/66)	Non-Toxin	1.34	0.76	-1	2.1	1.09	10.1
89	Glycoprotein	SAVYNNEFY	HLA-B*35:01	9	0.15019	90.91% (60/66)	Non-Toxin	1.49	1.35	-1.68	2.84	1.16	47.7
90	Glycoprotein	SAVYNNEFYY	HLA-A*11:01	10	0.18797	90.91% (60/66)	Non-Toxin	1.21	1.35	-1.51	2.55	1.04	32.5
91	Glycoprotein	SAVYNNEFYY	HLA-A*30:02	10	0.18797	90.91% (60/66)	Non-Toxin	1.21	1.35	-1.56	2.55	0.99	36.3
92	Glycoprotein	SMGIRPNSHY	HLA-B*15:01	10	0.05771	96.97% (64/66)	Non-Toxin	1.18	1.3	-1.47	2.47	1	29.6

#	Protein	Peptide	Allele	Length	Immunogenicity	Conservancy	Toxicity	Proteasome Score	TAP Score	MHC Score	Processing Score	Total Score	MHC IC50[nM]
93	Glycoprotein	TVYHCSAVY	HLA-A*30:02	9	-0.11974	100.00% (66/66)	Non-Toxin	1.57	1.46	-1.51	3.03	1.52	32.6
94	Glycoprotein	TVYHCSAVY	HLA-B*35:01	9	-0.11974	100.00% (66/66)	Non-Toxin	1.57	1.46	-1.53	3.03	1.5	34.2
95	Glycoprotein	TVYHCSAVY	HLA-B*15:01	9	-0.11974	100.00% (66/66)	Non-Toxin	1.57	1.46	-1.56	3.03	1.47	36.2
96	Matrix protein	FSLMDINPWL	HLA-A*02:01	10	0.08487	94.87% (37/39)	Non-Toxin	1.53	0.4	-0.6	1.92	1.32	4
97	Matrix protein	FSLMDINPWL	HLA-A*02:06	10	0.08487	94.87% (37/39)	Non-Toxin	1.53	0.4	-0.78	1.92	1.14	6
98	Matrix protein	IYMIPRTMLEF	HLA-A*23:01	11	0.03816	97.44% (38/39)	Non-Toxin	1.4	1.24	-1.34	2.64	1.29	22.1
99	Matrix protein	IYMIPRTMLEF	HLA-A*24:02	11	0.03816	97.44% (38/39)	Non-Toxin	1.4	1.24	-1.55	2.64	1.09	35.5
100	Matrix protein	KYYSVDYCR	HLA-A*31:01	9	-0.14121	97.44% (38/39)	Non-Toxin	1.09	0.87	-0.93	1.96	1.03	8.6
101	Matrix protein	KYYSVDYCRR	HLA-A*31:01	10	-0.12461	97.44% (38/39)	Non-Toxin	1.21	0.87	-1.1	2.09	0.99	12.6
102	Matrix protein	NAIAFNLLVY	HLA-B*35:01	10	0.19459	84.62% (33/39)	Non-Toxin	1.25	1.4	-1.55	2.65	1.1	35.3
103	Matrix protein	NYMYLICYGF	HLA-A*23:01	10	0.02401	97.44% (38/39)	Non-Toxin	1.29	1.32	-1.09	2.61	1.52	12.2
104	Matrix protein	NYMYLICYGF	HLA-A*24:02	10	0.02401	97.44% (38/39)	Non-Toxin	1.29	1.32	-1.34	2.61	1.27	21.7
105	Matrix protein	SLMDINPWL	HLA-A*02:01	9	0.20889	94.87% (37/39)	Non-Toxin	1.53	0.43	-0.53	1.96	1.42	3.4
106	Matrix protein	SLMDINPWL	HLA-A*02:06	9	0.20889	94.87% (37/39)	Non-Toxin	1.53	0.43	-0.66	1.96	1.29	4.6
107	Matrix protein	SLMDINPWL	HLA-A*02:03	9	0.20889	94.87% (37/39)	Non-Toxin	1.53	0.43	-0.91	1.96	1.04	8.2
108	Matrix protein	SMIPKYKIY	HLA-B*15:01	9	-0.28568	97.44% (38/39)	Non-Toxin	1.53	1.36	-1.57	2.89	1.33	36.9
109	Matrix protein	YMIPRTMLEF	HLA-B*15:01	10	0.00408	97.44% (38/39)	Non-Toxin	1.4	1.18	-0.79	2.57	1.79	6.1
110	Matrix protein	YMIPRTMLEF	HLA-A*23:01	10	0.00408	97.44% (38/39)	Non-Toxin	1.4	1.18	-1.39	2.57	1.18	24.7
111	nucleocapsid	AAFTLISMY	HLA-A*30:02	9	-0.05068	100.00% (13/13)	Non-Toxin	1.32	1.39	-1.7	2.72	1.01	50.5
112	nucleocapsid	AAFTLISMY	HLA-B*15:01	9	-0.05068	100.00% (13/13)	Non-Toxin	1.32	1.39	-2.02	2.72	0.69	105.9
113	nucleocapsid	AAFTLISMY	HLA-B*35:01	9	-0.05068	100.00% (13/13)	Non-Toxin	1.32	1.39	-2.15	2.72	0.56	141.5
114	nucleocapsid	AESMKVGAAF	HLA-B*44:02	10	-0.31242	100.00% (13/13)	Non-Toxin	1.23	1.17	-1.06	2.4	1.34	11.4
115	nucleocapsid	AESMKVGAAF	HLA-B*44:03	10	-0.31242	100.00% (13/13)	Non-Toxin	1.23	1.17	-1.18	2.4	1.22	15.1
116	nucleocapsid	AESMKVGAAF	HLA-B*15:01	10	-0.31242	100.00% (13/13)	Non-Toxin	1.23	1.17	-1.41	2.4	0.99	25.6
117	nucleocapsid	ATNSPELRW	HLA-B*58:01	9	-0.06424	100.00% (13/13)	Non-Toxin	1.42	0.33	-1.21	1.76	0.55	16.2
118	nucleocapsid	DIFEEAASF	HLA-B*35:01	9	0.20944	100.00% (13/13)	Non-Toxin	1.29	1.09	-1.87	2.38	0.52	73.7
119	nucleocapsid	DIFEEAASFR	HLA-A*68:01	10	0.23996	100.00% (13/13)	Non-Toxin	0.98	0.63	-1.07	1.62	0.55	11.8
120	nucleocapsid	EEAASFRSY	HLA-B*44:03	9	-0.05181	100.00% (13/13)	Non-Toxin	1.17	1.08	-1.35	2.26	0.91	22.3
121	nucleocapsid	EEAASFRSY	HLA-B*44:02	9	-0.05181	100.00% (13/13)	Non-Toxin	1.17	1.08	-1.37	2.26	0.89	23.5
122	nucleocapsid	EIISDIGNY	HLA-A*26:01	9	0.04843	100.00% (13/13)	Non-Toxin	1.19	1.29	-0.6	2.47	1.87	4
123	nucleocapsid	ESMKVGAAF	HLA-B*35:01	9	-0.14602	100.00% (13/13)	Non-Toxin	1.23	1.12	-1.55	2.35	0.8	35.4
124	nucleocapsid	ESMKVGAAF	HLA-A*26:01	9	-0.14602	100.00% (13/13)	Non-Toxin	1.23	1.12	-1.7	2.35	0.65	50.2
125	nucleocapsid	FAPGGYPLLW	HLA-B*53:01	10	0.03362	100.00% (13/13)	Non-Toxin	1.48	0.4	-1.21	1.89	0.68	16.1
126	nucleocapsid	FAPGGYPLLW	HLA-B*58:01	10	0.03362	100.00% (13/13)	Non-Toxin	1.48	0.4	-1.27	1.89	0.61	18.8
127	nucleocapsid	FMVEILIEV	HLA-A*02:06	9	0.40413	100.00% (13/13)	Non-Toxin	0.99	0.22	-0.32	1.22	0.9	2.1
128	nucleocapsid	FMVEILIEV	HLA-A*02:01	9	0.40413	100.00% (13/13)	Non-Toxin	0.99	0.22	-0.34	1.22	0.88	2.2
129	nucleocapsid	FMVEILIEV	HLA-A*02:03	9	0.40413	100.00% (13/13)	Non-Toxin	0.99	0.22	-0.43	1.22	0.79	2.7
130	nucleocapsid	FTLISMYSER	HLA-A*68:01	10	-0.28638	100.00% (13/13)	Non-Toxin	0.84	0.67	-0.88	1.51	0.63	7.5
131	nucleocapsid	GLSSDQVAEL	HLA-A*02:03	10	-0.18099	15.38% (2/13)	Non-Toxin	1.68	0.37	-1.46	2.05	0.58	29.1
132	nucleocapsid	GMAGFFATI	HLA-A*02:03	9	0.3267	100.00% (13/13)	Non-Toxin	1.35	0.22	-0.63	1.57	0.93	4.3
133	nucleocapsid	GPRAPYMVL	HLA-B*07:02	9	-0.08219	100.00% (13/13)	Non-Toxin	1.43	0.23	-0.76	1.66	0.9	5.8
134	nucleocapsid	GYLEPMYFRL	HLA-A*23:01	10	0.04091	100.00% (13/13)	Non-Toxin	1.46	0.52	-1.44	1.98	0.54	27.6
135	nucleocapsid	GYPLLWSF	HLA-A*23:01	8	0.04333	100.00% (13/13)	Non-Toxin	1.58	1.12	-1.84	2.7	0.85	69.9
136	nucleocapsid	GYPLLWSF	HLA-A*24:02	8	0.04333	100.00% (13/13)	Non-Toxin	1.58	1.12	-2.08	2.7	0.62	120.1
137	nucleocapsid	IQTKFAPGGY	HLA-A*30:02	10	-0.01434	100.00% (13/13)	Non-Toxin	1.19	1.32	-1.97	2.51	0.54	92.8
138	nucleocapsid	KFMVEILIEV	HLA-A*02:03	10	0.37202	100.00% (13/13)	Non-Toxin	0.99	0.34	-0.8	1.33	0.53	6.3
139	nucleocapsid	KFMVEILIEV	HLA-A*02:01	10	0.37202	100.00% (13/13)	Non-Toxin	0.99	0.34	-0.81	1.33	0.52	6.5
140	nucleocapsid	KTPFVDSRAY	HLA-A*30:02	10	0.08681	100.00% (13/13)	Non-Toxin	1.33	1.29	-1.7	2.62	0.92	50.2
141	nucleocapsid	LLSQSLSVRKF	HLA-B*15:01	11	-0.54672	100.00% (13/13)	Non-Toxin	1.46	1.17	-2.03	2.63	0.6	107.7

#	Protein	Peptide	Allele	Length	Immunogenicity	Conservancy	Toxicity	Proteasome Score	TAP Score	MHC Score	Processing Score	Total Score	MHC IC50[nM]
142	nucleocapsid	LLWSFAMGV	HLA-A*02:01	9	-0.07214	100.00% (13/13)	Non-Toxin	1.15	0.16	-0.63	1.31	0.67	4.3
143	nucleocapsid	LTNSLLNLR	HLA-A*68:01	9	-0.20175	100.00% (13/13)	Non-Toxin	0.98	0.65	-1.1	1.63	0.53	12.7
144	nucleocapsid	MAGFFATIR	HLA-A*68:01	9	0.39015	100.00% (13/13)	Non-Toxin	1.08	0.6	-0.99	1.69	0.7	9.7
145	nucleocapsid	MAGFFATIRF	HLA-B*58:01	10	0.46	100.00% (13/13)	Non-Toxin	1.45	1.06	-1.67	2.51	0.84	47.1
146	nucleocapsid	MAGFFATIRF	HLA-B*15:01	10	0.46	100.00% (13/13)	Non-Toxin	1.45	1.06	-1.95	2.51	0.56	89
147	nucleocapsid	MAGFFATIRF	HLA-B*35:01	10	0.46	100.00% (13/13)	Non-Toxin	1.45	1.06	-2	2.51	0.51	100.8
148	nucleocapsid	MVNGIPVMER	HLA-A*68:01	10	0.09996	100.00% (13/13)	Non-Toxin	1.09	0.71	-0.84	1.8	0.96	6.9
149	nucleocapsid	MVNGIPVMERR	HLA-A*68:01	11	0.14044	100.00% (13/13)	Non-Toxin	1.28	0.71	-1.36	1.98	0.62	23.1
150	nucleocapsid	NTIKSLMLLY	HLA-A*26:01	10	-0.52684	100.00% (13/13)	Non-Toxin	1.34	1.33	-1.74	2.67	0.94	54.4
151	nucleocapsid	NTIKSLMLLY	HLA-A*68:01	10	-0.52684	100.00% (13/13)	Non-Toxin	1.34	1.33	-2.05	2.67	0.62	112.5
152	nucleocapsid	NTIKSLMLLY	HLA-A*11:01	10	-0.52684	100.00% (13/13)	Non-Toxin	1.34	1.33	-2.1	2.67	0.58	124.5
153	nucleocapsid	QQKRVNPFF	HLA-B*15:01	9	0.07523	100.00% (13/13)	Non-Toxin	1.54	1.16	-1.95	2.69	0.74	90.1
154	nucleocapsid	QTKFAPGGY	HLA-A*30:02	9	0.12386	100.00% (13/13)	Non-Toxin	1.19	1.27	-1.89	2.46	0.57	77.2
155	nucleocapsid	RGYLEPMYFR	HLA-A*31:01	10	-0.02568	100.00% (13/13)	Non-Toxin	0.94	0.78	-1.02	1.72	0.7	10.4
156	nucleocapsid	RPGALIRSL	HLA-B*07:02	9	0.11187	100.00% (13/13)	Non-Toxin	1.37	0.25	-0.88	1.61	0.74	7.5
157	nucleocapsid	RPGALIRSLL	HLA-B*07:02	10	0.07179	100.00% (13/13)	Non-Toxin	1.57	0.25	-0.88	1.81	0.94	7.5
158	nucleocapsid	RSMGALNINR	HLA-A*31:01	10	0.10685	100.00% (13/13)	Non-Toxin	1.16	0.74	-1.07	1.89	0.82	11.7
159	nucleocapsid	RSYQSKLGR	HLA-A*31:01	9	-0.47142	100.00% (13/13)	Non-Toxin	0.88	0.8	-1.1	1.68	0.58	12.7
160	nucleocapsid	RYPALALNEF	HLA-A*24:02	10	0.10567	100.00% (13/13)	Non-Toxin	1.25	1.32	-1.09	2.57	1.47	12.4
161	nucleocapsid	RYPALALNEF	HLA-A*23:01	10	0.10567	100.00% (13/13)	Non-Toxin	1.25	1.32	-1.21	2.57	1.35	16.4
162	nucleocapsid	RYPALALNEF	HLA-B*07:02	10	0.10567	100.00% (13/13)	Non-Toxin	1.25	1.32	-1.89	2.57	0.68	77.6
163	nucleocapsid	RYPALALNEF	HLA-B*35:01	10	0.10567	100.00% (13/13)	Non-Toxin	1.25	1.32	-2.02	2.57	0.54	105.5
164	nucleocapsid	SQSLSVRKF	HLA-B*15:01	9	-0.26942	100.00% (13/13)	Non-Toxin	1.46	1.17	-1.84	2.63	0.79	69.4
165	nucleocapsid	TIKSLMLLY	HLA-A*30:02	9	-0.42841	100.00% (13/13)	Non-Toxin	1.34	1.38	-1.81	2.72	0.91	64.8
166	nucleocapsid	TIKSLMLLYR	HLA-A*31:01	10	-0.44023	100.00% (13/13)	Non-Toxin	1.17	0.75	-1.34	1.92	0.58	22
167	nucleocapsid	TLFALDVIR	HLA-A*68:01	9	0.20005	100.00% (13/13)	Non-Toxin	1.17	0.71	-1.18	1.88	0.7	15.2
168	nucleocapsid	TPFVDSRAY	HLA-B*35:01	9	0.01195	100.00% (13/13)	Non-Toxin	1.33	1.22	-0.79	2.56	1.77	6.1
169	nucleocapsid	VEIISDIGNY	HLA-A*26:01	10	0.18772	100.00% (13/13)	Non-Toxin	1.19	1.33	-1.82	2.52	0.7	66.8
170	nucleocapsid	VQQKRVNPF	HLA-B*15:01	9	-0.17728	100.00% (13/13)	Non-Toxin	1.01	1.12	-1.64	2.13	0.49	43.4
171	nucleocapsid	VQQKRVNPFF	HLA-B*15:01	10	-0.11105	100.00% (13/13)	Non-Toxin	1.54	1.12	-1.98	2.66	0.68	95.6
172	nucleocapsid	YLEPMYFRL	HLA-A*02:01	9	-0.0241	100.00% (13/13)	Non-Toxin	1.46	0.34	-0.85	1.8	0.96	7
173	nucleocapsid	YLEPMYFRL	HLA-A*02:06	9	-0.0241	100.00% (13/13)	Non-Toxin	1.46	0.34	-1.13	1.8	0.67	13.4
174	nucleocapsid	YPALALNEF	HLA-B*35:01	9	0.08224	100.00% (13/13)	Non-Toxin	1.25	1.02	-0.61	2.27	1.66	4.1
175	nucleocapsid	YPALALNEF	HLA-B*53:01	9	0.08224	100.00% (13/13)	Non-Toxin	1.25	1.02	-1.33	2.27	0.94	21.6
176	nucleocapsid	YPLLWSFAM	HLA-B*35:01	9	0.16687	100.00% (13/13)	Non-Toxin	1.01	-0.01	-0.32	1	0.68	2.1
177	nucleocapsid	YVQQKRVNPF	HLA-B*15:01	10	-0.28684	100.00% (13/13)	Non-Toxin	1.01	1.18	-1.59	2.19	0.6	38.6
178	phosphoprotein	CLVSDAKMLSY	HLA-A*01:01	11	-0.57469	59.26% (16/27)	Non-Toxin	1.48	1.31	-1.82	2.8	0.98	65.8
179	phosphoprotein	DPVVTDVVY	HLA-B*35:01	9	0.17258	100.00% (27/27)	Non-Toxin	1.74	1.05	-1.4	2.8	1.4	25.1
180	phosphoprotein	ELRSELIGY	HLA-A*26:01	9	0.06951	96.30% (26/27)	Non-Toxin	1.2	1.27	-1.92	2.46	0.55	82.8
181	phosphoprotein	FPHDTDRLNY	HLA-B*35:01	10	0.1278	100.00% (27/27)	Non-Toxin	1.41	1.06	-1.06	2.47	1.41	11.4
182	phosphoprotein	FPHDTDRLNY	HLA-B*53:01	10	0.1278	100.00% (27/27)	Non-Toxin	1.41	1.06	-1.9	2.47	0.57	79.2
183	phosphoprotein	FSFDNVKNF	HLA-B*35:01	9	-0.0929	100.00% (27/27)	Non-Toxin	1.81	1.15	-2.14	2.96	0.82	137
184	phosphoprotein	FSFDNVKNF	HLA-B*15:01	9	-0.0929	100.00% (27/27)	Non-Toxin	1.81	1.15	-2.25	2.96	0.71	178
185	phosphoprotein	FSFDNVKNFR	HLA-A*68:01	10	-0.04584	100.00% (27/27)	Non-Toxin	0.97	0.69	-0.58	1.66	1.08	3.8
186	phosphoprotein	FSFDNVKNFR	HLA-A*31:01	10	-0.04584	100.00% (27/27)	Non-Toxin	0.97	0.69	-1.15	1.66	0.51	14.1
187	phosphoprotein	GLNPTAVPF	HLA-B*15:01	9	0.08973	62.96% (17/27)	Non-Toxin	1.12	0.99	-1.4	2.12	0.72	24.9
188	phosphoprotein	IMPSDDFSNTF	HLA-B*35:01	11	-0.15138	96.30% (26/27)	Non-Toxin	1.53	1.13	-2.14	2.66	0.52	139.2
189	phosphoprotein	KLINLDMRL	HLA-A*02:01	9	-0.07119	100.00% (27/27)	Non-Toxin	1.61	0.51	-1.21	2.11	0.91	16.1
190	phosphoprotein	KLINLDMRL	HLA-A*02:03	9	-0.07119	100.00% (27/27)	Non-Toxin	1.61	0.51	-1.54	2.11	0.57	34.9

#	Protein	Peptide	Allele	Length	Immunogenicity	Conservancy	Toxicity	Proteasome Score	TAP Score	MHC Score	Processing Score	Total Score	MHC IC50[nM]
191	phosphoprotein	KMLSYAPEI	HLA-A*02:01	9	-0.0877	59.26% (16/27)	Non-Toxin	1.24	0.4	-0.68	1.65	0.97	4.8
192	phosphoprotein	KMLSYAPEI	HLA-A*02:06	9	-0.0877	59.26% (16/27)	Non-Toxin	1.24	0.4	-0.82	1.65	0.83	6.6
193	phosphoprotein	KMLSYAPEI	HLA-A*32:01	9	-0.0877	59.26% (16/27)	Non-Toxin	1.24	0.4	-1.13	1.65	0.52	13.4
194	phosphoprotein	KSRGIPIKK	HLA-A*30:01	9	0.15638	100.00% (27/27)	Non-Toxin	1.09	0.22	-0.73	1.31	0.58	5.4
195	phosphoprotein	LDPVVTDVVY	HLA-B*35:01	10	0.19578	100.00% (27/27)	Non-Toxin	1.74	1.11	-1.34	2.85	1.51	22.1
196	phosphoprotein	LFSFDNVKNF	HLA-A*23:01	10	-0.06752	100.00% (27/27)	Non-Toxin	1.81	1.2	-2.34	3.01	0.67	216.4
197	phosphoprotein	LVSDAKMLSY	HLA-A*01:01	10	-0.4746	59.26% (16/27)	Non-Toxin	1.48	1.33	-1.26	2.81	1.55	18
198	phosphoprotein	LVSDAKMLSY	HLA-B*15:01	10	-0.4746	59.26% (16/27)	Non-Toxin	1.48	1.33	-1.94	2.81	0.87	87.9
199	phosphoprotein	MPSDDFSNTF	HLA-B*35:01	10	-0.03429	96.30% (26/27)	Non-Toxin	1.53	0.99	-0.88	2.52	1.64	7.6
200	phosphoprotein	MPSDDFSNTF	HLA-B*53:01	10	-0.03429	96.30% (26/27)	Non-Toxin	1.53	0.99	-1.15	2.52	1.37	14.2
201	phosphoprotein	MPSDDFSNTFF	HLA-B*53:01	11	0.03819	96.30% (26/27)	Non-Toxin	1.28	0.99	-1.76	2.27	0.52	56.9
202	phosphoprotein	NPTAVPFTLR	HLA-A*68:01	10	0.21785	51.85% (14/27)	Non-Toxin	1.01	0.53	-1.03	1.54	0.51	10.7
203	phosphoprotein	NTFFPHDTDR	HLA-A*68:01	10	0.2431	100.00% (27/27)	Non-Toxin	0.69	0.72	-0.81	1.42	0.6	6.5
204	phosphoprotein	REDLILPEL	HLA-B*40:01	9	0.16434	62.96% (17/27)	Non-Toxin	1.61	0.41	-1	2.02	1.02	9.9
205	phosphoprotein	SPVIAEHYY	HLA-B*35:01	9	0.30481	96.30% (26/27)	Non-Toxin	1.36	1.13	-1.31	2.5	1.18	20.6
206	phosphoprotein	SVLMGVINSI	HLA-A*02:03	10	-0.08394	100.00% (27/27)	Non-Toxin	1.25	0.25	-0.86	1.5	0.64	7.2
207	phosphoprotein	VLAKTNTAL	HLA-A*02:03	9	-0.11697	100.00% (27/27)	Non-Toxin	1.54	0.52	-1.55	2.05	0.5	35.8
208	phosphoprotein	VLMGVINSI	HLA-A*02:03	9	0.04046	100.00% (27/27)	Non-Toxin	1.25	0.32	-0.61	1.57	0.96	4.1
209	phosphoprotein	VLMGVINSI	HLA-A*02:01	9	0.04046	100.00% (27/27)	Non-Toxin	1.25	0.32	-0.96	1.57	0.61	9.2
210	phosphoprotein	VLMGVINSI	HLA-A*02:06	9	0.04046	100.00% (27/27)	Non-Toxin	1.25	0.32	-1.03	1.57	0.54	10.7
211	phosphoprotein	VSDAKMLSY	HLA-A*01:01	9	-0.43475	59.26% (16/27)	Non-Toxin	1.48	1.26	-0.94	2.74	1.8	8.7
212	polymerase	AEFFSFFRTF	HLA-B*44:03	10	0.28526	100.00% (10/10)	Non-Toxin	1.49	1.17	-1.05	2.66	1.61	11.3
213	polymerase	AEFFSFFRTF	HLA-B*44:02	10	0.28526	100.00% (10/10)	Non-Toxin	1.49	1.17	-1.22	2.66	1.44	16.5
214	polymerase	AEFFSFFRTF	HLA-A*23:01	10	0.28526	100.00% (10/10)	Non-Toxin	1.49	1.17	-1.44	2.66	1.22	27.7
215	polymerase	AEFFSFFRTF	HLA-B*40:01	10	0.28526	100.00% (10/10)	Non-Toxin	1.49	1.17	-1.77	2.66	0.89	59.1
216	polymerase	AEFFSFFRTF	HLA-A*24:02	10	0.28526	100.00% (10/10)	Non-Toxin	1.49	1.17	-2.06	2.66	0.6	115.6
217	polymerase	AIRIATVYTW	HLA-B*58:01	10	0.28504	100.00% (10/10)	Non-Toxin	1.73	0.55	-1.77	2.28	0.51	59.2
218	polymerase	ALIASGVGKY	HLA-B*15:01	10	-0.10407	100.00% (10/10)	Non-Toxin	1.13	1.3	-1.6	2.42	0.83	39.7
219	polymerase	ALIPAPIGGF	HLA-B*15:01	10	0.23302	100.00% (10/10)	Non-Toxin	1.32	1.27	-1.69	2.59	0.9	48.9
220	polymerase	ALKSMSRCCF	HLA-B*15:01	10	-0.59685	90.00% (9/10)	Non-Toxin	1.34	1.15	-1.72	2.49	0.77	52.9
221	polymerase	ALWGLIDPL	HLA-A*02:01	9	0.23272	100.00% (10/10)	Non-Toxin	1.07	0.62	-0.92	1.7	0.78	8.3
222	polymerase	ALWGLIDPL	HLA-A*02:06	9	0.23272	100.00% (10/10)	Non-Toxin	1.07	0.62	-1.15	1.7	0.55	14
223	polymerase	APIGGFNYL	HLA-B*07:02	9	0.21288	100.00% (10/10)	Non-Toxin	1.51	0.31	-1.28	1.83	0.54	19.2
224	polymerase	APIMKAHAIF	HLA-B*07:02	10	-0.16417	100.00% (10/10)	Non-Toxin	1.24	1.04	-1.55	2.27	0.73	35.1
225	polymerase	APIMKAHAIF	HLA-B*15:01	10	-0.16417	100.00% (10/10)	Non-Toxin	1.24	1.04	-1.72	2.27	0.55	52.4
226	polymerase	ATIPFLFLSAY	HLA-A*11:01	11	0.12204	100.00% (10/10)	Non-Toxin	1.39	1.36	-2.05	2.75	0.7	112.7
227	polymerase	ATIPFLFLSAY	HLA-A*30:02	11	0.12204	100.00% (10/10)	Non-Toxin	1.39	1.36	-2.11	2.75	0.64	128.7
228	polymerase	AYPECNNILF	HLA-A*24:02	10	0.1381	90.00% (9/10)	Non-Toxin	1.47	1.27	-1.97	2.75	0.78	92.6
229	polymerase	AYPECNNILF	HLA-A*23:01	10	0.1381	90.00% (9/10)	Non-Toxin	1.47	1.27	-2.02	2.75	0.72	105.5
230	polymerase	CSVDLARALR	HLA-A*68:01	10	0.13828	100.00% (10/10)	Non-Toxin	1.06	0.68	-1.24	1.74	0.5	17.4
231	polymerase	DSYEYIINR	HLA-A*68:01	9	0.32977	100.00% (10/10)	Non-Toxin	1.14	0.63	-1.02	1.76	0.74	10.5
232	polymerase	EAWYLASQR	HLA-A*68:01	9	-0.11309	100.00% (10/10)	Non-Toxin	0.98	0.63	-1.05	1.61	0.56	11.3
233	polymerase	EFFSFFRTF	HLA-A*23:01	9	0.16209	100.00% (10/10)	Non-Toxin	1.49	1.2	-1.34	2.69	1.35	22
234	polymerase	EFFSFFRTF	HLA-A*24:02	9	0.16209	100.00% (10/10)	Non-Toxin	1.49	1.2	-1.96	2.69	0.73	90.6
235	polymerase	EIINIHECR	HLA-A*68:01	9	0.24974	100.00% (10/10)	Non-Toxin	1.31	0.58	-0.77	1.89	1.12	5.9
236	polymerase	EIINIHECR	HLA-A*33:01	9	0.24974	100.00% (10/10)	Non-Toxin	1.31	0.58	-1.2	1.89	0.68	16
237	polymerase	EILKSEISY	HLA-B*35:01	9	-0.27179	100.00% (10/10)	Non-Toxin	1.47	1.18	-2	2.65	0.65	100.9
238	polymerase	EIYGLPGFF	HLA-A*26:01	9	0.10866	100.00% (10/10)	Non-Toxin	1.3	1.09	-1.88	2.39	0.51	75.8
239	polymerase	ESMAIFAER	HLA-A*68:01	9	0.31369	100.00% (10/10)	Non-Toxin	1	0.58	-0.48	1.58	1.1	3

#	Protein	Peptide	Allele	Length	Immunogenicity	Conservancy	Toxicity	Proteasome Score	TAP Score	MHC Score	Processing Score	Total Score	MHC IC50[nM]
240	polymerase	ESMAIFAER	HLA-A*33:01	9	0.31369	100.00% (10/10)	Non-Toxin	1	0.58	-0.72	1.58	0.85	5.3
241	polymerase	ESMAIFAERL	HLA-A*68:02	10	0.37754	100.00% (10/10)	Non-Toxin	1.47	0.35	-1.17	1.82	0.65	14.7
242	polymerase	ETDDYNGIY	HLA-A*01:01	9	0.12619	100.00% (10/10)	Non-Toxin	1.41	1.12	-0.91	2.53	1.62	8.1
243	polymerase	ETIISTHLF	HLA-A*26:01	9	0.07338	100.00% (10/10)	Non-Toxin	1.09	1.04	-1.29	2.12	0.83	19.6
244	polymerase	ETTWIGNLDSY	HLA-A*26:01	11	0.30341	100.00% (10/10)	Non-Toxin	1.29	1.09	-1.78	2.38	0.61	59.8
245	polymerase	EVDNNHLIY	HLA-A*01:01	9	0.09324	100.00% (10/10)	Non-Toxin	1.3	1.17	-1.25	2.47	1.22	17.9
246	polymerase	EVDNNHLIY	HLA-B*35:01	9	0.09324	100.00% (10/10)	Non-Toxin	1.3	1.17	-1.9	2.47	0.58	78.6
247	polymerase	EVIDTTTML	HLA-A*68:02	9	0.07002	70.00% (7/10)	Non-Toxin	1.88	0.42	-1.18	2.31	1.12	15.2
248	polymerase	EVIDTTTML	HLA-A*26:01	9	0.07002	70.00% (7/10)	Non-Toxin	1.88	0.42	-1.4	2.31	0.9	25.3
249	polymerase	EVIDTTTMLR	HLA-A*68:01	10	0.02298	70.00% (7/10)	Non-Toxin	0.9	0.65	-0.8	1.55	0.75	6.3
250	polymerase	EYIINRTAGR	HLA-A*33:01	10	0.31058	100.00% (10/10)	Non-Toxin	1.16	0.68	-1.25	1.84	0.59	17.8
251	polymerase	FAERLDEIY	HLA-B*35:01	9	0.25692	100.00% (10/10)	Non-Toxin	1.41	1.19	-1.5	2.6	1.1	31.9
252	polymerase	FIDDLLSIL	HLA-A*02:06	9	-0.05358	100.00% (10/10)	Non-Toxin	1.7	0.37	-1.23	2.07	0.85	16.8
253	polymerase	FIDDLLSIL	HLA-A*02:01	9	-0.05358	100.00% (10/10)	Non-Toxin	1.7	0.37	-1.32	2.07	0.75	21.1
254	polymerase	FIYSKKIHY	HLA-B*15:01	9	-0.44945	90.00% (9/10)	Non-Toxin	1.38	1.39	-1.83	2.77	0.94	67.5
255	polymerase	FLDWASDPY	HLA-B*35:01	9	0.1247	100.00% (10/10)	Non-Toxin	1.08	1.18	-1.15	2.26	1.11	14.2
256	polymerase	FLDWASDPY	HLA-A*01:01	9	0.1247	100.00% (10/10)	Non-Toxin	1.08	1.18	-1.3	2.26	0.96	20
257	polymerase	FLIVDPELFAL	HLA-A*02:01	11	0.30426	100.00% (10/10)	Non-Toxin	1.59	0.34	-1.23	1.93	0.71	16.8
258	polymerase	FLIVDPELFAL	HLA-A*02:06	11	0.30426	100.00% (10/10)	Non-Toxin	1.59	0.34	-1.28	1.93	0.65	18.9
259	polymerase	FLMDRRVIL	HLA-B*08:01	9	0.17704	100.00% (10/10)	Non-Toxin	1.55	0.44	-0.9	1.99	1.09	7.9
260	polymerase	FLMDRRVIL	HLA-A*02:03	9	0.17704	100.00% (10/10)	Non-Toxin	1.55	0.44	-1	1.99	0.99	10
261	polymerase	FLMDRRVIL	HLA-A*02:01	9	0.17704	100.00% (10/10)	Non-Toxin	1.55	0.44	-1.12	1.99	0.86	13.3
262	polymerase	FNYLNLSRIF	HLA-A*23:01	10	-0.06375	100.00% (10/10)	Non-Toxin	1.29	1.06	-1.61	2.35	0.74	40.9
263	polymerase	FPISRLFNMY	HLA-B*35:01	10	-0.08153	100.00% (10/10)	Non-Toxin	1.46	1.11	-0.98	2.57	1.59	9.6
264	polymerase	FPISRLFNMY	HLA-B*53:01	10	-0.08153	100.00% (10/10)	Non-Toxin	1.46	1.11	-1.6	2.57	0.97	39.9
265	polymerase	FPLWSTEEL	HLA-B*35:01	9	0.23773	100.00% (10/10)	Non-Toxin	1.65	0.19	-0.91	1.84	0.93	8.1
266	polymerase	FPLWSTEEL	HLA-B*53:01	9	0.23773	100.00% (10/10)	Non-Toxin	1.65	0.19	-1.29	1.84	0.56	19.3
267	polymerase	FPVMGNRIY	HLA-B*35:01	9	-0.01495	100.00% (10/10)	Non-Toxin	1.57	1.15	-0.51	2.72	2.22	3.2
268	polymerase	FPVMGNRIY	HLA-B*53:01	9	-0.01495	100.00% (10/10)	Non-Toxin	1.57	1.15	-1.75	2.72	0.97	56.4
269	polymerase	FVIFYASLTY	HLA-B*15:01	10	0.05309	100.00% (10/10)	Non-Toxin	1.22	1.26	-1.28	2.48	1.2	19
270	polymerase	FVIFYASLTY	HLA-B*35:01	10	0.05309	100.00% (10/10)	Non-Toxin	1.22	1.26	-1.66	2.48	0.83	45.2
271	polymerase	FVNDENFDPY	HLA-B*35:01	10	0.23386	100.00% (10/10)	Non-Toxin	1.05	1.27	-1.3	2.31	1.01	20.1
272	polymerase	FVNDENFDPY	HLA-A*01:01	10	0.23386	100.00% (10/10)	Non-Toxin	1.05	1.27	-1.78	2.31	0.53	60.4
273	polymerase	FYASLTYLR	HLA-A*33:01	9	-0.13763	100.00% (10/10)	Non-Toxin	1.06	0.73	-0.95	1.79	0.84	8.9
274	polymerase	FYASLTYLRR	HLA-A*33:01	10	-0.10937	100.00% (10/10)	Non-Toxin	1.29	0.73	-1.32	2.02	0.7	21
275	polymerase	GIQFDCFMEL	HLA-A*02:06	10	0.0698	100.00% (10/10)	Non-Toxin	1.55	0.38	-1.32	1.93	0.61	20.7
276	polymerase	GLIDPLFPV	HLA-A*02:06	9	0.1366	100.00% (10/10)	Non-Toxin	0.88	0.07	-0.26	0.95	0.69	1.8
277	polymerase	GLIDPLFPV	HLA-A*02:01	9	0.1366	100.00% (10/10)	Non-Toxin	0.88	0.07	-0.28	0.95	0.67	1.9
278	polymerase	GLIDPLFPV	HLA-A*02:03	9	0.1366	100.00% (10/10)	Non-Toxin	0.88	0.07	-0.36	0.95	0.59	2.3
279	polymerase	GQSISIKWAF	HLA-B*15:01	10	0.05552	100.00% (10/10)	Non-Toxin	1.4	1.07	-1.78	2.47	0.7	59.9
280	polymerase	GSGSMMLLY	HLA-A*30:02	9	-0.50761	100.00% (10/10)	Non-Toxin	1.14	1.07	-1.6	2.21	0.61	39.9
281	polymerase	GTKFDTVSAF	HLA-B*15:01	10	0.0293	100.00% (10/10)	Non-Toxin	1.24	1.02	-1.75	2.25	0.51	55.6
282	polymerase	HLLAEFFSF	HLA-A*23:01	9	0.24561	100.00% (10/10)	Non-Toxin	1.14	1.08	-1.41	2.22	0.8	25.9
283	polymerase	HLLAEFFSF	HLA-A*32:01	9	0.24561	100.00% (10/10)	Non-Toxin	1.14	1.08	-1.47	2.22	0.74	29.7
284	polymerase	HLLAEFFSF	HLA-B*15:01	9	0.24561	100.00% (10/10)	Non-Toxin	1.14	1.08	-1.64	2.22	0.58	43.4
285	polymerase	HLLAEFFSFF	HLA-B*15:01	10	0.28625	100.00% (10/10)	Non-Toxin	1.37	1.08	-1.58	2.45	0.87	37.8
286	polymerase	HMLADKVLEY	HLA-B*15:01	10	-0.06463	100.00% (10/10)	Non-Toxin	1.56	1.22	-1.74	2.78	1.04	54.5
287	polymerase	HMLADKVLEY	HLA-A*30:02	10	-0.06463	100.00% (10/10)	Non-Toxin	1.56	1.22	-2.26	2.78	0.52	183.1
288	polymerase	HTEFNPHNHY	HLA-A*30:02	10	0.17709	100.00% (10/10)	Non-Toxin	1.36	1.14	-1.7	2.5	0.8	50.3

#	Protein	Peptide	Allele	Length	Immunogenicity	Conservancy	Toxicity	Proteasome Score	TAP Score	MHC Score	Processing Score	Total Score	MHC IC50[nM]
289	polymerase	IATVYTWAY	HLA-B*35:01	9	0.29688	100.00% (10/10)	Non-Toxin	1.56	1.34	-0.93	2.9	1.97	8.6
290	polymerase	IATVYTWAY	HLA-B*58:01	9	0.29688	100.00% (10/10)	Non-Toxin	1.56	1.34	-2.04	2.9	0.87	109
291	polymerase	IATVYTWAY	HLA-B*53:01	9	0.29688	100.00% (10/10)	Non-Toxin	1.56	1.34	-2.21	2.9	0.69	162.7
292	polymerase	IATVYTWAY	HLA-A*30:02	9	0.29688	100.00% (10/10)	Non-Toxin	1.56	1.34	-2.23	2.9	0.68	168.5
293	polymerase	IFYASLTYLR	HLA-A*31:01	10	-0.10679	100.00% (10/10)	Non-Toxin	1.06	0.83	-0.97	1.89	0.92	9.4
294	polymerase	IFYASLTYLR	HLA-A*33:01	10	-0.10679	100.00% (10/10)	Non-Toxin	1.06	0.83	-1.18	1.89	0.71	15.3
295	polymerase	IFYASLTYLRR	HLA-A*31:01	11	-0.07853	100.00% (10/10)	Non-Toxin	1.29	0.83	-1.59	2.12	0.53	38.9
296	polymerase	IHLLAEFFSF	HLA-A*23:01	10	0.23318	100.00% (10/10)	Non-Toxin	1.14	1.18	-1.79	2.32	0.52	62
297	polymerase	IIMLNEAMNY	HLA-B*15:01	10	-0.09211	100.00% (10/10)	Non-Toxin	1.48	1.37	-2.15	2.85	0.7	141.9
298	polymerase	IIMLNEAMNY	HLA-A*30:02	10	-0.09211	100.00% (10/10)	Non-Toxin	1.48	1.37	-2.32	2.85	0.53	210.3
299	polymerase	IISTHLFIY	HLA-A*30:02	9	0.18298	90.00% (9/10)	Non-Toxin	1.26	1.34	-1.85	2.6	0.74	71
300	polymerase	ILNIDNIHLL	HLA-A*02:03	10	0.29322	100.00% (10/10)	Non-Toxin	1.57	0.42	-1.36	1.99	0.63	22.7
301	polymerase	ILNIDNIHLL	HLA-A*02:01	10	0.29322	100.00% (10/10)	Non-Toxin	1.57	0.42	-1.4	1.99	0.59	25.1
302	polymerase	ILNPNLICIF	HLA-B*15:01	10	0.12718	100.00% (10/10)	Non-Toxin	1.35	1.08	-1.62	2.43	0.81	41.6
303	polymerase	ILVEHSHLI	HLA-A*02:03	9	0.01074	100.00% (10/10)	Non-Toxin	1.21	0.21	-0.9	1.42	0.52	8
304	polymerase	IMKKSFKAY	HLA-B*15:01	9	-0.49704	100.00% (10/10)	Non-Toxin	1.59	1.39	-1.07	2.98	1.92	11.7
305	polymerase	IMKKSFKAY	HLA-A*30:02	9	-0.49704	100.00% (10/10)	Non-Toxin	1.59	1.39	-1.83	2.98	1.15	67.8
306	polymerase	IMLNEAMNY	HLA-A*30:02	9	-0.02776	100.00% (10/10)	Non-Toxin	1.48	1.34	-1.81	2.83	1.01	64.9
307	polymerase	IMLNEAMNY	HLA-B*15:01	9	-0.02776	100.00% (10/10)	Non-Toxin	1.48	1.34	-1.81	2.83	1.01	65.3
308	polymerase	IMLNEAMNY	HLA-A*03:01	9	-0.02776	100.00% (10/10)	Non-Toxin	1.48	1.34	-2.15	2.83	0.68	141.1
309	polymerase	IMLNEAMNYF	HLA-B*15:01	10	-0.04743	100.00% (10/10)	Non-Toxin	1.38	1.17	-1.38	2.56	1.18	23.9
310	polymerase	IPAPIGGFNY	HLA-B*35:01	10	0.29106	100.00% (10/10)	Non-Toxin	1.26	1.18	-1.49	2.43	0.94	30.9
311	polymerase	IPFLFLSAY	HLA-B*35:01	9	0.01364	100.00% (10/10)	Non-Toxin	1.39	1.2	-0.67	2.59	1.92	4.7
312	polymerase	IPFLFLSAY	HLA-B*53:01	9	0.01364	100.00% (10/10)	Non-Toxin	1.39	1.2	-2.07	2.59	0.52	118.6
313	polymerase	IQFDCFMEL	HLA-A*02:06	9	0.02832	100.00% (10/10)	Non-Toxin	1.55	0.49	-0.81	2.05	1.23	6.5
314	polymerase	IQFDCFMEL	HLA-A*02:01	9	0.02832	100.00% (10/10)	Non-Toxin	1.55	0.49	-1.27	2.05	0.77	18.8
315	polymerase	IQQLLISTEF	HLA-B*15:01	10	0.00557	100.00% (10/10)	Non-Toxin	1.56	1.15	-1.73	2.71	0.98	53.3
316	polymerase	IRIATVYTWAY	HLA-A*30:02	11	0.40331	100.00% (10/10)	Non-Toxin	1.56	1.47	-2.43	3.03	0.6	267.3
317	polymerase	ISRLFNMYR	HLA-A*31:01	9	-0.03681	100.00% (10/10)	Non-Toxin	0.98	0.63	-0.69	1.61	0.92	4.9
318	polymerase	IVDPELFALY	HLA-A*01:01	10	0.21948	100.00% (10/10)	Non-Toxin	1.03	1.28	-1.34	2.32	0.98	21.8
319	polymerase	IYGLPGFFNW	HLA-A*23:01	10	0.22726	100.00% (10/10)	Non-Toxin	1.52	0.36	-0.94	1.89	0.94	8.8
320	polymerase	IYGLPGFFNW	HLA-A*24:02	10	0.22726	100.00% (10/10)	Non-Toxin	1.52	0.36	-1.25	1.89	0.64	17.6
321	polymerase	IYLMNWCDF	HLA-A*23:01	9	-0.01063	100.00% (10/10)	Non-Toxin	0.96	1.25	-1.09	2.21	1.12	12.3
322	polymerase	IYLMNWCDF	HLA-A*24:02	9	-0.01063	100.00% (10/10)	Non-Toxin	0.96	1.25	-1.24	2.21	0.97	17.5
323	polymerase	KALSPIKDEW	HLA-B*58:01	10	-0.17705	100.00% (10/10)	Non-Toxin	1.25	0.4	-0.89	1.65	0.76	7.8
324	polymerase	KEICAKQAQLY	HLA-B*44:03	11	-0.36316	100.00% (10/10)	Non-Toxin	1.18	1.33	-2	2.51	0.51	100.5
325	polymerase	KIWWKIIGY	HLA-A*32:01	9	0.34219	100.00% (10/10)	Non-Toxin	1.24	1.47	-2.12	2.7	0.58	131.2
326	polymerase	KKYYQIDQPF	HLA-A*23:01	10	-0.07148	100.00% (10/10)	Non-Toxin	0.86	1.27	-1.27	2.12	0.85	18.7
327	polymerase	KLFPSEYSI	HLA-A*32:01	9	-0.13979	100.00% (10/10)	Non-Toxin	1.34	0.36	-0.9	1.7	0.79	8
328	polymerase	KLFPSEYSI	HLA-A*02:01	9	-0.13979	100.00% (10/10)	Non-Toxin	1.34	0.36	-1.16	1.7	0.54	14.5
329	polymerase	KLKGLVVPL	HLA-A*02:03	9	0.02052	100.00% (10/10)	Non-Toxin	1.33	0.46	-1.2	1.79	0.59	15.9
330	polymerase	KLKGLVVPLF	HLA-B*15:01	10	0.01652	100.00% (10/10)	Non-Toxin	1.37	1.14	-1.65	2.51	0.87	44.6
331	polymerase	KLKGLVVPLF	HLA-A*32:01	10	0.01652	100.00% (10/10)	Non-Toxin	1.37	1.14	-2.01	2.51	0.5	103
332	polymerase	KQFHDDLKKY	HLA-A*30:02	10	-0.20469	100.00% (10/10)	Non-Toxin	1.58	1.42	-2.4	3	0.61	248.6
333	polymerase	KQFHDDLKKY	HLA-B*15:01	10	-0.20469	100.00% (10/10)	Non-Toxin	1.58	1.42	-2.42	3	0.59	260.2
334	polymerase	KSFLDYHTEF	HLA-B*58:01	10	0.16655	100.00% (10/10)	Non-Toxin	1.46	1.24	-1.51	2.69	1.19	32.2
335	polymerase	KSFLDYHTEF	HLA-B*57:01	10	0.16655	100.00% (10/10)	Non-Toxin	1.46	1.24	-1.71	2.69	0.99	50.9
336	polymerase	KSFLDYHTEF	HLA-A*32:01	10	0.16655	100.00% (10/10)	Non-Toxin	1.46	1.24	-1.92	2.69	0.77	83.2
337	polymerase	KSFLDYHTEF	HLA-A*23:01	10	0.16655	100.00% (10/10)	Non-Toxin	1.46	1.24	-2.01	2.69	0.68	102.4

#	Protein	Peptide	Allele	Length	Immunogenicity	Conservancy	Toxicity	Proteasome Score	TAP Score	MHC Score	Processing Score	Total Score	MHC IC50[nM]
338	polymerase	KSMSRCCFW	HLA-B*58:01	9	-0.20092	90.00% (9/10)	Non-Toxin	1.46	0.5	-0.69	1.96	1.27	4.9
339	polymerase	KSMSRCCFW	HLA-B*57:01	9	-0.20092	90.00% (9/10)	Non-Toxin	1.46	0.5	-0.95	1.96	1	9
340	polymerase	KSRELDFPLW	HLA-B*58:01	10	0.22271	100.00% (10/10)	Non-Toxin	1.56	0.49	-0.74	2.05	1.31	5.5
341	polymerase	KSRELDFPLW	HLA-B*57:01	10	0.22271	100.00% (10/10)	Non-Toxin	1.56	0.49	-0.83	2.05	1.22	6.7
342	polymerase	KTIKNITAR	HLA-A*31:01	9	0.0008	100.00% (10/10)	Non-Toxin	1.13	0.72	-1.13	1.85	0.72	13.4
343	polymerase	KTWTIATIPF	HLA-A*32:01	10	0.42104	100.00% (10/10)	Non-Toxin	0.97	1.19	-1.03	2.16	1.13	10.7
344	polymerase	KTWTIATIPF	HLA-B*58:01	10	0.42104	100.00% (10/10)	Non-Toxin	0.97	1.19	-1.45	2.16	0.71	28.2
345	polymerase	KVIVYSLIKF	HLA-A*32:01	10	-0.10408	100.00% (10/10)	Non-Toxin	1.56	1.28	-2.22	2.84	0.62	164.9
346	polymerase	KVIVYSLIKF	HLA-B*15:01	10	-0.10408	100.00% (10/10)	Non-Toxin	1.56	1.28	-2.26	2.84	0.58	181.5
347	polymerase	KWYECFLF	HLA-A*23:01	8	0.14789	100.00% (10/10)	Non-Toxin	1.15	1.36	-1.12	2.51	1.38	13.3
348	polymerase	KWYECFLF	HLA-A*24:02	8	0.14789	100.00% (10/10)	Non-Toxin	1.15	1.36	-1.55	2.51	0.96	35.1
349	polymerase	KWYECFLFW	HLA-A*23:01	9	0.21629	100.00% (10/10)	Non-Toxin	1.52	0.64	-1	2.16	1.16	10.1
350	polymerase	KWYECFLFW	HLA-A*24:02	9	0.21629	100.00% (10/10)	Non-Toxin	1.52	0.64	-1.5	2.16	0.66	31.6
351	polymerase	KWYECFLFWF	HLA-A*23:01	10	0.37883	100.00% (10/10)	Non-Toxin	1.24	1.36	-1	2.6	1.6	10.1
352	polymerase	KWYECFLFWF	HLA-A*24:02	10	0.37883	100.00% (10/10)	Non-Toxin	1.24	1.36	-1.5	2.6	1.1	31.4
353	polymerase	KYYQIDQPF	HLA-A*23:01	9	-0.07152	100.00% (10/10)	Non-Toxin	0.86	1.39	-0.95	2.24	1.29	9
354	polymerase	KYYQIDQPF	HLA-A*24:02	9	-0.07152	100.00% (10/10)	Non-Toxin	0.86	1.39	-1.24	2.24	1	17.3
355	polymerase	KYYQIDQPFF	HLA-A*23:01	10	-0.01656	100.00% (10/10)	Non-Toxin	1.33	1.39	-0.98	2.72	1.73	9.6
356	polymerase	KYYQIDQPFF	HLA-A*24:02	10	-0.01656	100.00% (10/10)	Non-Toxin	1.33	1.39	-1.3	2.72	1.42	19.8
357	polymerase	LAEFFSFFR	HLA-A*68:01	9	0.27577	100.00% (10/10)	Non-Toxin	1.02	0.63	-0.89	1.64	0.75	7.8
358	polymerase	LARALRSHMW	HLA-B*58:01	10	-0.13526	100.00% (10/10)	Non-Toxin	1.6	0.37	-1.14	1.96	0.82	13.9
359	polymerase	LARALRSHMW	HLA-B*57:01	10	-0.13526	100.00% (10/10)	Non-Toxin	1.6	0.37	-1.31	1.96	0.65	20.5
360	polymerase	LETDDYNGIY	HLA-A*01:01	10	0.15319	100.00% (10/10)	Non-Toxin	1.41	1.25	-0.93	2.66	1.73	8.6
361	polymerase	LFPVMGNRIY	HLA-B*35:01	10	0.01529	100.00% (10/10)	Non-Toxin	1.57	1.28	-1.68	2.85	1.16	48.4
362	polymerase	LIASGVGKY	HLA-A*30:02	9	-0.17931	100.00% (10/10)	Non-Toxin	1.13	1.4	-1.68	2.53	0.85	47.7
363	polymerase	LIASGVGKY	HLA-B*15:01	9	-0.17931	100.00% (10/10)	Non-Toxin	1.13	1.4	-1.8	2.53	0.73	63
364	polymerase	LIASGVGKYF	HLA-B*15:01	10	-0.23283	100.00% (10/10)	Non-Toxin	1.41	1.23	-1.63	2.64	1.01	42.4
365	polymerase	LISRSNALW	HLA-B*58:01	9	-0.14227	100.00% (10/10)	Non-Toxin	1.35	0.38	-1.06	1.73	0.67	11.5
366	polymerase	LKSMSRCCFW	HLA-B*58:01	10	-0.36895	90.00% (9/10)	Non-Toxin	1.46	0.47	-1.14	1.93	0.8	13.7
367	polymerase	LLAEFFSFF	HLA-B*15:01	9	0.26643	100.00% (10/10)	Non-Toxin	1.37	1.11	-1.2	2.47	1.27	16
368	polymerase	LLAEFFSFFR	HLA-A*68:01	10	0.35292	100.00% (10/10)	Non-Toxin	1.02	0.65	-0.99	1.67	0.68	9.7
369	polymerase	LLLPTQGPY	HLA-B*15:01	9	-0.06388	100.00% (10/10)	Non-Toxin	0.86	1.28	-1.47	2.13	0.67	29.2
370	polymerase	LLVTSYMIY	HLA-B*15:01	9	-0.18256	100.00% (10/10)	Non-Toxin	1.33	1.38	-1.32	2.71	1.38	21.1
371	polymerase	LLVTSYMIYL	HLA-A*02:01	10	-0.16738	100.00% (10/10)	Non-Toxin	1.53	0.52	-1.48	2.05	0.57	30.2
372	polymerase	LPRAAHEIL	HLA-B*07:02	9	0.28698	100.00% (10/10)	Non-Toxin	1.42	0.31	-1.02	1.74	0.72	10.4
373	polymerase	LPSGAQRLF	HLA-B*35:01	9	-0.05334	100.00% (10/10)	Non-Toxin	1.2	0.95	-1.5	2.15	0.64	31.9
374	polymerase	LSHPRVFKR	HLA-A*31:01	9	0.0614	100.00% (10/10)	Non-Toxin	1.07	0.74	-1.11	1.81	0.7	12.8
375	polymerase	LSNREVKIWW	HLA-B*58:01	10	0.22642	100.00% (10/10)	Non-Toxin	1.48	0.3	-1.24	1.78	0.54	17.5
376	polymerase	LSQNLLVTSY	HLA-B*15:01	10	-0.09075	100.00% (10/10)	Non-Toxin	1.6	1.32	-1.52	2.91	1.4	32.8
377	polymerase	LSQNLLVTSY	HLA-A*30:02	10	-0.09075	100.00% (10/10)	Non-Toxin	1.6	1.32	-2.07	2.91	0.85	116.6
378	polymerase	LTIDDCVKNW	HLA-B*58:01	10	-0.1123	100.00% (10/10)	Non-Toxin	1.5	0.43	-1.42	1.93	0.51	26.1
379	polymerase	LTPEMVLMY	HLA-A*01:01	9	-0.14695	100.00% (10/10)	Non-Toxin	1.52	1.23	-1.97	2.75	0.78	93.8
380	polymerase	LVLVCFPVY	HLA-B*35:01	9	0.1104	100.00% (10/10)	Non-Toxin	1.54	1.33	-2.25	2.86	0.61	176.8
381	polymerase	LVSNTSKHTY	HLA-B*15:01	10	-0.33653	100.00% (10/10)	Non-Toxin	1.52	1.27	-2.05	2.79	0.74	113.5
382	polymerase	LVSNTSKHTY	HLA-A*30:02	10	-0.33653	100.00% (10/10)	Non-Toxin	1.52	1.27	-2.11	2.79	0.68	129.8
383	polymerase	LWGLIDPLF	HLA-A*23:01	9	0.13448	100.00% (10/10)	Non-Toxin	1.16	1.2	-1.35	2.37	1.02	22.4
384	polymerase	LWGLIDPLF	HLA-A*24:02	9	0.13448	100.00% (10/10)	Non-Toxin	1.16	1.2	-1.59	2.37	0.77	39.2
385	polymerase	MIYLMNWCDF	HLA-A*23:01	10	-0.01369	100.00% (10/10)	Non-Toxin	0.96	1.23	-1.46	2.2	0.74	28.8
386	polymerase	MIYLMNWCDF	HLA-A*24:02	10	-0.01369	100.00% (10/10)	Non-Toxin	0.96	1.23	-1.66	2.2	0.54	45.6

#	Protein	Peptide	Allele	Length	Immunogenicity	Conservancy	Toxicity	Proteasome Score	TAP Score	MHC Score	Processing Score	Total Score	MHC IC50[nM]
387	polymerase	MLADKVLEY	HLA-B*15:01	9	-0.08698	100.00% (10/10)	Non-Toxin	1.56	1.3	-1.39	2.86	1.47	24.3
388	polymerase	MLADKVLEY	HLA-B*35:01	9	-0.08698	100.00% (10/10)	Non-Toxin	1.56	1.3	-1.45	2.86	1.4	28.4
389	polymerase	MLADKVLEY	HLA-A*03:01	9	-0.08698	100.00% (10/10)	Non-Toxin	1.56	1.3	-2.19	2.86	0.67	153.6
390	polymerase	MLADKVLEY	HLA-A*30:02	9	-0.08698	100.00% (10/10)	Non-Toxin	1.56	1.3	-2.26	2.86	0.6	180
391	polymerase	MLADKVLEY	HLA-A*01:01	9	-0.08698	100.00% (10/10)	Non-Toxin	1.56	1.3	-2.33	2.86	0.52	215.1
392	polymerase	MLEYVLSGAY	HLA-B*15:01	10	-0.04609	100.00% (10/10)	Non-Toxin	1.04	1.3	-1.37	2.33	0.97	23.3
393	polymerase	MLEYVLSGAY	HLA-A*30:02	10	-0.04609	100.00% (10/10)	Non-Toxin	1.04	1.3	-1.8	2.33	0.53	63.8
394	polymerase	MLNEAMNYF	HLA-B*15:01	9	-0.03617	100.00% (10/10)	Non-Toxin	1.38	1.09	-1.05	2.47	1.42	11.2
395	polymerase	MLNEAMNYF	HLA-B*35:01	9	-0.03617	100.00% (10/10)	Non-Toxin	1.38	1.09	-1.77	2.47	0.7	58.9
396	polymerase	MMLLYQSTL	HLA-A*02:01	9	-0.24434	100.00% (10/10)	Non-Toxin	1.51	0.52	-1.34	2.03	0.69	22
397	polymerase	MMLLYQSTL	HLA-A*02:03	9	-0.24434	100.00% (10/10)	Non-Toxin	1.51	0.52	-1.53	2.03	0.51	33.5
398	polymerase	MYCDVLEGR	HLA-A*33:01	9	0.13888	100.00% (10/10)	Non-Toxin	1.17	0.8	-1.45	1.97	0.52	28.4
399	polymerase	MYGWFFVPR	HLA-A*33:01	9	0.48645	100.00% (10/10)	Non-Toxin	0.67	0.71	-0.83	1.38	0.55	6.8
400	polymerase	NIMKKSFKAY	HLA-B*15:01	10	-0.69404	100.00% (10/10)	Non-Toxin	1.59	1.31	-2.05	2.9	0.86	111.2
401	polymerase	NIRRKVLIL	HLA-B*08:01	9	-0.03386	100.00% (10/10)	Non-Toxin	1.44	0.57	-1.19	2.01	0.82	15.5
402	polymerase	NLHGKRKSL	HLA-B*08:01	9	-0.39534	100.00% (10/10)	Non-Toxin	1.84	0.48	-1.7	2.31	0.61	50.3
403	polymerase	NTCSVDLAR	HLA-A*68:01	9	-0.10939	100.00% (10/10)	Non-Toxin	1.14	0.68	-0.96	1.82	0.86	9.2
404	polymerase	NTEAAVLSRY	HLA-A*26:01	10	0.03035	100.00% (10/10)	Non-Toxin	1.46	1.15	-1.77	2.61	0.84	58.9
405	polymerase	NTEAAVLSRY	HLA-A*01:01	10	0.03035	100.00% (10/10)	Non-Toxin	1.46	1.15	-1.91	2.61	0.7	80.7
406	polymerase	NTMYGWFFV	HLA-A*68:02	9	0.34799	100.00% (10/10)	Non-Toxin	0.84	0.16	-0.41	0.99	0.58	2.6
407	polymerase	NTSDLDFVIFY	HLA-A*01:01	11	0.313	100.00% (10/10)	Non-Toxin	1.68	1.25	-1.76	2.93	1.17	57.2
408	polymerase	NTSKSFLDY	HLA-A*01:01	9	-0.318	100.00% (10/10)	Non-Toxin	1.17	1.3	-1.73	2.47	0.74	54.1
409	polymerase	NYLNLSRIF	HLA-A*23:01	9	-0.0552	100.00% (10/10)	Non-Toxin	1.29	1.19	-1.74	2.48	0.74	55.1
410	polymerase	QQLLISTEF	HLA-B*15:01	9	0.05037	100.00% (10/10)	Non-Toxin	1.56	1.15	-1.95	2.71	0.76	89.6
411	polymerase	QSISIKWAF	HLA-B*58:01	9	0.01313	100.00% (10/10)	Non-Toxin	1.4	1.14	-1.51	2.54	1.03	32.4
412	polymerase	QSISIKWAF	HLA-A*32:01	9	0.01313	100.00% (10/10)	Non-Toxin	1.4	1.14	-1.9	2.54	0.64	79.3
413	polymerase	RALRSHMW	HLA-B*58:01	8	-0.23037	100.00% (10/10)	Non-Toxin	1.6	0.59	-1.63	2.18	0.55	42.4
414	polymerase	RALRSHMWR	HLA-A*31:01	9	-0.10095	100.00% (10/10)	Non-Toxin	0.93	0.85	-0.83	1.78	0.95	6.8
415	polymerase	REHMLADKVL	HLA-B*40:01	10	-0.2759	100.00% (10/10)	Non-Toxin	1.74	0.5	-1.39	2.23	0.84	24.6
416	polymerase	RELALGRVIY	HLA-B*44:03	10	0.21929	100.00% (10/10)	Non-Toxin	1.61	1.3	-1.8	2.91	1.1	63.4
417	polymerase	RELALGRVIY	HLA-B*44:02	10	0.21929	100.00% (10/10)	Non-Toxin	1.61	1.3	-2.06	2.91	0.84	115.3
418	polymerase	RELALGRVIY	HLA-B*15:01	10	0.21929	100.00% (10/10)	Non-Toxin	1.61	1.3	-2.13	2.91	0.78	133.8
419	polymerase	RELKLFPSEY	HLA-B*44:03	10	-0.20896	100.00% (10/10)	Non-Toxin	1.48	1.3	-1.66	2.78	1.13	45.3
420	polymerase	RELKLFPSEY	HLA-B*44:02	10	-0.20896	100.00% (10/10)	Non-Toxin	1.48	1.3	-1.88	2.78	0.91	75.5
421	polymerase	RELKLFPSEY	HLA-A*30:02	10	-0.20896	100.00% (10/10)	Non-Toxin	1.48	1.3	-2.14	2.78	0.65	136.5
422	polymerase	RELKLFPSEY	HLA-B*15:01	10	-0.20896	100.00% (10/10)	Non-Toxin	1.48	1.3	-2.16	2.78	0.63	143.4
423	polymerase	RIATVYTW	HLA-B*58:01	8	0.12124	100.00% (10/10)	Non-Toxin	1.73	0.56	-1.7	2.29	0.59	50.4
424	polymerase	RIATVYTWAY	HLA-A*30:02	10	0.3347	100.00% (10/10)	Non-Toxin	1.56	1.44	-1.54	3	1.46	35
425	polymerase	RIATVYTWAY	HLA-B*15:01	10	0.3347	100.00% (10/10)	Non-Toxin	1.56	1.44	-1.69	3	1.31	49.3
426	polymerase	RIATVYTWAY	HLA-B*58:01	10	0.3347	100.00% (10/10)	Non-Toxin	1.56	1.44	-2.19	3	0.81	156.3
427	polymerase	RIATVYTWAY	HLA-A*32:01	10	0.3347	100.00% (10/10)	Non-Toxin	1.56	1.44	-2.31	3	0.69	203.2
428	polymerase	RIATVYTWAY	HLA-A*03:01	10	0.3347	100.00% (10/10)	Non-Toxin	1.56	1.44	-2.49	3	0.51	309.1
429	polymerase	RIGLNSSSCY	HLA-A*30:02	10	-0.49441	100.00% (10/10)	Non-Toxin	1.41	1.42	-1.8	2.83	1.03	63.7
430	polymerase	RLETDDYNGIY	HLA-A*01:01	11	0.21143	100.00% (10/10)	Non-Toxin	1.41	1.34	-1.76	2.75	0.99	57.9
431	polymerase	RLFAKMTY	HLA-A*03:01	8	-0.26517	100.00% (10/10)	Non-Toxin	1.39	1.42	-2.31	2.81	0.5	205
432	polymerase	RLFAKMTYK	HLA-A*03:01	9	-0.26733	100.00% (10/10)	Non-Toxin	0.76	0.35	-0.53	1.11	0.58	3.4
433	polymerase	RLFAKMTYKMR	HLA-A*31:01	11	-0.55391	100.00% (10/10)	Non-Toxin	1.25	0.79	-1.16	2.04	0.88	14.6
434	polymerase	RLFNMYRSY	HLA-A*32:01	9	-0.19597	100.00% (10/10)	Non-Toxin	1.42	1.46	-1.11	2.88	1.77	12.8
435	polymerase	RLFNMYRSY	HLA-B*15:01	9	-0.19597	100.00% (10/10)	Non-Toxin	1.42	1.46	-1.18	2.88	1.69	15.3

#	Protein	Peptide	Allele	Length	Immunogenicity	Conservancy	Toxicity	Proteasome Score	TAP Score	MHC Score	Processing Score	Total Score	MHC IC50[nM]
436	polymerase	RLFNMYRSY	HLA-A*30:02	9	-0.19597	100.00% (10/10)	Non-Toxin	1.42	1.46	-1.2	2.88	1.68	15.8
437	polymerase	RLFNMYRSY	HLA-A*03:01	9	-0.19597	100.00% (10/10)	Non-Toxin	1.42	1.46	-1.52	2.88	1.36	33.1
438	polymerase	RLFNMYRSY	HLA-A*30:01	9	-0.19597	100.00% (10/10)	Non-Toxin	1.42	1.46	-2.19	2.88	0.69	155
439	polymerase	RLFNMYRSYF	HLA-A*32:01	10	-0.23617	100.00% (10/10)	Non-Toxin	1.16	1.29	-1.2	2.45	1.25	15.7
440	polymerase	RLFNMYRSYF	HLA-B*15:01	10	-0.23617	100.00% (10/10)	Non-Toxin	1.16	1.29	-1.34	2.45	1.1	22
441	polymerase	RLRDKSTQF	HLA-B*15:01	9	-0.36153	100.00% (10/10)	Non-Toxin	1.43	1.23	-1.6	2.65	1.06	39.5
442	polymerase	RLRLETDDY	HLA-A*30:02	9	0.17136	100.00% (10/10)	Non-Toxin	1.19	1.36	-1.98	2.55	0.57	96.4
443	polymerase	RLRMNLRAL	HLA-B*07:02	9	-0.1101	100.00% (10/10)	Non-Toxin	1.6	0.48	-1.32	2.08	0.76	20.9
444	polymerase	RLSHHDYNQF	HLA-B*15:01	10	-0.04467	100.00% (10/10)	Non-Toxin	1.48	1.24	-2.2	2.71	0.51	159.5
445	polymerase	RPRGRHTM	HLA-B*07:02	8	0.16451	100.00% (10/10)	Non-Toxin	1.31	0.18	-0.89	1.5	0.61	7.8
446	polymerase	RPRGRHTMV	HLA-B*07:02	9	0.06191	100.00% (10/10)	Non-Toxin	1.1	0.19	-0.64	1.29	0.64	4.4
447	polymerase	RPTKALRSAI	HLA-B*07:02	10	-0.24514	100.00% (10/10)	Non-Toxin	1.24	0.23	-0.68	1.48	0.8	4.8
448	polymerase	RSAIRIATVY	HLA-B*58:01	10	0.42033	100.00% (10/10)	Non-Toxin	1.43	1.42	-1.36	2.85	1.49	22.9
449	polymerase	RSAIRIATVY	HLA-A*30:02	10	0.42033	100.00% (10/10)	Non-Toxin	1.43	1.42	-1.5	2.85	1.36	31.4
450	polymerase	RSAIRIATVY	HLA-B*15:01	10	0.42033	100.00% (10/10)	Non-Toxin	1.43	1.42	-1.77	2.85	1.09	58.3
451	polymerase	RSAIRIATVY	HLA-B*57:01	10	0.42033	100.00% (10/10)	Non-Toxin	1.43	1.42	-1.9	2.85	0.95	80.2
452	polymerase	RSMFIDDLL	HLA-B*58:01	9	0.22352	100.00% (10/10)	Non-Toxin	1.55	0.57	-1.16	2.12	0.96	14.5
453	polymerase	RSPSHHLEPY	HLA-A*30:02	10	-0.03949	100.00% (10/10)	Non-Toxin	0.99	1.45	-1.73	2.44	0.7	54.3
454	polymerase	RSWNTSDLDF	HLA-B*58:01	10	-0.03343	100.00% (10/10)	Non-Toxin	1.21	1.32	-1.41	2.53	1.12	26
455	polymerase	RSWNTSDLDF	HLA-B*57:01	10	-0.03343	100.00% (10/10)	Non-Toxin	1.21	1.32	-1.91	2.53	0.63	80.5
456	polymerase	RSYFGLVLVCF	HLA-A*23:01	11	0.1719	100.00% (10/10)	Non-Toxin	1.48	1.28	-2.23	2.76	0.53	168.4
457	polymerase	RYESMAIFAER	HLA-A*31:01	11	0.06445	100.00% (10/10)	Non-Toxin	1	0.78	-1.21	1.78	0.56	16.4
458	polymerase	RYKCSNTSK	HLA-A*30:01	9	-0.35534	100.00% (10/10)	Non-Toxin	0.98	0.48	-0.86	1.46	0.6	7.2
459	polymerase	RYLPSGAQRLF	HLA-A*23:01	11	-0.1766	100.00% (10/10)	Non-Toxin	1.2	1.31	-1.64	2.51	0.86	44.1
460	polymerase	RYLPSGAQRLF	HLA-A*24:02	11	-0.1766	100.00% (10/10)	Non-Toxin	1.2	1.31	-1.9	2.51	0.61	78.6
461	polymerase	SAIEYAQLR	HLA-A*68:01	9	0.07294	100.00% (10/10)	Non-Toxin	0.94	0.72	-1.1	1.66	0.57	12.5
462	polymerase	SAIRIATVY	HLA-B*35:01	9	0.31859	100.00% (10/10)	Non-Toxin	1.43	1.4	-1.41	2.84	1.42	25.8
463	polymerase	SAIRIATVY	HLA-B*15:01	9	0.31859	100.00% (10/10)	Non-Toxin	1.43	1.4	-1.93	2.84	0.9	85.3
464	polymerase	SAIRIATVY	HLA-A*30:02	9	0.31859	100.00% (10/10)	Non-Toxin	1.43	1.4	-2.2	2.84	0.63	158.7
465	polymerase	SAIRIATVYTW	HLA-B*58:01	11	0.3592	100.00% (10/10)	Non-Toxin	1.73	0.52	-1.06	2.25	1.19	11.5
466	polymerase	SAIRIATVYTW	HLA-B*57:01	11	0.3592	100.00% (10/10)	Non-Toxin	1.73	0.52	-1.54	2.25	0.71	34.7
467	polymerase	SFCGIQFDCF	HLA-A*23:01	10	0.13082	100.00% (10/10)	Non-Toxin	1.49	1.13	-2.02	2.61	0.59	105.7
468	polymerase	SFLDYHTEF	HLA-A*23:01	9	0.13683	100.00% (10/10)	Non-Toxin	1.46	1.25	-1.55	2.71	1.16	35.2
469	polymerase	SFLDYHTEF	HLA-A*24:02	9	0.13683	100.00% (10/10)	Non-Toxin	1.46	1.25	-1.98	2.71	0.72	96.2
470	polymerase	SMFIDDLLSI	HLA-A*02:03	10	0.09866	100.00% (10/10)	Non-Toxin	1.16	0.41	-0.81	1.58	0.76	6.5
471	polymerase	SMIEPLVLAL	HLA-A*02:03	10	0.17471	100.00% (10/10)	Non-Toxin	1.46	0.54	-1.16	2	0.84	14.3
472	polymerase	SMIEPLVLAL	HLA-A*02:01	10	0.17471	100.00% (10/10)	Non-Toxin	1.46	0.54	-1.28	2	0.72	19
473	polymerase	SMIEPLVLAL	HLA-A*02:06	10	0.17471	100.00% (10/10)	Non-Toxin	1.46	0.54	-1.36	2	0.64	23
474	polymerase	SPSHHLEPY	HLA-B*35:01	9	0.07793	100.00% (10/10)	Non-Toxin	0.99	1.22	-1.02	2.21	1.19	10.4
475	polymerase	SQNLLVTSY	HLA-B*15:01	9	-0.0491	100.00% (10/10)	Non-Toxin	1.6	1.31	-1.38	2.9	1.52	24.2
476	polymerase	SQNLLVTSY	HLA-A*30:02	9	-0.0491	100.00% (10/10)	Non-Toxin	1.6	1.31	-1.87	2.9	1.03	74.7
477	polymerase	SQSKWYECF	HLA-B*15:01	9	-0.00548	100.00% (10/10)	Non-Toxin	1.23	1.17	-1.85	2.4	0.55	70
478	polymerase	SRLFNMYRSY	HLA-B*15:01	10	-0.11956	100.00% (10/10)	Non-Toxin	1.42	1.39	-1.69	2.82	1.13	49
479	polymerase	SRLFNMYRSY	HLA-A*30:02	10	-0.11956	100.00% (10/10)	Non-Toxin	1.42	1.39	-1.94	2.82	0.87	87.5
480	polymerase	SRLFNMYRSY	HLA-A*03:01	10	-0.11956	100.00% (10/10)	Non-Toxin	1.42	1.39	-2.24	2.82	0.57	174.8
481	polymerase	STHLFIYSK	HLA-A*11:01	9	0.13884	90.00% (9/10)	Non-Toxin	1.13	0.23	-0.72	1.36	0.64	5.3
482	polymerase	STLGQSISF	HLA-A*32:01	9	-0.22237	100.00% (10/10)	Non-Toxin	1.44	1.1	-1.63	2.54	0.91	42.7
483	polymerase	STLGQSISFY	HLA-A*30:02	10	-0.18934	100.00% (10/10)	Non-Toxin	1.32	1.27	-1.57	2.59	1.02	36.9
484	polymerase	STLGQSISFY	HLA-A*11:01	10	-0.18934	100.00% (10/10)	Non-Toxin	1.32	1.27	-1.65	2.59	0.94	44.4

#	Protein	Peptide	Allele	Length	Immunogenicity	Conservancy	Toxicity	Proteasome Score	TAP Score	MHC Score	Processing Score	Total Score	MHC IC50[nM]
485	polymerase	SVLNRVSRY	HLA-A*30:02	9	-0.03023	100.00% (10/10)	Non-Toxin	1.26	1.35	-1.63	2.61	0.97	43.1
486	polymerase	SVYPREVLSY	HLA-A*03:01	10	0.06838	100.00% (10/10)	Non-Toxin	1.31	1.37	-1.52	2.68	1.16	32.8
487	polymerase	SVYPREVLSY	HLA-A*11:01	10	0.06838	100.00% (10/10)	Non-Toxin	1.31	1.37	-1.53	2.68	1.15	34
488	polymerase	SVYPREVLSY	HLA-B*15:01	10	0.06838	100.00% (10/10)	Non-Toxin	1.31	1.37	-1.68	2.68	1	48.2
489	polymerase	SVYPREVLSY	HLA-A*30:02	10	0.06838	100.00% (10/10)	Non-Toxin	1.31	1.37	-1.87	2.68	0.81	73.5
490	polymerase	SYFGLVLVCF	HLA-A*23:01	10	0.0944	100.00% (10/10)	Non-Toxin	1.48	1.34	-1.04	2.82	1.78	10.9
491	polymerase	SYFGLVLVCF	HLA-A*24:02	10	0.0944	100.00% (10/10)	Non-Toxin	1.48	1.34	-1.51	2.82	1.31	32.6
492	polymerase	SYMIYLMNW	HLA-A*23:01	9	-0.0891	100.00% (10/10)	Non-Toxin	1.53	0.55	-1.07	2.09	1.02	11.7
493	polymerase	SYMIYLMNW	HLA-A*24:02	9	-0.0891	100.00% (10/10)	Non-Toxin	1.53	0.55	-1.31	2.09	0.78	20.2
494	polymerase	TEAAVLSRY	HLA-B*44:03	9	-0.02755	100.00% (10/10)	Non-Toxin	1.46	1.27	-1.57	2.73	1.16	37.2
495	polymerase	TEAAVLSRY	HLA-B*44:02	9	-0.02755	100.00% (10/10)	Non-Toxin	1.46	1.27	-1.82	2.73	0.91	66.5
496	polymerase	TEFNPHNHY	HLA-B*44:03	9	0.06458	100.00% (10/10)	Non-Toxin	1.36	1.24	-2.09	2.6	0.51	123.6
497	polymerase	TEFSINETL	HLA-B*40:01	9	0.10222	100.00% (10/10)	Non-Toxin	1.6	0.42	-1.14	2.02	0.88	13.7
498	polymerase	TEVDNNHLIY	HLA-A*01:01	10	0.12197	100.00% (10/10)	Non-Toxin	1.3	1.21	-1.57	2.52	0.95	37.2
499	polymerase	TIATIPFLF	HLA-A*23:01	9	0.26324	100.00% (10/10)	Non-Toxin	1.08	1.13	-1.61	2.21	0.6	40.9
500	polymerase	TIISTHLFIY	HLA-A*11:01	10	0.11215	90.00% (9/10)	Non-Toxin	1.26	1.27	-1.98	2.53	0.55	95.7
501	polymerase	TIPFLFLSAY	HLA-B*35:01	10	0.0902	100.00% (10/10)	Non-Toxin	1.39	1.38	-1.6	2.77	1.17	40
502	polymerase	TMVDLLSDL	HLA-A*02:03	9	-0.11218	100.00% (10/10)	Non-Toxin	1.2	0.48	-1.16	1.69	0.53	14.3
503	polymerase	TMYGWFFVPR	HLA-A*31:01	10	0.50116	100.00% (10/10)	Non-Toxin	0.67	0.83	-0.72	1.5	0.78	5.2
504	polymerase	TMYGWFFVPR	HLA-A*33:01	10	0.50116	100.00% (10/10)	Non-Toxin	0.67	0.83	-0.81	1.5	0.69	6.4
505	polymerase	TQKVHPNLPY	HLA-B*15:01	10	-0.02969	100.00% (10/10)	Non-Toxin	1	1.3	-1.43	2.3	0.87	26.8
506	polymerase	TSDLDFVIFY	HLA-A*01:01	10	0.35122	100.00% (10/10)	Non-Toxin	1.68	1.26	-0.87	2.94	2.07	7.4
507	polymerase	TSDLDFVIFY	HLA-A*11:01	10	0.35122	100.00% (10/10)	Non-Toxin	1.68	1.26	-2.31	2.94	0.63	203.7
508	polymerase	TSYMIYLMNW	HLA-B*58:01	10	-0.21432	100.00% (10/10)	Non-Toxin	1.53	0.49	-1.33	2.03	0.7	21.4
509	polymerase	TTWIGNLDSY	HLA-A*26:01	10	0.14414	100.00% (10/10)	Non-Toxin	1.29	1.25	-1.93	2.54	0.61	85.6
510	polymerase	TWTIATIPFLF	HLA-A*23:01	11	0.4339	100.00% (10/10)	Non-Toxin	1.08	1.23	-1.69	2.31	0.62	48.5
511	polymerase	VIFYASLTY	HLA-B*15:01	9	-0.07003	100.00% (10/10)	Non-Toxin	1.22	1.36	-1.45	2.58	1.13	28.2
512	polymerase	VIFYASLTY	HLA-B*35:01	9	-0.07003	100.00% (10/10)	Non-Toxin	1.22	1.36	-1.47	2.58	1.11	29.4
513	polymerase	VIFYASLTY	HLA-A*30:02	9	-0.07003	100.00% (10/10)	Non-Toxin	1.22	1.36	-1.65	2.58	0.92	45.1
514	polymerase	VIFYASLTY	HLA-A*03:01	9	-0.07003	100.00% (10/10)	Non-Toxin	1.22	1.36	-1.74	2.58	0.84	55.3
515	polymerase	VIFYASLTY	HLA-A*11:01	9	-0.07003	100.00% (10/10)	Non-Toxin	1.22	1.36	-1.85	2.58	0.73	70
516	polymerase	VIFYASLTY	HLA-A*32:01	9	-0.07003	100.00% (10/10)	Non-Toxin	1.22	1.36	-1.95	2.58	0.63	88.6
517	polymerase	VMMEDGLLV	HLA-A*02:01	9	0.08141	100.00% (10/10)	Non-Toxin	0.91	0.3	-0.69	1.21	0.52	4.9
518	polymerase	VSNTSKHTY	HLA-A*30:02	9	-0.27716	100.00% (10/10)	Non-Toxin	1.52	1.29	-1.89	2.82	0.93	76.8
519	polymerase	VSRLSHHDY	HLA-A*30:02	9	-0.08475	100.00% (10/10)	Non-Toxin	1.17	1.36	-1.98	2.52	0.54	95.8
520	polymerase	VTSYMIYLMNW	HLA-B*58:01	11	-0.26484	100.00% (10/10)	Non-Toxin	1.53	0.42	-1.41	1.95	0.55	25.7
521	polymerase	WAFEIHHRR	HLA-A*68:01	9	0.35634	100.00% (10/10)	Non-Toxin	0.86	0.75	-0.6	1.61	1.01	4
522	polymerase	WAYGDNEECW	HLA-B*58:01	10	0.19545	100.00% (10/10)	Non-Toxin	1.63	0.48	-1.39	2.11	0.71	24.7
523	polymerase	WESFCGIQF	HLA-B*40:01	9	0.08814	100.00% (10/10)	Non-Toxin	1.27	1.09	-1.77	2.36	0.59	58.3
524	polymerase	WESFCGIQF	HLA-B*44:03	9	0.08814	100.00% (10/10)	Non-Toxin	1.27	1.09	-1.85	2.36	0.51	70.6
525	polymerase	WTIATIPFL	HLA-A*02:06	9	0.30469	100.00% (10/10)	Non-Toxin	1.53	0.43	-0.76	1.96	1.2	5.8
526	polymerase	WTIATIPFL	HLA-A*68:02	9	0.30469	100.00% (10/10)	Non-Toxin	1.53	0.43	-1.24	1.96	0.72	17.4
527	polymerase	WTIATIPFL	HLA-A*02:01	9	0.30469	100.00% (10/10)	Non-Toxin	1.53	0.43	-1.37	1.96	0.59	23.5
528	polymerase	WTIATIPFLF	HLA-B*58:01	10	0.33185	100.00% (10/10)	Non-Toxin	1.08	1.11	-1.25	2.19	0.94	17.8
529	polymerase	YASLTYLRR	HLA-A*68:01	9	-0.00966	100.00% (10/10)	Non-Toxin	1.29	0.61	-1.35	1.9	0.56	22.2
530	polymerase	YESMAIFAER	HLA-A*68:01	10	0.13902	100.00% (10/10)	Non-Toxin	1	0.63	-0.76	1.63	0.87	5.8
531	polymerase	YGLPGFFNW	HLA-B*58:01	9	0.22346	100.00% (10/10)	Non-Toxin	1.52	0.27	-1.12	1.8	0.67	13.2
532	polymerase	YIINRTAGR	HLA-A*68:01	9	0.17645	100.00% (10/10)	Non-Toxin	1.16	0.65	-1.07	1.81	0.74	11.8
533	polymerase	YLPSGAQRL	HLA-A*02:03	9	-0.16776	100.00% (10/10)	Non-Toxin	1.69	0.47	-1.62	2.15	0.53	42.1

#	Protein	Peptide	Allele	Length	Immunogenicity	Conservancy	Toxicity	Proteasome Score	TAP Score	MHC Score	Processing Score	Total Score	MHC IC50[nM]
534	polymerase	YLRRGIIKQL	HLA-A*02:03	10	0.10708	100.00% (10/10)	Non-Toxin	1.81	0.44	-1.51	2.25	0.74	32.3
535	polymerase	YLTPEMVLMY	HLA-B*15:01	10	-0.14516	100.00% (10/10)	Non-Toxin	1.52	1.23	-2.16	2.75	0.58	145.9
536	polymerase	YMKDKALSPI	HLA-A*02:03	10	-0.37612	100.00% (10/10)	Non-Toxin	0.84	0.29	-0.59	1.13	0.54	3.9
537	polymerase	YPECNNILF	HLA-B*35:01	9	0.0717	90.00% (9/10)	Non-Toxin	1.47	0.96	-0.82	2.44	1.62	6.6
538	polymerase	YPECNNILF	HLA-B*53:01	9	0.0717	90.00% (9/10)	Non-Toxin	1.47	0.96	-1.26	2.44	1.18	18.2
539	polymerase	YPKGGIEGY	HLA-B*35:01	9	0.22668	100.00% (10/10)	Non-Toxin	1.35	1.12	-1.28	2.47	1.2	18.9
540	polymerase	YPREVLSY	HLA-B*35:01	8	0.00769	100.00% (10/10)	Non-Toxin	1.31	1.17	-1.84	2.48	0.64	69.2
541	polymerase	YQIDQPFFV	HLA-A*02:06	9	0.10948	100.00% (10/10)	Non-Toxin	1.07	0.17	-0.2	1.24	1.03	1.6
542	polymerase	YQIDQPFFV	HLA-A*02:01	9	0.10948	100.00% (10/10)	Non-Toxin	1.07	0.17	-0.34	1.24	0.9	2.2
543	polymerase	YQQAMLLGL	HLA-A*02:06	9	-0.16923	100.00% (10/10)	Non-Toxin	1.55	0.43	-0.96	1.98	1.02	9.2
544	polymerase	YQSTLGQSISF	HLA-B*15:01	11	-0.24531	100.00% (10/10)	Non-Toxin	1.44	1.14	-1.99	2.58	0.59	98
545	polymerase	YSNPDSTEVY	HLA-A*01:01	10	-0.0076	100.00% (10/10)	Non-Toxin	1.46	1.26	-1.72	2.72	1	52.3
546	polymerase	YSNPDSTEVY	HLA-B*15:01	10	-0.0076	100.00% (10/10)	Non-Toxin	1.46	1.26	-1.95	2.72	0.77	88.9
547	polymerase	YSNPDSTEVY	HLA-B*35:01	10	-0.0076	100.00% (10/10)	Non-Toxin	1.46	1.26	-2.16	2.72	0.56	145
548	polymerase	YTEVDNNHLIY	HLA-A*01:01	11	0.18189	100.00% (10/10)	Non-Toxin	1.3	1.12	-0.96	2.42	1.46	9.1
549	polymerase	YTPGFPISR	HLA-A*68:01	9	0.14972	100.00% (10/10)	Non-Toxin	1.08	0.69	-1	1.76	0.76	10.1
550	polymerase	YYQIDQPFF	HLA-A*23:01	9	0.06792	100.00% (10/10)	Non-Toxin	1.33	1.24	-1.43	2.57	1.14	26.8
551	polymerase	YYQIDQPFF	HLA-A*24:02	9	0.06792	100.00% (10/10)	Non-Toxin	1.33	1.24	-1.54	2.57	1.02	35
552	polymerase	YYQIDQPFFV	HLA-A*02:06	10	0.16188	100.00% (10/10)	Non-Toxin	1.07	0.27	-0.59	1.34	0.75	3.9
553	V protein	CLVSDAKMLSY	HLA-A*01:01	11	-0.57469	19.30% (11/57)	Non-Toxin	1.48	1.31	-1.82	2.8	0.98	65.8
554	V protein	DPVVTDVVY	HLA-B*35:01	9	0.17258	98.25% (56/57)	Non-Toxin	1.74	1.05	-1.4	2.8	1.4	25.1
555	V protein	GLNPTAVPF	HLA-B*15:01	9	0.08973	19.30% (11/57)	Non-Toxin	1.12	0.99	-1.4	2.12	0.72	24.9
556	V protein	KMLSYAPEI	HLA-A*02:01	9	-0.0877	19.30% (11/57)	Non-Toxin	1.24	0.4	-0.68	1.65	0.97	4.8
557	V protein	KMLSYAPEI	HLA-A*02:06	9	-0.0877	19.30% (11/57)	Non-Toxin	1.24	0.4	-0.82	1.65	0.83	6.6
558	V protein	KMLSYAPEI	HLA-A*32:01	9	-0.0877	19.30% (11/57)	Non-Toxin	1.24	0.4	-1.13	1.65	0.52	13.4
559	V protein	KSRGIPIKK	HLA-A*30:01	9	0.15638	100.00% (57/57)	Non-Toxin	1.05	0.22	-0.73	1.27	0.54	5.4
560	V protein	LDPVVTDVVY	HLA-B*35:01	10	0.19578	98.25% (56/57)	Non-Toxin	1.74	1.11	-1.34	2.85	1.51	22.1
561	V protein	LVSDAKMLSY	HLA-A*01:01	10	-0.4746	19.30% (11/57)	Non-Toxin	1.48	1.33	-1.26	2.81	1.55	18
562	V protein	LVSDAKMLSY	HLA-B*15:01	10	-0.4746	19.30% (11/57)	Non-Toxin	1.48	1.33	-1.94	2.81	0.87	87.9
563	V protein	NPTAVPFTLR	HLA-A*68:01	10	0.21785	19.30% (11/57)	Non-Toxin	1.01	0.53	-1.03	1.54	0.51	10.7
564	V protein	SPVIAEHYY	HLA-B*35:01	9	0.30481	98.25% (56/57)	Non-Toxin	1.36	1.13	-1.31	2.5	1.18	20.6
565	V protein	VSDAKMLSY	HLA-A*01:01	9	-0.43475	19.30% (11/57)	Non-Toxin	1.48	1.26	-0.94	2.74	1.8	8.7
566	W protein	CLVSDAKMLSY	HLA-A*01:01	11	-0.57469	20.51% (8/39)	Non-Toxin	1.48	1.31	-1.82	2.8	0.98	65.8
567	W protein	DPVVTDVVY	HLA-B*35:01	9	0.17258	97.44% (38/39)	Non-Toxin	1.74	1.05	-1.4	2.8	1.4	25.1
568	W protein	GLNPTAVPF	HLA-B*15:01	9	0.08973	20.51% (8/39)	Non-Toxin	1.12	0.99	-1.4	2.12	0.72	24.9
569	W protein	KMLSYAPEI	HLA-A*02:01	9	-0.0877	20.51% (8/39)	Non-Toxin	1.24	0.4	-0.68	1.65	0.97	4.8
570	W protein	KMLSYAPEI	HLA-A*02:06	9	-0.0877	20.51% (8/39)	Non-Toxin	1.24	0.4	-0.82	1.65	0.83	6.6
571	W protein	KMLSYAPEI	HLA-A*32:01	9	-0.0877	20.51% (8/39)	Non-Toxin	1.24	0.4	-1.13	1.65	0.52	13.4
572	W protein	KSRGIPIKK	HLA-A*30:01	9	0.15638	100.00% (39/39)	Non-Toxin	1.08	0.22	-0.73	1.31	0.57	5.4
573	W protein	LDPVVTDVVY	HLA-B*35:01	10	0.19578	97.44% (38/39)	Non-Toxin	1.74	1.11	-1.34	2.85	1.51	22.1
574	W protein	LVSDAKMLSY	HLA-A*01:01	10	-0.4746	20.51% (8/39)	Non-Toxin	1.48	1.33	-1.26	2.81	1.55	18
575	W protein	LVSDAKMLSY	HLA-B*15:01	10	-0.4746	20.51% (8/39)	Non-Toxin	1.48	1.33	-1.94	2.81	0.87	87.9
576	W protein	NPTAVPFTLR	HLA-A*68:01	10	0.21785	20.51% (8/39)	Non-Toxin	1.01	0.53	-1.03	1.54	0.51	10.7
577	W protein	QTRNIHLLGR	HLA-A*31:01	10	0.17139	97.44% (38/39)	Non-Toxin	1.11	0.73	-1.39	1.84	0.44	24.6
578	W protein	SPVIAEHYY	HLA-B*35:01	9	0.30481	100.00% (39/39)	Non-Toxin	1.36	1.13	-1.31	2.5	1.18	20.6
579	W protein	VSDAKMLSY	HLA-A*01:01	9	-0.43475	20.51% (8/39)	Non-Toxin	1.48	1.26	-0.94	2.74	1.8	8.7

Table S2: High Scoring CTL epitopes-HLA allele pairs screened from entire proteome of SARS-CoV-2 by the "MHC-I Binding Predictions" tool of IEDB. These epitopes were further utilized to identify the potentially immunogenic multiple epitope cluster based CTL Ag-Patches from the entire proteome of the SRAS-CoV-2. The IEDB recommended 2020.09 (NetMHCpan EL 4.1) method was used for this screening. The screened epitopes are in consensus with the previous studies[38].

#	Protein	Peptide	Allele	Length	Score	Percentile rank	Immunogenicity	Conservancy	Toxicity
1	C Protein	APVENLKKL	HLA-B*07:02	9	0.823222	0.07	-0.21059	1.67% (1/60)	Non-Toxin
2	C Protein	ASILLTLFR	HLA-A*31:01	9	0.728722	0.11	0.11682	100.00% (60/60)	Non-Toxin
3	C Protein	ASILLTLFR	HLA-A*11:01	9	0.665698	0.16	0.11682	100.00% (60/60)	Non-Toxin
4	C Protein	CPMLPKLQF	HLA-B*53:01	9	0.79741	0.04	-0.359	100.00% (60/60)	Non-Toxin
5	C Protein	CPMLPKLQF	HLA-B*35:01	9	0.787207	0.08	-0.359	100.00% (60/60)	Non-Toxin
6	C Protein	CPMLPKLQF	HLA-B*07:02	9	0.744382	0.11	-0.359	100.00% (60/60)	Non-Toxin
7	C Protein	EQTMGMYVLY	HLA-A*26:01	10	0.663978	0.06	-0.27722	100.00% (60/60)	Non-Toxin
8	C Protein	FLKKIGKL	HLA-B*08:01	8	0.710079	0.06	-0.3075	98.33% (59/60)	Non-Toxin
9	C Protein	GQKVQMTY	HLA-B*15:01	8	0.901547	0.02	-0.2738	95.00% (57/60)	Non-Toxin
10	C Protein	HQEQTMGMY	HLA-A*30:02	9	0.515293	0.16	-0.28556	100.00% (60/60)	Non-Toxin
11	C Protein	ITHQEQTMGMY	HLA-A*01:01	11	0.610291	0.14	-0.32286	100.00% (60/60)	Non-Toxin
12	C Protein	IYPVLLPQM	HLA-A*24:02	9	0.86784	0.04	-0.06034	100.00% (60/60)	Non-Toxin
13	C Protein	IYPVLLPQM	HLA-A*23:01	9	0.818931	0.04	-0.06034	100.00% (60/60)	Non-Toxin
14	C Protein	KLQFLKKIGK	HLA-A*03:01	10	0.719129	0.15	-0.21148	98.33% (59/60)	Non-Toxin
15	C Protein	KTEQKHGRTF	HLA-B*44:02	10	0.654226	0.12	-0.1643	26.67% (16/60)	Non-Toxin
16	C Protein	MASILLTLF	HLA-B*53:01	9	0.625818	0.07	0.08526	100.00% (60/60)	Non-Toxin
17	C Protein	MASILLTLF	HLA-B*58:01	9	0.816922	0.11	0.08526	100.00% (60/60)	Non-Toxin
18	C Protein	MEVLKEEAW	HLA-B*44:02	9	0.912517	0.02	-0.00615	78.33% (47/60)	Non-Toxin
19	C Protein	MEVLKEEAW	HLA-B*44:03	9	0.918867	0.03	-0.00615	78.33% (47/60)	Non-Toxin
20	C Protein	MMASILLTL	HLA-A*32:01	9	0.557134	0.07	-0.02129	100.00% (60/60)	Non-Toxin
21	C Protein	MMASILLTL	HLA-A*02:01	9	0.771922	0.09	-0.02129	100.00% (60/60)	Non-Toxin
22	C Protein	MMASILLTL	HLA-A*02:03	9	0.727441	0.09	-0.02129	100.00% (60/60)	Non-Toxin
23	C Protein	MTYNWTQW	HLA-B*57:01	8	0.913336	0.09	0.14677	95.00% (57/60)	Non-Toxin
24	C Protein	MTYNWTQW	HLA-B*58:01	8	0.808826	0.11	0.14677	95.00% (57/60)	Non-Toxin
25	C Protein	MTYNWTQWL	HLA-A*68:02	9	0.575361	0.14	0.27619	95.00% (57/60)	Non-Toxin
26	C Protein	MYVLYLMQR	HLA-A*33:01	9	0.734683	0.06	-0.22768	100.00% (60/60)	Non-Toxin
27	C Protein	QAFNNPASK	HLA-A*11:01	9	0.670372	0.16	-0.04889	73.33% (44/60)	Non-Toxin
28	C Protein	QAFNNPASK	HLA-A*30:01	9	0.476561	0.16	-0.04889	73.33% (44/60)	Non-Toxin
29	C Protein	QEQTMGMYVLY	HLA-B*44:03	11	0.693842	0.15	-0.28266	100.00% (60/60)	Non-Toxin
30	C Protein	QEQTMGMYVLY	HLA-B*44:02	11	0.57723	0.15	-0.28266	100.00% (60/60)	Non-Toxin
31	C Protein	QTMGMYVLY	HLA-A*26:01	9	0.775279	0.05	-0.16902	100.00% (60/60)	Non-Toxin
32	C Protein	QTMGMYVLY	HLA-A*30:02	9	0.715845	0.05	-0.16902	100.00% (60/60)	Non-Toxin
33	C Protein	QTMGMYVLY	HLA-A*32:01	9	0.43165	0.14	-0.16902	100.00% (60/60)	Non-Toxin
34	C Protein	QTMGMYVLY	HLA-A*01:01	9	0.576389	0.15	-0.16902	100.00% (60/60)	Non-Toxin
35	C Protein	RCCPMLPKLQF	HLA-B*07:02	11	0.814723	0.08	-0.5013	100.00% (60/60)	Non-Toxin
36	C Protein	RMMEVLKEEAW	HLA-A*32:01	11	0.668454	0.05	0.06476	78.33% (47/60)	Non-Toxin
37	C Protein	SILLTLFRR	HLA-A*31:01	9	0.676568	0.15	0.14164	100.00% (60/60)	Non-Toxin
38	C Protein	SKTEQKHGRTF	HLA-B*44:02	11	0.777859	0.06	-0.07969	26.67% (16/60)	Non-Toxin
39	C Protein	SKTEQKHGRTF	HLA-B*44:03	11	0.701106	0.14	-0.07969	26.67% (16/60)	Non-Toxin
40	C Protein	TEQKHGRTF	HLA-B*44:03	9	0.96597	0.01	-0.12484	26.67% (16/60)	Non-Toxin
41	C Protein	TEQKHGRTF	HLA-B*44:02	9	0.965601	0.01	-0.12484	26.67% (16/60)	Non-Toxin
42	C Protein	TGQKVQMTY	HLA-A*30:02	9	0.582272	0.11	-0.44896	95.00% (57/60)	Non-Toxin

#	Protein	Peptide	Allele	Length	Score	Percentile rank	Immunogenicity	Conservancy	Toxicity
43	C Protein	TLFRRTKKK	HLA-A*03:01	9	0.891743	0.03	-0.13098	100.00% (60/60)	Non-Toxin
44	C Protein	VLLPQMELL	HLA-A*02:01	9	0.870151	0.05	-0.21484	100.00% (60/60)	Non-Toxin
45	C Protein	VLLPQMELL	HLA-A*02:06	9	0.742716	0.09	-0.21484	100.00% (60/60)	Non-Toxin
46	C Protein	VPDMDLLQAL	HLA-B*07:02	10	0.69013	0.13	-0.24404	100.00% (60/60)	Non-Toxin
47	C Protein	VTGQKVQMTY	HLA-A*30:02	10	0.509179	0.16	-0.50992	95.00% (57/60)	Non-Toxin
48	C Protein	WTQWLQTLY	HLA-A*01:01	9	0.771517	0.07	0.09173	100.00% (60/60)	Non-Toxin
49	C Protein	YPVLLPQM	HLA-B*51:01	8	0.69854	0.07	-0.11676	100.00% (60/60)	Non-Toxin
50	C Protein	YPVLLPQM	HLA-B*35:01	8	0.717162	0.12	-0.11676	100.00% (60/60)	Non-Toxin
51	Glycoprotein	AENPVFTVF	HLA-B*44:03	9	0.9927	0.01	0.19402	100.00% (66/66)	Non-Toxin
52	Glycoprotein	AENPVFTVF	HLA-B*44:02	9	0.985092	0.01	0.19402	100.00% (66/66)	Non-Toxin
53	Glycoprotein	AENPVFTVF	HLA-B*40:01	9	0.961615	0.02	0.19402	100.00% (66/66)	Non-Toxin
54	Glycoprotein	ALQSIQQQI	HLA-A*02:03	9	0.75991	0.07	-0.34895	75.76% (50/66)	Non-Toxin
55	Glycoprotein	ALQSIQQQI	HLA-A*02:01	9	0.719158	0.12	-0.34895	75.76% (50/66)	Non-Toxin
56	Glycoprotein	AMDEGYFAY	HLA-A*01:01	9	0.938501	0.02	0.25913	90.91% (60/66)	Non-Toxin
57	Glycoprotein	AMDEGYFAY	HLA-A*30:02	9	0.709552	0.05	0.25913	90.91% (60/66)	Non-Toxin
58	Glycoprotein	AMIKDALQSI	HLA-A*02:03	10	0.69459	0.1	-0.31896	71.21% (47/66)	Non-Toxin
59	Glycoprotein	AQKTITNCF	HLA-B*15:01	9	0.969264	0.01	0.09824	100.00% (66/66)	Non-Toxin
60	Glycoprotein	ASDKGKNPSK	HLA-A*30:01	10	0.58442	0.09	-0.49891	75.76% (50/66)	Non-Toxin
61	Glycoprotein	ASFSWDTMIK	HLA-A*11:01	10	0.809104	0.07	0.07493	100.00% (66/66)	Non-Toxin
62	Glycoprotein	ASINENVNEK	HLA-A*11:01	10	0.759701	0.1	0.21979	100.00% (66/66)	Non-Toxin
63	Glycoprotein	AVKPKNNGESY	HLA-B*15:01	11	0.821766	0.05	-0.28402	71.21% (47/66)	Non-Toxin
64	Glycoprotein	AVKPKNNGESY	HLA-A*30:02	11	0.566904	0.12	-0.28402	71.21% (47/66)	Non-Toxin
65	Glycoprotein	AVYNNEFYY	HLA-A*30:02	9	0.891164	0.01	0.17688	90.91% (60/66)	Non-Toxin
66	Glycoprotein	AVYNNEFYY	HLA-A*11:01	9	0.880649	0.03	0.17688	90.91% (60/66)	Non-Toxin
67	Glycoprotein	CPNPLPFREY	HLA-B*53:01	10	0.52569	0.12	0.17752	100.00% (66/66)	Non-Toxin
68	Glycoprotein	DAFLIDRI	HLA-B*51:01	8	0.928331	0.01	0.221	100.00% (66/66)	Non-Toxin
69	Glycoprotein	DPVLNSTYW	HLA-B*53:01	9	0.922322	0.02	-0.12919	1.52% (1/66)	Non-Toxin
70	Glycoprotein	DSLGQPVFY	HLA-A*26:01	9	0.537647	0.1	0.0105	100.00% (66/66)	Non-Toxin
71	Glycoprotein	DVQTVNPLV	HLA-A*68:02	9	0.604285	0.12	0.01973	78.79% (52/66)	Non-Toxin
72	Glycoprotein	EENSKIVFI	HLA-B*44:02	9	0.814634	0.05	-0.15005	66.67% (44/66)	Non-Toxin
73	Glycoprotein	EENSKIVFI	HLA-B*44:03	9	0.806918	0.08	-0.15005	66.67% (44/66)	Non-Toxin
74	Glycoprotein	EIGPKVSL	HLA-B*08:01	8	0.686032	0.07	-0.31092	100.00% (66/66)	Non-Toxin
75	Glycoprotein	EISDQRLSI	HLA-A*68:02	9	0.605462	0.12	-0.20148	98.48% (65/66)	Non-Toxin
76	Glycoprotein	EIYDTGDNV	HLA-A*68:02	9	0.818284	0.05	0.10576	100.00% (66/66)	Non-Toxin
77	Glycoprotein	EIYDTGDNVIR	HLA-A*68:01	11	0.922043	0.06	0.22003	100.00% (66/66)	Non-Toxin
78	Glycoprotein	EVLDRGDEV	HLA-A*68:02	9	0.653933	0.1	0.17824	100.00% (66/66)	Non-Toxin
79	Glycoprotein	EVPSLFMTNV	HLA-A*68:02	10	0.673201	0.09	-0.20319	100.00% (66/66)	Non-Toxin
80	Glycoprotein	FAYSHLEKI	HLA-B*51:01	9	0.875973	0.02	-0.18811	74.24% (49/66)	Non-Toxin
81	Glycoprotein	FAYSHLEKI	HLA-A*02:06	9	0.778243	0.08	-0.18811	74.24% (49/66)	Non-Toxin
82	Glycoprotein	FLIDRINWI	HLA-A*02:03	9	0.955217	0.02	0.36516	100.00% (66/66)	Non-Toxin
83	Glycoprotein	FLIDRINWI	HLA-A*02:01	9	0.926293	0.03	0.36516	100.00% (66/66)	Non-Toxin
84	Glycoprotein	FLIDRINWI	HLA-A*02:06	9	0.829268	0.07	0.36516	100.00% (66/66)	Non-Toxin
85	Glycoprotein	FLIDRINWISA	HLA-A*02:03	11	0.833318	0.05	0.46339	100.00% (66/66)	Non-Toxin
86	Glycoprotein	FLIDRINWISA	HLA-A*02:01	11	0.779718	0.09	0.46339	100.00% (66/66)	Non-Toxin
87	Glycoprotein	FPAVGFLV	HLA-B*51:01	8	0.831141	0.03	0.18808	100.00% (66/66)	Non-Toxin
88	Glycoprotein	FSWDTMIKF	HLA-B*58:01	9	0.824772	0.1	-0.04696	100.00% (66/66)	Non-Toxin
89	Glycoprotein	GESYNQHQF	HLA-B*44:02	9	0.933642	0.02	-0.21314	75.76% (50/66)	Non-Toxin
90	Glycoprotein	GESYNQHQF	HLA-B*44:03	9	0.94744	0.03	-0.21314	75.76% (50/66)	Non-Toxin
91	Glycoprotein	GESYNQHQF	HLA-B*40:01	9	0.864791	0.08	-0.21314	75.76% (50/66)	Non-Toxin

#	Protein	Peptide	Allele	Length	Score	Percentile rank	Immunogenicity	Conservancy	Toxicity
92	Glycoprotein	GIKQGDTLY	HLA-B*15:01	9	0.892776	0.03	-0.1064	100.00% (66/66)	Non-Toxin
93	Glycoprotein	GLADKIGTEI	HLA-A*02:03	10	0.861059	0.04	0.07778	100.00% (66/66)	Non-Toxin
94	Glycoprotein	GLLDSKILSA	HLA-A*02:01	10	0.735282	0.11	-0.33312	98.48% (65/66)	Non-Toxin
95	Glycoprotein	GLLDSKILSA	HLA-A*02:03	10	0.65261	0.11	-0.33312	98.48% (65/66)	Non-Toxin
96	Glycoprotein	GTEIGPKVSL	HLA-B*40:01	10	0.970847	0.02	-0.0762	100.00% (66/66)	Non-Toxin
97	Glycoprotein	IGTEIGPKVSL	HLA-B*40:01	11	0.842277	0.09	0.00033	100.00% (66/66)	Non-Toxin
98	Glycoprotein	ILKPKLISY	HLA-B*15:01	9	0.974856	0.01	-0.28594	96.97% (64/66)	Non-Toxin
99	Glycoprotein	ILKPKLISY	HLA-A*30:02	9	0.7787	0.03	-0.28594	96.97% (64/66)	Non-Toxin
100	Glycoprotein	ILKPKLISY	HLA-A*32:01	9	0.613876	0.06	-0.28594	96.97% (64/66)	Non-Toxin
101	Glycoprotein	ILKPKLISYTL	HLA-B*08:01	11	0.565422	0.11	-0.30853	96.97% (64/66)	Non-Toxin
102	Glycoprotein	ILRSGLLKY	HLA-B*15:01	9	0.915685	0.02	-0.26247	98.48% (65/66)	Non-Toxin
103	Glycoprotein	ILRSGLLKY	HLA-A*30:02	9	0.735218	0.04	-0.26247	98.48% (65/66)	Non-Toxin
104	Glycoprotein	IYDSLGQPVF	HLA-A*24:02	10	0.835396	0.05	-0.23135	96.97% (64/66)	Non-Toxin
105	Glycoprotein	IYDSLGQPVF	HLA-A*23:01	10	0.736497	0.07	-0.23135	96.97% (64/66)	Non-Toxin
106	Glycoprotein	KGKNPSKVIK	HLA-A*30:01	10	0.583524	0.09	-0.33881	80.30% (53/66)	Non-Toxin
107	Glycoprotein	KILSAFNTV	HLA-A*02:06	9	0.844148	0.06	-0.00455	98.48% (65/66)	Non-Toxin
108	Glycoprotein	KINEGLLDSK	HLA-A*03:01	10	0.944226	0.02	0.03247	98.48% (65/66)	Non-Toxin
109	Glycoprotein	KINEGLLDSK	HLA-A*11:01	10	0.736511	0.11	0.03247	98.48% (65/66)	Non-Toxin
110	Glycoprotein	KIYDSLGQPV	HLA-A*02:03	10	0.726573	0.09	-0.22312	96.97% (64/66)	Non-Toxin
111	Glycoprotein	KIYDSLGQPVF	HLA-A*32:01	11	0.530682	0.09	-0.21206	96.97% (64/66)	Non-Toxin
112	Glycoprotein	KLISYTLPV	HLA-A*02:01	9	0.871446	0.05	-0.10617	95.45% (63/66)	Non-Toxin
113	Glycoprotein	KLISYTLPV	HLA-A*02:03	9	0.830418	0.05	-0.10617	95.45% (63/66)	Non-Toxin
114	Glycoprotein	KLISYTLPV	HLA-A*02:06	9	0.757053	0.09	-0.10617	95.45% (63/66)	Non-Toxin
115	Glycoprotein	KPENCRLSM	HLA-B*07:02	9	0.924779	0.04	-0.08381	98.48% (65/66)	Non-Toxin
116	Glycoprotein	KPKLISYTL	HLA-B*07:02	9	0.981271	0.01	-0.08773	96.97% (64/66)	Non-Toxin
117	Glycoprotein	KPKLISYTL	HLA-B*08:01	9	0.794497	0.04	-0.08773	96.97% (64/66)	Non-Toxin
118	Glycoprotein	KPKNNGESY	HLA-B*35:01	9	0.716665	0.12	-0.06307	71.21% (47/66)	Non-Toxin
119	Glycoprotein	KPQTEGVSNL	HLA-B*07:02	10	0.867951	0.05	0.02742	75.76% (50/66)	Non-Toxin
120	Glycoprotein	KSYYGTMDIK	HLA-A*03:01	10	0.829379	0.06	-0.00294	100.00% (66/66)	Non-Toxin
121	Glycoprotein	KSYYGTMDIKK	HLA-A*03:01	11	0.871061	0.04	-0.09792	100.00% (66/66)	Non-Toxin
122	Glycoprotein	KTITNCFLLK	HLA-A*11:01	10	0.736348	0.11	0.11782	98.48% (65/66)	Non-Toxin
123	Glycoprotein	KTSNQILKPK	HLA-A*03:01	10	0.876597	0.04	-0.24233	98.48% (65/66)	Non-Toxin
124	Glycoprotein	KTSNQILKPK	HLA-A*11:01	10	0.839489	0.06	-0.24233	98.48% (65/66)	Non-Toxin
125	Glycoprotein	KVMPYGPSGIK	HLA-A*03:01	11	0.94341	0.02	-0.09893	100.00% (66/66)	Non-Toxin
126	Glycoprotein	KVMPYGPSGIK	HLA-A*30:01	11	0.706511	0.04	-0.09893	100.00% (66/66)	Non-Toxin
127	Glycoprotein	KVMPYGPSGIK	HLA-A*11:01	11	0.79294	0.08	-0.09893	100.00% (66/66)	Non-Toxin
128	Glycoprotein	LAMDEGYFAY	HLA-A*01:01	10	0.829827	0.06	0.214	90.91% (60/66)	Non-Toxin
129	Glycoprotein	LAMDEGYFAY	HLA-B*35:01	10	0.778031	0.09	0.214	90.91% (60/66)	Non-Toxin
130	Glycoprotein	LLDSKILSA	HLA-A*02:01	9	0.802868	0.08	-0.35001	98.48% (65/66)	Non-Toxin
131	Glycoprotein	LLKNKIWCI	HLA-B*08:01	9	0.645816	0.08	-0.00579	98.48% (65/66)	Non-Toxin
132	Glycoprotein	LYFPAVGFL	HLA-A*23:01	9	0.673981	0.09	0.2008	100.00% (66/66)	Non-Toxin
133	Glycoprotein	LYFPAVGFL	HLA-A*24:02	9	0.670528	0.12	0.2008	100.00% (66/66)	Non-Toxin
134	Glycoprotein	MIKDALQSI	HLA-A*02:03	9	0.712158	0.1	-0.21444	71.21% (47/66)	Non-Toxin
135	Glycoprotein	MPTESKKV	HLA-B*51:01	8	0.612667	0.12	-0.43275	74.24% (49/66)	Non-Toxin
136	Glycoprotein	MPTESKKVRF	HLA-B*53:01	10	0.651627	0.07	-0.39567	74.24% (49/66)	Non-Toxin
137	Glycoprotein	MPYGPSGI	HLA-B*51:01	8	0.838764	0.03	-0.10503	100.00% (66/66)	Non-Toxin
138	Glycoprotein	MTRLAVKPK	HLA-A*30:01	9	0.74319	0.03	-0.10588	100.00% (66/66)	Non-Toxin
139	Glycoprotein	NPSKVIKSY	HLA-B*35:01	9	0.974763	0.01	-0.38388	80.30% (53/66)	Non-Toxin
140	Glycoprotein	NPSKVIKSY	HLA-B*53:01	9	0.754206	0.05	-0.38388	80.30% (53/66)	Non-Toxin

#	Protein	Peptide	Allele	Length	Score	Percentile rank	Immunogenicity	Conservancy	Toxicity
141	Glycoprotein	NQILKPKLISY	HLA-B*15:01	11	0.731875	0.12	-0.39354	96.97% (64/66)	Non-Toxin
142	Glycoprotein	QPVFYQASF	HLA-B*35:01	9	0.874428	0.05	-0.04508	100.00% (66/66)	Non-Toxin
143	Glycoprotein	QPVFYQASF	HLA-B*53:01	9	0.567699	0.09	-0.04508	100.00% (66/66)	Non-Toxin
144	Glycoprotein	QPVFYQASFSW	HLA-B*53:01	11	0.745175	0.05	-0.10069	100.00% (66/66)	Non-Toxin
145	Glycoprotein	QTAENPVFTV	HLA-A*68:02	10	0.896524	0.02	0.25669	100.00% (66/66)	Non-Toxin
146	Glycoprotein	QTVNPLVVNW	HLA-B*57:01	10	0.989017	0.01	0.05521	72.73% (48/66)	Non-Toxin
147	Glycoprotein	QTVNPLVVNW	HLA-B*58:01	10	0.940343	0.04	0.05521	72.73% (48/66)	Non-Toxin
148	Glycoprotein	RLSIGSPSK	HLA-A*03:01	9	0.924551	0.02	-0.14853	98.48% (65/66)	Non-Toxin
149	Glycoprotein	RLSIGSPSK	HLA-A*30:01	9	0.611386	0.07	-0.14853	98.48% (65/66)	Non-Toxin
150	Glycoprotein	RPKLFAVKI	HLA-B*07:02	9	0.817556	0.07	-0.02149	100.00% (66/66)	Non-Toxin
151	Glycoprotein	RPNSHYIL	HLA-B*07:02	8	0.865362	0.06	-0.02823	95.45% (63/66)	Non-Toxin
152	Glycoprotein	SAVYNNEFYY	HLA-A*30:02	10	0.571065	0.11	0.18797	90.91% (60/66)	Non-Toxin
153	Glycoprotein	SIGSPSKIY	HLA-A*30:02	9	0.638341	0.08	-0.42624	95.45% (63/66)	Non-Toxin
154	Glycoprotein	SINENVNEK	HLA-A*11:01	9	0.852696	0.05	0.18425	100.00% (66/66)	Non-Toxin
155	Glycoprotein	SLIDTSSTI	HLA-A*02:03	9	0.85892	0.04	-0.16935	100.00% (66/66)	Non-Toxin
156	Glycoprotein	SLIDTSSTI	HLA-A*02:01	9	0.724287	0.12	-0.16935	100.00% (66/66)	Non-Toxin
157	Glycoprotein	SMGIRPNSHY	HLA-B*15:01	10	0.75637	0.1	0.05771	96.97% (64/66)	Non-Toxin
158	Glycoprotein	SPSKIYDSL	HLA-B*07:02	9	0.936711	0.04	-0.22252	95.45% (63/66)	Non-Toxin
159	Glycoprotein	STASINENV	HLA-A*68:02	9	0.95749	0.01	0.05046	100.00% (66/66)	Non-Toxin
160	Glycoprotein	STDNQAMIK	HLA-A*11:01	9	0.798004	0.08	-0.14572	71.21% (47/66)	Non-Toxin
161	Glycoprotein	STITIPANI	HLA-A*68:02	9	0.852249	0.04	0.23066	100.00% (66/66)	Non-Toxin
162	Glycoprotein	SYNQHQFAL	HLA-A*24:02	9	0.75696	0.07	-0.07454	75.76% (50/66)	Non-Toxin
163	Glycoprotein	SYNQHQFAL	HLA-A*23:01	9	0.602283	0.11	-0.07454	75.76% (50/66)	Non-Toxin
164	Glycoprotein	TEIGPKVSL	HLA-B*40:01	9	0.993634	0.01	-0.19832	100.00% (66/66)	Non-Toxin
165	Glycoprotein	TEIGPKVSL	HLA-B*44:03	9	0.881783	0.04	-0.19832	100.00% (66/66)	Non-Toxin
166	Glycoprotein	TEIGPKVSL	HLA-B*44:02	9	0.809345	0.05	-0.19832	100.00% (66/66)	Non-Toxin
167	Glycoprotein	TPSNPNTVY	HLA-B*35:01	9	0.979377	0.01	-0.02022	75.76% (50/66)	Non-Toxin
168	Glycoprotein	TPSNPNTVY	HLA-B*53:01	9	0.663863	0.07	-0.02022	75.76% (50/66)	Non-Toxin
169	Glycoprotein	TSNQILKPK	HLA-A*11:01	9	0.771223	0.09	-0.18798	98.48% (65/66)	Non-Toxin
170	Glycoprotein	TSNQILKPK	HLA-A*30:01	9	0.530047	0.11	-0.18798	98.48% (65/66)	Non-Toxin
171	Glycoprotein	TVFKDNEVLY	HLA-A*26:01	10	0.631707	0.07	-0.04109	75.76% (50/66)	Non-Toxin
172	Glycoprotein	TVFKDNEVLY	HLA-A*30:02	10	0.58975	0.11	-0.04109	75.76% (50/66)	Non-Toxin
173	Glycoprotein	TVFKDNEVLYR	HLA-A*68:01	11	0.883557	0.09	-0.03886	75.76% (50/66)	Non-Toxin
174	Glycoprotein	TVNPLVVNW	HLA-B*57:01	9	0.99016	0.01	0.04586	92.42% (61/66)	Non-Toxin
175	Glycoprotein	TVNPLVVNW	HLA-B*58:01	9	0.98245	0.01	0.04586	92.42% (61/66)	Non-Toxin
176	Glycoprotein	TVNPLVVNW	HLA-A*32:01	9	0.882614	0.01	0.04586	92.42% (61/66)	Non-Toxin
177	Glycoprotein	TVNPLVVNW	HLA-B*53:01	9	0.853695	0.03	0.04586	92.42% (61/66)	Non-Toxin
178	Glycoprotein	TVNPLVVNWR	HLA-A*68:01	10	0.9277	0.05	0.17896	92.42% (61/66)	Non-Toxin
179	Glycoprotein	TVYHCSAVY	HLA-A*30:02	9	0.576146	0.11	-0.11974	100.00% (66/66)	Non-Toxin
180	Glycoprotein	TVYHCSAVY	HLA-A*26:01	9	0.502916	0.11	-0.11974	100.00% (66/66)	Non-Toxin
181	Glycoprotein	TYWSGSLMM	HLA-A*23:01	9	0.630795	0.1	-0.32926	100.00% (66/66)	Non-Toxin
182	Glycoprotein	TYWSGSLMMTR	HLA-A*33:01	11	0.584673	0.11	-0.52429	100.00% (66/66)	Non-Toxin
183	Glycoprotein	VFKDNEVLY	HLA-A*30:02	9	0.688437	0.06	0.06863	75.76% (50/66)	Non-Toxin
184	Glycoprotein	VFYQASFSW	HLA-A*23:01	9	0.89986	0.02	-0.23325	100.00% (66/66)	Non-Toxin
185	Glycoprotein	VFYQASFSW	HLA-A*24:02	9	0.84855	0.04	-0.23325	100.00% (66/66)	Non-Toxin
186	Glycoprotein	VFYQASFSW	HLA-A*32:01	9	0.489801	0.11	-0.23325	100.00% (66/66)	Non-Toxin
187	Glycoprotein	VIRPKLFAV	HLA-B*08:01	9	0.764439	0.05	-0.09314	100.00% (66/66)	Non-Toxin
188	Glycoprotein	VIRPKLFAV	HLA-A*02:03	9	0.692449	0.1	-0.09314	100.00% (66/66)	Non-Toxin
189	Glycoprotein	VPSLFMTNV	HLA-B*51:01	9	0.820342	0.03	-0.08718	100.00% (66/66)	Non-Toxin

#	Protein	Peptide	Allele	Length	Score	Percentile rank	Immunogenicity	Conservancy	Toxicity
190	Glycoprotein	VPSLFMTNVW	HLA-B*53:01	10	0.730907	0.05	-0.06666	100.00% (66/66)	Non-Toxin
191	Glycoprotein	VWTPSNPNTVY	HLA-B*35:01	11	0.709721	0.12	-0.12597	75.76% (50/66)	Non-Toxin
192	Glycoprotein	VYNNEFYYV	HLA-A*24:02	9	0.773742	0.06	0.19381	90.91% (60/66)	Non-Toxin
193	Glycoprotein	VYNNEFYYV	HLA-A*23:01	9	0.644897	0.1	0.19381	90.91% (60/66)	Non-Toxin
194	Glycoprotein	WTPSNPNTVY	HLA-B*35:01	10	0.774703	0.09	-0.13638	75.76% (50/66)	Non-Toxin
195	Glycoprotein	WTPSNPNTVY	HLA-A*01:01	10	0.637408	0.12	-0.13638	75.76% (50/66)	Non-Toxin
196	Glycoprotein	YILRSGLLK	HLA-A*03:01	9	0.759821	0.12	-0.09656	98.48% (65/66)	Non-Toxin
197	Matrix Protein	AAYPLGVGK	HLA-A*11:01	9	0.958713	0.01	0.06338	97.44% (38/39)	Non-Toxin
198	Matrix Protein	AAYPLGVGK	HLA-A*03:01	9	0.903419	0.03	0.06338	97.44% (38/39)	Non-Toxin
199	Matrix Protein	AAYPLGVGK	HLA-A*30:01	9	0.724233	0.04	0.06338	97.44% (38/39)	Non-Toxin
200	Matrix Protein	AVLQPSIPR	HLA-A*11:01	9	0.937807	0.01	-0.18085	38.46% (15/39)	Non-Toxin
201	Matrix Protein	AVLQPSIPR	HLA-A*31:01	9	0.877796	0.04	-0.18085	38.46% (15/39)	Non-Toxin
202	Matrix Protein	DENGSMIPKY	HLA-B*44:03	10	0.869933	0.05	-0.31018	97.44% (38/39)	Non-Toxin
203	Matrix Protein	DENGSMIPKY	HLA-B*44:02	10	0.705537	0.1	-0.31018	97.44% (38/39)	Non-Toxin
204	Matrix Protein	DVFIDNTGR	HLA-A*68:01	9	0.944623	0.04	0.23999	94.87% (37/39)	Non-Toxin
205	Matrix Protein	DVFIDNTGR	HLA-A*33:01	9	0.566445	0.12	0.23999	94.87% (37/39)	Non-Toxin
206	Matrix Protein	ESMEGVSDF	HLA-A*26:01	9	0.750935	0.05	-0.01105	97.44% (38/39)	Non-Toxin
207	Matrix Protein	FSLMDINPW	HLA-B*58:01	9	0.862849	0.08	-0.04536	94.87% (37/39)	Non-Toxin
208	Matrix Protein	GPLNHLVPW	HLA-B*53:01	9	0.536302	0.11	0.03931	97.44% (38/39)	Non-Toxin
209	Matrix Protein	HPQDLLEEL	HLA-B*35:01	9	0.910201	0.04	0.10648	97.44% (38/39)	Non-Toxin
210	Matrix Protein	HPQDLLEEL	HLA-B*53:01	9	0.770931	0.04	0.10648	97.44% (38/39)	Non-Toxin
211	Matrix Protein	HPQDLLEEL	HLA-B*07:02	9	0.747457	0.11	0.10648	97.44% (38/39)	Non-Toxin
212	Matrix Protein	IFNAVKVCR	HLA-A*33:01	9	0.719007	0.06	-0.12219	97.44% (38/39)	Non-Toxin
213	Matrix Protein	IFNAVKVCR	HLA-A*31:01	9	0.749649	0.09	-0.12219	97.44% (38/39)	Non-Toxin
214	Matrix Protein	INPWLNRLTW	HLA-B*53:01	10	0.540939	0.11	0.26423	94.87% (37/39)	Non-Toxin
215	Matrix Protein	IQLDKHQAL	HLA-B*08:01	9	0.770985	0.04	-0.23573	97.44% (38/39)	Non-Toxin
216	Matrix Protein	IQLDKHQAL	HLA-A*02:06	9	0.72365	0.1	-0.23573	97.44% (38/39)	Non-Toxin
217	Matrix Protein	IYMIPRTML	HLA-A*24:02	9	0.77957	0.06	0.045	97.44% (38/39)	Non-Toxin
218	Matrix Protein	IYMIPRTML	HLA-A*23:01	9	0.702654	0.08	0.045	97.44% (38/39)	Non-Toxin
219	Matrix Protein	IYMIPRTMLEF	HLA-A*24:02	11	0.779077	0.06	0.03816	97.44% (38/39)	Non-Toxin
220	Matrix Protein	IYMIPRTMLEF	HLA-A*23:01	11	0.734098	0.07	0.03816	97.44% (38/39)	Non-Toxin
221	Matrix Protein	KIDRMKLQF	HLA-A*32:01	9	0.565259	0.07	-0.39176	94.87% (37/39)	Non-Toxin
222	Matrix Protein	KINGVISKR	HLA-A*31:01	9	0.95937	0.01	-0.06814	94.87% (37/39)	Non-Toxin
223	Matrix Protein	KINGVISKR	HLA-A*03:01	9	0.828358	0.06	-0.06814	94.87% (37/39)	Non-Toxin
224	Matrix Protein	KIYTPGANER	HLA-A*31:01	10	0.798054	0.06	0.14993	97.44% (38/39)	Non-Toxin
225	Matrix Protein	KIYTPGANER	HLA-A*03:01	10	0.82045	0.07	0.14993	97.44% (38/39)	Non-Toxin
226	Matrix Protein	KIYTPGANERK	HLA-A*03:01	11	0.960329	0.01	0.20681	97.44% (38/39)	Non-Toxin
227	Matrix Protein	KLNDSGIYM	HLA-A*02:01	9	0.766353	0.09	0.00118	97.44% (38/39)	Non-Toxin
228	Matrix Protein	KLNDSGIYM	HLA-A*02:03	9	0.659643	0.11	0.00118	97.44% (38/39)	Non-Toxin
229	Matrix Protein	KVASFMLHL	HLA-A*32:01	9	0.757709	0.02	-0.19553	79.49% (31/39)	Non-Toxin
230	Matrix Protein	KVASFMLHL	HLA-A*02:06	9	0.802942	0.07	-0.19553	79.49% (31/39)	Non-Toxin
231	Matrix Protein	KYNNYMYLI	HLA-A*24:02	9	0.902303	0.02	-0.18711	97.44% (38/39)	Non-Toxin
232	Matrix Protein	KYNNYMYLI	HLA-A*23:01	9	0.8084	0.05	-0.18711	97.44% (38/39)	Non-Toxin
233	Matrix Protein	KYYSVDYCR	HLA-A*31:01	9	0.860264	0.04	-0.14121	97.44% (38/39)	Non-Toxin
234	Matrix Protein	KYYSVDYCRR	HLA-A*31:01	10	0.735617	0.1	-0.12461	97.44% (38/39)	Non-Toxin
235	Matrix Protein	LQPSIPREF	HLA-B*15:01	9	0.769308	0.08	0.05127	38.46% (15/39)	Non-Toxin
236	Matrix Protein	NAIAFNLLV	HLA-B*51:01	9	0.620195	0.12	0.17464	84.62% (33/39)	Non-Toxin
237	Matrix Protein	NPWLNRLTW	HLA-B*53:01	9	0.949646	0.01	0.11648	94.87% (37/39)	Non-Toxin
238	Matrix Protein	QLDKHQAL	HLA-B*08:01	8	0.588707	0.1	-0.25432	97.44% (38/39)	Non-Toxin

#	Protein	Peptide	Allele	Length	Score	Percentile rank	Immunogenicity	Conservancy	Toxicity
239	Matrix Protein	QPSIPREFM	HLA-B*07:02	9	0.861829	0.06	0.27104	38.46% (15/39)	Non-Toxin
240	Matrix Protein	RIFFLSITK	HLA-A*03:01	9	0.949438	0.02	0.12427	97.44% (38/39)	Non-Toxin
241	Matrix Protein	RIFFLSITK	HLA-A*11:01	9	0.88709	0.03	0.12427	97.44% (38/39)	Non-Toxin
242	Matrix Protein	RIFFLSITK	HLA-A*30:01	9	0.736092	0.04	0.12427	97.44% (38/39)	Non-Toxin
243	Matrix Protein	RLFAQMGFQK	HLA-A*03:01	10	0.946666	0.02	-0.14341	94.87% (37/39)	Non-Toxin
244	Matrix Protein	SESMEGVSDF	HLA-B*44:02	10	0.78351	0.06	-0.1877	97.44% (38/39)	Non-Toxin
245	Matrix Protein	SESMEGVSDF	HLA-B*44:03	10	0.816639	0.07	-0.1877	97.44% (38/39)	Non-Toxin
246	Matrix Protein	SLDKDGFKV	HLA-A*02:01	9	0.823165	0.07	-0.1835	82.05% (32/39)	Non-Toxin
247	Matrix Protein	SLHIKINGV	HLA-A*02:03	9	0.913363	0.03	0.07404	94.87% (37/39)	Non-Toxin
248	Matrix Protein	SLMDINPWL	HLA-A*02:01	9	0.967916	0.02	0.20889	94.87% (37/39)	Non-Toxin
249	Matrix Protein	SLMDINPWL	HLA-A*02:06	9	0.917932	0.03	0.20889	94.87% (37/39)	Non-Toxin
250	Matrix Protein	SLMDINPWL	HLA-A*02:03	9	0.778072	0.06	0.20889	94.87% (37/39)	Non-Toxin
251	Matrix Protein	SMIPKYKIY	HLA-B*15:01	9	0.884702	0.03	-0.28568	97.44% (38/39)	Non-Toxin
252	Matrix Protein	SMIPKYKIY	HLA-A*30:02	9	0.732656	0.04	-0.28568	97.44% (38/39)	Non-Toxin
253	Matrix Protein	SPSSWEHGGYL	HLA-B*07:02	11	0.719483	0.11	0.18287	43.59% (17/39)	Non-Toxin
254	Matrix Protein	SSGPLNHLVPW	HLA-B*57:01	11	0.983765	0.02	0.03216	97.44% (38/39)	Non-Toxin
255	Matrix Protein	SSGPLNHLVPW	HLA-B*58:01	11	0.964931	0.03	0.03216	97.44% (38/39)	Non-Toxin
256	Matrix Protein	VAAVLQPSI	HLA-B*51:01	9	0.761215	0.05	-0.17162	38.46% (15/39)	Non-Toxin
257	Matrix Protein	VSDFSPSSW	HLA-B*58:01	9	0.979107	0.01	-0.28282	97.44% (38/39)	Non-Toxin
258	Matrix Protein	VSDFSPSSW	HLA-B*57:01	9	0.94504	0.06	-0.28282	97.44% (38/39)	Non-Toxin
259	Matrix Protein	YLDKVEPEI	HLA-A*02:01	9	0.970643	0.02	-0.02621	97.44% (38/39)	Non-Toxin
260	Matrix Protein	YLDKVEPEI	HLA-A*02:06	9	0.901827	0.04	-0.02621	97.44% (38/39)	Non-Toxin
261	Matrix Protein	YMIPRTML	HLA-B*08:01	8	0.840889	0.03	-0.02922	97.44% (38/39)	Non-Toxin
262	Matrix Protein	YMIPRTMLEF	HLA-B*15:01	10	0.718913	0.12	0.00408	97.44% (38/39)	Non-Toxin
263	Nucleoprotein	AAFTLISMY	HLA-A*30:02	9	0.739162	0.04	-0.05068	100.00% (13/13)	Non-Toxin
264	Nucleoprotein	AAFTLISMY	HLA-B*35:01	9	0.710899	0.12	-0.05068	100.00% (13/13)	Non-Toxin
265	Nucleoprotein	AATATLTTK	HLA-A*11:01	9	0.746321	0.1	0.13477	100.00% (13/13)	Non-Toxin
266	Nucleoprotein	AEESETRRW	HLA-B*44:02	9	0.995607	0.01	0.07399	100.00% (13/13)	Non-Toxin
267	Nucleoprotein	AEESETRRW	HLA-B*44:03	9	0.995024	0.01	0.07399	100.00% (13/13)	Non-Toxin
268	Nucleoprotein	AESMKVGAAF	HLA-B*44:02	10	0.870437	0.03	-0.31242	100.00% (13/13)	Non-Toxin
269	Nucleoprotein	AESMKVGAAF	HLA-B*44:03	10	0.874851	0.05	-0.31242	100.00% (13/13)	Non-Toxin
270	Nucleoprotein	APGGYPLLW	HLA-B*53:01	9	0.84362	0.03	0.01522	100.00% (13/13)	Non-Toxin
271	Nucleoprotein	ASFRSYQSK	HLA-A*30:01	9	0.868071	0.01	-0.26892	100.00% (13/13)	Non-Toxin
272	Nucleoprotein	ASFRSYQSK	HLA-A*11:01	9	0.913021	0.02	-0.26892	100.00% (13/13)	Non-Toxin
273	Nucleoprotein	ASFRSYQSK	HLA-A*03:01	9	0.837943	0.05	-0.26892	100.00% (13/13)	Non-Toxin
274	Nucleoprotein	ATDDPAISNK	HLA-A*11:01	10	0.910367	0.02	0.0387	84.62% (11/13)	Non-Toxin
275	Nucleoprotein	ATNSPELRW	HLA-B*57:01	9	0.989609	0.01	-0.06424	100.00% (13/13)	Non-Toxin
276	Nucleoprotein	ATNSPELRW	HLA-B*58:01	9	0.989266	0.01	-0.06424	100.00% (13/13)	Non-Toxin
277	Nucleoprotein	ATNSPELRW	HLA-A*32:01	9	0.742075	0.03	-0.06424	100.00% (13/13)	Non-Toxin
278	Nucleoprotein	DIFEEAASF	HLA-A*26:01	9	0.844817	0.03	0.20944	100.00% (13/13)	Non-Toxin
279	Nucleoprotein	DTAEESETR	HLA-A*68:01	9	0.938362	0.04	0.1624	100.00% (13/13)	Non-Toxin
280	Nucleoprotein	DTAEESETRR	HLA-A*68:01	10	0.898259	0.07	0.2071	100.00% (13/13)	Non-Toxin
281	Nucleoprotein	EEAASFRSY	HLA-B*44:03	9	0.995846	0.01	-0.05181	100.00% (13/13)	Non-Toxin
282	Nucleoprotein	EEAASFRSY	HLA-B*44:02	9	0.9927	0.01	-0.05181	100.00% (13/13)	Non-Toxin
283	Nucleoprotein	EEMEGLMRI	HLA-B*44:03	9	0.916195	0.03	-0.05165	100.00% (13/13)	Non-Toxin
284	Nucleoprotein	EEMEGLMRI	HLA-B*44:02	9	0.853212	0.03	-0.05165	100.00% (13/13)	Non-Toxin
285	Nucleoprotein	EEMEGLMRIL	HLA-B*44:03	10	0.731993	0.12	0.02209	100.00% (13/13)	Non-Toxin
286	Nucleoprotein	EESETRRW	HLA-B*44:02	8	0.849991	0.03	0.17725	100.00% (13/13)	Non-Toxin
287	Nucleoprotein	EESETRRW	HLA-B*44:03	8	0.859907	0.05	0.17725	100.00% (13/13)	Non-Toxin

#	Protein	Peptide	Allele	Length	Score	Percentile rank	Immunogenicity	Conservancy	Toxicity
288	Nucleoprotein	EESETRRWAKY	HLA-B*44:02	11	0.914295	0.02	0.30118	100.00% (13/13)	Non-Toxin
289	Nucleoprotein	EESETRRWAKY	HLA-B*44:03	11	0.925687	0.03	0.30118	100.00% (13/13)	Non-Toxin
290	Nucleoprotein	EIISDIGNY	HLA-A*26:01	9	0.990437	0.01	0.04843	100.00% (13/13)	Non-Toxin
291	Nucleoprotein	EIISDIGNYV	HLA-A*68:02	10	0.858595	0.04	0.05221	100.00% (13/13)	Non-Toxin
292	Nucleoprotein	ESMKVGAAF	HLA-A*26:01	9	0.767075	0.05	-0.14602	100.00% (13/13)	Non-Toxin
293	Nucleoprotein	FAMGVATTI	HLA-B*51:01	9	0.899183	0.02	0.10957	100.00% (13/13)	Non-Toxin
294	Nucleoprotein	FAPGGYPLLW	HLA-B*53:01	10	0.841903	0.03	0.03362	100.00% (13/13)	Non-Toxin
295	Nucleoprotein	FMVEILIEV	HLA-A*02:06	9	0.969999	0.01	0.40413	100.00% (13/13)	Non-Toxin
296	Nucleoprotein	FMVEILIEV	HLA-A*02:01	9	0.974244	0.02	0.40413	100.00% (13/13)	Non-Toxin
297	Nucleoprotein	FMVEILIEV	HLA-A*02:03	9	0.947906	0.02	0.40413	100.00% (13/13)	Non-Toxin
298	Nucleoprotein	FVDSRAYGL	HLA-A*02:06	9	0.675642	0.12	-0.05536	100.00% (13/13)	Non-Toxin
299	Nucleoprotein	GLMRILKTA	HLA-A*02:03	9	0.739614	0.08	-0.04508	100.00% (13/13)	Non-Toxin
300	Nucleoprotein	GMAGFFATI	HLA-A*02:03	9	0.694943	0.1	0.3267	100.00% (13/13)	Non-Toxin
301	Nucleoprotein	GPRAPYMVL	HLA-B*07:02	9	0.994488	0.01	-0.08219	100.00% (13/13)	Non-Toxin
302	Nucleoprotein	GPRAPYMVLL	HLA-B*07:02	10	0.915242	0.04	-0.09517	100.00% (13/13)	Non-Toxin
303	Nucleoprotein	GYLEPMYFR	HLA-A*31:01	9	0.841577	0.05	-0.01367	100.00% (13/13)	Non-Toxin
304	Nucleoprotein	GYLEPMYFR	HLA-A*33:01	9	0.659681	0.08	-0.01367	100.00% (13/13)	Non-Toxin
305	Nucleoprotein	GYPLLWSF	HLA-A*23:01	8	0.576849	0.12	0.04333	100.00% (13/13)	Non-Toxin
306	Nucleoprotein	IGPRAPYMVL	HLA-B*07:02	10	0.88781	0.05	-0.05178	100.00% (13/13)	Non-Toxin
307	Nucleoprotein	IISDIGNYV	HLA-A*02:03	9	0.750805	0.07	0.1225	100.00% (13/13)	Non-Toxin
308	Nucleoprotein	IISDIGNYV	HLA-A*02:06	9	0.788065	0.08	0.1225	100.00% (13/13)	Non-Toxin
309	Nucleoprotein	IISDIGNYV	HLA-A*68:02	9	0.64388	0.11	0.1225	100.00% (13/13)	Non-Toxin
310	Nucleoprotein	ILIAKAVTA	HLA-A*02:03	9	0.664601	0.11	-0.03308	100.00% (13/13)	Non-Toxin
311	Nucleoprotein	ITIEAQIW	HLA-B*58:01	8	0.878098	0.07	0.18533	100.00% (13/13)	Non-Toxin
312	Nucleoprotein	KFAPGGYPLLW	HLA-A*23:01	11	0.701594	0.08	0.03766	100.00% (13/13)	Non-Toxin
313	Nucleoprotein	KFAPGGYPLLW	HLA-A*24:02	11	0.712723	0.09	0.03766	100.00% (13/13)	Non-Toxin
314	Nucleoprotein	KGKTPFVDSR	HLA-A*31:01	10	0.752877	0.09	0.03318	100.00% (13/13)	Non-Toxin
315	Nucleoprotein	KTARDSSKGK	HLA-A*30:01	10	0.603713	0.08	-0.39265	100.00% (13/13)	Non-Toxin
316	Nucleoprotein	KTPFVDSRAY	HLA-A*30:02	10	0.570315	0.11	0.08681	100.00% (13/13)	Non-Toxin
317	Nucleoprotein	LEESIQTKF	HLA-B*44:03	9	0.848272	0.06	-0.20665	100.00% (13/13)	Non-Toxin
318	Nucleoprotein	LEESIQTKF	HLA-B*44:02	9	0.768804	0.07	-0.20665	100.00% (13/13)	Non-Toxin
319	Nucleoprotein	MVNGIPVMER	HLA-A*68:01	10	0.956768	0.03	0.09996	100.00% (13/13)	Non-Toxin
320	Nucleoprotein	MVNGIPVMER	HLA-A*31:01	10	0.742197	0.1	0.09996	100.00% (13/13)	Non-Toxin
321	Nucleoprotein	MVNGIPVMER	HLA-A*33:01	10	0.619777	0.1	0.09996	100.00% (13/13)	Non-Toxin
322	Nucleoprotein	MVNGIPVMERR	HLA-A*68:01	11	0.865518	0.12	0.14044	100.00% (13/13)	Non-Toxin
323	Nucleoprotein	NLLSQSLSV	HLA-A*02:01	9	0.847652	0.06	-0.54462	100.00% (13/13)	Non-Toxin
324	Nucleoprotein	NPFFALTQQW	HLA-B*53:01	10	0.875745	0.03	0.0542	100.00% (13/13)	Non-Toxin
325	Nucleoprotein	NRGYLEPMYFR	HLA-A*33:01	11	0.589758	0.11	-0.01684	100.00% (13/13)	Non-Toxin
326	Nucleoprotein	NTIKSLMLL	HLA-A*68:02	9	0.719062	0.08	-0.50002	100.00% (13/13)	Non-Toxin
327	Nucleoprotein	NTIKSLMLLY	HLA-A*26:01	10	0.560325	0.09	-0.52684	100.00% (13/13)	Non-Toxin
328	Nucleoprotein	PFFALTQQW	HLA-A*23:01	9	0.643026	0.1	-0.06233	100.00% (13/13)	Non-Toxin
329	Nucleoprotein	QQKRVNPFF	HLA-B*15:01	9	0.826914	0.05	0.07523	100.00% (13/13)	Non-Toxin
330	Nucleoprotein	QTKFAPGGY	HLA-A*26:01	9	0.541353	0.1	0.12386	100.00% (13/13)	Non-Toxin
331	Nucleoprotein	QVAELAAAV	HLA-A*68:02	9	0.920491	0.02	0.19536	100.00% (13/13)	Non-Toxin
332	Nucleoprotein	REAKFAAGGVL	HLA-B*40:01	11	0.772258	0.12	0.07052	100.00% (13/13)	Non-Toxin
333	Nucleoprotein	REIGPRAPY	HLA-B*44:02	9	0.837864	0.04	0.14176	100.00% (13/13)	Non-Toxin
334	Nucleoprotein	REIGPRAPY	HLA-B*44:03	9	0.872258	0.05	0.14176	100.00% (13/13)	Non-Toxin
335	Nucleoprotein	REMSISSL	HLA-B*40:01	8	0.822674	0.1	-0.38922	100.00% (13/13)	Non-Toxin
336	Nucleoprotein	RGYLEPMYFR	HLA-A*31:01	10	0.864046	0.04	-0.02568	100.00% (13/13)	Non-Toxin

#	Protein	Peptide	Allele	Length	Score	Percentile rank	Immunogenicity	Conservancy	Toxicity
337	Nucleoprotein	RLTNSLLNLR	HLA-A*31:01	10	0.77221	0.08	-0.18819	100.00% (13/13)	Non-Toxin
338	Nucleoprotein	RPGALIRSL	HLA-B*07:02	9	0.981392	0.01	0.11187	100.00% (13/13)	Non-Toxin
339	Nucleoprotein	RPGALIRSLL	HLA-B*07:02	10	0.925615	0.04	0.07179	100.00% (13/13)	Non-Toxin
340	Nucleoprotein	RSMGALNINR	HLA-A*31:01	10	0.841767	0.05	0.10685	100.00% (13/13)	Non-Toxin
341	Nucleoprotein	RSRLAAKAAK	HLA-A*30:01	10	0.602552	0.08	-0.06528	100.00% (13/13)	Non-Toxin
342	Nucleoprotein	RSYQSKLGR	HLA-A*31:01	9	0.944357	0.01	-0.47142	100.00% (13/13)	Non-Toxin
343	Nucleoprotein	RSYQSKLGR	HLA-A*30:01	9	0.585278	0.09	-0.47142	100.00% (13/13)	Non-Toxin
344	Nucleoprotein	RSYQSKLGR	HLA-A*03:01	9	0.775679	0.11	-0.47142	100.00% (13/13)	Non-Toxin
345	Nucleoprotein	RYPALALNEF	HLA-A*24:02	10	0.937849	0.02	0.10567	100.00% (13/13)	Non-Toxin
346	Nucleoprotein	RYPALALNEF	HLA-A*23:01	10	0.866903	0.03	0.10567	100.00% (13/13)	Non-Toxin
347	Nucleoprotein	SAKGRAVEI	HLA-B*08:01	9	0.720362	0.06	0.14467	100.00% (13/13)	Non-Toxin
348	Nucleoprotein	SETRRWAKY	HLA-B*44:02	9	0.874815	0.03	0.23061	100.00% (13/13)	Non-Toxin
349	Nucleoprotein	SETRRWAKY	HLA-B*44:03	9	0.870731	0.05	0.23061	100.00% (13/13)	Non-Toxin
350	Nucleoprotein	SGRQSVTFK	HLA-A*30:01	9	0.7602	0.02	-0.12084	100.00% (13/13)	Non-Toxin
351	Nucleoprotein	SLADSVPSSSV	HLA-A*02:03	11	0.912606	0.03	-0.48869	100.00% (13/13)	Non-Toxin
352	Nucleoprotein	SLADSVPSSSV	HLA-A*02:01	11	0.906387	0.03	-0.48869	100.00% (13/13)	Non-Toxin
353	Nucleoprotein	SLADSVPSSSV	HLA-A*02:06	11	0.725435	0.1	-0.48869	100.00% (13/13)	Non-Toxin
354	Nucleoprotein	SLLNDPDIEAV	HLA-A*02:01	11	0.787782	0.09	0.25493	100.00% (13/13)	Non-Toxin
355	Nucleoprotein	SPELRWEL	HLA-B*08:01	8	0.548582	0.12	0.36475	100.00% (13/13)	Non-Toxin
356	Nucleoprotein	SQSLSVRKF	HLA-B*15:01	9	0.888119	0.03	-0.26942	100.00% (13/13)	Non-Toxin
357	Nucleoprotein	SSKGKTPFV	HLA-A*30:01	9	0.651272	0.06	-0.15032	100.00% (13/13)	Non-Toxin
358	Nucleoprotein	SVRKFMVEI	HLA-A*30:01	9	0.518708	0.12	-0.15816	100.00% (13/13)	Non-Toxin
359	Nucleoprotein	TAEESETRRW	HLA-B*44:02	10	0.924372	0.02	0.18011	100.00% (13/13)	Non-Toxin
360	Nucleoprotein	TAEESETRRW	HLA-B*44:03	10	0.914083	0.03	0.18011	100.00% (13/13)	Non-Toxin
361	Nucleoprotein	TIKSLMLLY	HLA-A*30:02	9	0.747027	0.04	-0.42841	100.00% (13/13)	Non-Toxin
362	Nucleoprotein	TIKSLMLLY	HLA-A*26:01	9	0.708049	0.06	-0.42841	100.00% (13/13)	Non-Toxin
363	Nucleoprotein	TIRFGLETR	HLA-A*33:01	9	0.563756	0.12	0.26434	100.00% (13/13)	Non-Toxin
364	Nucleoprotein	TPFVDSRAY	HLA-B*35:01	9	0.992487	0.01	0.01195	100.00% (13/13)	Non-Toxin
365	Nucleoprotein	TPFVDSRAY	HLA-B*53:01	9	0.656965	0.07	0.01195	100.00% (13/13)	Non-Toxin
366	Nucleoprotein	TTIDRSMGA	HLA-A*68:02	9	0.709706	0.08	-0.16821	100.00% (13/13)	Non-Toxin
367	Nucleoprotein	VPATNSPEL	HLA-B*07:02	9	0.931907	0.04	-0.06113	100.00% (13/13)	Non-Toxin
368	Nucleoprotein	VPATNSPEL	HLA-B*35:01	9	0.868157	0.05	-0.06113	100.00% (13/13)	Non-Toxin
369	Nucleoprotein	VPATNSPEL	HLA-B*53:01	9	0.568069	0.09	-0.06113	100.00% (13/13)	Non-Toxin
370	Nucleoprotein	VPATNSPEL	HLA-B*51:01	9	0.60984	0.12	-0.06113	100.00% (13/13)	Non-Toxin
371	Nucleoprotein	VPATNSPELRW	HLA-B*53:01	11	0.826206	0.03	-0.01131	100.00% (13/13)	Non-Toxin
372	Nucleoprotein	VPATNSPELRW	HLA-B*57:01	11	0.973675	0.04	-0.01131	100.00% (13/13)	Non-Toxin
373	Nucleoprotein	VPATNSPELRW	HLA-B*58:01	11	0.936312	0.04	-0.01131	100.00% (13/13)	Non-Toxin
374	Nucleoprotein	VQQKRVNPF	HLA-B*15:01	9	0.83591	0.05	-0.17728	100.00% (13/13)	Non-Toxin
375	Nucleoprotein	VTFKREMSI	HLA-A*32:01	9	0.464414	0.12	-0.27921	100.00% (13/13)	Non-Toxin
376	Nucleoprotein	YLEPMYFRL	HLA-A*02:01	9	0.860397	0.05	-0.0241	100.00% (13/13)	Non-Toxin
377	Nucleoprotein	YLEPMYFRL	HLA-A*02:06	9	0.703857	0.11	-0.0241	100.00% (13/13)	Non-Toxin
378	Nucleoprotein	YPALALNEF	HLA-B*35:01	9	0.993417	0.01	0.08224	100.00% (13/13)	Non-Toxin
379	Nucleoprotein	YPALALNEF	HLA-B*53:01	9	0.942342	0.01	0.08224	100.00% (13/13)	Non-Toxin
380	Nucleoprotein	YPALALNEF	HLA-B*07:02	9	0.715817	0.12	0.08224	100.00% (13/13)	Non-Toxin
381	Nucleoprotein	YPLLWSFAM	HLA-B*35:01	9	0.909678	0.04	0.16687	100.00% (13/13)	Non-Toxin
382	Phosphoprotein	AEGSDDIQL	HLA-B*40:01	9	0.882909	0.07	-0.06835	100.00% (27/27)	Non-Toxin
383	Phosphoprotein	AENDEEIQEI	HLA-B*44:02	10	0.683639	0.11	0.30124	100.00% (27/27)	Non-Toxin
384	Phosphoprotein	AQPPYHWSI	HLA-A*02:06	9	0.788192	0.08	0.10237	62.96% (17/27)	Non-Toxin
385	Phosphoprotein	AVKEEPPQK	HLA-A*30:01	9	0.892611	0.01	0.04077	62.96% (17/27)	Non-Toxin

#	Protein	Peptide	Allele	Length	Score	Percentile rank	Immunogenicity	Conservancy	Toxicity
386	Phosphoprotein	AVKEEPPQK	HLA-A*11:01	9	0.901199	0.02	0.04077	62.96% (17/27)	Non-Toxin
387	Phosphoprotein	AVKEEPPQK	HLA-A*03:01	9	0.883607	0.04	0.04077	62.96% (17/27)	Non-Toxin
388	Phosphoprotein	CLVSDAKMLSY	HLA-A*01:01	11	0.667959	0.1	-0.57469	59.26% (16/27)	Non-Toxin
389	Phosphoprotein	DAQPPYHW	HLA-B*53:01	8	0.601371	0.08	-0.03574	62.96% (17/27)	Non-Toxin
390	Phosphoprotein	DAQPPYHWSI	HLA-B*51:01	10	0.606259	0.12	0.05757	62.96% (17/27)	Non-Toxin
391	Phosphoprotein	DILEQQSLF	HLA-A*26:01	9	0.609284	0.08	-0.27079	62.96% (17/27)	Non-Toxin
392	Phosphoprotein	DPAKDSPVI	HLA-B*51:01	9	0.73593	0.06	-0.32367	55.56% (15/27)	Non-Toxin
393	Phosphoprotein	DPVVTDVVY	HLA-B*35:01	9	0.932553	0.03	0.17258	100.00% (27/27)	Non-Toxin
394	Phosphoprotein	EIPKIINKL	HLA-A*68:02	9	0.801002	0.05	-0.09718	100.00% (27/27)	Non-Toxin
395	Phosphoprotein	EIPKIINKL	HLA-A*26:01	9	0.591638	0.08	-0.09718	100.00% (27/27)	Non-Toxin
396	Phosphoprotein	EIQEIANTV	HLA-A*68:02	9	0.908477	0.02	0.2468	100.00% (27/27)	Non-Toxin
397	Phosphoprotein	ELRSELIGY	HLA-A*26:01	9	0.770159	0.05	0.06951	96.30% (26/27)	Non-Toxin
398	Phosphoprotein	EPYGAAVQL	HLA-B*51:01	9	0.763162	0.05	0.07499	100.00% (27/27)	Non-Toxin
399	Phosphoprotein	EPYGAAVQL	HLA-B*35:01	9	0.763708	0.09	0.07499	100.00% (27/27)	Non-Toxin
400	Phosphoprotein	EPYGAAVQL	HLA-B*53:01	9	0.526772	0.12	0.07499	100.00% (27/27)	Non-Toxin
401	Phosphoprotein	ESIDRVLAK	HLA-A*68:01	9	0.96351	0.02	0.16828	100.00% (27/27)	Non-Toxin
402	Phosphoprotein	FPHDTDRLNY	HLA-B*35:01	10	0.969478	0.02	0.1278	100.00% (27/27)	Non-Toxin
403	Phosphoprotein	FPHDTDRLNY	HLA-B*53:01	10	0.768043	0.04	0.1278	100.00% (27/27)	Non-Toxin
404	Phosphoprotein	FSFDNVKNF	HLA-A*26:01	9	0.498887	0.12	-0.0929	100.00% (27/27)	Non-Toxin
405	Phosphoprotein	FSFDNVKNFR	HLA-A*68:01	10	0.889376	0.08	-0.04584	100.00% (27/27)	Non-Toxin
406	Phosphoprotein	FTSSPERGW	HLA-B*58:01	9	0.966072	0.02	-0.07324	100.00% (27/27)	Non-Toxin
407	Phosphoprotein	FTSSPERGW	HLA-B*57:01	9	0.970202	0.04	-0.07324	100.00% (27/27)	Non-Toxin
408	Phosphoprotein	GLNPTAVPF	HLA-B*15:01	9	0.800904	0.07	0.08973	62.96% (17/27)	Non-Toxin
409	Phosphoprotein	GPQTSRNVNL	HLA-B*07:02	10	0.865424	0.06	-0.08427	62.96% (17/27)	Non-Toxin
410	Phosphoprotein	GSEDPIIREL	HLA-B*40:01	10	0.90568	0.06	0.40108	62.96% (17/27)	Non-Toxin
411	Phosphoprotein	GTIGKRVSNTR	HLA-A*31:01	11	0.734465	0.11	-0.18061	100.00% (27/27)	Non-Toxin
412	Phosphoprotein	IPIKKGTDAKY	HLA-B*35:01	11	0.715985	0.12	-0.3851	55.56% (15/27)	Non-Toxin
413	Phosphoprotein	IPKIINKL	HLA-B*51:01	8	0.687106	0.08	0.00543	100.00% (27/27)	Non-Toxin
414	Phosphoprotein	IQLDPVVTDV	HLA-A*02:06	10	0.787286	0.08	0.1327	100.00% (27/27)	Non-Toxin
415	Phosphoprotein	IQLDPVVTDVV	HLA-A*02:06	11	0.735367	0.1	0.1677	100.00% (27/27)	Non-Toxin
416	Phosphoprotein	KDAQPPYHW	HLA-B*44:02	9	0.781823	0.06	-0.10932	62.96% (17/27)	Non-Toxin
417	Phosphoprotein	KEEPPQKRL	HLA-B*40:01	9	0.929366	0.05	-0.25026	62.96% (17/27)	Non-Toxin
418	Phosphoprotein	KEEPPQKRL	HLA-B*44:02	9	0.787822	0.05	-0.25026	62.96% (17/27)	Non-Toxin
419	Phosphoprotein	KEEPPQKRL	HLA-B*44:03	9	0.801772	0.08	-0.25026	62.96% (17/27)	Non-Toxin
420	Phosphoprotein	KEIPKIINKL	HLA-B*40:01	10	0.904097	0.06	-0.05454	100.00% (27/27)	Non-Toxin
421	Phosphoprotein	KIINKLESI	HLA-A*02:03	9	0.806346	0.05	-0.19591	100.00% (27/27)	Non-Toxin
422	Phosphoprotein	KIINKLESI	HLA-A*32:01	9	0.600792	0.06	-0.19591	100.00% (27/27)	Non-Toxin
423	Phosphoprotein	KIINKLESI	HLA-A*02:06	9	0.814257	0.07	-0.19591	100.00% (27/27)	Non-Toxin
424	Phosphoprotein	KLESIDRVLAK	HLA-A*03:01	11	0.891681	0.03	0.11999	100.00% (27/27)	Non-Toxin
425	Phosphoprotein	KLINLDMRL	HLA-A*02:01	9	0.902861	0.03	-0.07119	100.00% (27/27)	Non-Toxin
426	Phosphoprotein	KLINLDMRL	HLA-A*02:06	9	0.744941	0.09	-0.07119	100.00% (27/27)	Non-Toxin
427	Phosphoprotein	KMLSYAPEI	HLA-A*02:01	9	0.836092	0.06	-0.0877	59.26% (16/27)	Non-Toxin
428	Phosphoprotein	KMLSYAPEI	HLA-A*32:01	9	0.521775	0.09	-0.0877	59.26% (16/27)	Non-Toxin
429	Phosphoprotein	KNQKEIQKTY	HLA-B*15:01	10	0.948535	0.01	-0.29586	100.00% (27/27)	Non-Toxin
430	Phosphoprotein	KNQKEIQKTY	HLA-A*30:02	10	0.596421	0.11	-0.29586	100.00% (27/27)	Non-Toxin
431	Phosphoprotein	KPNESIGRTI	HLA-B*07:02	10	0.723959	0.11	0.16541	59.26% (16/27)	Non-Toxin
432	Phosphoprotein	KPVIGRDIL	HLA-B*07:02	9	0.912854	0.04	0.32552	62.96% (17/27)	Non-Toxin
433	Phosphoprotein	KSRGIPIKK	HLA-A*30:01	9	0.969386	0.01	0.15638	100.00% (27/27)	Non-Toxin
434	Phosphoprotein	KSRGIPIKK	HLA-A*03:01	9	0.889371	0.04	0.15638	100.00% (27/27)	Non-Toxin

#	Protein	Peptide	Allele	Length	Score	Percentile rank	Immunogenicity	Conservancy	Toxicity
435	Phosphoprotein	KSRGIPIKK	HLA-A*31:01	9	0.738898	0.1	0.15638	100.00% (27/27)	Non-Toxin
436	Phosphoprotein	KTLIRTHIK	HLA-A*30:01	9	0.821307	0.02	0.32232	100.00% (27/27)	Non-Toxin
437	Phosphoprotein	KTNTALSTI	HLA-A*32:01	9	0.51221	0.1	-0.05232	100.00% (27/27)	Non-Toxin
438	Phosphoprotein	KYIMPSDDF	HLA-A*24:02	9	0.820254	0.05	-0.26835	96.30% (26/27)	Non-Toxin
439	Phosphoprotein	KYIMPSDDF	HLA-A*23:01	9	0.684082	0.09	-0.26835	96.30% (26/27)	Non-Toxin
440	Phosphoprotein	LDPVVTDVVY	HLA-B*35:01	10	0.768381	0.09	0.19578	100.00% (27/27)	Non-Toxin
441	Phosphoprotein	LEQQSLFSF	HLA-B*44:03	9	0.747962	0.1	-0.32356	100.00% (27/27)	Non-Toxin
442	Phosphoprotein	LEQQSLFSF	HLA-B*44:02	9	0.661797	0.12	-0.32356	100.00% (27/27)	Non-Toxin
443	Phosphoprotein	LVSDAKMLSY	HLA-A*01:01	10	0.928402	0.02	-0.4746	59.26% (16/27)	Non-Toxin
444	Phosphoprotein	MPKSRGIPI	HLA-B*08:01	9	0.812819	0.03	-0.04833	100.00% (27/27)	Non-Toxin
445	Phosphoprotein	MPKSRGIPI	HLA-B*07:02	9	0.939144	0.04	-0.04833	100.00% (27/27)	Non-Toxin
446	Phosphoprotein	MPSDDFSNTF	HLA-B*35:01	10	0.97685	0.01	-0.03429	96.30% (26/27)	Non-Toxin
447	Phosphoprotein	MPSDDFSNTF	HLA-B*53:01	10	0.926862	0.02	-0.03429	96.30% (26/27)	Non-Toxin
448	Phosphoprotein	MPSDDFSNTFF	HLA-B*53:01	11	0.829443	0.03	0.03819	96.30% (26/27)	Non-Toxin
449	Phosphoprotein	MPSDDFSNTFF	HLA-B*35:01	11	0.811041	0.07	0.03819	96.30% (26/27)	Non-Toxin
450	Phosphoprotein	NEPYGAAVQL	HLA-B*40:01	10	0.850427	0.08	0.06777	100.00% (27/27)	Non-Toxin
451	Phosphoprotein	NPTAVPFTL	HLA-B*53:01	9	0.830012	0.03	0.20321	51.85% (14/27)	Non-Toxin
452	Phosphoprotein	NPTAVPFTL	HLA-B*35:01	9	0.8582	0.06	0.20321	51.85% (14/27)	Non-Toxin
453	Phosphoprotein	NPTAVPFTL	HLA-B*51:01	9	0.680366	0.08	0.20321	51.85% (14/27)	Non-Toxin
454	Phosphoprotein	NPTAVPFTL	HLA-B*07:02	9	0.712601	0.12	0.20321	51.85% (14/27)	Non-Toxin
455	Phosphoprotein	NPTAVPFTLR	HLA-A*68:01	10	0.878383	0.11	0.21785	51.85% (14/27)	Non-Toxin
456	Phosphoprotein	NQKEIQKTY	HLA-B*15:01	9	0.96002	0.01	-0.10801	100.00% (27/27)	Non-Toxin
457	Phosphoprotein	NTFFPHDTDR	HLA-A*68:01	10	0.934647	0.04	0.2431	100.00% (27/27)	Non-Toxin
458	Phosphoprotein	NVNLDSIKLY	HLA-A*26:01	10	0.542042	0.1	-0.21596	100.00% (27/27)	Non-Toxin
459	Phosphoprotein	QKNQKEIQKTY	HLA-B*15:01	11	0.794229	0.07	-0.36992	100.00% (27/27)	Non-Toxin
460	Phosphoprotein	QLDPVVTDV	HLA-A*02:01	9	0.870003	0.05	0.12082	100.00% (27/27)	Non-Toxin
461	Phosphoprotein	QLDPVVTDV	HLA-A*02:06	9	0.714887	0.11	0.12082	100.00% (27/27)	Non-Toxin
462	Phosphoprotein	QLDPVVTDVVY	HLA-A*01:01	11	0.83942	0.05	0.19408	100.00% (27/27)	Non-Toxin
463	Phosphoprotein	QPPYHWSI	HLA-B*51:01	8	0.619756	0.12	0.09307	62.96% (17/27)	Non-Toxin
464	Phosphoprotein	QPSIKDQTKAW	HLA-B*53:01	11	0.626939	0.07	-0.34358	51.85% (14/27)	Non-Toxin
465	Phosphoprotein	QQGKDAQPPY	HLA-B*15:01	10	0.871265	0.03	-0.27118	59.26% (16/27)	Non-Toxin
466	Phosphoprotein	REDLILPEL	HLA-B*40:01	9	0.980354	0.01	0.16434	62.96% (17/27)	Non-Toxin
467	Phosphoprotein	RELLKENSL	HLA-B*40:01	9	0.956669	0.02	-0.23263	62.96% (17/27)	Non-Toxin
468	Phosphoprotein	RETDLVHL	HLA-B*40:01	8	0.804553	0.1	0.09028	100.00% (27/27)	Non-Toxin
469	Phosphoprotein	RLNHIEEQV	HLA-A*02:03	9	0.755732	0.07	0.27112	100.00% (27/27)	Non-Toxin
470	Phosphoprotein	RLNHIEEQV	HLA-A*02:01	9	0.811652	0.08	0.27112	100.00% (27/27)	Non-Toxin
471	Phosphoprotein	RQRPGTPMPK	HLA-A*30:01	10	0.918589	0.01	-0.08868	62.96% (17/27)	Non-Toxin
472	Phosphoprotein	RQRPGTPMPK	HLA-A*03:01	10	0.796456	0.09	-0.08868	62.96% (17/27)	Non-Toxin
473	Phosphoprotein	RTIEGQSIR	HLA-A*31:01	9	0.856405	0.04	0.00605	59.26% (16/27)	Non-Toxin
474	Phosphoprotein	RVLAKTNTA	HLA-A*30:01	9	0.524121	0.12	-0.12047	100.00% (27/27)	Non-Toxin
475	Phosphoprotein	SDAKMLSY	HLA-A*01:01	8	0.630839	0.12	-0.52536	59.26% (16/27)	Non-Toxin
476	Phosphoprotein	SEDPIIREL	HLA-B*40:01	9	0.994468	0.01	0.3531	62.96% (17/27)	Non-Toxin
477	Phosphoprotein	SEDPIIREL	HLA-B*44:03	9	0.974423	0.01	0.3531	62.96% (17/27)	Non-Toxin
478	Phosphoprotein	SEDPIIREL	HLA-B*44:02	9	0.959815	0.01	0.3531	62.96% (17/27)	Non-Toxin
479	Phosphoprotein	SEDPIIRELL	HLA-B*40:01	10	0.925972	0.05	0.38198	62.96% (17/27)	Non-Toxin
480	Phosphoprotein	SEDPIIRELL	HLA-B*44:02	10	0.705619	0.1	0.38198	62.96% (17/27)	Non-Toxin
481	Phosphoprotein	SEDPIIRELL	HLA-B*44:03	10	0.743206	0.11	0.38198	62.96% (17/27)	Non-Toxin
482	Phosphoprotein	SFDNVKNFR	HLA-A*33:01	9	0.608098	0.11	-0.09917	100.00% (27/27)	Non-Toxin
483	Phosphoprotein	SIRDNLQAK	HLA-A*30:01	9	0.814193	0.02	-0.05252	51.85% (14/27)	Non-Toxin

#	Protein	Peptide	Allele	Length	Score	Percentile rank	Immunogenicity	Conservancy	Toxicity
484	Phosphoprotein	SPVIAEHYY	HLA-B*35:01	9	0.967986	0.02	0.30481	96.30% (26/27)	Non-Toxin
485	Phosphoprotein	SPVIAEHYY	HLA-B*53:01	9	0.694604	0.06	0.30481	96.30% (26/27)	Non-Toxin
486	Phosphoprotein	SSIQQPSIK	HLA-A*11:01	9	0.782261	0.09	-0.25846	100.00% (27/27)	Non-Toxin
487	Phosphoprotein	SSRDVIKTL	HLA-A*30:01	9	0.548679	0.1	0.04528	51.85% (14/27)	Non-Toxin
488	Phosphoprotein	SSSEVIVGI	HLA-A*68:02	9	0.833703	0.04	0.26717	100.00% (27/27)	Non-Toxin
489	Phosphoprotein	STIEGHLVSM	HLA-A*26:01	10	0.612154	0.08	0.13619	100.00% (27/27)	Non-Toxin
490	Phosphoprotein	STSPTDGTIGK	HLA-A*11:01	11	0.936819	0.01	0.1962	62.96% (17/27)	Non-Toxin
491	Phosphoprotein	TFFPHDTDR	HLA-A*33:01	9	0.623034	0.1	0.12494	100.00% (27/27)	Non-Toxin
492	Phosphoprotein	TPMPKSRGI	HLA-B*07:02	9	0.892711	0.05	-0.37041	100.00% (27/27)	Non-Toxin
493	Phosphoprotein	TPMPKSRGI	HLA-B*51:01	9	0.709065	0.07	-0.37041	100.00% (27/27)	Non-Toxin
494	Phosphoprotein	TPMPKSRGI	HLA-B*08:01	9	0.547576	0.12	-0.37041	100.00% (27/27)	Non-Toxin
495	Phosphoprotein	VLMGVINSI	HLA-A*02:03	9	0.891655	0.03	0.04046	100.00% (27/27)	Non-Toxin
496	Phosphoprotein	VLMGVINSI	HLA-A*02:01	9	0.889812	0.04	0.04046	100.00% (27/27)	Non-Toxin
497	Phosphoprotein	VLMGVINSI	HLA-A*02:06	9	0.786218	0.08	0.04046	100.00% (27/27)	Non-Toxin
498	Phosphoprotein	VLMGVINSI	HLA-A*32:01	9	0.499192	0.1	0.04046	100.00% (27/27)	Non-Toxin
499	Phosphoprotein	VSDAKMLSY	HLA-A*01:01	9	0.994971	0.01	-0.43475	59.26% (16/27)	Non-Toxin
500	Phosphoprotein	VSDAKMLSY	HLA-A*30:02	9	0.699815	0.06	-0.43475	59.26% (16/27)	Non-Toxin
501	Phosphoprotein	YGFTSSPERGW	HLA-B*57:01	11	0.915052	0.09	-0.09821	100.00% (27/27)	Non-Toxin
502	Phosphoprotein	YPSAGTENV	HLA-B*51:01	9	0.630068	0.11	0.13593	92.59% (25/27)	Non-Toxin
503	V Protein	AEGSDDIQL	HLA-B*40:01	9	0.882909	0.07	-0.06835	94.74% (54/57)	Non-Toxin
504	V Protein	AQPPYHWSI	HLA-A*02:06	9	0.788192	0.08	0.10237	19.30% (11/57)	Non-Toxin
505	V Protein	AVKEEPPQK	HLA-A*30:01	9	0.892611	0.01	0.04077	19.30% (11/57)	Non-Toxin
506	V Protein	AVKEEPPQK	HLA-A*11:01	9	0.901199	0.02	0.04077	19.30% (11/57)	Non-Toxin
507	V Protein	AVKEEPPQK	HLA-A*03:01	9	0.883607	0.04	0.04077	19.30% (11/57)	Non-Toxin
508	V Protein	CLVSDAKMLSY	HLA-A*01:01	11	0.667959	0.1	-0.57469	19.30% (11/57)	Non-Toxin
509	V Protein	DAQPPYHW	HLA-B*53:01	8	0.601371	0.08	-0.03574	19.30% (11/57)	Non-Toxin
510	V Protein	DAQPPYHWSI	HLA-B*51:01	10	0.606259	0.12	0.05757	19.30% (11/57)	Non-Toxin
511	V Protein	DPAKDSPVI	HLA-B*51:01	9	0.73593	0.06	-0.32367	17.54% (10/57)	Non-Toxin
512	V Protein	DPVVTDVVY	HLA-B*35:01	9	0.932553	0.03	0.17258	98.25% (56/57)	Non-Toxin
513	V Protein	FTSSPERGW	HLA-B*58:01	9	0.966072	0.02	-0.07324	100.00% (57/57)	Non-Toxin
514	V Protein	FTSSPERGW	HLA-B*57:01	9	0.970202	0.04	-0.07324	100.00% (57/57)	Non-Toxin
515	V Protein	GLNPTAVPF	HLA-B*15:01	9	0.800904	0.07	0.08973	19.30% (11/57)	Non-Toxin
516	V Protein	GPQTSRNVNL	HLA-B*07:02	10	0.865424	0.06	-0.08427	19.30% (11/57)	Non-Toxin
517	V Protein	GSEDPIIREL	HLA-B*40:01	10	0.90568	0.06	0.40108	19.30% (11/57)	Non-Toxin
518	V Protein	GTIGKRVSNTR	HLA-A*31:01	11	0.734465	0.11	-0.18061	100.00% (57/57)	Non-Toxin
519	V Protein	IQLDPVVTDV	HLA-A*02:06	10	0.787286	0.08	0.1327	94.74% (54/57)	Non-Toxin
520	V Protein	IQLDPVVTDVV	HLA-A*02:06	11	0.735367	0.1	0.1677	94.74% (54/57)	Non-Toxin
521	V Protein	ISICWDGKRAW	HLA-B*57:01	11	0.974216	0.04	0.12279	87.72% (50/57)	Non-Toxin
522	V Protein	ISICWDGKRAW	HLA-B*58:01	11	0.85944	0.08	0.12279	87.72% (50/57)	Non-Toxin
523	V Protein	KDAQPPYHW	HLA-B*44:02	9	0.781823	0.06	-0.10932	19.30% (11/57)	Non-Toxin
524	V Protein	KEEPPQKRL	HLA-B*40:01	9	0.929366	0.05	-0.25026	19.30% (11/57)	Non-Toxin
525	V Protein	KEEPPQKRL	HLA-B*44:02	9	0.787822	0.05	-0.25026	19.30% (11/57)	Non-Toxin
526	V Protein	KEEPPQKRL	HLA-B*44:03	9	0.801772	0.08	-0.25026	19.30% (11/57)	Non-Toxin
527	V Protein	KMLSYAPEI	HLA-A*02:01	9	0.836092	0.06	-0.0877	19.30% (11/57)	Non-Toxin
528	V Protein	KMLSYAPEI	HLA-A*32:01	9	0.521775	0.09	-0.0877	19.30% (11/57)	Non-Toxin
529	V Protein	KNQKEIQKTY	HLA-B*15:01	10	0.948535	0.01	-0.29586	100.00% (57/57)	Non-Toxin
530	V Protein	KNQKEIQKTY	HLA-A*30:02	10	0.596421	0.11	-0.29586	100.00% (57/57)	Non-Toxin
531	V Protein	KPNESIGRTI	HLA-B*07:02	10	0.723959	0.11	0.16541	19.30% (11/57)	Non-Toxin
532	V Protein	KSRGIPIKK	HLA-A*30:01	9	0.969386	0.01	0.15638	100.00% (57/57)	Non-Toxin

#	Protein	Peptide	Allele	Length	Score	Percentile rank	Immunogenicity	Conservancy	Toxicity
533	V Protein	KSRGIPIKK	HLA-A*03:01	9	0.889371	0.04	0.15638	100.00% (57/57)	Non-Toxin
534	V Protein	KSRGIPIKK	HLA-A*31:01	9	0.738898	0.1	0.15638	100.00% (57/57)	Non-Toxin
535	V Protein	LDPVVTDVVY	HLA-B*35:01	10	0.768381	0.09	0.19578	98.25% (56/57)	Non-Toxin
536	V Protein	LVSDAKMLSY	HLA-A*01:01	10	0.928402	0.02	-0.4746	19.30% (11/57)	Non-Toxin
537	V Protein	MPKSRGIPI	HLA-B*08:01	9	0.812819	0.03	-0.04833	100.00% (57/57)	Non-Toxin
538	V Protein	MPKSRGIPI	HLA-B*07:02	9	0.939144	0.04	-0.04833	100.00% (57/57)	Non-Toxin
539	V Protein	NPTAVPFTL	HLA-B*53:01	9	0.830012	0.03	0.20321	19.30% (11/57)	Non-Toxin
540	V Protein	NPTAVPFTL	HLA-B*35:01	9	0.8582	0.06	0.20321	19.30% (11/57)	Non-Toxin
541	V Protein	NPTAVPFTL	HLA-B*51:01	9	0.680366	0.08	0.20321	19.30% (11/57)	Non-Toxin
542	V Protein	NPTAVPFTL	HLA-B*07:02	9	0.712601	0.12	0.20321	19.30% (11/57)	Non-Toxin
543	V Protein	NPTAVPFTLR	HLA-A*68:01	10	0.878383	0.11	0.21785	19.30% (11/57)	Non-Toxin
544	V Protein	NQKEIQKTY	HLA-B*15:01	9	0.96002	0.01	-0.10801	100.00% (57/57)	Non-Toxin
545	V Protein	NVNLDSIKLY	HLA-A*26:01	10	0.542042	0.1	-0.21596	100.00% (57/57)	Non-Toxin
546	V Protein	QKNQKEIQKTY	HLA-B*15:01	11	0.794229	0.07	-0.36992	100.00% (57/57)	Non-Toxin
547	V Protein	QLDPVVTDV	HLA-A*02:01	9	0.870003	0.05	0.12082	100.00% (57/57)	Non-Toxin
548	V Protein	QLDPVVTDV	HLA-A*02:06	9	0.714887	0.11	0.12082	100.00% (57/57)	Non-Toxin
549	V Protein	QLDPVVTDVVY	HLA-A*01:01	11	0.83942	0.05	0.19408	98.25% (56/57)	Non-Toxin
550	V Protein	QPPYHWSI	HLA-B*51:01	8	0.619756	0.12	0.09307	19.30% (11/57)	Non-Toxin
551	V Protein	QPSIKDQTKAW	HLA-B*53:01	11	0.626939	0.07	-0.34358	19.30% (11/57)	Non-Toxin
552	V Protein	QQGKDAQPPY	HLA-B*15:01	10	0.871265	0.03	-0.27118	19.30% (11/57)	Non-Toxin
553	V Protein	RELLKENSL	HLA-B*40:01	9	0.956669	0.02	-0.23263	19.30% (11/57)	Non-Toxin
554	V Protein	RETDLVHL	HLA-B*40:01	8	0.804553	0.1	0.09028	98.25% (56/57)	Non-Toxin
555	V Protein	RQRPGTPMPK	HLA-A*30:01	10	0.918589	0.01	-0.08868	19.30% (11/57)	Non-Toxin
556	V Protein	RQRPGTPMPK	HLA-A*03:01	10	0.796456	0.09	-0.08868	19.30% (11/57)	Non-Toxin
557	V Protein	RTIEGQSIR	HLA-A*31:01	9	0.856405	0.04	0.00605	19.30% (11/57)	Non-Toxin
558	V Protein	SDAKMLSY	HLA-A*01:01	8	0.630839	0.12	-0.52536	19.30% (11/57)	Non-Toxin
559	V Protein	SEDPIIREL	HLA-B*40:01	9	0.994468	0.01	0.3531	19.30% (11/57)	Non-Toxin
560	V Protein	SEDPIIREL	HLA-B*44:03	9	0.974423	0.01	0.3531	19.30% (11/57)	Non-Toxin
561	V Protein	SEDPIIREL	HLA-B*44:02	9	0.959815	0.01	0.3531	19.30% (11/57)	Non-Toxin
562	V Protein	SEDPIIRELL	HLA-B*40:01	10	0.925972	0.05	0.38198	19.30% (11/57)	Non-Toxin
563	V Protein	SEDPIIRELL	HLA-B*44:02	10	0.705619	0.1	0.38198	19.30% (11/57)	Non-Toxin
564	V Protein	SEDPIIRELL	HLA-B*44:03	10	0.743206	0.11	0.38198	19.30% (11/57)	Non-Toxin
565	V Protein	SIRDNLQAK	HLA-A*30:01	9	0.814193	0.02	-0.05252	19.30% (11/57)	Non-Toxin
566	V Protein	SPVIAEHYY	HLA-B*35:01	9	0.967986	0.02	0.30481	98.25% (56/57)	Non-Toxin
567	V Protein	SPVIAEHYY	HLA-B*53:01	9	0.694604	0.06	0.30481	98.25% (56/57)	Non-Toxin
568	V Protein	SSIQQPSIK	HLA-A*11:01	9	0.782261	0.09	-0.25846	100.00% (57/57)	Non-Toxin
569	V Protein	SSSEVIVGI	HLA-A*68:02	9	0.833703	0.04	0.26717	98.25% (56/57)	Non-Toxin
570	V Protein	STSPTDGTIGK	HLA-A*11:01	11	0.936819	0.01	0.1962	100.00% (57/57)	Non-Toxin
571	V Protein	TPMPKSRGI	HLA-B*07:02	9	0.892711	0.05	-0.37041	100.00% (57/57)	Non-Toxin
572	V Protein	TPMPKSRGI	HLA-B*51:01	9	0.709065	0.07	-0.37041	100.00% (57/57)	Non-Toxin
573	V Protein	TPMPKSRGI	HLA-B*08:01	9	0.547576	0.12	-0.37041	100.00% (57/57)	Non-Toxin
574	V Protein	VSDAKMLSY	HLA-A*01:01	9	0.994971	0.01	-0.43475	19.30% (11/57)	Non-Toxin
575	V Protein	VSDAKMLSY	HLA-A*30:02	9	0.699815	0.06	-0.43475	19.30% (11/57)	Non-Toxin
576	V Protein	YGFTSSPERGW	HLA-B*57:01	11	0.915052	0.09	-0.09821	100.00% (57/57)	Non-Toxin
577	W Protein	AEGSDDIQL	HLA-B*40:01	9	0.882909	0.07	-0.06835	100.00% (39/39)	Non-Toxin
578	W Protein	AVKEKSPQK	HLA-A*30:01	9	0.945499	0.01	-0.41202	79.49% (31/39)	Non-Toxin
579	W Protein	AVKEKSPQK	HLA-A*03:01	9	0.928687	0.02	-0.41202	79.49% (31/39)	Non-Toxin
580	W Protein	AVKEKSPQK	HLA-A*11:01	9	0.835471	0.06	-0.41202	79.49% (31/39)	Non-Toxin
581	W Protein	CLVSDAKVLSY	HLA-A*01:01	11	0.72848	0.09	-0.37053	76.92% (30/39)	Non-Toxin

#	Protein	Peptide	Allele	Length	Score	Percentile rank	Immunogenicity	Conservancy	Toxicity
582	W Protein	DPVVTDVVY	HLA-B*35:01	9	0.932553	0.03	0.17258	97.44% (38/39)	Non-Toxin
583	W Protein	FTSSPERGW	HLA-B*58:01	9	0.966072	0.02	-0.07324	100.00% (39/39)	Non-Toxin
584	W Protein	FTSSPERGW	HLA-B*57:01	9	0.970202	0.04	-0.07324	100.00% (39/39)	Non-Toxin
585	W Protein	GLNPTAIPF	HLA-B*15:01	9	0.761814	0.09	0.16721	79.49% (31/39)	Non-Toxin
586	W Protein	GMFEDHPPTK	HLA-A*03:01	10	0.923007	0.02	0.19473	100.00% (39/39)	Non-Toxin
587	W Protein	GMFEDHPPTKK	HLA-A*03:01	11	0.930908	0.02	0.07737	100.00% (39/39)	Non-Toxin
588	W Protein	GSEDPIIQEL	HLA-B*40:01	10	0.955951	0.03	0.25964	79.49% (31/39)	Non-Toxin
589	W Protein	GTIGKRVSNTR	HLA-A*31:01	11	0.734465	0.11	-0.18061	100.00% (39/39)	Non-Toxin
590	W Protein	HIIEGQSTR	HLA-A*68:01	9	0.94979	0.03	-0.04903	33.33% (13/39)	Non-Toxin
591	W Protein	HLLGRKTCL	HLA-B*08:01	9	0.892932	0.02	-0.12084	92.31% (36/39)	Non-Toxin
592	W Protein	HPPTKKARVSM	HLA-B*07:02	11	0.84177	0.07	-0.35954	100.00% (39/39)	Non-Toxin
593	W Protein	IPFTPKNL	HLA-B*51:01	8	0.707336	0.07	-0.1422	79.49% (31/39)	Non-Toxin
594	W Protein	IPFTPKNLSV	HLA-B*51:01	10	0.838223	0.03	-0.25585	76.92% (30/39)	Non-Toxin
595	W Protein	IQLDPVVTDV	HLA-A*02:06	10	0.787286	0.08	0.1327	100.00% (39/39)	Non-Toxin
596	W Protein	IQLDPVVTDVV	HLA-A*02:06	11	0.735367	0.1	0.1677	100.00% (39/39)	Non-Toxin
597	W Protein	KEKSPQKRL	HLA-B*44:02	9	0.697768	0.1	-0.50807	79.49% (31/39)	Non-Toxin
598	W Protein	KNQKEIQKTY	HLA-B*15:01	10	0.948535	0.01	-0.29586	100.00% (39/39)	Non-Toxin
599	W Protein	KNQKEIQKTY	HLA-A*30:02	10	0.596421	0.11	-0.29586	100.00% (39/39)	Non-Toxin
600	W Protein	KQRPGTPMPK	HLA-A*30:01	10	0.915351	0.01	-0.08868	79.49% (31/39)	Non-Toxin
601	W Protein	KQRPGTPMPK	HLA-A*03:01	10	0.799411	0.09	-0.08868	79.49% (31/39)	Non-Toxin
602	W Protein	KSRGIPIKK	HLA-A*30:01	9	0.969386	0.01	0.15638	100.00% (39/39)	Non-Toxin
603	W Protein	KSRGIPIKK	HLA-A*03:01	9	0.889371	0.04	0.15638	100.00% (39/39)	Non-Toxin
604	W Protein	KSRGIPIKK	HLA-A*31:01	9	0.738898	0.1	0.15638	100.00% (39/39)	Non-Toxin
605	W Protein	KVLSYAPEI	HLA-A*32:01	9	0.600689	0.06	-0.0877	76.92% (30/39)	Non-Toxin
606	W Protein	KVLSYAPEI	HLA-A*02:06	9	0.752378	0.09	-0.0877	76.92% (30/39)	Non-Toxin
607	W Protein	LDPVVTDVVY	HLA-B*35:01	10	0.768381	0.09	0.19578	97.44% (38/39)	Non-Toxin
608	W Protein	LVSDAKVLSY	HLA-A*01:01	10	0.949801	0.02	-0.27044	76.92% (30/39)	Non-Toxin
609	W Protein	MPKSRGIPI	HLA-B*08:01	9	0.812819	0.03	-0.04833	100.00% (39/39)	Non-Toxin
610	W Protein	MPKSRGIPI	HLA-B*07:02	9	0.939144	0.04	-0.04833	100.00% (39/39)	Non-Toxin
611	W Protein	NQKEIQKTY	HLA-B*15:01	9	0.96002	0.01	-0.10801	100.00% (39/39)	Non-Toxin
612	W Protein	NVNLDSIKLY	HLA-A*26:01	10	0.542042	0.1	-0.21596	100.00% (39/39)	Non-Toxin
613	W Protein	QELLKENSF	HLA-B*44:02	9	0.85788	0.03	-0.23263	79.49% (31/39)	Non-Toxin
614	W Protein	QELLKENSF	HLA-B*44:03	9	0.877268	0.04	-0.23263	79.49% (31/39)	Non-Toxin
615	W Protein	QGKDAQPLYY	HLA-A*01:01	10	0.671592	0.1	-0.14434	79.49% (31/39)	Non-Toxin
616	W Protein	QKNQKEIQKTY	HLA-B*15:01	11	0.794229	0.07	-0.36992	100.00% (39/39)	Non-Toxin
617	W Protein	QLDPVVTDV	HLA-A*02:01	9	0.870003	0.05	0.12082	100.00% (39/39)	Non-Toxin
618	W Protein	QLDPVVTDV	HLA-A*02:06	9	0.714887	0.11	0.12082	100.00% (39/39)	Non-Toxin
619	W Protein	QLDPVVTDVVY	HLA-A*01:01	11	0.83942	0.05	0.19408	97.44% (38/39)	Non-Toxin
620	W Protein	QQGKDAQPLY	HLA-B*15:01	10	0.888736	0.03	-0.27118	79.49% (31/39)	Non-Toxin
621	W Protein	RETDLVHL	HLA-B*40:01	8	0.804553	0.1	0.09028	100.00% (39/39)	Non-Toxin
622	W Protein	SAVKEKSPQK	HLA-A*30:01	10	0.597659	0.08	-0.54887	76.92% (30/39)	Non-Toxin
623	W Protein	SDAKVLSY	HLA-A*01:01	8	0.693711	0.1	-0.31416	76.92% (30/39)	Non-Toxin
624	W Protein	SEDPIIQEL	HLA-B*40:01	9	0.996375	0.01	0.21166	79.49% (31/39)	Non-Toxin
625	W Protein	SEDPIIQEL	HLA-B*44:03	9	0.963793	0.01	0.21166	79.49% (31/39)	Non-Toxin
626	W Protein	SEDPIIQEL	HLA-B*44:02	9	0.940116	0.01	0.21166	79.49% (31/39)	Non-Toxin
627	W Protein	SEDPIIQELL	HLA-B*40:01	10	0.95565	0.03	0.22422	79.49% (31/39)	Non-Toxin
628	W Protein	SGSEDPIIQEL	HLA-B*40:01	11	0.854104	0.08	0.27347	79.49% (31/39)	Non-Toxin
629	W Protein	SPQKRLPM	HLA-B*07:02	8	0.831128	0.07	-0.224	74.36% (29/39)	Non-Toxin
630	W Protein	SPQKRLPM	HLA-B*08:01	8	0.612801	0.09	-0.224	74.36% (29/39)	Non-Toxin

#	Protein	Peptide	Allele	Length	Score	Percentile rank	Immunogenicity	Conservancy	Toxicity
631	W Protein	SPQKRLPML	HLA-B*07:02	9	0.972555	0.02	-0.3266	74.36% (29/39)	Non-Toxin
632	W Protein	SPQKRLPML	HLA-B*08:01	9	0.897326	0.02	-0.3266	74.36% (29/39)	Non-Toxin
633	W Protein	SPVIAEHYY	HLA-B*35:01	9	0.967986	0.02	0.30481	100.00% (39/39)	Non-Toxin
634	W Protein	SPVIAEHYY	HLA-B*53:01	9	0.694604	0.06	0.30481	100.00% (39/39)	Non-Toxin
635	W Protein	SQQGKDAQPLY	HLA-B*15:01	11	0.87658	0.03	-0.27868	79.49% (31/39)	Non-Toxin
636	W Protein	SQQGKDAQPLY	HLA-A*30:02	11	0.650536	0.08	-0.27868	79.49% (31/39)	Non-Toxin
637	W Protein	SSIQQPSIK	HLA-A*11:01	9	0.782261	0.09	-0.25846	100.00% (39/39)	Non-Toxin
638	W Protein	SSSEVIVGI	HLA-A*68:02	9	0.833703	0.04	0.26717	97.44% (38/39)	Non-Toxin
639	W Protein	STRDSLQIK	HLA-A*30:01	9	0.935075	0.01	-0.15242	71.79% (28/39)	Non-Toxin
640	W Protein	STRDSLQIK	HLA-A*11:01	9	0.789457	0.08	-0.15242	71.79% (28/39)	Non-Toxin
641	W Protein	STSPTDGTIGK	HLA-A*11:01	11	0.936819	0.01	0.1962	100.00% (39/39)	Non-Toxin
642	W Protein	TPMPKSRGI	HLA-B*07:02	9	0.892711	0.05	-0.37041	100.00% (39/39)	Non-Toxin
643	W Protein	TPMPKSRGI	HLA-B*51:01	9	0.709065	0.07	-0.37041	100.00% (39/39)	Non-Toxin
644	W Protein	VPAKDSPVI	HLA-B*51:01	9	0.838774	0.03	-0.32367	74.36% (29/39)	Non-Toxin
645	W Protein	VSDAKVLSY	HLA-A*01:01	9	0.996544	0.01	-0.23059	76.92% (30/39)	Non-Toxin
646	W Protein	VSDAKVLSY	HLA-A*30:02	9	0.751839	0.04	-0.23059	76.92% (30/39)	Non-Toxin
647	W Protein	YGFTSSPERGW	HLA-B*57:01	11	0.915052	0.09	-0.09821	100.00% (39/39)	Non-Toxin
648	Fusion Protein	AIGPPVFTDK	HLA-A*11:01	10	0.883408	0.03	0.18516	97.30% (36/37)	Non-Toxin
649	Fusion Protein	AIGPPVFTDK	HLA-A*03:01	10	0.860799	0.05	0.18516	97.30% (36/37)	Non-Toxin
650	Fusion Protein	ALYEAMKNA	HLA-A*02:03	9	0.941496	0.02	-0.21343	100.00% (37/37)	Non-Toxin
651	Fusion Protein	ALYEAMKNA	HLA-A*02:01	9	0.790189	0.09	-0.21343	100.00% (37/37)	Non-Toxin
652	Fusion Protein	ALYEAMKNA	HLA-A*02:06	9	0.713829	0.11	-0.21343	100.00% (37/37)	Non-Toxin
653	Fusion Protein	AQITAGVAL	HLA-B*15:01	9	0.809091	0.06	0.20996	97.30% (36/37)	Non-Toxin
654	Fusion Protein	AQITAGVALY	HLA-B*15:01	10	0.964206	0.01	0.21876	97.30% (36/37)	Non-Toxin
655	Fusion Protein	AQITAGVALY	HLA-A*30:02	10	0.598327	0.11	0.21876	97.30% (36/37)	Non-Toxin
656	Fusion Protein	AYIQELLPVSF	HLA-A*23:01	11	0.869392	0.03	-0.06972	100.00% (37/37)	Non-Toxin
657	Fusion Protein	AYIQELLPVSF	HLA-A*24:02	11	0.86702	0.04	-0.06972	100.00% (37/37)	Non-Toxin
658	Fusion Protein	DISSQISSM	HLA-A*26:01	9	0.75497	0.05	-0.44397	97.30% (36/37)	Non-Toxin
659	Fusion Protein	DPVSNSMTI	HLA-B*51:01	9	0.836921	0.03	-0.44062	97.30% (36/37)	Non-Toxin
660	Fusion Protein	DSITGQIIY	HLA-A*26:01	9	0.644882	0.07	0.1963	100.00% (37/37)	Non-Toxin
661	Fusion Protein	DSITGQIIY	HLA-B*35:01	9	0.728264	0.11	0.1963	100.00% (37/37)	Non-Toxin
662	Fusion Protein	DSITGQIIYV	HLA-A*68:02	10	0.642853	0.11	0.2379	100.00% (37/37)	Non-Toxin
663	Fusion Protein	DTVNPSLISM	HLA-A*26:01	10	0.635248	0.07	-0.15979	100.00% (37/37)	Non-Toxin
664	Fusion Protein	DYATPMTNNMR	HLA-A*33:01	11	0.638953	0.09	-0.20639	97.30% (36/37)	Non-Toxin
665	Fusion Protein	ELVVSSHVPR	HLA-A*68:01	10	0.920002	0.06	-0.20845	100.00% (37/37)	Non-Toxin
666	Fusion Protein	ESDSITGQIIY	HLA-A*01:01	11	0.964183	0.01	0.12217	100.00% (37/37)	Non-Toxin
667	Fusion Protein	ESTNEAVVKL	HLA-A*68:02	10	0.71031	0.08	0.08939	97.30% (36/37)	Non-Toxin
668	Fusion Protein	ETAEKTVY	HLA-A*26:01	8	0.556129	0.09	-0.02517	100.00% (37/37)	Non-Toxin
669	Fusion Protein	ETAEKTVYV	HLA-A*68:02	9	0.994144	0.01	-0.02733	100.00% (37/37)	Non-Toxin
670	Fusion Protein	ETAEKTVYV	HLA-A*26:01	9	0.644069	0.07	-0.02733	100.00% (37/37)	Non-Toxin
671	Fusion Protein	ETAEKTVYVL	HLA-A*68:02	10	0.902807	0.02	0.00111	100.00% (37/37)	Non-Toxin
672	Fusion Protein	ETLLRTLGY	HLA-A*26:01	9	0.85457	0.03	0.08262	97.30% (36/37)	Non-Toxin
673	Fusion Protein	EWISIVPNF	HLA-A*23:01	9	0.872759	0.03	0.03205	97.30% (36/37)	Non-Toxin
674	Fusion Protein	EWISIVPNF	HLA-A*24:02	9	0.836855	0.04	0.03205	97.30% (36/37)	Non-Toxin
675	Fusion Protein	FALSNGVLF	HLA-B*35:01	9	0.806076	0.08	-0.11611	100.00% (37/37)	Non-Toxin
676	Fusion Protein	FALSNGVLF	HLA-B*53:01	9	0.587275	0.09	-0.11611	100.00% (37/37)	Non-Toxin
677	Fusion Protein	FILVRNTL	HLA-B*08:01	8	0.715157	0.06	0.11501	100.00% (37/37)	Non-Toxin
678	Fusion Protein	FPILTEIQQAY	HLA-B*35:01	11	0.988425	0.01	0.113	100.00% (37/37)	Non-Toxin
679	Fusion Protein	FPILTEIQQAY	HLA-B*53:01	11	0.738909	0.05	0.113	100.00% (37/37)	Non-Toxin

#	Protein	Peptide	Allele	Length	Score	Percentile rank	Immunogenicity	Conservancy	Toxicity
680	Fusion Protein	GLVKGVTRK	HLA-A*03:01	9	0.811953	0.08	-0.06874	43.24% (16/37)	Non-Toxin
681	Fusion Protein	GQIIYVDLSSY	HLA-B*15:01	11	0.850268	0.04	-0.0114	45.95% (17/37)	Non-Toxin
682	Fusion Protein	IIYVDLSSY	HLA-B*15:01	9	0.841065	0.05	-0.18478	45.95% (17/37)	Non-Toxin
683	Fusion Protein	IIYVDLSSY	HLA-A*30:02	9	0.6073	0.1	-0.18478	45.95% (17/37)	Non-Toxin
684	Fusion Protein	IIYVDLSSYY	HLA-A*30:02	10	0.577055	0.11	-0.24637	45.95% (17/37)	Non-Toxin
685	Fusion Protein	ILTEIQQAY	HLA-B*15:01	9	0.920345	0.02	0.05901	100.00% (37/37)	Non-Toxin
686	Fusion Protein	ILTEIQQAY	HLA-A*30:02	9	0.696212	0.06	0.05901	100.00% (37/37)	Non-Toxin
687	Fusion Protein	ILTEIQQAY	HLA-B*35:01	9	0.721227	0.11	0.05901	100.00% (37/37)	Non-Toxin
688	Fusion Protein	IQQAYIQEL	HLA-A*02:06	9	0.780163	0.08	0.08419	100.00% (37/37)	Non-Toxin
689	Fusion Protein	ISECSVGILHY	HLA-A*01:01	11	0.655221	0.11	0.02517	100.00% (37/37)	Non-Toxin
690	Fusion Protein	IVIKMIPNV	HLA-A*02:06	9	0.865559	0.05	-0.23266	97.30% (36/37)	Non-Toxin
691	Fusion Protein	IYKNNTHDL	HLA-A*24:02	9	0.727965	0.08	-0.00601	100.00% (37/37)	Non-Toxin
692	Fusion Protein	IYVDLSSYY	HLA-A*30:02	9	0.662808	0.07	-0.27259	45.95% (17/37)	Non-Toxin
693	Fusion Protein	KIKSNPLTK	HLA-A*03:01	9	0.968066	0.01	-0.23989	100.00% (37/37)	Non-Toxin
694	Fusion Protein	KIKSNPLTK	HLA-A*30:01	9	0.960464	0.01	-0.23989	100.00% (37/37)	Non-Toxin
695	Fusion Protein	KIKSNPLTK	HLA-A*11:01	9	0.792886	0.08	-0.23989	100.00% (37/37)	Non-Toxin
696	Fusion Protein	KLSKIGLVK	HLA-A*03:01	9	0.949033	0.02	-0.09444	100.00% (37/37)	Non-Toxin
697	Fusion Protein	KLSKIGLVK	HLA-A*30:01	9	0.540892	0.11	-0.09444	100.00% (37/37)	Non-Toxin
698	Fusion Protein	KMIPNVSNM	HLA-B*15:01	9	0.760931	0.09	-0.0788	97.30% (36/37)	Non-Toxin
699	Fusion Protein	KMIPNVSNM	HLA-A*02:03	9	0.704273	0.1	-0.0788	97.30% (36/37)	Non-Toxin
700	Fusion Protein	KMIPNVSNM	HLA-A*32:01	9	0.498504	0.1	-0.0788	97.30% (36/37)	Non-Toxin
701	Fusion Protein	KYKIKSNPLTK	HLA-A*30:01	11	0.710571	0.04	-0.3106	100.00% (37/37)	Non-Toxin
702	Fusion Protein	KYKIKSNPLTK	HLA-A*03:01	11	0.785606	0.1	-0.3106	100.00% (37/37)	Non-Toxin
703	Fusion Protein	KYLSDLLF	HLA-A*23:01	8	0.641251	0.1	-0.16827	97.30% (36/37)	Non-Toxin
704	Fusion Protein	KYLSDLLFVF	HLA-A*23:01	10	0.918982	0.02	-0.04679	97.30% (36/37)	Non-Toxin
705	Fusion Protein	KYLSDLLFVF	HLA-A*24:02	10	0.905914	0.02	-0.04679	97.30% (36/37)	Non-Toxin
706	Fusion Protein	LLDTVNPSL	HLA-A*02:01	9	0.952173	0.02	-0.02565	100.00% (37/37)	Non-Toxin
707	Fusion Protein	LLDTVNPSL	HLA-A*02:06	9	0.841892	0.06	-0.02565	100.00% (37/37)	Non-Toxin
708	Fusion Protein	LQDPVSNSM	HLA-A*02:06	9	0.736395	0.1	-0.22161	97.30% (36/37)	Non-Toxin
709	Fusion Protein	LSLDLALSK	HLA-A*11:01	9	0.76376	0.1	-0.06127	97.30% (36/37)	Non-Toxin
710	Fusion Protein	LSLDLALSKY	HLA-A*01:01	10	0.912099	0.03	-0.23004	97.30% (36/37)	Non-Toxin
711	Fusion Protein	LTEIQQAY	HLA-A*01:01	8	0.72202	0.09	-0.0224	100.00% (37/37)	Non-Toxin
712	Fusion Protein	LTKDIVIKM	HLA-A*26:01	9	0.60023	0.08	0.1071	100.00% (37/37)	Non-Toxin
713	Fusion Protein	LVKGVTRKY	HLA-A*26:01	9	0.677122	0.06	-0.04148	43.24% (16/37)	Non-Toxin
714	Fusion Protein	LVKGVTRKY	HLA-B*15:01	9	0.784744	0.08	-0.04148	43.24% (16/37)	Non-Toxin
715	Fusion Protein	LVKGVTRKY	HLA-A*30:02	9	0.647528	0.08	-0.04148	43.24% (16/37)	Non-Toxin
716	Fusion Protein	MTIQAISQA	HLA-A*68:02	9	0.811518	0.05	-0.11728	97.30% (36/37)	Non-Toxin
717	Fusion Protein	MTIQAISQAF	HLA-A*26:01	10	0.525524	0.11	-0.13629	97.30% (36/37)	Non-Toxin
718	Fusion Protein	NPLTKDIVI	HLA-B*51:01	9	0.709989	0.07	-0.01722	100.00% (37/37)	Non-Toxin
719	Fusion Protein	NTYSRLEDR	HLA-A*33:01	9	0.686379	0.08	-0.03025	100.00% (37/37)	Non-Toxin
720	Fusion Protein	NVIISLGKY	HLA-A*26:01	9	0.85858	0.03	-0.09182	100.00% (37/37)	Non-Toxin
721	Fusion Protein	PTSSGDLYY	HLA-A*01:01	9	0.90589	0.03	-0.17781	100.00% (37/37)	Non-Toxin
722	Fusion Protein	QETAEKTVY	HLA-B*44:02	9	0.911123	0.02	0.00335	100.00% (37/37)	Non-Toxin
723	Fusion Protein	QETAEKTVY	HLA-B*44:03	9	0.944413	0.03	0.00335	100.00% (37/37)	Non-Toxin
724	Fusion Protein	QETAEKTVYV	HLA-A*68:02	10	0.760356	0.06	0.00869	100.00% (37/37)	Non-Toxin
725	Fusion Protein	QITAGVALY	HLA-A*26:01	9	0.636128	0.07	0.15037	97.30% (36/37)	Non-Toxin
726	Fusion Protein	RFALSNGVLF	HLA-A*23:01	10	0.697575	0.08	-0.1056	100.00% (37/37)	Non-Toxin
727	Fusion Protein	RFALSNGVLF	HLA-A*24:02	10	0.723486	0.09	-0.1056	100.00% (37/37)	Non-Toxin
728	Fusion Protein	RLAGVIMAGV	HLA-A*02:03	10	0.694341	0.1	0.10412	100.00% (37/37)	Non-Toxin

#	Protein	Peptide	Allele	Length	Score	Percentile rank	Immunogenicity	Conservancy	Toxicity
729	Fusion Protein	RLLDTVNPSL	HLA-A*02:01	10	0.879786	0.04	-0.01539	100.00% (37/37)	Non-Toxin
730	Fusion Protein	RLNGILTPI	HLA-A*02:03	9	0.781955	0.06	0.17744	100.00% (37/37)	Non-Toxin
731	Fusion Protein	RLNGILTPI	HLA-A*32:01	9	0.53261	0.09	0.17744	100.00% (37/37)	Non-Toxin
732	Fusion Protein	RLNGILTPIK	HLA-A*03:01	10	0.909081	0.03	0.25574	100.00% (37/37)	Non-Toxin
733	Fusion Protein	RPTSSGDLYY	HLA-A*01:01	10	0.759856	0.08	-0.27261	100.00% (37/37)	Non-Toxin
734	Fusion Protein	RVRPTSSGDLY	HLA-A*30:02	11	0.778439	0.03	-0.23462	100.00% (37/37)	Non-Toxin
735	Fusion Protein	RVRPTSSGDLY	HLA-A*30:01	11	0.531437	0.11	-0.23462	100.00% (37/37)	Non-Toxin
736	Fusion Protein	SECSVGILHY	HLA-B*44:03	10	0.909429	0.04	0.02405	100.00% (37/37)	Non-Toxin
737	Fusion Protein	SECSVGILHY	HLA-B*44:02	10	0.824313	0.05	0.02405	100.00% (37/37)	Non-Toxin
738	Fusion Protein	SEWISIVPNF	HLA-B*44:03	10	0.779422	0.09	0.20004	97.30% (36/37)	Non-Toxin
739	Fusion Protein	SITGQIIYV	HLA-A*02:06	9	0.884539	0.05	0.16934	100.00% (37/37)	Non-Toxin
740	Fusion Protein	SITGQIIYV	HLA-A*02:01	9	0.817011	0.07	0.16934	100.00% (37/37)	Non-Toxin
741	Fusion Protein	SITGQIIYV	HLA-A*02:03	9	0.743961	0.08	0.16934	100.00% (37/37)	Non-Toxin
742	Fusion Protein	SITGQIIYV	HLA-A*68:02	9	0.703353	0.08	0.16934	100.00% (37/37)	Non-Toxin
743	Fusion Protein	SIVPNFILV	HLA-A*02:06	9	0.911247	0.03	0.21198	100.00% (37/37)	Non-Toxin
744	Fusion Protein	SIVPNFILV	HLA-A*68:02	9	0.769957	0.06	0.21198	100.00% (37/37)	Non-Toxin
745	Fusion Protein	SIVPNFILV	HLA-A*02:03	9	0.672035	0.11	0.21198	100.00% (37/37)	Non-Toxin
746	Fusion Protein	SLDLALSKY	HLA-A*01:01	9	0.938569	0.02	-0.24192	97.30% (36/37)	Non-Toxin
747	Fusion Protein	SLGKYLGSV	HLA-A*02:03	9	0.747405	0.07	-0.2881	100.00% (37/37)	Non-Toxin
748	Fusion Protein	SMLSMIILY	HLA-A*30:02	9	0.674773	0.06	-0.10995	100.00% (37/37)	Non-Toxin
749	Fusion Protein	SSMNQSLQQSK	HLA-A*11:01	11	0.844997	0.05	-0.65167	100.00% (37/37)	Non-Toxin
750	Fusion Protein	SSYYIIVRV	HLA-A*68:02	9	0.837314	0.04	0.31504	45.95% (17/37)	Non-Toxin
751	Fusion Protein	STNEAVVKL	HLA-A*68:02	9	0.826345	0.04	0.08445	97.30% (36/37)	Non-Toxin
752	Fusion Protein	SVMENYKTR	HLA-A*68:01	9	0.905726	0.07	-0.12535	100.00% (37/37)	Non-Toxin
753	Fusion Protein	SVMENYKTR	HLA-A*31:01	9	0.769175	0.08	-0.12535	100.00% (37/37)	Non-Toxin
754	Fusion Protein	SVMENYKTR	HLA-A*33:01	9	0.610846	0.1	-0.12535	100.00% (37/37)	Non-Toxin
755	Fusion Protein	SYYIIVRVY	HLA-A*30:02	9	0.709825	0.05	0.36898	45.95% (17/37)	Non-Toxin
756	Fusion Protein	SYYIIVRVYF	HLA-A*23:01	10	0.623632	0.1	0.38392	45.95% (17/37)	Non-Toxin
757	Fusion Protein	TAAQITAGV	HLA-A*68:02	9	0.962136	0.01	0.1151	97.30% (36/37)	Non-Toxin
758	Fusion Protein	TELSLDLAL	HLA-B*40:01	9	0.9368	0.05	-0.14649	97.30% (36/37)	Non-Toxin
759	Fusion Protein	TPIKGALEI	HLA-B*51:01	9	0.83588	0.03	-0.05483	100.00% (37/37)	Non-Toxin
760	Fusion Protein	TPIKGALEI	HLA-B*53:01	9	0.61083	0.08	-0.05483	100.00% (37/37)	Non-Toxin
761	Fusion Protein	TPIKGALEIY	HLA-B*35:01	10	0.920784	0.03	0.04912	100.00% (37/37)	Non-Toxin
762	Fusion Protein	TVNPSLISM	HLA-A*26:01	9	0.712661	0.06	-0.16914	100.00% (37/37)	Non-Toxin
763	Fusion Protein	TVNPSLISM	HLA-A*68:02	9	0.633604	0.11	-0.16914	100.00% (37/37)	Non-Toxin
764	Fusion Protein	VLGNVIISL	HLA-A*02:01	9	0.879121	0.04	0.18563	100.00% (37/37)	Non-Toxin
765	Fusion Protein	VPNFILVRNTL	HLA-B*07:02	11	0.74277	0.11	0.34064	100.00% (37/37)	Non-Toxin
766	Fusion Protein	VPRFALSNGVL	HLA-B*07:02	11	0.834371	0.07	0.04743	100.00% (37/37)	Non-Toxin
767	Fusion Protein	VRPTSSGDLYY	HLA-A*01:01	11	0.811377	0.06	-0.24438	100.00% (37/37)	Non-Toxin
768	Fusion Protein	VSFNNDNSEW	HLA-B*58:01	10	0.944867	0.04	-0.04042	97.30% (36/37)	Non-Toxin
769	Fusion Protein	VSFNNDNSEW	HLA-B*57:01	10	0.946468	0.06	-0.04042	97.30% (36/37)	Non-Toxin
770	Fusion Protein	VVSSHVPRF	HLA-A*32:01	9	0.603745	0.06	-0.12893	100.00% (37/37)	Non-Toxin
771	Fusion Protein	VYFPILTEI	HLA-A*24:02	9	0.978204	0.01	0.23726	100.00% (37/37)	Non-Toxin
772	Fusion Protein	VYFPILTEI	HLA-A*23:01	9	0.978018	0.01	0.23726	100.00% (37/37)	Non-Toxin
773	Fusion Protein	YATPMTNNM	HLA-B*35:01	9	0.776901	0.09	-0.14226	97.30% (36/37)	Non-Toxin
774	Fusion Protein	YEKLSKIGL	HLA-B*40:01	9	0.858409	0.08	-0.31314	100.00% (37/37)	Non-Toxin
775	Fusion Protein	YVDLSSYY	HLA-A*01:01	8	0.865485	0.05	-0.32391	45.95% (17/37)	Non-Toxin
776	Fusion Protein	YYIIVRVYF	HLA-A*23:01	9	0.888305	0.03	0.29872	100.00% (37/37)	Non-Toxin
777	Fusion Protein	YYIIVRVYF	HLA-A*24:02	9	0.856049	0.04	0.29872	100.00% (37/37)	Non-Toxin

#	Protein	Peptide	Allele	Length	Score	Percentile rank	Immunogenicity	Conservancy	Toxicity
778	Polymerase	AEFFSFFRTF	HLA-B*44:03	10	0.823382	0.07	0.28526	100.00% (10/10)	Non-Toxin
779	Polymerase	AEFFSFFRTF	HLA-B*44:02	10	0.727453	0.09	0.28526	100.00% (10/10)	Non-Toxin
780	Polymerase	AELPIPEY	HLA-B*44:03	8	0.874582	0.05	0.1889	100.00% (10/10)	Non-Toxin
781	Polymerase	AELPIPEY	HLA-B*44:02	8	0.731635	0.08	0.1889	100.00% (10/10)	Non-Toxin
782	Polymerase	AEQDETVISL	HLA-B*40:01	10	0.981355	0.01	0.17454	100.00% (10/10)	Non-Toxin
783	Polymerase	AGRLFAKMTYK	HLA-A*03:01	11	0.811549	0.08	-0.18696	100.00% (10/10)	Non-Toxin
784	Polymerase	ALIASGVGK	HLA-A*03:01	9	0.883642	0.04	0.00801	100.00% (10/10)	Non-Toxin
785	Polymerase	ALIASGVGKY	HLA-B*15:01	10	0.778333	0.08	-0.10407	100.00% (10/10)	Non-Toxin
786	Polymerase	ALSHPRVFK	HLA-A*03:01	9	0.971332	0.01	0.12001	100.00% (10/10)	Non-Toxin
787	Polymerase	ALSHPRVFK	HLA-A*11:01	9	0.875731	0.04	0.12001	100.00% (10/10)	Non-Toxin
788	Polymerase	ALSHPRVFK	HLA-A*30:01	9	0.666133	0.06	0.12001	100.00% (10/10)	Non-Toxin
789	Polymerase	ALSHPRVFKR	HLA-A*31:01	10	0.787573	0.07	0.03011	100.00% (10/10)	Non-Toxin
790	Polymerase	ALSPIKDEW	HLA-A*32:01	9	0.662995	0.05	-0.06104	100.00% (10/10)	Non-Toxin
791	Polymerase	ALSPIKDEW	HLA-B*58:01	9	0.871421	0.08	-0.06104	100.00% (10/10)	Non-Toxin
792	Polymerase	ALYLGQSISIK	HLA-A*03:01	11	0.813961	0.07	-0.18984	100.00% (10/10)	Non-Toxin
793	Polymerase	APIGGFNYL	HLA-B*07:02	9	0.907381	0.04	0.21288	100.00% (10/10)	Non-Toxin
794	Polymerase	APIMKAHAI	HLA-B*08:01	9	0.854209	0.03	-0.25651	100.00% (10/10)	Non-Toxin
795	Polymerase	APIMKAHAI	HLA-B*07:02	9	0.928737	0.04	-0.25651	100.00% (10/10)	Non-Toxin
796	Polymerase	ASKHIIRLK	HLA-A*30:01	9	0.884537	0.01	0.25463	100.00% (10/10)	Non-Toxin
797	Polymerase	AYPECNNILF	HLA-A*24:02	10	0.872057	0.04	0.1381	90.00% (9/10)	Non-Toxin
798	Polymerase	AYPECNNILF	HLA-A*23:01	10	0.758397	0.06	0.1381	90.00% (9/10)	Non-Toxin
799	Polymerase	DAELPIPEY	HLA-B*35:01	9	0.904508	0.04	0.18496	100.00% (10/10)	Non-Toxin
800	Polymerase	DALEAMVGR	HLA-A*68:01	9	0.924229	0.06	0.02459	100.00% (10/10)	Non-Toxin
801	Polymerase	DALEAMVGR	HLA-A*33:01	9	0.726337	0.06	0.02459	100.00% (10/10)	Non-Toxin
802	Polymerase	DEHELLKTL	HLA-B*44:03	9	0.820297	0.07	-0.06931	100.00% (10/10)	Non-Toxin
803	Polymerase	DEHELLKTLF	HLA-B*44:03	10	0.767646	0.1	-0.08707	100.00% (10/10)	Non-Toxin
804	Polymerase	DGLLVSKI	HLA-B*51:01	8	0.700667	0.07	-0.31229	100.00% (10/10)	Non-Toxin
805	Polymerase	DIGSDINTLR	HLA-A*68:01	10	0.911368	0.07	0.01592	100.00% (10/10)	Non-Toxin
806	Polymerase	DIKLGNVKR	HLA-A*33:01	9	0.843853	0.02	-0.14541	100.00% (10/10)	Non-Toxin
807	Polymerase	DIKYQPLISR	HLA-A*33:01	10	0.812091	0.03	-0.1921	100.00% (10/10)	Non-Toxin
808	Polymerase	DIKYQPLISR	HLA-A*68:01	10	0.902947	0.07	-0.1921	100.00% (10/10)	Non-Toxin
809	Polymerase	DLKKYYQI	HLA-B*08:01	8	0.579563	0.1	-0.39184	100.00% (10/10)	Non-Toxin
810	Polymerase	DPYNMLEYV	HLA-B*51:01	9	0.882661	0.02	-0.10681	100.00% (10/10)	Non-Toxin
811	Polymerase	DSQSITKTI	HLA-B*51:01	9	0.758219	0.05	-0.19725	100.00% (10/10)	Non-Toxin
812	Polymerase	DSYEYIINR	HLA-A*68:01	9	0.978937	0.01	0.32977	100.00% (10/10)	Non-Toxin
813	Polymerase	DSYEYIINR	HLA-A*33:01	9	0.915796	0.01	0.32977	100.00% (10/10)	Non-Toxin
814	Polymerase	DVLKAITPV	HLA-A*68:02	9	0.681954	0.09	-0.03094	100.00% (10/10)	Non-Toxin
815	Polymerase	EALIASGVGKY	HLA-A*26:01	11	0.749702	0.05	-0.01822	100.00% (10/10)	Non-Toxin
816	Polymerase	EAMVGRYI	HLA-B*51:01	8	0.641085	0.1	0.06314	100.00% (10/10)	Non-Toxin
817	Polymerase	EAWYLASQR	HLA-A*68:01	9	0.946822	0.04	-0.11309	100.00% (10/10)	Non-Toxin
818	Polymerase	EAWYLASQR	HLA-A*33:01	9	0.761082	0.05	-0.11309	100.00% (10/10)	Non-Toxin
819	Polymerase	EFFSFFRTF	HLA-A*23:01	9	0.831873	0.04	0.16209	100.00% (10/10)	Non-Toxin
820	Polymerase	EFFSFFRTF	HLA-A*24:02	9	0.747448	0.07	0.16209	100.00% (10/10)	Non-Toxin
821	Polymerase	EFNPHNHYK	HLA-A*33:01	9	0.712964	0.06	0.03729	100.00% (10/10)	Non-Toxin
822	Polymerase	EICAKQAQLY	HLA-A*26:01	10	0.622105	0.07	-0.36834	100.00% (10/10)	Non-Toxin
823	Polymerase	EIINIHECR	HLA-A*68:01	9	0.954696	0.03	0.24974	100.00% (10/10)	Non-Toxin
824	Polymerase	EIINIHECR	HLA-A*33:01	9	0.785381	0.03	0.24974	100.00% (10/10)	Non-Toxin
825	Polymerase	EIYGLPGFF	HLA-A*26:01	9	0.832767	0.04	0.10866	100.00% (10/10)	Non-Toxin
826	Polymerase	ELKLDSDLSMY	HLA-A*01:01	11	0.729152	0.09	-0.45172	100.00% (10/10)	Non-Toxin

#	Protein	Peptide	Allele	Length	Score	Percentile rank	Immunogenicity	Conservancy	Toxicity
827	Polymerase	ELKLFPSEY	HLA-A*26:01	9	0.712676	0.06	-0.05872	100.00% (10/10)	Non-Toxin
828	Polymerase	ESMAIFAER	HLA-A*68:01	9	0.986103	0.01	0.31369	100.00% (10/10)	Non-Toxin
829	Polymerase	ESMAIFAER	HLA-A*33:01	9	0.920339	0.01	0.31369	100.00% (10/10)	Non-Toxin
830	Polymerase	ETDDYNGIY	HLA-A*01:01	9	0.979774	0.01	0.12619	100.00% (10/10)	Non-Toxin
831	Polymerase	ETDDYNGIY	HLA-A*26:01	9	0.534209	0.1	0.12619	100.00% (10/10)	Non-Toxin
832	Polymerase	ETDDYNGIYHL	HLA-A*68:02	11	0.683156	0.09	0.19368	100.00% (10/10)	Non-Toxin
833	Polymerase	ETIISTHLF	HLA-A*26:01	9	0.944687	0.01	0.07338	100.00% (10/10)	Non-Toxin
834	Polymerase	ETIISTHLFIY	HLA-A*26:01	11	0.612948	0.08	0.25144	90.00% (9/10)	Non-Toxin
835	Polymerase	ETKQAGRLF	HLA-A*26:01	9	0.698682	0.06	-0.07936	100.00% (10/10)	Non-Toxin
836	Polymerase	ETNTRIAAI	HLA-A*68:02	9	0.762928	0.06	0.26852	100.00% (10/10)	Non-Toxin
837	Polymerase	ETTVKSDIKY	HLA-A*26:01	10	0.619616	0.07	-0.30976	100.00% (10/10)	Non-Toxin
838	Polymerase	ETTWIGNLDSY	HLA-A*26:01	11	0.797646	0.04	0.30341	100.00% (10/10)	Non-Toxin
839	Polymerase	EVDNNHLIY	HLA-A*01:01	9	0.982598	0.01	0.09324	100.00% (10/10)	Non-Toxin
840	Polymerase	EVDNNHLIY	HLA-A*26:01	9	0.562828	0.09	0.09324	100.00% (10/10)	Non-Toxin
841	Polymerase	EVNDQGITSV	HLA-A*68:02	10	0.871511	0.03	0.0018	100.00% (10/10)	Non-Toxin
842	Polymerase	EVPDALEAM	HLA-A*26:01	9	0.823839	0.04	0.15374	100.00% (10/10)	Non-Toxin
843	Polymerase	EVPDALEAMV	HLA-A*68:02	10	0.680838	0.09	0.07069	100.00% (10/10)	Non-Toxin
844	Polymerase	EYIINRTAGR	HLA-A*33:01	10	0.678265	0.08	0.31058	100.00% (10/10)	Non-Toxin
845	Polymerase	FAERLDEIY	HLA-A*01:01	9	0.732737	0.09	0.25692	100.00% (10/10)	Non-Toxin
846	Polymerase	FALYLGQSI	HLA-B*51:01	9	0.699321	0.07	-0.18064	100.00% (10/10)	Non-Toxin
847	Polymerase	FEKLSQNLL	HLA-B*40:01	9	0.885711	0.07	-0.36324	100.00% (10/10)	Non-Toxin
848	Polymerase	FIDDLLSIL	HLA-A*02:06	9	0.820214	0.07	-0.05358	100.00% (10/10)	Non-Toxin
849	Polymerase	FIDDLLSIL	HLA-A*02:01	9	0.753755	0.1	-0.05358	100.00% (10/10)	Non-Toxin
850	Polymerase	FIYSKKIHY	HLA-B*15:01	9	0.878613	0.03	-0.44945	90.00% (9/10)	Non-Toxin
851	Polymerase	FIYSKKIHY	HLA-A*26:01	9	0.676405	0.06	-0.44945	90.00% (9/10)	Non-Toxin
852	Polymerase	FIYSKKIHY	HLA-A*30:02	9	0.647328	0.08	-0.44945	90.00% (9/10)	Non-Toxin
853	Polymerase	FLMDRRVIL	HLA-B*08:01	9	0.946245	0.01	0.17704	100.00% (10/10)	Non-Toxin
854	Polymerase	FLMDRRVIL	HLA-A*02:03	9	0.817851	0.05	0.17704	100.00% (10/10)	Non-Toxin
855	Polymerase	FLMDRRVIL	HLA-A*02:01	9	0.851636	0.06	0.17704	100.00% (10/10)	Non-Toxin
856	Polymerase	FNKVKSAL	HLA-B*08:01	8	0.82196	0.03	-0.36117	100.00% (10/10)	Non-Toxin
857	Polymerase	FPISRLFNM	HLA-B*35:01	9	0.92836	0.03	0.01171	100.00% (10/10)	Non-Toxin
858	Polymerase	FPISRLFNM	HLA-B*51:01	9	0.769648	0.05	0.01171	100.00% (10/10)	Non-Toxin
859	Polymerase	FPISRLFNM	HLA-B*08:01	9	0.744343	0.05	0.01171	100.00% (10/10)	Non-Toxin
860	Polymerase	FPISRLFNM	HLA-B*53:01	9	0.716075	0.05	0.01171	100.00% (10/10)	Non-Toxin
861	Polymerase	FPISRLFNMY	HLA-B*35:01	10	0.81414	0.07	-0.08153	100.00% (10/10)	Non-Toxin
862	Polymerase	FPLWSTEEL	HLA-B*35:01	9	0.919156	0.03	0.23773	100.00% (10/10)	Non-Toxin
863	Polymerase	FPLWSTEEL	HLA-B*53:01	9	0.742916	0.05	0.23773	100.00% (10/10)	Non-Toxin
864	Polymerase	FPLWSTEEL	HLA-B*51:01	9	0.677867	0.08	0.23773	100.00% (10/10)	Non-Toxin
865	Polymerase	FPVMGNRI	HLA-B*51:01	8	0.813447	0.04	-0.09271	100.00% (10/10)	Non-Toxin
866	Polymerase	FPVMGNRIY	HLA-B*35:01	9	0.988335	0.01	-0.01495	100.00% (10/10)	Non-Toxin
867	Polymerase	FPVMGNRIY	HLA-B*53:01	9	0.709672	0.06	-0.01495	100.00% (10/10)	Non-Toxin
868	Polymerase	FTDQKIRSM	HLA-B*08:01	9	0.590356	0.1	-0.24706	100.00% (10/10)	Non-Toxin
869	Polymerase	FYASLTYLR	HLA-A*33:01	9	0.814289	0.03	-0.13763	100.00% (10/10)	Non-Toxin
870	Polymerase	GEGSGSMML	HLA-B*40:01	9	0.943271	0.04	-0.529	100.00% (10/10)	Non-Toxin
871	Polymerase	GEKVDTNLIY	HLA-B*44:03	10	0.761614	0.1	0.09325	100.00% (10/10)	Non-Toxin
872	Polymerase	GFFNWMHKR	HLA-A*31:01	9	0.864797	0.04	-0.01681	100.00% (10/10)	Non-Toxin
873	Polymerase	GFFNWMHKR	HLA-A*33:01	9	0.652583	0.09	-0.01681	100.00% (10/10)	Non-Toxin
874	Polymerase	GIEGYSQKTW	HLA-B*44:02	10	0.795517	0.05	-0.36646	100.00% (10/10)	Non-Toxin
875	Polymerase	GLIDPLFPV	HLA-A*02:01	9	0.976885	0.01	0.1366	100.00% (10/10)	Non-Toxin

#	Protein	Peptide	Allele	Length	Score	Percentile rank	Immunogenicity	Conservancy	Toxicity
876	Polymerase	GLIDPLFPV	HLA-A*02:06	9	0.971103	0.01	0.1366	100.00% (10/10)	Non-Toxin
877	Polymerase	GLIDPLFPV	HLA-A*02:03	9	0.942868	0.02	0.1366	100.00% (10/10)	Non-Toxin
878	Polymerase	GLIRSGLRK	HLA-A*03:01	9	0.863636	0.05	-0.01304	100.00% (10/10)	Non-Toxin
879	Polymerase	GLLDTTKGL	HLA-A*02:01	9	0.791648	0.08	-0.06914	100.00% (10/10)	Non-Toxin
880	Polymerase	GLLDTTKGL	HLA-A*02:03	9	0.689945	0.1	-0.06914	100.00% (10/10)	Non-Toxin
881	Polymerase	GLQPKLVSR	HLA-A*31:01	9	0.875448	0.04	-0.33102	90.00% (9/10)	Non-Toxin
882	Polymerase	GRLFAKMTYK	HLA-A*03:01	10	0.832314	0.06	-0.1924	100.00% (10/10)	Non-Toxin
883	Polymerase	GSGSMMLLY	HLA-A*30:02	9	0.658464	0.07	-0.50761	100.00% (10/10)	Non-Toxin
884	Polymerase	GYSQKTWTI	HLA-A*24:02	9	0.876993	0.04	-0.1341	100.00% (10/10)	Non-Toxin
885	Polymerase	GYSQKTWTI	HLA-A*23:01	9	0.718316	0.08	-0.1341	100.00% (10/10)	Non-Toxin
886	Polymerase	HIKNNIRRK	HLA-A*30:01	9	0.60974	0.07	0.11639	100.00% (10/10)	Non-Toxin
887	Polymerase	HLDSPIVSGK	HLA-A*03:01	10	0.800942	0.08	-0.12143	100.00% (10/10)	Non-Toxin
888	Polymerase	HLLAEFFSF	HLA-A*32:01	9	0.699547	0.04	0.24561	100.00% (10/10)	Non-Toxin
889	Polymerase	HMLADKVLEY	HLA-B*15:01	10	0.796862	0.07	-0.06463	100.00% (10/10)	Non-Toxin
890	Polymerase	HMRDKSTEI	HLA-B*08:01	9	0.893986	0.02	-0.23535	100.00% (10/10)	Non-Toxin
891	Polymerase	HPNLPYKV	HLA-B*51:01	8	0.661363	0.09	-0.20954	100.00% (10/10)	Non-Toxin
892	Polymerase	HTEFNPHNHY	HLA-A*01:01	10	0.963169	0.01	0.17709	100.00% (10/10)	Non-Toxin
893	Polymerase	HTEFNPHNHY	HLA-B*44:03	10	0.915897	0.03	0.17709	100.00% (10/10)	Non-Toxin
894	Polymerase	HTEFNPHNHY	HLA-A*30:02	10	0.754132	0.04	0.17709	100.00% (10/10)	Non-Toxin
895	Polymerase	HTEFNPHNHY	HLA-B*44:02	10	0.820447	0.05	0.17709	100.00% (10/10)	Non-Toxin
896	Polymerase	HTEFNPHNHY	HLA-A*26:01	10	0.67573	0.06	0.17709	100.00% (10/10)	Non-Toxin
897	Polymerase	HTYKVLSNA	HLA-A*68:02	9	0.768275	0.06	-0.33184	100.00% (10/10)	Non-Toxin
898	Polymerase	HVDTSSRSW	HLA-B*58:01	9	0.910284	0.06	-0.32355	100.00% (10/10)	Non-Toxin
899	Polymerase	HVDTSSRSW	HLA-B*53:01	9	0.689189	0.06	-0.32355	100.00% (10/10)	Non-Toxin
900	Polymerase	IATVYTWAY	HLA-B*35:01	9	0.771632	0.09	0.29688	100.00% (10/10)	Non-Toxin
901	Polymerase	IAYPECNNI	HLA-B*51:01	9	0.742431	0.06	0.02515	90.00% (9/10)	Non-Toxin
902	Polymerase	IEGYSQKTW	HLA-B*44:02	9	0.930451	0.02	-0.42218	100.00% (10/10)	Non-Toxin
903	Polymerase	IEGYSQKTW	HLA-B*44:03	9	0.934111	0.03	-0.42218	100.00% (10/10)	Non-Toxin
904	Polymerase	ILNIDNIHLL	HLA-A*02:01	10	0.814141	0.07	0.29322	100.00% (10/10)	Non-Toxin
905	Polymerase	ILNPNLICI	HLA-A*02:01	9	0.764226	0.1	0.05082	100.00% (10/10)	Non-Toxin
906	Polymerase	ILRNSPNPMLK	HLA-A*03:01	11	0.833016	0.06	-0.33303	100.00% (10/10)	Non-Toxin
907	Polymerase	ILVEHSHLI	HLA-A*02:03	9	0.917118	0.03	0.01074	100.00% (10/10)	Non-Toxin
908	Polymerase	ILVEHSHLI	HLA-A*02:01	9	0.908684	0.03	0.01074	100.00% (10/10)	Non-Toxin
909	Polymerase	ILVEHSHLI	HLA-A*02:06	9	0.784133	0.08	0.01074	100.00% (10/10)	Non-Toxin
910	Polymerase	IMKKSFKAY	HLA-B*15:01	9	0.934486	0.01	-0.49704	100.00% (10/10)	Non-Toxin
911	Polymerase	IMLNEAMNY	HLA-A*30:02	9	0.656456	0.08	-0.02776	100.00% (10/10)	Non-Toxin
912	Polymerase	IMTESVLQK	HLA-A*03:01	9	0.902445	0.03	-0.08593	100.00% (10/10)	Non-Toxin
913	Polymerase	IPAPIGGFNY	HLA-B*35:01	10	0.916029	0.04	0.29106	100.00% (10/10)	Non-Toxin
914	Polymerase	IPAPIGGFNY	HLA-B*53:01	10	0.72893	0.05	0.29106	100.00% (10/10)	Non-Toxin
915	Polymerase	IPFLFLSAY	HLA-B*35:01	9	0.962059	0.02	0.01364	100.00% (10/10)	Non-Toxin
916	Polymerase	IPGQRELKL	HLA-B*08:01	9	0.699375	0.07	-0.09627	100.00% (10/10)	Non-Toxin
917	Polymerase	IPGQRELKL	HLA-B*07:02	9	0.780531	0.09	-0.09627	100.00% (10/10)	Non-Toxin
918	Polymerase	IQFDCFMEL	HLA-A*02:06	9	0.819612	0.07	0.02832	100.00% (10/10)	Non-Toxin
919	Polymerase	ISRLFNMYR	HLA-A*31:01	9	0.796194	0.07	-0.03681	100.00% (10/10)	Non-Toxin
920	Polymerase	ITSQGMTSK	HLA-A*11:01	9	0.858372	0.04	-0.36646	100.00% (10/10)	Non-Toxin
921	Polymerase	ITSQGMTSK	HLA-A*03:01	9	0.790546	0.09	-0.36646	100.00% (10/10)	Non-Toxin
922	Polymerase	IYGLPGFFNW	HLA-A*23:01	10	0.894838	0.02	0.22726	100.00% (10/10)	Non-Toxin
923	Polymerase	IYGLPGFFNW	HLA-A*24:02	10	0.873025	0.04	0.22726	100.00% (10/10)	Non-Toxin
924	Polymerase	KALNLSPLIQR	HLA-A*31:01	11	0.846067	0.05	-0.15861	100.00% (10/10)	Non-Toxin

#	Protein	Peptide	Allele	Length	Score	Percentile rank	Immunogenicity	Conservancy	Toxicity
925	Polymerase	KALSPIKDEW	HLA-B*57:01	10	0.991766	0.01	-0.17705	100.00% (10/10)	Non-Toxin
926	Polymerase	KALSPIKDEW	HLA-B*58:01	10	0.983327	0.01	-0.17705	100.00% (10/10)	Non-Toxin
927	Polymerase	KAYNIISKK	HLA-A*03:01	9	0.975367	0.01	-0.01845	100.00% (10/10)	Non-Toxin
928	Polymerase	KAYNIISKK	HLA-A*11:01	9	0.94852	0.01	-0.01845	100.00% (10/10)	Non-Toxin
929	Polymerase	KAYNIISKK	HLA-A*30:01	9	0.878783	0.01	-0.01845	100.00% (10/10)	Non-Toxin
930	Polymerase	KEICAKQAQLY	HLA-B*44:03	11	0.847747	0.06	-0.36316	100.00% (10/10)	Non-Toxin
931	Polymerase	KEICAKQAQLY	HLA-B*44:02	11	0.771193	0.07	-0.36316	100.00% (10/10)	Non-Toxin
932	Polymerase	KETKQAGRLF	HLA-B*44:02	10	0.698587	0.1	-0.21	100.00% (10/10)	Non-Toxin
933	Polymerase	KFAGSVLNR	HLA-A*31:01	9	0.861168	0.04	-0.08858	100.00% (10/10)	Non-Toxin
934	Polymerase	KFKDTKSSELY	HLA-A*30:02	11	0.653727	0.08	-0.45869	100.00% (10/10)	Non-Toxin
935	Polymerase	KGLIRSGLRK	HLA-A*03:01	10	0.833249	0.06	0.0724	100.00% (10/10)	Non-Toxin
936	Polymerase	KIWWKIIGY	HLA-A*32:01	9	0.723173	0.03	0.34219	100.00% (10/10)	Non-Toxin
937	Polymerase	KIWWKIIGY	HLA-A*30:02	9	0.6134	0.1	0.34219	100.00% (10/10)	Non-Toxin
938	Polymerase	KKYYQIDQPF	HLA-A*23:01	10	0.636779	0.1	-0.07148	100.00% (10/10)	Non-Toxin
939	Polymerase	KLDSDLSMY	HLA-A*01:01	9	0.923022	0.03	-0.39033	100.00% (10/10)	Non-Toxin
940	Polymerase	KLDSDLSMY	HLA-A*30:02	9	0.715847	0.05	-0.39033	100.00% (10/10)	Non-Toxin
941	Polymerase	KLFPSEYSI	HLA-A*32:01	9	0.910285	0.01	-0.13979	100.00% (10/10)	Non-Toxin
942	Polymerase	KLFPSEYSI	HLA-A*02:01	9	0.956367	0.02	-0.13979	100.00% (10/10)	Non-Toxin
943	Polymerase	KLFPSEYSI	HLA-A*02:06	9	0.881674	0.05	-0.13979	100.00% (10/10)	Non-Toxin
944	Polymerase	KLFPSEYSI	HLA-A*02:03	9	0.832089	0.05	-0.13979	100.00% (10/10)	Non-Toxin
945	Polymerase	KLISAIEYA	HLA-A*02:01	9	0.820528	0.07	0.12245	100.00% (10/10)	Non-Toxin
946	Polymerase	KLISAIEYA	HLA-A*02:06	9	0.80208	0.07	0.12245	100.00% (10/10)	Non-Toxin
947	Polymerase	KLISAIEYA	HLA-A*02:03	9	0.775657	0.07	0.12245	100.00% (10/10)	Non-Toxin
948	Polymerase	KLKGLVVPL	HLA-A*02:03	9	0.740366	0.08	0.02052	100.00% (10/10)	Non-Toxin
949	Polymerase	KLMTKTLPK	HLA-A*03:01	9	0.974443	0.01	-0.20724	100.00% (10/10)	Non-Toxin
950	Polymerase	KLMTKTLPK	HLA-A*30:01	9	0.769015	0.02	-0.20724	100.00% (10/10)	Non-Toxin
951	Polymerase	KQAGRLFAK	HLA-A*30:01	9	0.662612	0.06	0.20842	100.00% (10/10)	Non-Toxin
952	Polymerase	KQAGRLFAK	HLA-A*03:01	9	0.817215	0.07	0.20842	100.00% (10/10)	Non-Toxin
953	Polymerase	KQAQLYFER	HLA-A*31:01	9	0.888244	0.03	0.03916	100.00% (10/10)	Non-Toxin
954	Polymerase	KQFHDDLKKY	HLA-B*15:01	10	0.880014	0.03	-0.20469	100.00% (10/10)	Non-Toxin
955	Polymerase	KQFHDDLKKY	HLA-A*30:02	10	0.743434	0.04	-0.20469	100.00% (10/10)	Non-Toxin
956	Polymerase	KQFHDDLKKYY	HLA-A*30:02	11	0.732901	0.04	-0.28421	100.00% (10/10)	Non-Toxin
957	Polymerase	KQFHDDLKKYY	HLA-B*15:01	11	0.752747	0.1	-0.28421	100.00% (10/10)	Non-Toxin
958	Polymerase	KSDKTGKKCY	HLA-A*01:01	10	0.673985	0.1	-0.5555	70.00% (7/10)	Non-Toxin
959	Polymerase	KSDKTGKKCYY	HLA-A*01:01	11	0.759146	0.08	-0.59966	70.00% (7/10)	Non-Toxin
960	Polymerase	KSFLDYHTEF	HLA-B*57:01	10	0.939633	0.07	0.16655	100.00% (10/10)	Non-Toxin
961	Polymerase	KSFLDYHTEF	HLA-B*58:01	10	0.836987	0.09	0.16655	100.00% (10/10)	Non-Toxin
962	Polymerase	KSGLQPKLVSR	HLA-A*31:01	11	0.908371	0.02	-0.40602	90.00% (9/10)	Non-Toxin
963	Polymerase	KSRELDFPLW	HLA-B*57:01	10	0.994996	0.01	0.22271	100.00% (10/10)	Non-Toxin
964	Polymerase	KSRELDFPLW	HLA-B*58:01	10	0.968245	0.02	0.22271	100.00% (10/10)	Non-Toxin
965	Polymerase	KSSELYHIK	HLA-A*30:01	9	0.729676	0.04	0.13783	100.00% (10/10)	Non-Toxin
966	Polymerase	KSSELYHIK	HLA-A*11:01	9	0.805859	0.08	0.13783	100.00% (10/10)	Non-Toxin
967	Polymerase	KTIKNITAR	HLA-A*31:01	9	0.968599	0.01	0.0008	100.00% (10/10)	Non-Toxin
968	Polymerase	KTIKNITAR	HLA-A*11:01	9	0.822354	0.06	0.0008	100.00% (10/10)	Non-Toxin
969	Polymerase	KTLPKGMQER	HLA-A*31:01	10	0.929844	0.01	-0.39632	100.00% (10/10)	Non-Toxin
970	Polymerase	KTLPKGMQERR	HLA-A*31:01	11	0.908707	0.02	-0.35706	100.00% (10/10)	Non-Toxin
971	Polymerase	KTPEDDIFIHY	HLA-A*01:01	11	0.915235	0.03	0.51137	90.00% (9/10)	Non-Toxin
972	Polymerase	KVHPNLPYK	HLA-A*03:01	9	0.983067	0.01	-0.02892	100.00% (10/10)	Non-Toxin
973	Polymerase	KVHPNLPYK	HLA-A*30:01	9	0.9816	0.01	-0.02892	100.00% (10/10)	Non-Toxin

#	Protein	Peptide	Allele	Length	Score	Percentile rank	Immunogenicity	Conservancy	Toxicity
974	Polymerase	KVHPNLPYK	HLA-A*11:01	9	0.975148	0.01	-0.02892	100.00% (10/10)	Non-Toxin
975	Polymerase	KVHPNLPYK	HLA-A*31:01	9	0.810218	0.06	-0.02892	100.00% (10/10)	Non-Toxin
976	Polymerase	KVHPNLPYKVK	HLA-A*30:01	11	0.797072	0.02	-0.18992	100.00% (10/10)	Non-Toxin
977	Polymerase	KVHPNLPYKVK	HLA-A*03:01	11	0.786951	0.1	-0.18992	100.00% (10/10)	Non-Toxin
978	Polymerase	KVIGMLQNI	HLA-A*32:01	9	0.655429	0.05	-0.20568	100.00% (10/10)	Non-Toxin
979	Polymerase	KVIGMLQNI	HLA-A*02:06	9	0.858934	0.06	-0.20568	100.00% (10/10)	Non-Toxin
980	Polymerase	KVIGMLQNITR	HLA-A*31:01	11	0.800345	0.06	-0.08839	100.00% (10/10)	Non-Toxin
981	Polymerase	KVKKEICAK	HLA-A*30:01	9	0.803901	0.02	-0.08686	100.00% (10/10)	Non-Toxin
982	Polymerase	KVKKEICAK	HLA-A*03:01	9	0.783982	0.1	-0.08686	100.00% (10/10)	Non-Toxin
983	Polymerase	KVLEYAPIMK	HLA-A*03:01	10	0.933888	0.02	0.13093	100.00% (10/10)	Non-Toxin
984	Polymerase	KVLEYAPIMK	HLA-A*11:01	10	0.908142	0.02	0.13093	100.00% (10/10)	Non-Toxin
985	Polymerase	KVREHMLADK	HLA-A*30:01	10	0.709284	0.04	0.01359	100.00% (10/10)	Non-Toxin
986	Polymerase	KVREHMLADK	HLA-A*03:01	10	0.789649	0.09	0.01359	100.00% (10/10)	Non-Toxin
987	Polymerase	KWAFEIHHRR	HLA-A*31:01	10	0.833449	0.05	0.44559	100.00% (10/10)	Non-Toxin
988	Polymerase	KWYECFLFW	HLA-A*23:01	9	0.643792	0.1	0.21629	100.00% (10/10)	Non-Toxin
989	Polymerase	KYFKENGMVK	HLA-A*30:01	10	0.576802	0.09	-0.17998	100.00% (10/10)	Non-Toxin
990	Polymerase	KYYQIDQPF	HLA-A*23:01	9	0.949346	0.01	-0.07152	100.00% (10/10)	Non-Toxin
991	Polymerase	KYYQIDQPF	HLA-A*24:02	9	0.942987	0.01	-0.07152	100.00% (10/10)	Non-Toxin
992	Polymerase	KYYQIDQPFF	HLA-A*23:01	10	0.941388	0.01	-0.01656	100.00% (10/10)	Non-Toxin
993	Polymerase	KYYQIDQPFF	HLA-A*24:02	10	0.930055	0.02	-0.01656	100.00% (10/10)	Non-Toxin
994	Polymerase	KYYQIDQPFFV	HLA-A*02:06	11	0.743572	0.09	0.0774	100.00% (10/10)	Non-Toxin
995	Polymerase	LADKVLEY	HLA-A*01:01	8	0.722731	0.09	-0.09554	100.00% (10/10)	Non-Toxin
996	Polymerase	LAKTVAQTV	HLA-B*51:01	9	0.795097	0.04	-0.02899	100.00% (10/10)	Non-Toxin
997	Polymerase	LARALRSHMW	HLA-B*57:01	10	0.923896	0.09	-0.13526	100.00% (10/10)	Non-Toxin
998	Polymerase	LETDDYNGIY	HLA-A*01:01	10	0.931807	0.02	0.15319	100.00% (10/10)	Non-Toxin
999	Polymerase	LGQSISIKW	HLA-B*58:01	9	0.932578	0.04	-0.24388	100.00% (10/10)	Non-Toxin
1000	Polymerase	LGQSISIKW	HLA-B*57:01	9	0.94388	0.07	-0.24388	100.00% (10/10)	Non-Toxin
1001	Polymerase	LIASGVGKY	HLA-A*26:01	9	0.58391	0.09	-0.17931	100.00% (10/10)	Non-Toxin
1002	Polymerase	LISRSNALW	HLA-B*58:01	9	0.894877	0.06	-0.14227	100.00% (10/10)	Non-Toxin
1003	Polymerase	LKLDSDLSMY	HLA-A*01:01	10	0.84994	0.05	-0.37344	100.00% (10/10)	Non-Toxin
1004	Polymerase	LPAHASKHI	HLA-B*51:01	9	0.765138	0.05	-0.23548	90.00% (9/10)	Non-Toxin
1005	Polymerase	LPIPEYTEV	HLA-B*51:01	9	0.976165	0.01	0.21732	100.00% (10/10)	Non-Toxin
1006	Polymerase	LPIPEYTEV	HLA-B*53:01	9	0.570883	0.09	0.21732	100.00% (10/10)	Non-Toxin
1007	Polymerase	LPSGAQRLF	HLA-B*53:01	9	0.875556	0.03	-0.05334	100.00% (10/10)	Non-Toxin
1008	Polymerase	LPSGAQRLF	HLA-B*35:01	9	0.893121	0.05	-0.05334	100.00% (10/10)	Non-Toxin
1009	Polymerase	LPYKVKKEI	HLA-B*51:01	9	0.950014	0.01	-0.5045	100.00% (10/10)	Non-Toxin
1010	Polymerase	LPYKVKKEI	HLA-B*07:02	9	0.776658	0.09	-0.5045	100.00% (10/10)	Non-Toxin
1011	Polymerase	LSHPRVFKR	HLA-A*31:01	9	0.915212	0.02	0.0614	100.00% (10/10)	Non-Toxin
1012	Polymerase	LSHPRVFKR	HLA-A*33:01	9	0.727337	0.06	0.0614	100.00% (10/10)	Non-Toxin
1013	Polymerase	LSNREVKIW	HLA-B*57:01	9	0.991806	0.01	0.0821	100.00% (10/10)	Non-Toxin
1014	Polymerase	LSNREVKIW	HLA-B*58:01	9	0.980314	0.01	0.0821	100.00% (10/10)	Non-Toxin
1015	Polymerase	LSNREVKIWW	HLA-B*57:01	10	0.95387	0.06	0.22642	100.00% (10/10)	Non-Toxin
1016	Polymerase	LTIDDCVKNW	HLA-B*57:01	10	0.965981	0.05	-0.1123	100.00% (10/10)	Non-Toxin
1017	Polymerase	LTIDDCVKNW	HLA-B*58:01	10	0.914447	0.05	-0.1123	100.00% (10/10)	Non-Toxin
1018	Polymerase	LTPEMVLMY	HLA-A*01:01	9	0.786954	0.07	-0.14695	100.00% (10/10)	Non-Toxin
1019	Polymerase	LTPEMVLMY	HLA-A*26:01	9	0.633998	0.07	-0.14695	100.00% (10/10)	Non-Toxin
1020	Polymerase	LWGLIDPLF	HLA-A*23:01	9	0.652191	0.1	0.13448	100.00% (10/10)	Non-Toxin
1021	Polymerase	LYLGQSISI	HLA-A*24:02	9	0.716974	0.09	-0.22237	100.00% (10/10)	Non-Toxin
1022	Polymerase	MLADKVLEY	HLA-B*15:01	9	0.949274	0.01	-0.08698	100.00% (10/10)	Non-Toxin

#	Protein	Peptide	Allele	Length	Score	Percentile rank	Immunogenicity	Conservancy	Toxicity
1023	Polymerase	MLADKVLEY	HLA-A*30:02	9	0.808511	0.02	-0.08698	100.00% (10/10)	Non-Toxin
1024	Polymerase	MLADKVLEY	HLA-A*01:01	9	0.844751	0.05	-0.08698	100.00% (10/10)	Non-Toxin
1025	Polymerase	MLADKVLEY	HLA-A*26:01	9	0.754185	0.05	-0.08698	100.00% (10/10)	Non-Toxin
1026	Polymerase	MLADKVLEY	HLA-B*35:01	9	0.83305	0.07	-0.08698	100.00% (10/10)	Non-Toxin
1027	Polymerase	MLADKVLEYA	HLA-A*02:03	10	0.694179	0.1	-0.06288	100.00% (10/10)	Non-Toxin
1028	Polymerase	MTESVLQKV	HLA-A*68:02	9	0.716639	0.08	-0.32797	100.00% (10/10)	Non-Toxin
1029	Polymerase	MYCDVLEGR	HLA-A*33:01	9	0.659634	0.08	0.13888	100.00% (10/10)	Non-Toxin
1030	Polymerase	NIRRKVLIL	HLA-B*08:01	9	0.94942	0.01	-0.03386	100.00% (10/10)	Non-Toxin
1031	Polymerase	NITARTILR	HLA-A*33:01	9	0.688272	0.08	0.24475	100.00% (10/10)	Non-Toxin
1032	Polymerase	NLHGKRKSL	HLA-B*08:01	9	0.949676	0.01	-0.39534	100.00% (10/10)	Non-Toxin
1033	Polymerase	NLSPLIQRY	HLA-A*30:02	9	0.779952	0.03	-0.0179	100.00% (10/10)	Non-Toxin
1034	Polymerase	NLSPLIQRY	HLA-A*26:01	9	0.642122	0.07	-0.0179	100.00% (10/10)	Non-Toxin
1035	Polymerase	NPDSTEVYL	HLA-B*53:01	9	0.584975	0.09	0.00546	100.00% (10/10)	Non-Toxin
1036	Polymerase	NPPIKTGVL	HLA-B*08:01	9	0.592548	0.1	0.00958	100.00% (10/10)	Non-Toxin
1037	Polymerase	NPQEKICVL	HLA-B*08:01	9	0.884446	0.02	-0.04295	100.00% (10/10)	Non-Toxin
1038	Polymerase	NTMYGWFFV	HLA-A*68:02	9	0.656525	0.1	0.34799	100.00% (10/10)	Non-Toxin
1039	Polymerase	NTSDLDFVIFY	HLA-A*01:01	11	0.704239	0.1	0.313	100.00% (10/10)	Non-Toxin
1040	Polymerase	NTSKHTYKV	HLA-A*68:02	9	0.869929	0.03	-0.33178	100.00% (10/10)	Non-Toxin
1041	Polymerase	NTSKSFLDY	HLA-A*01:01	9	0.836734	0.05	-0.318	100.00% (10/10)	Non-Toxin
1042	Polymerase	NVKRPTKAL	HLA-B*08:01	9	0.746773	0.05	-0.15132	100.00% (10/10)	Non-Toxin
1043	Polymerase	NVKRPTKALR	HLA-A*33:01	10	0.704769	0.07	-0.16738	100.00% (10/10)	Non-Toxin
1044	Polymerase	NYLNLSRIF	HLA-A*23:01	9	0.864851	0.03	-0.0552	100.00% (10/10)	Non-Toxin
1045	Polymerase	NYLNLSRIF	HLA-A*24:02	9	0.871082	0.04	-0.0552	100.00% (10/10)	Non-Toxin
1046	Polymerase	QESKSRELDF	HLA-B*44:02	10	0.695373	0.1	-0.28355	100.00% (10/10)	Non-Toxin
1047	Polymerase	QPFFVPTKI	HLA-B*51:01	9	0.952773	0.01	0.09232	100.00% (10/10)	Non-Toxin
1048	Polymerase	QPFFVPTKI	HLA-B*53:01	9	0.59665	0.08	0.09232	100.00% (10/10)	Non-Toxin
1049	Polymerase	QPLISRSNAL	HLA-B*07:02	10	0.91172	0.04	-0.11871	100.00% (10/10)	Non-Toxin
1050	Polymerase	QQLLISTEF	HLA-B*15:01	9	0.881393	0.03	0.05037	100.00% (10/10)	Non-Toxin
1051	Polymerase	QSISIKWAF	HLA-A*32:01	9	0.558811	0.07	0.01313	100.00% (10/10)	Non-Toxin
1052	Polymerase	QSISIKWAF	HLA-B*57:01	9	0.919057	0.09	0.01313	100.00% (10/10)	Non-Toxin
1053	Polymerase	QSISIKWAF	HLA-B*58:01	9	0.849445	0.09	0.01313	100.00% (10/10)	Non-Toxin
1054	Polymerase	QTVLEIITK	HLA-A*11:01	9	0.789251	0.08	0.36002	100.00% (10/10)	Non-Toxin
1055	Polymerase	QVDAELPIPEY	HLA-A*01:01	11	0.688061	0.1	0.29689	100.00% (10/10)	Non-Toxin
1056	Polymerase	RALRSHMW	HLA-B*57:01	8	0.913389	0.09	-0.23037	100.00% (10/10)	Non-Toxin
1057	Polymerase	RALRSHMW	HLA-B*58:01	8	0.836902	0.09	-0.23037	100.00% (10/10)	Non-Toxin
1058	Polymerase	RALRSHMWR	HLA-A*31:01	9	0.753547	0.08	-0.10095	100.00% (10/10)	Non-Toxin
1059	Polymerase	RAVIKNSQK	HLA-A*30:01	9	0.591082	0.08	-0.27607	100.00% (10/10)	Non-Toxin
1060	Polymerase	REHSSIRVPY	HLA-B*44:02	10	0.791683	0.05	-0.11039	100.00% (10/10)	Non-Toxin
1061	Polymerase	REHSSIRVPY	HLA-B*44:03	10	0.82941	0.06	-0.11039	100.00% (10/10)	Non-Toxin
1062	Polymerase	REKNGFKEVW	HLA-B*44:02	10	0.893812	0.03	-0.02389	90.00% (9/10)	Non-Toxin
1063	Polymerase	REKNGFKEVW	HLA-B*44:03	10	0.877629	0.04	-0.02389	90.00% (9/10)	Non-Toxin
1064	Polymerase	RELALGRVI	HLA-B*40:01	9	0.80287	0.1	0.12467	100.00% (10/10)	Non-Toxin
1065	Polymerase	RELKLFPSEY	HLA-B*44:03	10	0.80047	0.08	-0.20896	100.00% (10/10)	Non-Toxin
1066	Polymerase	RELKLFPSEY	HLA-B*44:02	10	0.701365	0.1	-0.20896	100.00% (10/10)	Non-Toxin
1067	Polymerase	RIATVYTW	HLA-A*32:01	8	0.515644	0.1	0.12124	100.00% (10/10)	Non-Toxin
1068	Polymerase	RIYNIVSMI	HLA-A*32:01	9	0.63658	0.05	-0.08147	100.00% (10/10)	Non-Toxin
1069	Polymerase	RLETDDYNGIY	HLA-A*01:01	11	0.841886	0.05	0.21143	100.00% (10/10)	Non-Toxin
1070	Polymerase	RLFAKMTYK	HLA-A*03:01	9	0.992137	0.01	-0.26733	100.00% (10/10)	Non-Toxin
1071	Polymerase	RLFAKMTYK	HLA-A*30:01	9	0.857769	0.01	-0.26733	100.00% (10/10)	Non-Toxin

#	Protein	Peptide	Allele	Length	Score	Percentile rank	Immunogenicity	Conservancy	Toxicity
1072	Polymerase	RLFAKMTYK	HLA-A*11:01	9	0.869853	0.04	-0.26733	100.00% (10/10)	Non-Toxin
1073	Polymerase	RLFAKMTYK	HLA-A*31:01	9	0.82979	0.05	-0.26733	100.00% (10/10)	Non-Toxin
1074	Polymerase	RLFNMYRSY	HLA-A*32:01	9	0.853832	0.01	-0.19597	100.00% (10/10)	Non-Toxin
1075	Polymerase	RLFNMYRSY	HLA-A*30:02	9	0.835929	0.01	-0.19597	100.00% (10/10)	Non-Toxin
1076	Polymerase	RLFNMYRSY	HLA-B*15:01	9	0.902758	0.02	-0.19597	100.00% (10/10)	Non-Toxin
1077	Polymerase	RLFNMYRSYF	HLA-A*32:01	10	0.538636	0.08	-0.23617	100.00% (10/10)	Non-Toxin
1078	Polymerase	RLNLHGKRK	HLA-A*03:01	9	0.840472	0.05	-0.10162	100.00% (10/10)	Non-Toxin
1079	Polymerase	RLRDKSTQF	HLA-B*15:01	9	0.964699	0.01	-0.36153	100.00% (10/10)	Non-Toxin
1080	Polymerase	RLRDKSTQF	HLA-A*32:01	9	0.781152	0.02	-0.36153	100.00% (10/10)	Non-Toxin
1081	Polymerase	RLRDKSTQF	HLA-B*08:01	9	0.714697	0.06	-0.36153	100.00% (10/10)	Non-Toxin
1082	Polymerase	RLRDKSTQFK	HLA-A*03:01	10	0.951989	0.01	-0.3248	100.00% (10/10)	Non-Toxin
1083	Polymerase	RLRDKSTQFK	HLA-A*30:01	10	0.799443	0.02	-0.3248	100.00% (10/10)	Non-Toxin
1084	Polymerase	RMMMETTVK	HLA-A*03:01	9	0.794141	0.09	-0.04278	100.00% (10/10)	Non-Toxin
1085	Polymerase	RPRGRHTM	HLA-B*07:02	8	0.977882	0.02	0.16451	100.00% (10/10)	Non-Toxin
1086	Polymerase	RPRGRHTM	HLA-B*08:01	8	0.652974	0.08	0.16451	100.00% (10/10)	Non-Toxin
1087	Polymerase	RPRGRHTMV	HLA-B*07:02	9	0.977509	0.02	0.06191	100.00% (10/10)	Non-Toxin
1088	Polymerase	RPRGRHTMV	HLA-B*08:01	9	0.613908	0.09	0.06191	100.00% (10/10)	Non-Toxin
1089	Polymerase	RPRGRHTMVDL	HLA-B*07:02	11	0.946034	0.03	0.0531	100.00% (10/10)	Non-Toxin
1090	Polymerase	RPSVENHKY	HLA-B*35:01	9	0.814186	0.07	-0.01945	100.00% (10/10)	Non-Toxin
1091	Polymerase	RPTKALRSA	HLA-B*07:02	9	0.751886	0.1	-0.22972	100.00% (10/10)	Non-Toxin
1092	Polymerase	RPTKALRSAI	HLA-B*07:02	10	0.927416	0.04	-0.24514	100.00% (10/10)	Non-Toxin
1093	Polymerase	RSKLMTKTLPK	HLA-A*30:01	11	0.584701	0.09	-0.40366	100.00% (10/10)	Non-Toxin
1094	Polymerase	RTFGHPILEAK	HLA-A*03:01	11	0.977686	0.01	0.31932	100.00% (10/10)	Non-Toxin
1095	Polymerase	RTFGHPILEAK	HLA-A*11:01	11	0.962112	0.01	0.31932	100.00% (10/10)	Non-Toxin
1096	Polymerase	RTFGHPILEAK	HLA-A*30:01	11	0.834546	0.01	0.31932	100.00% (10/10)	Non-Toxin
1097	Polymerase	RYEDNTGTKF	HLA-A*24:02	10	0.74196	0.07	0.02498	100.00% (10/10)	Non-Toxin
1098	Polymerase	RYKCSNTSK	HLA-A*30:01	9	0.731337	0.04	-0.35534	100.00% (10/10)	Non-Toxin
1099	Polymerase	RYLPSGAQR	HLA-A*31:01	9	0.915958	0.02	-0.17862	100.00% (10/10)	Non-Toxin
1100	Polymerase	RYLPSGAQRL	HLA-A*24:02	10	0.864861	0.04	-0.17355	100.00% (10/10)	Non-Toxin
1101	Polymerase	RYLPSGAQRL	HLA-A*23:01	10	0.793876	0.05	-0.17355	100.00% (10/10)	Non-Toxin
1102	Polymerase	RYLPSGAQRLF	HLA-A*24:02	11	0.963175	0.01	-0.1766	100.00% (10/10)	Non-Toxin
1103	Polymerase	RYLPSGAQRLF	HLA-A*23:01	11	0.950483	0.01	-0.1766	100.00% (10/10)	Non-Toxin
1104	Polymerase	SAIEYAQLR	HLA-A*68:01	9	0.939067	0.04	0.07294	100.00% (10/10)	Non-Toxin
1105	Polymerase	SAIRIATV	HLA-B*51:01	8	0.646464	0.1	0.29447	100.00% (10/10)	Non-Toxin
1106	Polymerase	SAIRIATVY	HLA-B*35:01	9	0.96342	0.02	0.31859	100.00% (10/10)	Non-Toxin
1107	Polymerase	SAIRIATVY	HLA-B*15:01	9	0.78797	0.08	0.31859	100.00% (10/10)	Non-Toxin
1108	Polymerase	SAIRIATVY	HLA-A*30:02	9	0.60604	0.1	0.31859	100.00% (10/10)	Non-Toxin
1109	Polymerase	SAIRIATVYTW	HLA-B*57:01	11	0.950289	0.06	0.3592	100.00% (10/10)	Non-Toxin
1110	Polymerase	SAIRIATVYTW	HLA-B*58:01	11	0.850536	0.09	0.3592	100.00% (10/10)	Non-Toxin
1111	Polymerase	SASKKIFNL	HLA-B*08:01	9	0.713945	0.06	-0.2604	100.00% (10/10)	Non-Toxin
1112	Polymerase	SAYETNTRI	HLA-B*51:01	9	0.755332	0.05	0.19426	100.00% (10/10)	Non-Toxin
1113	Polymerase	SFLDYHTEF	HLA-A*23:01	9	0.914152	0.02	0.13683	100.00% (10/10)	Non-Toxin
1114	Polymerase	SFLDYHTEF	HLA-A*24:02	9	0.908613	0.02	0.13683	100.00% (10/10)	Non-Toxin
1115	Polymerase	SGLQPKLVSR	HLA-A*31:01	10	0.771077	0.08	-0.41322	90.00% (9/10)	Non-Toxin
1116	Polymerase	SIMTESVLQK	HLA-A*11:01	10	0.90894	0.02	-0.11972	100.00% (10/10)	Non-Toxin
1117	Polymerase	SIMTESVLQK	HLA-A*03:01	10	0.896155	0.03	-0.11972	100.00% (10/10)	Non-Toxin
1118	Polymerase	SITSQGMTSK	HLA-A*11:01	10	0.826196	0.06	-0.46287	100.00% (10/10)	Non-Toxin
1119	Polymerase	SLREDIITSK	HLA-A*03:01	10	0.900281	0.03	0.33013	100.00% (10/10)	Non-Toxin
1120	Polymerase	SLYILRQSK	HLA-A*03:01	9	0.893911	0.03	-0.02378	100.00% (10/10)	Non-Toxin

#	Protein	Peptide	Allele	Length	Score	Percentile rank	Immunogenicity	Conservancy	Toxicity
1121	Polymerase	SLYILRQSK	HLA-A*30:01	9	0.6114	0.07	-0.02378	100.00% (10/10)	Non-Toxin
1122	Polymerase	SMFIDDLLSI	HLA-A*02:03	10	0.689384	0.1	0.09866	100.00% (10/10)	Non-Toxin
1123	Polymerase	SMIEPLVLA	HLA-A*02:03	9	0.895288	0.03	0.15107	100.00% (10/10)	Non-Toxin
1124	Polymerase	SMIEPLVLA	HLA-A*02:06	9	0.898162	0.04	0.15107	100.00% (10/10)	Non-Toxin
1125	Polymerase	SMIEPLVLA	HLA-A*02:01	9	0.886276	0.04	0.15107	100.00% (10/10)	Non-Toxin
1126	Polymerase	SMNTKSASK	HLA-A*03:01	9	0.886178	0.04	-0.39241	100.00% (10/10)	Non-Toxin
1127	Polymerase	SMNTKSASK	HLA-A*30:01	9	0.577742	0.09	-0.39241	100.00% (10/10)	Non-Toxin
1128	Polymerase	SMYMKDKAL	HLA-B*08:01	9	0.83085	0.03	-0.52616	100.00% (10/10)	Non-Toxin
1129	Polymerase	SPIKDEWDSVY	HLA-B*35:01	11	0.891279	0.05	0.06638	100.00% (10/10)	Non-Toxin
1130	Polymerase	SPIVSGKLI	HLA-B*51:01	9	0.650402	0.1	-0.23294	100.00% (10/10)	Non-Toxin
1131	Polymerase	SPNPMLKGL	HLA-B*07:02	9	0.96201	0.03	-0.3569	100.00% (10/10)	Non-Toxin
1132	Polymerase	SPSHHLEPY	HLA-B*35:01	9	0.926763	0.03	0.07793	100.00% (10/10)	Non-Toxin
1133	Polymerase	SQNLLVTSY	HLA-B*15:01	9	0.988794	0.01	-0.0491	100.00% (10/10)	Non-Toxin
1134	Polymerase	SQNLLVTSY	HLA-A*30:02	9	0.843801	0.01	-0.0491	100.00% (10/10)	Non-Toxin
1135	Polymerase	STEFSINETL	HLA-B*40:01	10	0.956627	0.02	0.21989	100.00% (10/10)	Non-Toxin
1136	Polymerase	STHLFIYSK	HLA-A*11:01	9	0.957062	0.01	0.13884	90.00% (9/10)	Non-Toxin
1137	Polymerase	STHLFIYSK	HLA-A*30:01	9	0.710044	0.04	0.13884	90.00% (9/10)	Non-Toxin
1138	Polymerase	STLGQSISF	HLA-A*32:01	9	0.807368	0.02	-0.22237	100.00% (10/10)	Non-Toxin
1139	Polymerase	STLGQSISFY	HLA-A*30:02	10	0.639465	0.08	-0.18934	100.00% (10/10)	Non-Toxin
1140	Polymerase	STSNNLSHR	HLA-A*68:01	9	0.922277	0.06	-0.19767	100.00% (10/10)	Non-Toxin
1141	Polymerase	SVLEGKFRL	HLA-A*02:06	9	0.833349	0.06	0.05619	100.00% (10/10)	Non-Toxin
1142	Polymerase	SVLEGKFRLR	HLA-A*31:01	10	0.797535	0.06	0.06755	100.00% (10/10)	Non-Toxin
1143	Polymerase	SVLNRVSRY	HLA-A*30:02	9	0.832181	0.02	-0.03023	100.00% (10/10)	Non-Toxin
1144	Polymerase	SVLNRVSRY	HLA-A*26:01	9	0.790896	0.04	-0.03023	100.00% (10/10)	Non-Toxin
1145	Polymerase	SVYPREVLSY	HLA-A*30:02	10	0.87422	0.01	0.06838	100.00% (10/10)	Non-Toxin
1146	Polymerase	SVYPREVLSY	HLA-B*15:01	10	0.905934	0.02	0.06838	100.00% (10/10)	Non-Toxin
1147	Polymerase	SVYPREVLSY	HLA-A*03:01	10	0.881268	0.04	0.06838	100.00% (10/10)	Non-Toxin
1148	Polymerase	SVYPREVLSY	HLA-A*26:01	10	0.811638	0.04	0.06838	100.00% (10/10)	Non-Toxin
1149	Polymerase	SVYPREVLSY	HLA-A*11:01	10	0.800093	0.08	0.06838	100.00% (10/10)	Non-Toxin
1150	Polymerase	SVYPREVLSY	HLA-A*32:01	10	0.539727	0.08	0.06838	100.00% (10/10)	Non-Toxin
1151	Polymerase	SYFGLVLVCF	HLA-A*23:01	10	0.744381	0.07	0.0944	100.00% (10/10)	Non-Toxin
1152	Polymerase	SYMIYLMNW	HLA-A*23:01	9	0.822194	0.04	-0.0891	100.00% (10/10)	Non-Toxin
1153	Polymerase	SYMIYLMNW	HLA-A*24:02	9	0.804882	0.06	-0.0891	100.00% (10/10)	Non-Toxin
1154	Polymerase	TEFNPHNHY	HLA-B*44:03	9	0.982412	0.01	0.06458	100.00% (10/10)	Non-Toxin
1155	Polymerase	TEFNPHNHY	HLA-B*44:02	9	0.965017	0.01	0.06458	100.00% (10/10)	Non-Toxin
1156	Polymerase	TEFSINETL	HLA-B*40:01	9	0.992205	0.01	0.10222	100.00% (10/10)	Non-Toxin
1157	Polymerase	TEFSINETL	HLA-B*44:03	9	0.774084	0.09	0.10222	100.00% (10/10)	Non-Toxin
1158	Polymerase	TEFSINETLTL	HLA-B*40:01	11	0.922328	0.06	0.14219	100.00% (10/10)	Non-Toxin
1159	Polymerase	TEIILNPNL	HLA-B*40:01	9	0.912165	0.06	0.14709	100.00% (10/10)	Non-Toxin
1160	Polymerase	TESVLQKVM	HLA-B*44:03	9	0.799231	0.08	-0.28988	100.00% (10/10)	Non-Toxin
1161	Polymerase	TESVLQKVM	HLA-B*44:02	9	0.720928	0.09	-0.28988	100.00% (10/10)	Non-Toxin
1162	Polymerase	TETIISTHL	HLA-B*40:01	9	0.944836	0.04	0.17205	100.00% (10/10)	Non-Toxin
1163	Polymerase	TETIISTHLF	HLA-B*44:03	10	0.750623	0.1	0.17238	100.00% (10/10)	Non-Toxin
1164	Polymerase	TETIISTHLF	HLA-B*44:02	10	0.696101	0.1	0.17238	100.00% (10/10)	Non-Toxin
1165	Polymerase	TEVDNNHLI	HLA-B*40:01	9	0.848758	0.09	0.04415	100.00% (10/10)	Non-Toxin
1166	Polymerase	TEVDNNHLI	HLA-B*44:03	9	0.773184	0.09	0.04415	100.00% (10/10)	Non-Toxin
1167	Polymerase	TEVDNNHLIY	HLA-B*44:03	10	0.921608	0.03	0.12197	100.00% (10/10)	Non-Toxin
1168	Polymerase	TEVDNNHLIY	HLA-A*01:01	10	0.912782	0.03	0.12197	100.00% (10/10)	Non-Toxin
1169	Polymerase	TEVDNNHLIY	HLA-B*44:02	10	0.812143	0.05	0.12197	100.00% (10/10)	Non-Toxin

#	Protein	Peptide	Allele	Length	Score	Percentile rank	Immunogenicity	Conservancy	Toxicity
1170	Polymerase	TGKLKGLVV	HLA-B*08:01	9	0.616068	0.09	-0.2445	100.00% (10/10)	Non-Toxin
1171	Polymerase	TIATIPFLF	HLA-A*32:01	9	0.530401	0.09	0.26324	100.00% (10/10)	Non-Toxin
1172	Polymerase	TIMNCVTKK	HLA-A*11:01	9	0.771159	0.09	-0.17039	90.00% (9/10)	Non-Toxin
1173	Polymerase	TKTIKNITAR	HLA-A*68:01	10	0.894531	0.08	0.11112	100.00% (10/10)	Non-Toxin
1174	Polymerase	TLGQSISFY	HLA-A*30:02	9	0.614879	0.1	-0.2126	100.00% (10/10)	Non-Toxin
1175	Polymerase	TLVDETRSA	HLA-A*02:03	9	0.6851	0.1	0.11678	100.00% (10/10)	Non-Toxin
1176	Polymerase	TPEDDIFIHY	HLA-B*35:01	10	0.834987	0.06	0.44744	100.00% (10/10)	Non-Toxin
1177	Polymerase	TPEDDIFIHY	HLA-B*53:01	10	0.63585	0.07	0.44744	100.00% (10/10)	Non-Toxin
1178	Polymerase	TPEDDIFIHY	HLA-B*44:03	10	0.796685	0.08	0.44744	100.00% (10/10)	Non-Toxin
1179	Polymerase	TPGFPISRL	HLA-B*07:02	9	0.83853	0.07	0.1339	100.00% (10/10)	Non-Toxin
1180	Polymerase	TPGFPISRLF	HLA-B*53:01	10	0.721765	0.05	0.12907	100.00% (10/10)	Non-Toxin
1181	Polymerase	TPVSTSNNL	HLA-B*07:02	9	0.815954	0.08	-0.28024	100.00% (10/10)	Non-Toxin
1182	Polymerase	TSDLDFVIFY	HLA-A*01:01	10	0.973912	0.01	0.35122	100.00% (10/10)	Non-Toxin
1183	Polymerase	TTVKSDIKY	HLA-A*26:01	9	0.64814	0.07	-0.3575	100.00% (10/10)	Non-Toxin
1184	Polymerase	TTVKSDIKY	HLA-A*01:01	9	0.69926	0.1	-0.3575	100.00% (10/10)	Non-Toxin
1185	Polymerase	TTWIGNLDSY	HLA-A*26:01	10	0.554422	0.09	0.14414	100.00% (10/10)	Non-Toxin
1186	Polymerase	TVAQTVLEI	HLA-A*68:02	9	0.671157	0.09	0.02194	100.00% (10/10)	Non-Toxin
1187	Polymerase	TVLEIITKA	HLA-A*02:06	9	0.880375	0.05	0.25879	100.00% (10/10)	Non-Toxin
1188	Polymerase	TVLEIITKA	HLA-A*68:02	9	0.740554	0.07	0.25879	100.00% (10/10)	Non-Toxin
1189	Polymerase	VIFYASLTY	HLA-A*30:02	9	0.722156	0.04	-0.07003	100.00% (10/10)	Non-Toxin
1190	Polymerase	VIFYASLTY	HLA-B*15:01	9	0.829588	0.05	-0.07003	100.00% (10/10)	Non-Toxin
1191	Polymerase	VIKNSQKPK	HLA-A*30:01	9	0.606311	0.08	-0.53513	100.00% (10/10)	Non-Toxin
1192	Polymerase	VMMEDGLLV	HLA-A*02:01	9	0.871134	0.05	0.08141	100.00% (10/10)	Non-Toxin
1193	Polymerase	VMMEDGLLV	HLA-A*02:06	9	0.746373	0.09	0.08141	100.00% (10/10)	Non-Toxin
1194	Polymerase	VMMEDGLLV	HLA-A*02:03	9	0.702884	0.1	0.08141	100.00% (10/10)	Non-Toxin
1195	Polymerase	VSNTSKHTY	HLA-A*30:02	9	0.859883	0.01	-0.27716	100.00% (10/10)	Non-Toxin
1196	Polymerase	VSNTSKHTY	HLA-A*01:01	9	0.736085	0.09	-0.27716	100.00% (10/10)	Non-Toxin
1197	Polymerase	VYPREVLSY	HLA-A*24:02	9	0.720043	0.09	0.07882	100.00% (10/10)	Non-Toxin
1198	Polymerase	VYPREVLSY	HLA-A*23:01	9	0.647236	0.1	0.07882	100.00% (10/10)	Non-Toxin
1199	Polymerase	WAFEIHHRR	HLA-A*33:01	9	0.829405	0.02	0.35634	100.00% (10/10)	Non-Toxin
1200	Polymerase	WAFEIHHRR	HLA-A*68:01	9	0.939272	0.04	0.35634	100.00% (10/10)	Non-Toxin
1201	Polymerase	YEDNTGTKF	HLA-B*44:02	9	0.709644	0.09	-0.02285	100.00% (10/10)	Non-Toxin
1202	Polymerase	YEDNTGTKF	HLA-B*40:01	9	0.805676	0.1	-0.02285	100.00% (10/10)	Non-Toxin
1203	Polymerase	YEDNTGTKF	HLA-B*44:03	9	0.769024	0.1	-0.02285	100.00% (10/10)	Non-Toxin
1204	Polymerase	YGLPGFFNW	HLA-B*58:01	9	0.949328	0.04	0.22346	100.00% (10/10)	Non-Toxin
1205	Polymerase	YGLPGFFNW	HLA-B*57:01	9	0.963054	0.05	0.22346	100.00% (10/10)	Non-Toxin
1206	Polymerase	YHTEFNPHNHY	HLA-B*44:03	11	0.790711	0.08	0.25414	100.00% (10/10)	Non-Toxin
1207	Polymerase	YLASQRVNI	HLA-A*02:03	9	0.772641	0.07	-0.18679	100.00% (10/10)	Non-Toxin
1208	Polymerase	YLEDEQFNV	HLA-A*02:01	9	0.965747	0.02	0.1383	100.00% (10/10)	Non-Toxin
1209	Polymerase	YLEDEQFNV	HLA-A*02:06	9	0.902384	0.04	0.1383	100.00% (10/10)	Non-Toxin
1210	Polymerase	YLEDEQFNVSY	HLA-A*01:01	11	0.869685	0.05	0.08561	100.00% (10/10)	Non-Toxin
1211	Polymerase	YLPSGAQRL	HLA-A*02:03	9	0.722541	0.09	-0.16776	100.00% (10/10)	Non-Toxin
1212	Polymerase	YLPSGAQRLF	HLA-B*53:01	10	0.636284	0.07	-0.17081	100.00% (10/10)	Non-Toxin
1213	Polymerase	YLTPEMVLM	HLA-A*02:01	9	0.749817	0.1	-0.038	100.00% (10/10)	Non-Toxin
1214	Polymerase	YPECNNILF	HLA-B*53:01	9	0.766098	0.04	0.0717	90.00% (9/10)	Non-Toxin
1215	Polymerase	YPECNNILF	HLA-B*35:01	9	0.85598	0.06	0.0717	90.00% (9/10)	Non-Toxin
1216	Polymerase	YPKGGIEGY	HLA-B*35:01	9	0.981469	0.01	0.22668	100.00% (10/10)	Non-Toxin
1217	Polymerase	YPKGGIEGY	HLA-B*53:01	9	0.663676	0.07	0.22668	100.00% (10/10)	Non-Toxin
1218	Polymerase	YPKGGIEGY	HLA-A*26:01	9	0.537597	0.1	0.22668	100.00% (10/10)	Non-Toxin

#	Protein	Peptide	Allele	Length	Score	Percentile rank	Immunogenicity	Conservancy	Toxicity
1219	Polymerase	YPREVLSY	HLA-B*35:01	8	0.924899	0.03	0.00769	100.00% (10/10)	Non-Toxin
1220	Polymerase	YPVLERTRI	HLA-B*51:01	9	0.790006	0.04	0.21146	100.00% (10/10)	Non-Toxin
1221	Polymerase	YQIDQPFFV	HLA-A*02:06	9	0.99605	0.01	0.10948	100.00% (10/10)	Non-Toxin
1222	Polymerase	YQIDQPFFV	HLA-A*02:01	9	0.9837	0.01	0.10948	100.00% (10/10)	Non-Toxin
1223	Polymerase	YQIDQPFFV	HLA-A*02:03	9	0.894139	0.03	0.10948	100.00% (10/10)	Non-Toxin
1224	Polymerase	YSNPDSTEVY	HLA-A*01:01	10	0.945	0.02	-0.0076	100.00% (10/10)	Non-Toxin
1225	Polymerase	YTEVDNNHLIY	HLA-A*01:01	11	0.989212	0.01	0.18189	100.00% (10/10)	Non-Toxin
1226	Polymerase	YTPGFPISR	HLA-A*68:01	9	0.941523	0.04	0.14972	100.00% (10/10)	Non-Toxin
1227	Polymerase	YTPGFPISRLF	HLA-B*57:01	11	0.914005	0.09	0.14477	100.00% (10/10)	Non-Toxin
1228	Polymerase	YYLTPEMVL	HLA-A*24:02	9	0.743055	0.07	-0.00517	100.00% (10/10)	Non-Toxin
1229	Polymerase	YYLTPEMVL	HLA-A*23:01	9	0.688171	0.09	-0.00517	100.00% (10/10)	Non-Toxin
1230	Polymerase	YYQIDQPFF	HLA-A*24:02	9	0.973241	0.01	0.06792	100.00% (10/10)	Non-Toxin
1231	Polymerase	YYQIDQPFF	HLA-A*23:01	9	0.944604	0.01	0.06792	100.00% (10/10)	Non-Toxin
1232	Polymerase	YYQIDQPFFV	HLA-A*02:06	10	0.811423	0.07	0.16188	100.00% (10/10)	Non-Toxin

Table S3: High Percentile Ranking HTL epitopes-HLA allele pairs screened from entire proteome of SARS-CoV-2 by the "MHC-II Binding Predictions" tool of IEDB. These epitopes were further utilized to identify the potentially immunogenic multiple epitope cluster based HTL Ag-Patches from the entire proteome of the SRAS-CoV-2. The screened epitopes are in consensus with the previous studies[38].

#	Protein	Peptide	Allele	Conservancy	Toxicity	Percentile rank	Method used
1	c protein	ASILLTLFRRTKKKY	HLA-DPA1*03:01/DPB1*04:02	100.00% (60/60)	Non-Toxin	0.62	Consensus (comb.lib./smm/nn)
2	c protein	ASILLTLFRRTKKKY	HLA-DRB1*11:01	100.00% (60/60)	Non-Toxin	0.63	Consensus (smm/nn/sturniolo)
3	c protein	CLRMMEMLKEETWRI	HLA-DRB3*01:01	21.67% (13/60)	Non-Toxin	0.27	Consensus (comb.lib./smm/nn)
4	c protein	CPMLPKLQFLKKIGK	HLA-DRB1*11:01	98.33% (59/60)	Non-Toxin	0.19	Consensus (smm/nn/sturniolo)
5	c protein	CPMLPKLQFLKKIGK	HLA-DRB5*01:01	98.33% (59/60)	Non-Toxin	0.7	Consensus (smm/nn/sturniolo)
6	c protein	DDQVFNNPASKIKQK	HLA-DRB1*13:02	21.67% (13/60)	Non-Toxin	0.27	Consensus (smm/nn/sturniolo)
7	c protein	DQVFNNPASKIKQKP	HLA-DRB1*13:02	21.67% (13/60)	Non-Toxin	0.4	Consensus (smm/nn/sturniolo)
8	c protein	EETWRIYPVLLPQME	HLA-DRB1*08:02	21.67% (13/60)	Non-Toxin	0.41	Consensus (smm/nn/sturniolo)
9	c protein	EMLKEETWRIYPVLL	HLA-DRB3*01:01	21.67% (13/60)	Non-Toxin	0.72	Consensus (comb.lib./smm/nn)
10	c protein	ETWRIYPVLLPQMEL	HLA-DRB1*08:02	21.67% (13/60)	Non-Toxin	0.35	Consensus (smm/nn/sturniolo)
11	c protein	FRRTKKKYRRHTDDQ	HLA-DRB5*01:01	95.00% (57/60)	Non-Toxin	0.17	Consensus (smm/nn/sturniolo)
12	c protein	HTDDQVFNNPASKIK	HLA-DRB1*13:02	21.67% (13/60)	Non-Toxin	0.41	Consensus (smm/nn/sturniolo)
13	c protein	ILLTLFRRTKKKYRR	HLA-DRB5*01:01	100.00% (60/60)	Non-Toxin	0.17	Consensus (smm/nn/sturniolo)
14	c protein	ILLTLFRRTKKKYRR	HLA-DRB1*11:01	100.00% (60/60)	Non-Toxin	0.38	Consensus (smm/nn/sturniolo)
15	c protein	IYPVLLPQMELLERE	HLA-DPA1*03:01/DPB1*04:02	21.67% (13/60)	Non-Toxin	1.01	Consensus (comb.lib./smm/nn)
16	c protein	KEETWRIYPVLLPQM	HLA-DRB1*08:02	21.67% (13/60)	Non-Toxin	0.63	Consensus (smm/nn/sturniolo)
17	c protein	KVQMTYNWTQWLQTL	HLA-DPA1*01:03/DPB1*02:01	95.00% (57/60)	Non-Toxin	0.09	Consensus (comb.lib./smm/nn)
18	c protein	KVQMTYNWTQWLQTL	HLA-DPA1*01/DPB1*04:01	95.00% (57/60)	Non-Toxin	0.87	Consensus (comb.lib./smm/nn)
19	c protein	LFRRTKKKYRRHTDD	HLA-DRB5*01:01	95.00% (57/60)	Non-Toxin	0.17	Consensus (smm/nn/sturniolo)
20	c protein	LLTLFRRTKKKYRRH	HLA-DRB5*01:01	100.00% (60/60)	Non-Toxin	0.17	Consensus (smm/nn/sturniolo)
21	c protein	LLTLFRRTKKKYRRH	HLA-DRB1*11:01	100.00% (60/60)	Non-Toxin	0.34	Consensus (smm/nn/sturniolo)
22	c protein	LQTLYTMIMEENVPD	HLA-DQA1*04:01/DQB1*04:02	100.00% (60/60)	Non-Toxin	0.22	Consensus (comb.lib./smm/nn)
23	c protein	LRMMEMLKEETWRIY	HLA-DRB3*01:01	21.67% (13/60)	Non-Toxin	0.18	Consensus (comb.lib./smm/nn)
24	c protein	LTLFRRTKKKYRRHT	HLA-DRB5*01:01	100.00% (60/60)	Non-Toxin	0.17	Consensus (smm/nn/sturniolo)
25	c protein	LTLFRRTKKKYRRHT	HLA-DRB1*11:01	100.00% (60/60)	Non-Toxin	0.52	Consensus (smm/nn/sturniolo)
26	c protein	MASILLTLFRRTKKK	HLA-DPA1*03:01/DPB1*04:02	100.00% (60/60)	Non-Toxin	0.39	Consensus (comb.lib./smm/nn)
27	c protein	MASILLTLFRRTKKK	HLA-DPA1*01:03/DPB1*02:01	100.00% (60/60)	Non-Toxin	0.82	Consensus (comb.lib./smm/nn)
28	c protein	MEMLKEETWRIYPVL	HLA-DRB3*01:01	21.67% (13/60)	Non-Toxin	0.18	Consensus (comb.lib./smm/nn)
29	c protein	MLKEETWRIYPVLLP	HLA-DRB3*01:01	21.67% (13/60)	Non-Toxin	0.16	Consensus (comb.lib./smm/nn)
30	c protein	MLPKLQFLKKIGKLI	HLA-DRB1*11:01	98.33% (59/60)	Non-Toxin	0.19	Consensus (smm/nn/sturniolo)
31	c protein	MLPKLQFLKKIGKLI	HLA-DRB5*01:01	98.33% (59/60)	Non-Toxin	0.7	Consensus (smm/nn/sturniolo)
32	c protein	MMASILLTLFRRTKK	HLA-DPA1*03:01/DPB1*04:02	100.00% (60/60)	Non-Toxin	0.35	Consensus (comb.lib./smm/nn)
33	c protein	MMASILLTLFRRTKK	HLA-DPA1*01:03/DPB1*02:01	100.00% (60/60)	Non-Toxin	0.73	Consensus (comb.lib./smm/nn)
34	c protein	MMEMLKEETWRIYPV	HLA-DRB3*01:01	21.67% (13/60)	Non-Toxin	0.18	Consensus (comb.lib./smm/nn)
35	c protein	MTYNWTQWLQTLYTM	HLA-DPA1*01:03/DPB1*02:01	95.00% (57/60)	Non-Toxin	0.23	Consensus (comb.lib./smm/nn)
36	c protein	PMLPKLQFLKKIGKL	HLA-DRB1*11:01	98.33% (59/60)	Non-Toxin	0.19	Consensus (smm/nn/sturniolo)
37	c protein	PMLPKLQFLKKIGKL	HLA-DRB5*01:01	98.33% (59/60)	Non-Toxin	0.7	Consensus (smm/nn/sturniolo)
38	c protein	QKVQMTYNWTQWLQT	HLA-DPA1*01:03/DPB1*02:01	95.00% (57/60)	Non-Toxin	0.14	Consensus (comb.lib./smm/nn)
39	c protein	QMTYNWTQWLQTLYT	HLA-DPA1*01:03/DPB1*02:01	95.00% (57/60)	Non-Toxin	0.1	Consensus (comb.lib./smm/nn)
40	c protein	QMTYNWTQWLQTLYT	HLA-DPA1*01/DPB1*04:01	95.00% (57/60)	Non-Toxin	0.77	Consensus (comb.lib./smm/nn)
41	c protein	QMTYNWTQWLQTLYT	HLA-DPA1*02:01/DPB1*01:01	95.00% (57/60)	Non-Toxin	0.79	Consensus (comb.lib./smm/nn)
42	c protein	QMTYNWTQWLQTLYT	HLA-DPA1*03:01/DPB1*04:02	95.00% (57/60)	Non-Toxin	0.86	Consensus (comb.lib./smm/nn)

#	Protein	Peptide	Allele	Conservancy	Toxicity	Percentile rank	Method used
43	c protein	QTLYTMIMEENVPDM	HLA-DQA1*04:01/DQB1*04:02	100.00% (60/60)	Non-Toxin	0.43	Consensus (comb.lib./smm/nn)
44	c protein	QWLQTLYTMIMEENV	HLA-DQA1*04:01/DQB1*04:02	100.00% (60/60)	Non-Toxin	0.09	Consensus (comb.lib./smm/nn)
45	c protein	RHTDDQVFNNPASKI	HLA-DRB1*13:02	21.67% (13/60)	Non-Toxin	0.61	Consensus (smm/nn/sturniolo)
46	c protein	RMMEMLKEETWRIYP	HLA-DRB3*01:01	21.67% (13/60)	Non-Toxin	0.19	Consensus (comb.lib./smm/nn)
47	c protein	SILLTLFRRTKKKYR	HLA-DRB5*01:01	100.00% (60/60)	Non-Toxin	0.17	Consensus (smm/nn/sturniolo)
48	c protein	SILLTLFRRTKKKYR	HLA-DRB1*11:01	100.00% (60/60)	Non-Toxin	0.4	Consensus (smm/nn/sturniolo)
49	c protein	TDDQVFNNPASKIKQ	HLA-DRB1*13:02	21.67% (13/60)	Non-Toxin	0.3	Consensus (smm/nn/sturniolo)
50	c protein	TLFRRTKKKYRRHTD	HLA-DRB5*01:01	95.00% (57/60)	Non-Toxin	0.17	Consensus (smm/nn/sturniolo)
51	c protein	TLFRRTKKKYRRHTD	HLA-DRB1*11:01	95.00% (57/60)	Non-Toxin	0.95	Consensus (smm/nn/sturniolo)
52	c protein	TLYTMIMEENVPDMD	HLA-DQA1*04:01/DQB1*04:02	100.00% (60/60)	Non-Toxin	0.72	Consensus (comb.lib./smm/nn)
53	c protein	TQWLQTLYTMIMEEN	HLA-DQA1*04:01/DQB1*04:02	100.00% (60/60)	Non-Toxin	0.08	Consensus (comb.lib./smm/nn)
54	c protein	TWRIYPVLLPQMELL	HLA-DRB1*08:02	21.67% (13/60)	Non-Toxin	0.36	Consensus (smm/nn/sturniolo)
55	c protein	TYNWTQWLQTLYTMI	HLA-DPA1*01:03/DPB1*02:01	95.00% (57/60)	Non-Toxin	0.52	Consensus (comb.lib./smm/nn)
56	c protein	TYNWTQWLQTLYTMI	HLA-DPA1*02:01/DPB1*01:01	95.00% (57/60)	Non-Toxin	0.96	Consensus (comb.lib./smm/nn)
57	c protein	VQMTYNWTQWLQTLY	HLA-DPA1*01:03/DPB1*02:01	95.00% (57/60)	Non-Toxin	0.08	Consensus (comb.lib./smm/nn)
58	c protein	VQMTYNWTQWLQTLY	HLA-DPA1*01/DPB1*04:01	95.00% (57/60)	Non-Toxin	0.7	Consensus (comb.lib./smm/nn)
59	c protein	VQMTYNWTQWLQTLY	HLA-DPA1*02:01/DPB1*01:01	95.00% (57/60)	Non-Toxin	0.8	Consensus (comb.lib./smm/nn)
60	c protein	WLQTLYTMIMEENVP	HLA-DQA1*04:01/DQB1*04:02	100.00% (60/60)	Non-Toxin	0.1	Consensus (comb.lib./smm/nn)
61	c protein	WRIYPVLLPQMELLE	HLA-DRB1*08:02	100.00% (60/60)	Non-Toxin	0.66	Consensus (smm/nn/sturniolo)
62	c protein	WTQWLQTLYTMIMEE	HLA-DQA1*04:01/DQB1*04:02	100.00% (60/60)	Non-Toxin	0.1	Consensus (comb.lib./smm/nn)
63	fusion protein	AEKTVYVLTALQDYI	HLA-DQA1*05:01/DQB1*02:01	100.00% (37/37)	Non-Toxin	0.46	Consensus (comb.lib./smm/nn)
64	fusion protein	AFGGNYETLLRTLGY	HLA-DRB1*11:01	97.30% (36/37)	Non-Toxin	0.44	Consensus (smm/nn/sturniolo)
65	fusion protein	AGVAIGIATAAQITA	HLA-DRB1*08:02	97.30% (36/37)	Non-Toxin	0.31	Consensus (smm/nn/sturniolo)
66	fusion protein	AGVIMAGVAIGIATA	HLA-DQA1*05:01/DQB1*03:01	97.30% (36/37)	Non-Toxin	0.27	Consensus (comb.lib./smm/nn)
67	fusion protein	ALSKYLSDLLFVFGP	HLA-DRB3*01:01	97.30% (36/37)	Non-Toxin	0.01	Consensus (comb.lib./smm/nn)
68	fusion protein	ASLCIGLITFISFII	HLA-DRB1*15:01	100.00% (37/37)	Non-Toxin	0.11	Consensus (smm/nn/sturniolo)
69	fusion protein	ASLCIGLITFISFII	HLA-DPA1*01:03/DPB1*02:01	100.00% (37/37)	Non-Toxin	0.37	Consensus (comb.lib./smm/nn)
70	fusion protein	CIGLITFISFIIVEK	HLA-DPA1*01:03/DPB1*02:01	100.00% (37/37)	Non-Toxin	0.05	Consensus (comb.lib./smm/nn)
71	fusion protein	CIGLITFISFIIVEK	HLA-DRB1*15:01	100.00% (37/37)	Non-Toxin	0.1	Consensus (smm/nn/sturniolo)
72	fusion protein	CNLLILILMISECSV	HLA-DRB1*11:01	40.54% (15/37)	Non-Toxin	0.12	Consensus (smm/nn/sturniolo)
73	fusion protein	CNLLILILMISECSV	HLA-DRB1*04:05	40.54% (15/37)	Non-Toxin	0.47	Consensus (smm/nn/sturniolo)
74	fusion protein	CPRELVVSSHVPRFA	HLA-DRB1*03:01	100.00% (37/37)	Non-Toxin	0.06	Consensus (smm/nn/sturniolo)
75	fusion protein	CYCNLLILILMISEC	HLA-DPA1*03:01/DPB1*04:02	40.54% (15/37)	Non-Toxin	0.12	Consensus (comb.lib./smm/nn)
76	fusion protein	CYCNLLILILMISEC	HLA-DRB1*11:01	40.54% (15/37)	Non-Toxin	0.28	Consensus (smm/nn/sturniolo)
77	fusion protein	DIVIKMIPNVSNMSQ	HLA-DRB1*08:02	97.30% (36/37)	Non-Toxin	0.1	Consensus (smm/nn/sturniolo)
78	fusion protein	DKRCYCNLLILILMI	HLA-DPA1*03:01/DPB1*04:02	40.54% (15/37)	Non-Toxin	0.05	Consensus (comb.lib./smm/nn)
79	fusion protein	DLALSKYLSDLLFVF	HLA-DRB3*01:01	97.30% (36/37)	Non-Toxin	0.01	Consensus (comb.lib./smm/nn)
80	fusion protein	DLSSYYIIVRVYFPI	HLA-DRB3*01:01	45.95% (17/37)	Non-Toxin	0.05	Consensus (comb.lib./smm/nn)
81	fusion protein	DLSSYYIIVRVYFPI	HLA-DPA1*02:01/DPB1*01:01	45.95% (17/37)	Non-Toxin	0.43	Consensus (comb.lib./smm/nn)
82	fusion protein	DLVGDVRLAGVIMAG	HLA-DRB1*03:01	100.00% (37/37)	Non-Toxin	0.43	Consensus (smm/nn/sturniolo)
83	fusion protein	DNSEWISIVPNFILV	HLA-DRB5*01:01	97.30% (36/37)	Non-Toxin	0.38	Consensus (smm/nn/sturniolo)
84	fusion protein	DPVSNSMTIQAISQA	HLA-DQA1*01:02/DQB1*06:02	97.30% (36/37)	Non-Toxin	0.06	Consensus (comb.lib./smm/nn)
85	fusion protein	DTVNPSLISMLSMII	HLA-DRB1*15:01	100.00% (37/37)	Non-Toxin	0.3	Consensus (smm/nn/sturniolo)
86	fusion protein	EIGFCLITKRSVICN	HLA-DRB1*11:01	100.00% (37/37)	Non-Toxin	0.42	Consensus (smm/nn/sturniolo)
87	fusion protein	EKKRNTYSRLEDRRV	HLA-DRB1*09:01	100.00% (37/37)	Non-Toxin	0.25	Consensus (comb.lib./smm/nn)
88	fusion protein	EKTVYVLTALQDYIN	HLA-DQA1*05:01/DQB1*02:01	100.00% (37/37)	Non-Toxin	0.49	Consensus (comb.lib./smm/nn)
89	fusion protein	ELVVSSHVPRFALSN	HLA-DRB1*03:01	100.00% (37/37)	Non-Toxin	0.06	Consensus (smm/nn/sturniolo)

#	Protein	Peptide	Allele	Conservancy	Toxicity	Percentile rank	Method used
90	fusion protein	FGGNYETLLRTLGYA	HLA-DRB1*11:01	97.30% (36/37)	Non-Toxin	0.18	Consensus (smm/nn/sturniolo)
91	fusion protein	FISFIIVEKKRNTYS	HLA-DRB1*11:01	100.00% (37/37)	Non-Toxin	0.34	Consensus (smm/nn/sturniolo)
92	fusion protein	GGNYETLLRTLGYAT	HLA-DRB1*11:01	94.59% (35/37)	Non-Toxin	0.09	Consensus (smm/nn/sturniolo)
93	fusion protein	GLITFISFIIVEKKR	HLA-DPA1*01:03/DPB1*02:01	100.00% (37/37)	Non-Toxin	0.05	Consensus (comb.lib./smm/nn)
94	fusion protein	GNYETLLRTLGYATE	HLA-DRB1*11:01	94.59% (35/37)	Non-Toxin	0.22	Consensus (smm/nn/sturniolo)
95	fusion protein	GQIIYVDLSSYYIIV	HLA-DRB1*15:01	45.95% (17/37)	Non-Toxin	0.28	Consensus (smm/nn/sturniolo)
96	fusion protein	GQIIYVDLSSYYIIV	HLA-DRB3*01:01	45.95% (17/37)	Non-Toxin	0.4	Consensus (comb.lib./smm/nn)
97	fusion protein	GVAIGIATAAQITAG	HLA-DRB1*08:02	97.30% (36/37)	Non-Toxin	0.32	Consensus (smm/nn/sturniolo)
98	fusion protein	GVIMAGVAIGIATAA	HLA-DQA1*05:01/DQB1*03:01	97.30% (36/37)	Non-Toxin	0.37	Consensus (comb.lib./smm/nn)
99	fusion protein	HDLVGDVRLAGVIMA	HLA-DRB1*03:01	100.00% (37/37)	Non-Toxin	0.05	Consensus (smm/nn/sturniolo)
100	fusion protein	IGLITFISFIIVEKK	HLA-DPA1*01:03/DPB1*02:01	100.00% (37/37)	Non-Toxin	0.05	Consensus (comb.lib./smm/nn)
101	fusion protein	IGLITFISFIIVEKK	HLA-DRB1*15:01	100.00% (37/37)	Non-Toxin	0.1	Consensus (smm/nn/sturniolo)
102	fusion protein	IIVRVYFPILTEIQQ	HLA-DPA1*02:01/DPB1*01:01	100.00% (37/37)	Non-Toxin	0.27	Consensus (comb.lib./smm/nn)
103	fusion protein	IIVRVYFPILTEIQQ	HLA-DPA1*01:03/DPB1*02:01	100.00% (37/37)	Non-Toxin	0.28	Consensus (comb.lib./smm/nn)
104	fusion protein	IIVRVYFPILTEIQQ	HLA-DQA1*01:01/DQB1*05:01	100.00% (37/37)	Non-Toxin	0.38	Consensus (comb.lib./smm/nn)
105	fusion protein	IKMIPNVSNMSQCTG	HLA-DRB1*08:02	97.30% (36/37)	Non-Toxin	0.3	Consensus (smm/nn/sturniolo)
106	fusion protein	IKSNPLTKDIVIKMI	HLA-DRB1*03:01	100.00% (37/37)	Non-Toxin	0.25	Consensus (smm/nn/sturniolo)
107	fusion protein	ILDKRCYCNLLILIL	HLA-DPA1*03:01/DPB1*04:02	40.54% (15/37)	Non-Toxin	0.06	Consensus (comb.lib./smm/nn)
108	fusion protein	ISFIIVEKKRNTYSR	HLA-DRB1*11:01	100.00% (37/37)	Non-Toxin	0.44	Consensus (smm/nn/sturniolo)
109	fusion protein	ISIVPNFILVRNTLI	HLA-DRB1*07:01	100.00% (37/37)	Non-Toxin	0.07	Consensus (comb.lib./smm/nn)
110	fusion protein	ISIVPNFILVRNTLI	HLA-DRB1*01:01	100.00% (37/37)	Non-Toxin	0.28	Consensus (comb.lib./smm/nn)
111	fusion protein	ISIVPNFILVRNTLI	HLA-DRB1*04:05	100.00% (37/37)	Non-Toxin	0.34	Consensus (smm/nn/sturniolo)
112	fusion protein	ISMLSMIILYVLSIA	HLA-DRB4*01:01	100.00% (37/37)	Non-Toxin	0.16	Consensus (comb.lib./smm/nn)
113	fusion protein	ISMLSMIILYVLSIA	HLA-DRB1*11:01	100.00% (37/37)	Non-Toxin	0.39	Consensus (smm/nn/sturniolo)
114	fusion protein	ITGQIIYVDLSSYYI	HLA-DRB3*01:01	45.95% (17/37)	Non-Toxin	0.42	Consensus (comb.lib./smm/nn)
115	fusion protein	IVIKMIPNVSNMSQC	HLA-DRB1*08:02	97.30% (36/37)	Non-Toxin	0.11	Consensus (smm/nn/sturniolo)
116	fusion protein	IVPNFILVRNTLISN	HLA-DRB1*01:01	100.00% (37/37)	Non-Toxin	0.28	Consensus (comb.lib./smm/nn)
117	fusion protein	IVPNFILVRNTLISN	HLA-DRB1*07:01	100.00% (37/37)	Non-Toxin	0.29	Consensus (comb.lib./smm/nn)
118	fusion protein	IVPNFILVRNTLISN	HLA-DRB1*04:05	100.00% (37/37)	Non-Toxin	0.34	Consensus (smm/nn/sturniolo)
119	fusion protein	IVPNFILVRNTLISN	HLA-DRB1*08:02	100.00% (37/37)	Non-Toxin	0.42	Consensus (smm/nn/sturniolo)
120	fusion protein	IVRVYFPILTEIQQA	HLA-DPA1*02:01/DPB1*01:01	100.00% (37/37)	Non-Toxin	0.27	Consensus (comb.lib./smm/nn)
121	fusion protein	IVRVYFPILTEIQQA	HLA-DQA1*01:01/DQB1*05:01	100.00% (37/37)	Non-Toxin	0.43	Consensus (comb.lib./smm/nn)
122	fusion protein	IVRVYFPILTEIQQA	HLA-DPA1*01:03/DPB1*02:01	100.00% (37/37)	Non-Toxin	0.47	Consensus (comb.lib./smm/nn)
123	fusion protein	KCPRELVVSSHVPRF	HLA-DRB1*03:01	100.00% (37/37)	Non-Toxin	0.06	Consensus (smm/nn/sturniolo)
124	fusion protein	KDIVIKMIPNVSNMS	HLA-DRB1*08:02	97.30% (36/37)	Non-Toxin	0.16	Consensus (smm/nn/sturniolo)
125	fusion protein	KIKSNPLTKDIVIKM	HLA-DRB1*03:01	100.00% (37/37)	Non-Toxin	0.25	Consensus (smm/nn/sturniolo)
126	fusion protein	KNNTHDLVGDVRLAG	HLA-DRB1*03:01	100.00% (37/37)	Non-Toxin	0.08	Consensus (smm/nn/sturniolo)
127	fusion protein	KQTELSLDLALSKYL	HLA-DRB1*03:01	97.30% (36/37)	Non-Toxin	0.37	Consensus (smm/nn/sturniolo)
128	fusion protein	KRCYCNLLILILMIS	HLA-DPA1*03:01/DPB1*04:02	40.54% (15/37)	Non-Toxin	0.05	Consensus (comb.lib./smm/nn)
129	fusion protein	KRCYCNLLILILMIS	HLA-DRB1*11:01	40.54% (15/37)	Non-Toxin	0.19	Consensus (smm/nn/sturniolo)
130	fusion protein	KSNPLTKDIVIKMIP	HLA-DRB1*03:01	97.30% (36/37)	Non-Toxin	0.25	Consensus (smm/nn/sturniolo)
131	fusion protein	KTVYVLTALQDYINT	HLA-DQA1*05:01/DQB1*02:01	100.00% (37/37)	Non-Toxin	0.49	Consensus (comb.lib./smm/nn)
132	fusion protein	KYLSDLLFVFGPNLQ	HLA-DRB3*01:01	97.30% (36/37)	Non-Toxin	0.01	Consensus (comb.lib./smm/nn)
133	fusion protein	LAGVIMAGVAIGIAT	HLA-DQA1*05:01/DQB1*03:01	97.30% (36/37)	Non-Toxin	0.32	Consensus (comb.lib./smm/nn)
134	fusion protein	LALSKYLSDLLFVFG	HLA-DRB3*01:01	97.30% (36/37)	Non-Toxin	0.01	Consensus (comb.lib./smm/nn)
135	fusion protein	LCIGLITFISFIIVE	HLA-DPA1*01:03/DPB1*02:01	100.00% (37/37)	Non-Toxin	0.04	Consensus (comb.lib./smm/nn)
136	fusion protein	LCIGLITFISFIIVE	HLA-DRB1*15:01	100.00% (37/37)	Non-Toxin	0.11	Consensus (smm/nn/sturniolo)

S.No.	Protein	Peptide	Allele	Conservancy	Toxicity	Percentile rank	Method used
137	fusion protein	LDKRCYCNLLILILM	HLA-DPA1*03:01/DPB1*04:02	40.54% (15/37)	Non-Toxin	0.06	Consensus (comb.lib./smm/nn)
138	fusion protein	LISMLSMIILYVLSI	HLA-DRB4*01:01	100.00% (37/37)	Non-Toxin	0.16	Consensus (comb.lib./smm/nn)
139	fusion protein	LISMLSMIILYVLSI	HLA-DRB1*11:01	100.00% (37/37)	Non-Toxin	0.3	Consensus (smm/nn/sturniolo)
140	fusion protein	LITFISFIIVEKKRN	HLA-DPA1*01:03/DPB1*02:01	100.00% (37/37)	Non-Toxin	0.37	Consensus (comb.lib./smm/nn)
141	fusion protein	LLILILMISECSVGI	HLA-DRB1*11:01	72.97% (27/37)	Non-Toxin	0.3	Consensus (smm/nn/sturniolo)
142	fusion protein	LLPVSFNNDNSEWIS	HLA-DRB3*01:01	97.30% (36/37)	Non-Toxin	0.5	Consensus (comb.lib./smm/nn)
143	fusion protein	LPVSFNNDNSEWISI	HLA-DRB3*01:01	97.30% (36/37)	Non-Toxin	0.46	Consensus (comb.lib./smm/nn)
144	fusion protein	LQDPVSNSMTIQAIS	HLA-DQA1*01:02/DQB1*06:02	97.30% (36/37)	Non-Toxin	0.06	Consensus (comb.lib./smm/nn)
145	fusion protein	LRTLGYATEDFDDLL	HLA-DQA1*05:01/DQB1*02:01	97.30% (36/37)	Non-Toxin	0.46	Consensus (comb.lib./smm/nn)
146	fusion protein	LSKYLSDLLFVFGPN	HLA-DRB3*01:01	97.30% (36/37)	Non-Toxin	0.01	Consensus (comb.lib./smm/nn)
147	fusion protein	LSMIILYVLSIASLC	HLA-DRB1*11:01	100.00% (37/37)	Non-Toxin	0.05	Consensus (smm/nn/sturniolo)
148	fusion protein	LSMIILYVLSIASLC	HLA-DRB1*15:01	100.00% (37/37)	Non-Toxin	0.23	Consensus (smm/nn/sturniolo)
149	fusion protein	LSMIILYVLSIASLC	HLA-DRB1*04:01	100.00% (37/37)	Non-Toxin	0.42	Consensus (smm/nn/sturniolo)
150	fusion protein	LSSYYIIVRVYFPIL	HLA-DPA1*01:03/DPB1*02:01	45.95% (17/37)	Non-Toxin	0.04	Consensus (comb.lib./smm/nn)
151	fusion protein	LSSYYIIVRVYFPIL	HLA-DRB3*01:01	45.95% (17/37)	Non-Toxin	0.05	Consensus (comb.lib./smm/nn)
152	fusion protein	LSSYYIIVRVYFPIL	HLA-DPA1*01/DPB1*04:01	45.95% (17/37)	Non-Toxin	0.15	Consensus (comb.lib./smm/nn)
153	fusion protein	LSSYYIIVRVYFPIL	HLA-DPA1*02:01/DPB1*01:01	45.95% (17/37)	Non-Toxin	0.27	Consensus (comb.lib./smm/nn)
154	fusion protein	LTEIQQAYIQELLPV	HLA-DQA1*05:01/DQB1*02:01	100.00% (37/37)	Non-Toxin	0.3	Consensus (comb.lib./smm/nn)
155	fusion protein	LTKDIVIKMIPNVSN	HLA-DRB1*08:02	97.30% (36/37)	Non-Toxin	0.22	Consensus (smm/nn/sturniolo)
156	fusion protein	LTKDIVIKMIPNVSN	HLA-DRB1*03:01	97.30% (36/37)	Non-Toxin	0.25	Consensus (smm/nn/sturniolo)
157	fusion protein	LVGDVRLAGVIMAGV	HLA-DRB1*03:01	100.00% (37/37)	Non-Toxin	0.41	Consensus (smm/nn/sturniolo)
158	fusion protein	LVVSSHVPRFALSNG	HLA-DRB1*03:01	100.00% (37/37)	Non-Toxin	0.4	Consensus (smm/nn/sturniolo)
159	fusion protein	MIILYVLSIASLCIG	HLA-DRB1*11:01	100.00% (37/37)	Non-Toxin	0.05	Consensus (smm/nn/sturniolo)
160	fusion protein	MIILYVLSIASLCIG	HLA-DRB1*15:01	100.00% (37/37)	Non-Toxin	0.28	Consensus (smm/nn/sturniolo)
161	fusion protein	MIILYVLSIASLCIG	HLA-DRB1*04:01	100.00% (37/37)	Non-Toxin	0.4	Consensus (smm/nn/sturniolo)
162	fusion protein	MLSMIILYVLSIASL	HLA-DRB1*11:01	100.00% (37/37)	Non-Toxin	0.05	Consensus (smm/nn/sturniolo)
163	fusion protein	MLSMIILYVLSIASL	HLA-DRB1*15:01	100.00% (37/37)	Non-Toxin	0.23	Consensus (smm/nn/sturniolo)
164	fusion protein	MLSMIILYVLSIASL	HLA-DRB1*04:01	100.00% (37/37)	Non-Toxin	0.46	Consensus (smm/nn/sturniolo)
165	fusion protein	MLSMIILYVLSIASL	HLA-DPA1*02:01/DPB1*01:01	100.00% (37/37)	Non-Toxin	0.51	Consensus (comb.lib./smm/nn)
166	fusion protein	MVVILDKRCYCNLLI	HLA-DRB1*03:01	40.54% (15/37)	Non-Toxin	0.15	Consensus (smm/nn/sturniolo)
167	fusion protein	MVVILDKRCYCNLLI	HLA-DRB1*11:01	40.54% (15/37)	Non-Toxin	0.48	Consensus (smm/nn/sturniolo)
168	fusion protein	NDNSEWISIVPNFIL	HLA-DRB5*01:01	97.30% (36/37)	Non-Toxin	0.38	Consensus (smm/nn/sturniolo)
169	fusion protein	NFILVRNTLISNIEI	HLA-DRB1*08:02	100.00% (37/37)	Non-Toxin	0.4	Consensus (smm/nn/sturniolo)
170	fusion protein	NLLILILMISECSVG	HLA-DRB1*11:01	72.97% (27/37)	Non-Toxin	0.26	Consensus (smm/nn/sturniolo)
171	fusion protein	NLLILILMISECSVG	HLA-DRB1*04:05	72.97% (27/37)	Non-Toxin	0.47	Consensus (smm/nn/sturniolo)
172	fusion protein	NLQDPVSNSMTIQAI	HLA-DQA1*01:02/DQB1*06:02	97.30% (36/37)	Non-Toxin	0.18	Consensus (comb.lib./smm/nn)
173	fusion protein	NNDNSEWISIVPNFI	HLA-DRB5*01:01	97.30% (36/37)	Non-Toxin	0.38	Consensus (smm/nn/sturniolo)
174	fusion protein	NNTHDLVGDVRLAGV	HLA-DRB1*03:01	100.00% (37/37)	Non-Toxin	0.06	Consensus (smm/nn/sturniolo)
175	fusion protein	NPLTKDIVIKMIPNV	HLA-DRB1*03:01	97.30% (36/37)	Non-Toxin	0.25	Consensus (smm/nn/sturniolo)
176	fusion protein	NPSLISMLSMIILYV	HLA-DRB4*01:01	100.00% (37/37)	Non-Toxin	0.17	Consensus (comb.lib./smm/nn)
177	fusion protein	NPSLISMLSMIILYV	HLA-DRB1*15:01	100.00% (37/37)	Non-Toxin	0.27	Consensus (smm/nn/sturniolo)
178	fusion protein	NSEWISIVPNFILVR	HLA-DRB5*01:01	97.30% (36/37)	Non-Toxin	0.38	Consensus (smm/nn/sturniolo)
179	fusion protein	NSMTIQAISQAFGGN	HLA-DRB1*08:02	97.30% (36/37)	Non-Toxin	0.35	Consensus (smm/nn/sturniolo)
180	fusion protein	NTHDLVGDVRLAGVI	HLA-DRB1*03:01	100.00% (37/37)	Non-Toxin	0.05	Consensus (smm/nn/sturniolo)
181	fusion protein	PLTKDIVIKMIPNVS	HLA-DRB1*03:01	97.30% (36/37)	Non-Toxin	0.25	Consensus (smm/nn/sturniolo)
182	fusion protein	PNFILVRNTLISNIE	HLA-DRB1*01:01	100.00% (37/37)	Non-Toxin	0.28	Consensus (comb.lib./smm/nn)
183	fusion protein	PNFILVRNTLISNIE	HLA-DRB1*08:02	100.00% (37/37)	Non-Toxin	0.34	Consensus (smm/nn/sturniolo)

#	Protein	Peptide	Allele	Conservancy	Toxicity	Percentile rank	Method used
184	fusion protein	PNFILVRNTLISNIE	HLA-DRB1*04:05	100.00% (37/37)	Non-Toxin	0.34	Consensus (smm/nn/sturniolo)
185	fusion protein	PRELVVSSHVPRFAL	HLA-DRB1*03:01	100.00% (37/37)	Non-Toxin	0.06	Consensus (smm/nn/sturniolo)
186	fusion protein	PSLISMLSMIILYVL	HLA-DRB4*01:01	100.00% (37/37)	Non-Toxin	0.15	Consensus (comb.lib./smm/nn)
187	fusion protein	PSLISMLSMIILYVL	HLA-DRB1*15:01	100.00% (37/37)	Non-Toxin	0.27	Consensus (smm/nn/sturniolo)
188	fusion protein	PVSFNNDNSEWISIV	HLA-DRB3*01:01	97.30% (36/37)	Non-Toxin	0.48	Consensus (comb.lib./smm/nn)
189	fusion protein	PVSNSMTIQAISQAF	HLA-DQA1*01:02/DQB1*06:02	97.30% (36/37)	Non-Toxin	0.07	Consensus (comb.lib./smm/nn)
190	fusion protein	QDPVSNSMTIQAISQ	HLA-DQA1*01:02/DQB1*06:02	97.30% (36/37)	Non-Toxin	0.06	Consensus (comb.lib./smm/nn)
191	fusion protein	QIIYVDLSSYYIIVR	HLA-DRB1*15:01	45.95% (17/37)	Non-Toxin	0.33	Consensus (smm/nn/sturniolo)
192	fusion protein	QTELSLDLALSKYLS	HLA-DRB1*03:01	97.30% (36/37)	Non-Toxin	0.28	Consensus (smm/nn/sturniolo)
193	fusion protein	RCYCNLLILILMISE	HLA-DPA1*03:01/DPB1*04:02	40.54% (15/37)	Non-Toxin	0.05	Consensus (comb.lib./smm/nn)
194	fusion protein	RCYCNLLILILMISE	HLA-DRB1*11:01	40.54% (15/37)	Non-Toxin	0.17	Consensus (smm/nn/sturniolo)
195	fusion protein	RELVVSSHVPRFALS	HLA-DRB1*03:01	100.00% (37/37)	Non-Toxin	0.06	Consensus (smm/nn/sturniolo)
196	fusion protein	RLAGVIMAGVAIGIA	HLA-DQA1*05:01/DQB1*03:01	100.00% (37/37)	Non-Toxin	0.46	Consensus (comb.lib./smm/nn)
197	fusion protein	RTLGYATEDFDDLLE	HLA-DQA1*05:01/DQB1*02:01	97.30% (36/37)	Non-Toxin	0.5	Consensus (comb.lib./smm/nn)
198	fusion protein	SEWISIVPNFILVRN	HLA-DRB5*01:01	97.30% (36/37)	Non-Toxin	0.38	Consensus (smm/nn/sturniolo)
199	fusion protein	SITGQIIYVDLSSYY	HLA-DRB3*01:01	45.95% (17/37)	Non-Toxin	0.49	Consensus (comb.lib./smm/nn)
200	fusion protein	SIVPNFILVRNTLIS	HLA-DRB1*07:01	100.00% (37/37)	Non-Toxin	0.13	Consensus (comb.lib./smm/nn)
201	fusion protein	SIVPNFILVRNTLIS	HLA-DRB1*01:01	100.00% (37/37)	Non-Toxin	0.28	Consensus (comb.lib./smm/nn)
202	fusion protein	SIVPNFILVRNTLIS	HLA-DRB1*04:05	100.00% (37/37)	Non-Toxin	0.34	Consensus (smm/nn/sturniolo)
203	fusion protein	SKYLSDLLFVFGPNL	HLA-DRB3*01:01	97.30% (36/37)	Non-Toxin	0.01	Consensus (comb.lib./smm/nn)
204	fusion protein	SLCIGLITFISFIIV	HLA-DPA1*01:03/DPB1*02:01	100.00% (37/37)	Non-Toxin	0.04	Consensus (comb.lib./smm/nn)
205	fusion protein	SLCIGLITFISFIIV	HLA-DRB1*15:01	100.00% (37/37)	Non-Toxin	0.11	Consensus (smm/nn/sturniolo)
206	fusion protein	SLISMLSMIILYVLS	HLA-DRB4*01:01	100.00% (37/37)	Non-Toxin	0.15	Consensus (comb.lib./smm/nn)
207	fusion protein	SMIILYVLSIASLCI	HLA-DRB1*11:01	100.00% (37/37)	Non-Toxin	0.05	Consensus (smm/nn/sturniolo)
208	fusion protein	SMIILYVLSIASLCI	HLA-DRB1*15:01	100.00% (37/37)	Non-Toxin	0.23	Consensus (smm/nn/sturniolo)
209	fusion protein	SMIILYVLSIASLCI	HLA-DRB1*04:01	100.00% (37/37)	Non-Toxin	0.38	Consensus (smm/nn/sturniolo)
210	fusion protein	SMLSMIILYVLSIAS	HLA-DRB1*11:01	100.00% (37/37)	Non-Toxin	0.05	Consensus (smm/nn/sturniolo)
211	fusion protein	SMLSMIILYVLSIAS	HLA-DRB1*15:01	100.00% (37/37)	Non-Toxin	0.23	Consensus (smm/nn/sturniolo)
212	fusion protein	SMTIQAISQAFGGNY	HLA-DRB1*08:02	97.30% (36/37)	Non-Toxin	0.37	Consensus (smm/nn/sturniolo)
213	fusion protein	SNPLTKDIVIKMIPN	HLA-DRB1*03:01	97.30% (36/37)	Non-Toxin	0.25	Consensus (smm/nn/sturniolo)
214	fusion protein	SSYYIIVRVYFPILT	HLA-DPA1*01:03/DPB1*02:01	45.95% (17/37)	Non-Toxin	0.04	Consensus (comb.lib./smm/nn)
215	fusion protein	SSYYIIVRVYFPILT	HLA-DRB3*01:01	45.95% (17/37)	Non-Toxin	0.05	Consensus (comb.lib./smm/nn)
216	fusion protein	SSYYIIVRVYFPILT	HLA-DPA1*01/DPB1*04:01	45.95% (17/37)	Non-Toxin	0.15	Consensus (comb.lib./smm/nn)
217	fusion protein	SSYYIIVRVYFPILT	HLA-DPA1*02:01/DPB1*01:01	45.95% (17/37)	Non-Toxin	0.27	Consensus (comb.lib./smm/nn)
218	fusion protein	SYYIIVRVYFPILTE	HLA-DPA1*01:03/DPB1*02:01	45.95% (17/37)	Non-Toxin	0.05	Consensus (comb.lib./smm/nn)
219	fusion protein	SYYIIVRVYFPILTE	HLA-DPA1*01/DPB1*04:01	45.95% (17/37)	Non-Toxin	0.16	Consensus (comb.lib./smm/nn)
220	fusion protein	SYYIIVRVYFPILTE	HLA-DRB3*01:01	45.95% (17/37)	Non-Toxin	0.24	Consensus (comb.lib./smm/nn)
221	fusion protein	SYYIIVRVYFPILTE	HLA-DPA1*02:01/DPB1*01:01	45.95% (17/37)	Non-Toxin	0.27	Consensus (comb.lib./smm/nn)
222	fusion protein	SYYIIVRVYFPILTE	HLA-DQA1*01:01/DQB1*05:01	45.95% (17/37)	Non-Toxin	0.3	Consensus (comb.lib./smm/nn)
223	fusion protein	TGQIIYVDLSSYYII	HLA-DRB1*15:01	45.95% (17/37)	Non-Toxin	0.38	Consensus (smm/nn/sturniolo)
224	fusion protein	TGQIIYVDLSSYYII	HLA-DRB3*01:01	45.95% (17/37)	Non-Toxin	0.39	Consensus (comb.lib./smm/nn)
225	fusion protein	THDLVGDVRLAGVIM	HLA-DRB1*03:01	100.00% (37/37)	Non-Toxin	0.05	Consensus (smm/nn/sturniolo)
226	fusion protein	TKDIVIKMIPNVSNM	HLA-DRB1*08:02	97.30% (36/37)	Non-Toxin	0.22	Consensus (smm/nn/sturniolo)
227	fusion protein	TVNPSLISMLSMIIL	HLA-DRB4*01:01	100.00% (37/37)	Non-Toxin	0.22	Consensus (comb.lib./smm/nn)
228	fusion protein	TVNPSLISMLSMIIL	HLA-DRB1*15:01	100.00% (37/37)	Non-Toxin	0.27	Consensus (smm/nn/sturniolo)
229	fusion protein	VIKMIPNVSNMSQCT	HLA-DRB1*08:02	97.30% (36/37)	Non-Toxin	0.21	Consensus (smm/nn/sturniolo)
230	fusion protein	VNPSLISMLSMIILY	HLA-DRB4*01:01	100.00% (37/37)	Non-Toxin	0.22	Consensus (comb.lib./smm/nn)

#	Protein	Peptide	Allele	Conservancy	Toxicity	Percentile rank	Method used
231	fusion protein	VNPSLISMLSMIILY	HLA-DRB1*15:01	100.00% (37/37)	Non-Toxin	0.27	Consensus (smm/nn/sturniolo)
232	fusion protein	VPNFILVRNTLISNI	HLA-DRB1*01:01	100.00% (37/37)	Non-Toxin	0.28	Consensus (comb.lib./smm/nn)
233	fusion protein	VPNFILVRNTLISNI	HLA-DRB1*08:02	100.00% (37/37)	Non-Toxin	0.33	Consensus (smm/nn/sturniolo)
234	fusion protein	VPNFILVRNTLISNI	HLA-DRB1*04:05	100.00% (37/37)	Non-Toxin	0.34	Consensus (smm/nn/sturniolo)
235	fusion protein	VPNFILVRNTLISNI	HLA-DRB1*07:01	100.00% (37/37)	Non-Toxin	0.43	Consensus (comb.lib./smm/nn)
236	fusion protein	VRLAGVIMAGVAIGI	HLA-DQA1*05:01/DQB1*03:01	100.00% (37/37)	Non-Toxin	0.48	Consensus (comb.lib./smm/nn)
237	fusion protein	VSNSMTIQAISQAFG	HLA-DQA1*01:02/DQB1*06:02	97.30% (36/37)	Non-Toxin	0.46	Consensus (comb.lib./smm/nn)
238	fusion protein	YCNLLILILMISECS	HLA-DPA1*03:01/DPB1*04:02	40.54% (15/37)	Non-Toxin	0.12	Consensus (comb.lib./smm/nn)
239	fusion protein	YCNLLILILMISECS	HLA-DRB1*11:01	40.54% (15/37)	Non-Toxin	0.12	Consensus (smm/nn/sturniolo)
240	fusion protein	YCNLLILILMISECS	HLA-DRB1*04:05	40.54% (15/37)	Non-Toxin	0.47	Consensus (smm/nn/sturniolo)
241	fusion protein	YIIVRVYFPILTEIQ	HLA-DPA1*01:03/DPB1*02:01	100.00% (37/37)	Non-Toxin	0.08	Consensus (comb.lib./smm/nn)
242	fusion protein	YIIVRVYFPILTEIQ	HLA-DPA1*01/DPB1*04:01	100.00% (37/37)	Non-Toxin	0.15	Consensus (comb.lib./smm/nn)
243	fusion protein	YIIVRVYFPILTEIQ	HLA-DPA1*02:01/DPB1*01:01	100.00% (37/37)	Non-Toxin	0.27	Consensus (comb.lib./smm/nn)
244	fusion protein	YIIVRVYFPILTEIQ	HLA-DQA1*01:01/DQB1*05:01	100.00% (37/37)	Non-Toxin	0.28	Consensus (comb.lib./smm/nn)
245	fusion protein	YLSDLLFVFGPNLQD	HLA-DRB3*01:01	97.30% (36/37)	Non-Toxin	0.01	Consensus (comb.lib./smm/nn)
246	fusion protein	YYIIVRVYFPILTEI	HLA-DPA1*01:03/DPB1*02:01	100.00% (37/37)	Non-Toxin	0.04	Consensus (comb.lib./smm/nn)
247	fusion protein	YYIIVRVYFPILTEI	HLA-DPA1*01/DPB1*04:01	100.00% (37/37)	Non-Toxin	0.14	Consensus (comb.lib./smm/nn)
248	fusion protein	YYIIVRVYFPILTEI	HLA-DPA1*02:01/DPB1*01:01	100.00% (37/37)	Non-Toxin	0.27	Consensus (comb.lib./smm/nn)
249	fusion protein	YYIIVRVYFPILTEI	HLA-DQA1*01:01/DQB1*05:01	100.00% (37/37)	Non-Toxin	0.28	Consensus (comb.lib./smm/nn)
250	fusion protein	YYIIVRVYFPILTEI	HLA-DRB3*01:01	100.00% (37/37)	Non-Toxin	0.36	Consensus (comb.lib./smm/nn)
251	glycoprotein	AFLIDRINWISAGVF	HLA-DRB3*01:01	100.00% (66/66)	Non-Toxin	0.01	Consensus (comb.lib./smm/nn)
252	glycoprotein	ALLGSIVIIVMNIMI	HLA-DRB4*01:01	98.48% (65/66)	Non-Toxin	0.2	Consensus (comb.lib./smm/nn)
253	glycoprotein	ALLGSIVIIVMNIMI	HLA-DRB1*15:01	98.48% (65/66)	Non-Toxin	0.34	Consensus (smm/nn/sturniolo)
254	glycoprotein	AMDEGYFAYSHLERI	HLA-DPA1*03:01/DPB1*04:02	24.24% (16/66)	Non-Toxin	0.08	Consensus (comb.lib./smm/nn)
255	glycoprotein	AMDEGYFAYSHLERI	HLA-DPA1*02:01/DPB1*01:01	24.24% (16/66)	Non-Toxin	0.25	Consensus (comb.lib./smm/nn)
256	glycoprotein	AMDEGYFAYSHLERI	HLA-DPA1*01:03/DPB1*02:01	24.24% (16/66)	Non-Toxin	0.31	Consensus (comb.lib./smm/nn)
257	glycoprotein	ASFSWDTMIKFGDVL	HLA-DRB3*01:01	19.70% (13/66)	Non-Toxin	0.01	Consensus (comb.lib./smm/nn)
258	glycoprotein	AVIKDALQGIQQQIK	HLA-DRB4*01:01	24.24% (16/66)	Non-Toxin	0.35	Consensus (comb.lib./smm/nn)
259	glycoprotein	AYSHLERIGSCSRGV	HLA-DRB1*11:01	24.24% (16/66)	Non-Toxin	0.38	Consensus (smm/nn/sturniolo)
260	glycoprotein	CSAVYNNEFYYVLCA	HLA-DPA1*01/DPB1*04:01	90.91% (60/66)	Non-Toxin	0.37	Consensus (comb.lib./smm/nn)
261	glycoprotein	CWEGVYNDAFLIDRI	HLA-DRB3*01:01	100.00% (66/66)	Non-Toxin	0.18	Consensus (comb.lib./smm/nn)
262	glycoprotein	CWEGVYNDAFLIDRI	HLA-DPA1*01:03/DPB1*02:01	100.00% (66/66)	Non-Toxin	0.34	Consensus (comb.lib./smm/nn)
263	glycoprotein	DAFLIDRINWISAGV	HLA-DRB3*01:01	100.00% (66/66)	Non-Toxin	0.01	Consensus (comb.lib./smm/nn)
264	glycoprotein	DAFLIDRINWISAGV	HLA-DRB1*03:01	100.00% (66/66)	Non-Toxin	0.22	Consensus (smm/nn/sturniolo)
265	glycoprotein	DALQGIQQQIKGLAD	HLA-DRB4*01:01	24.24% (16/66)	Non-Toxin	0.35	Consensus (comb.lib./smm/nn)
266	glycoprotein	DEGYFAYSHLERIGS	HLA-DPA1*03:01/DPB1*04:02	24.24% (16/66)	Non-Toxin	0.03	Consensus (comb.lib./smm/nn)
267	glycoprotein	DEGYFAYSHLERIGS	HLA-DPA1*01:03/DPB1*02:01	24.24% (16/66)	Non-Toxin	0.22	Consensus (comb.lib./smm/nn)
268	glycoprotein	DEGYFAYSHLERIGS	HLA-DPA1*02:01/DPB1*01:01	24.24% (16/66)	Non-Toxin	0.29	Consensus (comb.lib./smm/nn)
269	glycoprotein	DEGYFAYSHLERIGS	HLA-DRB1*11:01	24.24% (16/66)	Non-Toxin	0.38	Consensus (smm/nn/sturniolo)
270	glycoprotein	DEGYFAYSHLERIGS	HLA-DPA1*02:01/DPB1*14:01	24.24% (16/66)	Non-Toxin	0.48	NetMHCIIpan
271	glycoprotein	DEGYFAYSHLERIGS	HLA-DPA1*01/DPB1*04:01	24.24% (16/66)	Non-Toxin	0.51	Consensus (comb.lib./smm/nn)
272	glycoprotein	DSKILSAFNTVIALL	HLA-DPA1*03:01/DPB1*04:02	98.48% (65/66)	Non-Toxin	0.44	Consensus (comb.lib./smm/nn)
273	glycoprotein	EGVYNDAFLIDRINW	HLA-DPA1*01:03/DPB1*02:01	100.00% (66/66)	Non-Toxin	0.35	Consensus (comb.lib./smm/nn)
274	glycoprotein	EGYFAYSHLERIGSC	HLA-DPA1*03:01/DPB1*04:02	24.24% (16/66)	Non-Toxin	0.02	Consensus (comb.lib./smm/nn)
275	glycoprotein	EGYFAYSHLERIGSC	HLA-DPA1*01:03/DPB1*02:01	24.24% (16/66)	Non-Toxin	0.21	Consensus (comb.lib./smm/nn)
276	glycoprotein	EGYFAYSHLERIGSC	HLA-DRB1*11:01	24.24% (16/66)	Non-Toxin	0.38	Consensus (smm/nn/sturniolo)
277	glycoprotein	EGYFAYSHLERIGSC	HLA-DPA1*02:01/DPB1*01:01	24.24% (16/66)	Non-Toxin	0.38	Consensus (comb.lib./smm/nn)

#	Protein	Peptide	Allele	Conservancy	Toxicity	Percentile rank	Method used
278	glycoprotein	EGYFAYSHLERIGSC	HLA-DPA1*01/DPB1*04:01	24.24% (16/66)	Non-Toxin	0.45	Consensus (comb.lib./smm/nn)
279	glycoprotein	EGYFAYSHLERIGSC	HLA-DPA1*02:01/DPB1*14:01	24.24% (16/66)	Non-Toxin	0.5	NetMHCIIpan
280	glycoprotein	EICWEGVYNDAFLID	HLA-DRB3*01:01	25.76% (17/66)	Non-Toxin	0.17	Consensus (comb.lib./smm/nn)
281	glycoprotein	EICWEGVYNDAFLID	HLA-DPA1*01:03/DPB1*02:01	25.76% (17/66)	Non-Toxin	0.37	Consensus (comb.lib./smm/nn)
282	glycoprotein	FAYSHLERIGSCSRG	HLA-DRB1*11:01	24.24% (16/66)	Non-Toxin	0.38	Consensus (smm/nn/sturniolo)
283	glycoprotein	FLIDRINWISAGVFL	HLA-DRB3*01:01	100.00% (66/66)	Non-Toxin	0.01	Consensus (comb.lib./smm/nn)
284	glycoprotein	FSWDTMIKFGDVLTV	HLA-DRB3*01:01	19.70% (13/66)	Non-Toxin	0.01	Consensus (comb.lib./smm/nn)
285	glycoprotein	FYQASFSWDTMIKFG	HLA-DRB3*01:01	100.00% (66/66)	Non-Toxin	0.26	Consensus (comb.lib./smm/nn)
286	glycoprotein	GSIVIIVMNIMIIQN	HLA-DRB4*01:01	98.48% (65/66)	Non-Toxin	0.15	Consensus (comb.lib./smm/nn)
287	glycoprotein	GSIVIIVMNIMIIQN	HLA-DRB1*15:01	98.48% (65/66)	Non-Toxin	0.31	Consensus (smm/nn/sturniolo)
288	glycoprotein	GVYNDAFLIDRINWI	HLA-DRB3*01:01	100.00% (66/66)	Non-Toxin	0.01	Consensus (comb.lib./smm/nn)
289	glycoprotein	GVYNDAFLIDRINWI	HLA-DRB1*03:01	100.00% (66/66)	Non-Toxin	0.22	Consensus (smm/nn/sturniolo)
290	glycoprotein	GYFAYSHLERIGSCS	HLA-DPA1*03:01/DPB1*04:02	24.24% (16/66)	Non-Toxin	0.03	Consensus (comb.lib./smm/nn)
291	glycoprotein	GYFAYSHLERIGSCS	HLA-DPA1*01:03/DPB1*02:01	24.24% (16/66)	Non-Toxin	0.26	Consensus (comb.lib./smm/nn)
292	glycoprotein	GYFAYSHLERIGSCS	HLA-DRB1*11:01	24.24% (16/66)	Non-Toxin	0.38	Consensus (smm/nn/sturniolo)
293	glycoprotein	HCSAVYNNEFYYVLC	HLA-DPA1*01/DPB1*04:01	90.91% (60/66)	Non-Toxin	0.38	Consensus (comb.lib./smm/nn)
294	glycoprotein	IALLGSIVIIVMNIM	HLA-DRB4*01:01	98.48% (65/66)	Non-Toxin	0.21	Consensus (comb.lib./smm/nn)
295	glycoprotein	IALLGSIVIIVMNIM	HLA-DRB1*15:01	98.48% (65/66)	Non-Toxin	0.46	Consensus (smm/nn/sturniolo)
296	glycoprotein	ICWEGVYNDAFLIDR	HLA-DRB3*01:01	25.76% (17/66)	Non-Toxin	0.17	Consensus (comb.lib./smm/nn)
297	glycoprotein	ICWEGVYNDAFLIDR	HLA-DPA1*01:03/DPB1*02:01	25.76% (17/66)	Non-Toxin	0.36	Consensus (comb.lib./smm/nn)
298	glycoprotein	IIVMNIMIIQNYTRS	HLA-DRB4*01:01	98.48% (65/66)	Non-Toxin	0.1	Consensus (comb.lib./smm/nn)
299	glycoprotein	IIVMNIMIIQNYTRS	HLA-DRB5*01:01	98.48% (65/66)	Non-Toxin	0.12	Consensus (smm/nn/sturniolo)
300	glycoprotein	IIVMNIMIIQNYTRS	HLA-DRB1*15:01	98.48% (65/66)	Non-Toxin	0.39	Consensus (smm/nn/sturniolo)
301	glycoprotein	IKDALQGIQQQIKGL	HLA-DRB4*01:01	24.24% (16/66)	Non-Toxin	0.3	Consensus (comb.lib./smm/nn)
302	glycoprotein	ILSAFNTVIALLGSI	HLA-DRB1*07:01	98.48% (65/66)	Non-Toxin	0.44	Consensus (comb.lib./smm/nn)
303	glycoprotein	ILSAFNTVIALLGSI	HLA-DPA1*03:01/DPB1*04:02	98.48% (65/66)	Non-Toxin	0.46	Consensus (comb.lib./smm/nn)
304	glycoprotein	ITNCFLLKNKIWCIS	HLA-DRB1*11:01	98.48% (65/66)	Non-Toxin	0.43	Consensus (smm/nn/sturniolo)
305	glycoprotein	IVIIVMNIMIIQNYT	HLA-DRB4*01:01	98.48% (65/66)	Non-Toxin	0.08	Consensus (comb.lib./smm/nn)
306	glycoprotein	IVMNIMIIQNYTRST	HLA-DRB4*01:01	98.48% (65/66)	Non-Toxin	0.1	Consensus (comb.lib./smm/nn)
307	glycoprotein	IVMNIMIIQNYTRST	HLA-DRB5*01:01	98.48% (65/66)	Non-Toxin	0.12	Consensus (smm/nn/sturniolo)
308	glycoprotein	IVMNIMIIQNYTRST	HLA-DRB1*15:01	98.48% (65/66)	Non-Toxin	0.44	Consensus (smm/nn/sturniolo)
309	glycoprotein	KDALQGIQQQIKGLA	HLA-DRB4*01:01	24.24% (16/66)	Non-Toxin	0.34	Consensus (comb.lib./smm/nn)
310	glycoprotein	KILSAFNTVIALLGS	HLA-DPA1*03:01/DPB1*04:02	98.48% (65/66)	Non-Toxin	0.46	Consensus (comb.lib./smm/nn)
311	glycoprotein	KILSAFNTVIALLGS	HLA-DRB1*07:01	98.48% (65/66)	Non-Toxin	0.47	Consensus (comb.lib./smm/nn)
312	glycoprotein	KTITNCFLLKNKIWC	HLA-DRB1*11:01	98.48% (65/66)	Non-Toxin	0.45	Consensus (smm/nn/sturniolo)
313	glycoprotein	LGSIVIIVMNIMIIQ	HLA-DRB4*01:01	98.48% (65/66)	Non-Toxin	0.14	Consensus (comb.lib./smm/nn)
314	glycoprotein	LGSIVIIVMNIMIIQ	HLA-DRB1*15:01	98.48% (65/66)	Non-Toxin	0.3	Consensus (smm/nn/sturniolo)
315	glycoprotein	LLGSIVIIVMNIMII	HLA-DRB4*01:01	98.48% (65/66)	Non-Toxin	0.14	Consensus (comb.lib./smm/nn)
316	glycoprotein	LLGSIVIIVMNIMII	HLA-DRB1*15:01	98.48% (65/66)	Non-Toxin	0.3	Consensus (smm/nn/sturniolo)
317	glycoprotein	LPNNICLQKTSNQIL	HLA-DRB1*07:01	96.97% (64/66)	Non-Toxin	0.34	Consensus (comb.lib./smm/nn)
318	glycoprotein	LSAFNTVIALLGSIV	HLA-DRB1*07:01	98.48% (65/66)	Non-Toxin	0.41	Consensus (comb.lib./smm/nn)
319	glycoprotein	LSAFNTVIALLGSIV	HLA-DPA1*03:01/DPB1*04:02	98.48% (65/66)	Non-Toxin	0.48	Consensus (comb.lib./smm/nn)
320	glycoprotein	MDEGYFAYSHLERIG	HLA-DPA1*03:01/DPB1*04:02	24.24% (16/66)	Non-Toxin	0.05	Consensus (comb.lib./smm/nn)
321	glycoprotein	MDEGYFAYSHLERIG	HLA-DPA1*01:03/DPB1*02:01	24.24% (16/66)	Non-Toxin	0.23	Consensus (comb.lib./smm/nn)
322	glycoprotein	MDEGYFAYSHLERIG	HLA-DPA1*02:01/DPB1*01:01	24.24% (16/66)	Non-Toxin	0.27	Consensus (comb.lib./smm/nn)
323	glycoprotein	MNIMIIQNYTRSTDN	HLA-DRB5*01:01	98.48% (65/66)	Non-Toxin	0.13	Consensus (smm/nn/sturniolo)
324	glycoprotein	NDAFLIDRINWISAG	HLA-DRB3*01:01	100.00% (66/66)	Non-Toxin	0.01	Consensus (comb.lib./smm/nn)

#	Protein	Peptide	Allele	Conservancy	Toxicity	Percentile rank	Method used
325	glycoprotein	NDAFLIDRINWISAG	HLA-DRB1*03:01	100.00% (66/66)	Non-Toxin	0.22	Consensus (smm/nn/sturniolo)
326	glycoprotein	PEICWEGVYNDAFLI	HLA-DRB3*01:01	25.76% (17/66)	Non-Toxin	0.19	Consensus (comb.lib./smm/nn)
327	glycoprotein	QASFSWDTMIKFGDV	HLA-DRB3*01:01	100.00% (66/66)	Non-Toxin	0.01	Consensus (comb.lib./smm/nn)
328	glycoprotein	SAFNTVIALLGSIVI	HLA-DRB1*07:01	98.48% (65/66)	Non-Toxin	0.38	Consensus (comb.lib./smm/nn)
329	glycoprotein	SAVYNNEFYYVLCAV	HLA-DPA1*01/DPB1*04:01	89.39% (59/66)	Non-Toxin	0.32	Consensus (comb.lib./smm/nn)
330	glycoprotein	SFSWDTMIKFGDVLT	HLA-DRB3*01:01	19.70% (13/66)	Non-Toxin	0.01	Consensus (comb.lib./smm/nn)
331	glycoprotein	SIVIIVMNIMIIQNY	HLA-DRB4*01:01	98.48% (65/66)	Non-Toxin	0.08	Consensus (comb.lib./smm/nn)
332	glycoprotein	SIVIIVMNIMIIQNY	HLA-DRB1*15:01	98.48% (65/66)	Non-Toxin	0.48	Consensus (smm/nn/sturniolo)
333	glycoprotein	SKILSAFNTVIALLG	HLA-DRB1*07:01	98.48% (65/66)	Non-Toxin	0.32	Consensus (comb.lib./smm/nn)
334	glycoprotein	SKILSAFNTVIALLG	HLA-DPA1*03:01/DPB1*04:02	98.48% (65/66)	Non-Toxin	0.48	Consensus (comb.lib./smm/nn)
335	glycoprotein	TITNCFLLKNKIWCI	HLA-DRB1*11:01	98.48% (65/66)	Non-Toxin	0.44	Consensus (smm/nn/sturniolo)
336	glycoprotein	VFYQASFSWDTMIKF	HLA-DRB3*01:01	100.00% (66/66)	Non-Toxin	0.23	Consensus (comb.lib./smm/nn)
337	glycoprotein	VIIVMNIMIIQNYTR	HLA-DRB4*01:01	98.48% (65/66)	Non-Toxin	0.1	Consensus (comb.lib./smm/nn)
338	glycoprotein	VIIVMNIMIIQNYTR	HLA-DRB5*01:01	98.48% (65/66)	Non-Toxin	0.12	Consensus (smm/nn/sturniolo)
339	glycoprotein	VIIVMNIMIIQNYTR	HLA-DRB1*15:01	98.48% (65/66)	Non-Toxin	0.31	Consensus (smm/nn/sturniolo)
340	glycoprotein	VIKDALQGIQQQIKG	HLA-DRB4*01:01	24.24% (16/66)	Non-Toxin	0.35	Consensus (comb.lib./smm/nn)
341	glycoprotein	VMNIMIIQNYTRSTD	HLA-DRB5*01:01	98.48% (65/66)	Non-Toxin	0.12	Consensus (smm/nn/sturniolo)
342	glycoprotein	VYNDAFLIDRINWIS	HLA-DRB3*01:01	100.00% (66/66)	Non-Toxin	0.01	Consensus (comb.lib./smm/nn)
343	glycoprotein	VYNDAFLIDRINWIS	HLA-DRB1*03:01	100.00% (66/66)	Non-Toxin	0.22	Consensus (smm/nn/sturniolo)
344	glycoprotein	WEGVYNDAFLIDRIN	HLA-DRB3*01:01	100.00% (66/66)	Non-Toxin	0.17	Consensus (comb.lib./smm/nn)
345	glycoprotein	WEGVYNDAFLIDRIN	HLA-DPA1*01:03/DPB1*02:01	100.00% (66/66)	Non-Toxin	0.34	Consensus (comb.lib./smm/nn)
346	glycoprotein	YFAYSHLERIGSCSR	HLA-DPA1*03:01/DPB1*04:02	24.24% (16/66)	Non-Toxin	0.1	Consensus (comb.lib./smm/nn)
347	glycoprotein	YFAYSHLERIGSCSR	HLA-DRB1*11:01	24.24% (16/66)	Non-Toxin	0.38	Consensus (smm/nn/sturniolo)
348	glycoprotein	YHCSAVYNNEFYYVL	HLA-DPA1*01/DPB1*04:01	90.91% (60/66)	Non-Toxin	0.36	Consensus (comb.lib./smm/nn)
349	glycoprotein	YNDAFLIDRINWISA	HLA-DRB3*01:01	100.00% (66/66)	Non-Toxin	0.01	Consensus (comb.lib./smm/nn)
350	glycoprotein	YNDAFLIDRINWISA	HLA-DRB1*03:01	100.00% (66/66)	Non-Toxin	0.22	Consensus (smm/nn/sturniolo)
351	glycoprotein	YQASFSWDTMIKFGD	HLA-DRB3*01:01	100.00% (66/66)	Non-Toxin	0.01	Consensus (comb.lib./smm/nn)
352	glycoprotein	YSHLERIGSCSRGVS	HLA-DRB1*11:01	24.24% (16/66)	Non-Toxin	0.38	Consensus (smm/nn/sturniolo)
353	Matrix Protein	ALRIFFLSITKLNDS	HLA-DPA1*01/DPB1*04:01	97.44% (38/39)	Non-Toxin	0.21	Consensus (comb.lib./smm/nn)
354	Matrix Protein	ALRIFFLSITKLNDS	HLA-DPA1*02:01/DPB1*14:01	97.44% (38/39)	Non-Toxin	0.3	NetMHCIIpan
355	Matrix Protein	CFSLMDINPWLNRLT	HLA-DRB3*01:01	94.87% (37/39)	Non-Toxin	0.07	Consensus (comb.lib./smm/nn)
356	Matrix Protein	DKHQALRIFFLSITK	HLA-DPA1*01/DPB1*04:01	97.44% (38/39)	Non-Toxin	0.05	Consensus (comb.lib./smm/nn)
357	Matrix Protein	DKHQALRIFFLSITK	HLA-DPA1*01:03/DPB1*02:01	97.44% (38/39)	Non-Toxin	0.08	Consensus (comb.lib./smm/nn)
358	Matrix Protein	DSGIYMIPRTMLEFR	HLA-DRB1*11:01	97.44% (38/39)	Non-Toxin	0.44	Consensus (smm/nn/sturniolo)
359	Matrix Protein	EFMIYDDVFIDNTGR	HLA-DRB3*01:01	94.87% (37/39)	Non-Toxin	0.02	Consensus (comb.lib./smm/nn)
360	Matrix Protein	EFRRNNAIAFNLLVY	HLA-DRB3*02:02	84.62% (33/39)	Non-Toxin	0.35	NetMHCIIpan
361	Matrix Protein	FMIYDDVFIDNTGRI	HLA-DRB3*01:01	94.87% (37/39)	Non-Toxin	0.01	Consensus (comb.lib./smm/nn)
362	Matrix Protein	FMIYDDVFIDNTGRI	HLA-DRB1*03:01	94.87% (37/39)	Non-Toxin	0.08	Consensus (smm/nn/sturniolo)
363	Matrix Protein	GKRKKIRTIAAYPLG	HLA-DRB1*08:02	97.44% (38/39)	Non-Toxin	0.32	Consensus (smm/nn/sturniolo)
364	Matrix Protein	GNFVRRAGKYYSVDY	HLA-DRB1*11:01	79.49% (31/39)	Non-Toxin	0.38	Consensus (smm/nn/sturniolo)
365	Matrix Protein	HIKINGVISKRLFAQ	HLA-DRB1*11:01	94.87% (37/39)	Non-Toxin	0.48	Consensus (smm/nn/sturniolo)
366	Matrix Protein	HLGNFVRRAGKYYSV	HLA-DRB1*11:01	79.49% (31/39)	Non-Toxin	0.38	Consensus (smm/nn/sturniolo)
367	Matrix Protein	HLVPWKKVLTSGSIF	HLA-DRB1*08:02	35.90% (14/39)	Non-Toxin	0.36	Consensus (smm/nn/sturniolo)
368	Matrix Protein	HQALRIFFLSITKLN	HLA-DPA1*01/DPB1*04:01	97.44% (38/39)	Non-Toxin	0.05	Consensus (comb.lib./smm/nn)
369	Matrix Protein	HQALRIFFLSITKLN	HLA-DPA1*01:03/DPB1*02:01	97.44% (38/39)	Non-Toxin	0.07	Consensus (comb.lib./smm/nn)
370	Matrix Protein	HQALRIFFLSITKLN	HLA-DPA1*02:01/DPB1*14:01	97.44% (38/39)	Non-Toxin	0.5	NetMHCIIpan
371	Matrix Protein	IKINGVISKRLFAQM	HLA-DRB1*11:01	94.87% (37/39)	Non-Toxin	0.48	Consensus (smm/nn/sturniolo)

#	Protein	Peptide	Allele	Conservancy	Toxicity	Percentile rank	Method used
372	Matrix Protein	IPREFMIYDDVFIDN	HLA-DRB3*01:01	38.46% (15/39)	Non-Toxin	0.01	Consensus (comb.lib./smm/nn)
373	Matrix Protein	IPREFMIYDDVFIDN	HLA-DQA1*05:01/DQB1*02:01	38.46% (15/39)	Non-Toxin	0.34	Consensus (comb.lib./smm/nn)
374	Matrix Protein	IPREFMIYDDVFIDN	HLA-DQA1*01:01/DQB1*05:01	38.46% (15/39)	Non-Toxin	0.36	Consensus (comb.lib./smm/nn)
375	Matrix Protein	IYDDVFIDNTGRILK	HLA-DRB3*01:01	94.87% (37/39)	Non-Toxin	0.01	Consensus (comb.lib./smm/nn)
376	Matrix Protein	IYDDVFIDNTGRILK	HLA-DRB1*03:01	94.87% (37/39)	Non-Toxin	0.05	Consensus (smm/nn/sturniolo)
377	Matrix Protein	KHQALRIFFLSITKL	HLA-DPA1*01/DPB1*04:01	97.44% (38/39)	Non-Toxin	0.04	Consensus (comb.lib./smm/nn)
378	Matrix Protein	KHQALRIFFLSITKL	HLA-DPA1*01:03/DPB1*02:01	97.44% (38/39)	Non-Toxin	0.06	Consensus (comb.lib./smm/nn)
379	Matrix Protein	KKIRTIAAYPLGVGK	HLA-DRB1*08:02	97.44% (38/39)	Non-Toxin	0.42	Consensus (smm/nn/sturniolo)
380	Matrix Protein	KNLCFSLMDINPWLN	HLA-DRB3*01:01	94.87% (37/39)	Non-Toxin	0.06	Consensus (comb.lib./smm/nn)
381	Matrix Protein	KRKKIRTIAAYPLGV	HLA-DRB1*08:02	97.44% (38/39)	Non-Toxin	0.36	Consensus (smm/nn/sturniolo)
382	Matrix Protein	KRKKIRTIAAYPLGV	HLA-DRB5*01:01	97.44% (38/39)	Non-Toxin	0.5	Consensus (smm/nn/sturniolo)
383	Matrix Protein	LCFSLMDINPWLNRL	HLA-DRB3*01:01	94.87% (37/39)	Non-Toxin	0.06	Consensus (comb.lib./smm/nn)
384	Matrix Protein	LDKHQALRIFFLSIT	HLA-DPA1*01/DPB1*04:01	97.44% (38/39)	Non-Toxin	0.06	Consensus (comb.lib./smm/nn)
385	Matrix Protein	LDKHQALRIFFLSIT	HLA-DPA1*01:03/DPB1*02:01	97.44% (38/39)	Non-Toxin	0.08	Consensus (comb.lib./smm/nn)
386	Matrix Protein	LEFRRNNAIAFNLLV	HLA-DRB3*02:02	84.62% (33/39)	Non-Toxin	0.08	NetMHCIIpan
387	Matrix Protein	LEFRRNNAIAFNLLV	HLA-DRB3*01:01	84.62% (33/39)	Non-Toxin	0.46	Consensus (comb.lib./smm/nn)
388	Matrix Protein	LEFRRNNAIAFNLLV	HLA-DRB1*13:02	84.62% (33/39)	Non-Toxin	0.48	Consensus (smm/nn/sturniolo)
389	Matrix Protein	LGNFVRRAGKYYSVD	HLA-DRB1*11:01	79.49% (31/39)	Non-Toxin	0.38	Consensus (smm/nn/sturniolo)
390	Matrix Protein	LHIKINGVISKRLFA	HLA-DRB1*07:01	94.87% (37/39)	Non-Toxin	0.4	Consensus (comb.lib./smm/nn)
391	Matrix Protein	LHIKINGVISKRLFA	HLA-DRB1*11:01	94.87% (37/39)	Non-Toxin	0.48	Consensus (smm/nn/sturniolo)
392	Matrix Protein	LHIKINGVISKRLFA	HLA-DRB3*02:02	94.87% (37/39)	Non-Toxin	0.5	NetMHCIIpan
393	Matrix Protein	LHLGNFVRRAGKYYS	HLA-DRB1*11:01	79.49% (31/39)	Non-Toxin	0.38	Consensus (smm/nn/sturniolo)
394	Matrix Protein	LNHLVPWKKVLTSGS	HLA-DRB1*08:02	35.90% (14/39)	Non-Toxin	0.45	Consensus (smm/nn/sturniolo)
395	Matrix Protein	LRIFFLSITKLNDSG	HLA-DPA1*02:01/DPB1*14:01	97.44% (38/39)	Non-Toxin	0.48	NetMHCIIpan
396	Matrix Protein	LSLHIKINGVISKRL	HLA-DRB1*07:01	94.87% (37/39)	Non-Toxin	0.4	Consensus (comb.lib./smm/nn)
397	Matrix Protein	MIYDDVFIDNTGRIL	HLA-DRB3*01:01	94.87% (37/39)	Non-Toxin	0.01	Consensus (comb.lib./smm/nn)
398	Matrix Protein	MIYDDVFIDNTGRIL	HLA-DRB1*03:01	94.87% (37/39)	Non-Toxin	0.06	Consensus (smm/nn/sturniolo)
399	Matrix Protein	MLEFRRNNAIAFNLL	HLA-DRB3*02:02	97.44% (38/39)	Non-Toxin	0.06	NetMHCIIpan
400	Matrix Protein	MLEFRRNNAIAFNLL	HLA-DRB3*01:01	97.44% (38/39)	Non-Toxin	0.22	Consensus (comb.lib./smm/nn)
401	Matrix Protein	MLEFRRNNAIAFNLL	HLA-DRB1*13:02	97.44% (38/39)	Non-Toxin	0.48	Consensus (smm/nn/sturniolo)
402	Matrix Protein	MYLICYGFVEDVERT	HLA-DQA1*01:01/DQB1*05:01	97.44% (38/39)	Non-Toxin	0.28	Consensus (comb.lib./smm/nn)
403	Matrix Protein	NFVRRAGKYYSVDYC	HLA-DRB1*11:01	79.49% (31/39)	Non-Toxin	0.38	Consensus (smm/nn/sturniolo)
404	Matrix Protein	NHLVPWKKVLTSGSI	HLA-DRB1*08:02	35.90% (14/39)	Non-Toxin	0.29	Consensus (smm/nn/sturniolo)
405	Matrix Protein	NLCFSLMDINPWLNR	HLA-DRB3*01:01	94.87% (37/39)	Non-Toxin	0.06	Consensus (comb.lib./smm/nn)
406	Matrix Protein	NYMYLICYGFVEDVE	HLA-DQA1*01:01/DQB1*05:01	97.44% (38/39)	Non-Toxin	0.39	Consensus (comb.lib./smm/nn)
407	Matrix Protein	PREFMIYDDVFIDNT	HLA-DRB3*01:01	94.87% (37/39)	Non-Toxin	0.01	Consensus (comb.lib./smm/nn)
408	Matrix Protein	PREFMIYDDVFIDNT	HLA-DQA1*01:01/DQB1*05:01	94.87% (37/39)	Non-Toxin	0.35	Consensus (comb.lib./smm/nn)
409	Matrix Protein	PREFMIYDDVFIDNT	HLA-DQA1*05:01/DQB1*02:01	94.87% (37/39)	Non-Toxin	0.39	Consensus (comb.lib./smm/nn)
410	Matrix Protein	PRTMLEFRRNNAIAF	HLA-DRB3*01:01	97.44% (38/39)	Non-Toxin	0.3	Consensus (comb.lib./smm/nn)
411	Matrix Protein	PRTMLEFRRNNAIAF	HLA-DRB1*13:02	97.44% (38/39)	Non-Toxin	0.51	Consensus (smm/nn/sturniolo)
412	Matrix Protein	PSIPREFMIYDDVFI	HLA-DRB3*01:01	38.46% (15/39)	Non-Toxin	0.11	Consensus (comb.lib./smm/nn)
413	Matrix Protein	PSIPREFMIYDDVFI	HLA-DQA1*05:01/DQB1*02:01	38.46% (15/39)	Non-Toxin	0.41	Consensus (comb.lib./smm/nn)
414	Matrix Protein	QALRIFFLSITKLND	HLA-DPA1*01/DPB1*04:01	97.44% (38/39)	Non-Toxin	0.19	Consensus (comb.lib./smm/nn)
415	Matrix Protein	QALRIFFLSITKLND	HLA-DPA1*02:01/DPB1*14:01	97.44% (38/39)	Non-Toxin	0.33	NetMHCIIpan
416	Matrix Protein	QKNLCFSLMDINPWL	HLA-DRB3*01:01	94.87% (37/39)	Non-Toxin	0.06	Consensus (comb.lib./smm/nn)
417	Matrix Protein	QLDKHQALRIFFLSI	HLA-DPA1*01/DPB1*04:01	97.44% (38/39)	Non-Toxin	0.06	Consensus (comb.lib./smm/nn)
418	Matrix Protein	QLDKHQALRIFFLSI	HLA-DPA1*01:03/DPB1*02:01	97.44% (38/39)	Non-Toxin	0.09	Consensus (comb.lib./smm/nn)

#	Protein	Peptide	Allele	Conservancy	Toxicity	Percentile rank	Method used
419	Matrix Protein	REFMIYDDVFIDNTG	HLA-DRB3*01:01	94.87% (37/39)	Non-Toxin	0.01	Consensus (comb.lib./smm/nn)
420	Matrix Protein	REFMIYDDVFIDNTG	HLA-DQA1*01:01/DQB1*05:01	94.87% (37/39)	Non-Toxin	0.35	Consensus (comb.lib./smm/nn)
421	Matrix Protein	RKKIRTIAAYPLGVG	HLA-DRB1*08:02	97.44% (38/39)	Non-Toxin	0.36	Consensus (smm/nn/sturniolo)
422	Matrix Protein	RTMLEFRRNNAIAFN	HLA-DRB3*02:02	97.44% (38/39)	Non-Toxin	0.1	NetMHCIIpan
423	Matrix Protein	RTMLEFRRNNAIAFN	HLA-DRB3*01:01	97.44% (38/39)	Non-Toxin	0.27	Consensus (comb.lib./smm/nn)
424	Matrix Protein	SIPREFMIYDDVFID	HLA-DRB3*01:01	38.46% (15/39)	Non-Toxin	0.01	Consensus (comb.lib./smm/nn)
425	Matrix Protein	SIPREFMIYDDVFID	HLA-DQA1*05:01/DQB1*02:01	38.46% (15/39)	Non-Toxin	0.34	Consensus (comb.lib./smm/nn)
426	Matrix Protein	SIPREFMIYDDVFID	HLA-DQA1*01:01/DQB1*05:01	38.46% (15/39)	Non-Toxin	0.43	Consensus (comb.lib./smm/nn)
427	Matrix Protein	SLHIKINGVISKRLF	HLA-DRB1*07:01	94.87% (37/39)	Non-Toxin	0.34	Consensus (comb.lib./smm/nn)
428	Matrix Protein	TMLEFRRNNAIAFNL	HLA-DRB3*02:02	97.44% (38/39)	Non-Toxin	0.06	NetMHCIIpan
429	Matrix Protein	TMLEFRRNNAIAFNL	HLA-DRB3*01:01	97.44% (38/39)	Non-Toxin	0.23	Consensus (comb.lib./smm/nn)
430	Matrix Protein	TMLEFRRNNAIAFNL	HLA-DRB1*13:02	97.44% (38/39)	Non-Toxin	0.48	Consensus (smm/nn/sturniolo)
431	Matrix Protein	YDDVFIDNTGRILKG	HLA-DRB3*01:01	94.87% (37/39)	Non-Toxin	0.01	Consensus (comb.lib./smm/nn)
432	Matrix Protein	YDDVFIDNTGRILKG	HLA-DRB1*03:01	94.87% (37/39)	Non-Toxin	0.06	Consensus (smm/nn/sturniolo)
433	Matrix Protein	YLICYGFVEDVERTP	HLA-DQA1*01:01/DQB1*05:01	97.44% (38/39)	Non-Toxin	0.27	Consensus (comb.lib./smm/nn)
434	Matrix Protein	YMYLICYGFVEDVER	HLA-DQA1*01:01/DQB1*05:01	97.44% (38/39)	Non-Toxin	0.35	Consensus (comb.lib./smm/nn)
435	nucleocapsid	AELAAAVQETSAGRQ	HLA-DQA1*04:01/DQB1*04:02	100.00% (13/13)	Non-Toxin	0.27	Consensus (comb.lib./smm/nn)
436	nucleocapsid	AGGIDQNMANRLGLS	HLA-DRB1*13:02	100.00% (13/13)	Non-Toxin	0.15	Consensus (smm/nn/sturniolo)
437	nucleocapsid	ALIRSLLNDPDIEAV	HLA-DRB3*01:01	100.00% (13/13)	Non-Toxin	0.07	Consensus (comb.lib./smm/nn)
438	nucleocapsid	ALIRSLLNDPDIEAV	HLA-DRB1*04:05	100.00% (13/13)	Non-Toxin	0.19	Consensus (smm/nn/sturniolo)
439	nucleocapsid	AVEIISDIGNYVEET	HLA-DRB3*01:01	100.00% (13/13)	Non-Toxin	0.26	Consensus (comb.lib./smm/nn)
440	nucleocapsid	AVEIISDIGNYVEET	HLA-DRB1*13:02	100.00% (13/13)	Non-Toxin	0.27	Consensus (smm/nn/sturniolo)
441	nucleocapsid	AVIIDVGSMVNGIPV	HLA-DRB1*03:01	100.00% (13/13)	Non-Toxin	0.45	Consensus (smm/nn/sturniolo)
442	nucleocapsid	DIEAVIIDVGSMVNG	HLA-DRB1*03:01	100.00% (13/13)	Non-Toxin	0.45	Consensus (smm/nn/sturniolo)
443	nucleocapsid	DMSTLVSAVITIEAQ	HLA-DQA1*04:01/DQB1*04:02	100.00% (13/13)	Non-Toxin	0.03	Consensus (comb.lib./smm/nn)
444	nucleocapsid	DPDIEAVIIDVGSMV	HLA-DRB1*03:01	100.00% (13/13)	Non-Toxin	0.45	Consensus (smm/nn/sturniolo)
445	nucleocapsid	DQVAELAAAVQETSA	HLA-DQA1*04:01/DQB1*04:02	15.38% (2/13)	Non-Toxin	0.01	Consensus (comb.lib./smm/nn)
446	nucleocapsid	DQVAELAAAVQETSA	HLA-DQA1*03:01/DQB1*03:02	15.38% (2/13)	Non-Toxin	0.03	Consensus (comb.lib./smm/nn)
447	nucleocapsid	EAVIIDVGSMVNGIP	HLA-DRB1*03:01	100.00% (13/13)	Non-Toxin	0.45	Consensus (smm/nn/sturniolo)
448	nucleocapsid	EFQSDLNTIKSLMLL	HLA-DRB1*07:01	100.00% (13/13)	Non-Toxin	0.11	Consensus (comb.lib./smm/nn)
449	nucleocapsid	ELRWELTLFALDVIR	HLA-DQA1*01:01/DQB1*05:01	100.00% (13/13)	Non-Toxin	0.02	Consensus (comb.lib./smm/nn)
450	nucleocapsid	ELRWELTLFALDVIR	HLA-DPA1*01/DPB1*04:01	100.00% (13/13)	Non-Toxin	0.18	Consensus (comb.lib./smm/nn)
451	nucleocapsid	ELTLFALDVIRSPSA	HLA-DQA1*01:01/DQB1*05:01	100.00% (13/13)	Non-Toxin	0.2	Consensus (comb.lib./smm/nn)
452	nucleocapsid	ELTLFALDVIRSPSA	HLA-DRB1*11:01	100.00% (13/13)	Non-Toxin	0.44	Consensus (smm/nn/sturniolo)
453	nucleocapsid	ERPGALIRSLLNDPD	HLA-DRB1*04:05	100.00% (13/13)	Non-Toxin	0.22	Consensus (smm/nn/sturniolo)
454	nucleocapsid	FQSDLNTIKSLMLLY	HLA-DRB1*07:01	100.00% (13/13)	Non-Toxin	0.24	Consensus (comb.lib./smm/nn)
455	nucleocapsid	GALIRSLLNDPDIEA	HLA-DRB3*01:01	100.00% (13/13)	Non-Toxin	0.07	Consensus (comb.lib./smm/nn)
456	nucleocapsid	GALIRSLLNDPDIEA	HLA-DRB1*04:05	100.00% (13/13)	Non-Toxin	0.19	Consensus (smm/nn/sturniolo)
457	nucleocapsid	GGIDQNMANRLGLSS	HLA-DRB1*13:02	100.00% (13/13)	Non-Toxin	0.15	Consensus (smm/nn/sturniolo)
458	nucleocapsid	GGYPLLWSFAMGVAT	HLA-DRB1*09:01	100.00% (13/13)	Non-Toxin	0.44	Consensus (comb.lib./smm/nn)
459	nucleocapsid	GLRITDMSTLVSAVI	HLA-DQA1*04:01/DQB1*04:02	100.00% (13/13)	Non-Toxin	0.45	Consensus (comb.lib./smm/nn)
460	nucleocapsid	GLSSDQVAELAAAVQ	HLA-DQA1*04:01/DQB1*04:02	15.38% (2/13)	Non-Toxin	0.17	Consensus (comb.lib./smm/nn)
461	nucleocapsid	GMAGFFATIRFGLET	HLA-DPA1*01/DPB1*04:01	100.00% (13/13)	Non-Toxin	0.43	Consensus (comb.lib./smm/nn)
462	nucleocapsid	GRAVEIISDIGNYVE	HLA-DRB3*01:01	100.00% (13/13)	Non-Toxin	0.19	Consensus (comb.lib./smm/nn)
463	nucleocapsid	GYPLLWSFAMGVATT	HLA-DRB1*09:01	100.00% (13/13)	Non-Toxin	0.46	Consensus (comb.lib./smm/nn)
464	nucleocapsid	HAGGIDQNMANRLGL	HLA-DRB1*13:02	100.00% (13/13)	Non-Toxin	0.15	Consensus (smm/nn/sturniolo)
465	nucleocapsid	HHAGGIDQNMANRLG	HLA-DRB1*13:02	100.00% (13/13)	Non-Toxin	0.24	Consensus (smm/nn/sturniolo)

#	Protein	Peptide	Allele	Conservancy	Toxicity	Percentile rank	Method used
466	nucleocapsid	IEAVIIDVGSMVNGI	HLA-DRB1*03:01	100.00% (13/13)	Non-Toxin	0.45	Consensus (smm/nn/sturniolo)
467	nucleocapsid	IRIFVPATNSPELRW	HLA-DRB1*04:05	100.00% (13/13)	Non-Toxin	0.11	Consensus (smm/nn/sturniolo)
468	nucleocapsid	IRSLLNDPDIEAVII	HLA-DRB3*01:01	100.00% (13/13)	Non-Toxin	0.05	Consensus (comb.lib./smm/nn)
469	nucleocapsid	ITDMSTLVSAVITIE	HLA-DQA1*04:01/DQB1*04:02	100.00% (13/13)	Non-Toxin	0.05	Consensus (comb.lib./smm/nn)
470	nucleocapsid	KGRAVEIISDIGNYV	HLA-DRB3*01:01	100.00% (13/13)	Non-Toxin	0.19	Consensus (comb.lib./smm/nn)
471	nucleocapsid	KIRIFVPATNSPELR	HLA-DRB1*04:05	100.00% (13/13)	Non-Toxin	0.12	Consensus (smm/nn/sturniolo)
472	nucleocapsid	LGLSSDQVAELAAAV	HLA-DQA1*04:01/DQB1*04:02	15.38% (2/13)	Non-Toxin	0.16	Consensus (comb.lib./smm/nn)
473	nucleocapsid	LIRSLLNDPDIEAVI	HLA-DRB3*01:01	100.00% (13/13)	Non-Toxin	0.06	Consensus (comb.lib./smm/nn)
474	nucleocapsid	LLWSFAMGVATTIDR	HLA-DRB1*09:01	100.00% (13/13)	Non-Toxin	0.01	Consensus (comb.lib./smm/nn)
475	nucleocapsid	LRITDMSTLVSAVIT	HLA-DQA1*04:01/DQB1*04:02	100.00% (13/13)	Non-Toxin	0.43	Consensus (comb.lib./smm/nn)
476	nucleocapsid	LRWELTLFALDVIRS	HLA-DQA1*01:01/DQB1*05:01	100.00% (13/13)	Non-Toxin	0.02	Consensus (comb.lib./smm/nn)
477	nucleocapsid	LRWELTLFALDVIRS	HLA-DPA1*01/DPB1*04:01	100.00% (13/13)	Non-Toxin	0.21	Consensus (comb.lib./smm/nn)
478	nucleocapsid	LSSDQVAELAAAVQE	HLA-DQA1*04:01/DQB1*04:02	15.38% (2/13)	Non-Toxin	0.01	Consensus (comb.lib./smm/nn)
479	nucleocapsid	LSSDQVAELAAAVQE	HLA-DQA1*03:01/DQB1*03:02	15.38% (2/13)	Non-Toxin	0.02	Consensus (comb.lib./smm/nn)
480	nucleocapsid	LTEMRNLLSQSLSVR	HLA-DRB4*01:01	100.00% (13/13)	Non-Toxin	0.39	Consensus (comb.lib./smm/nn)
481	nucleocapsid	LTLFALDVIRSPSAA	HLA-DQA1*01:01/DQB1*05:01	100.00% (13/13)	Non-Toxin	0.22	Consensus (comb.lib./smm/nn)
482	nucleocapsid	LTLFALDVIRSPSAA	HLA-DRB1*11:01	100.00% (13/13)	Non-Toxin	0.42	Consensus (smm/nn/sturniolo)
483	nucleocapsid	LTNSLLNLRSRLAAK	HLA-DRB1*15:01	100.00% (13/13)	Non-Toxin	0.49	Consensus (smm/nn/sturniolo)
484	nucleocapsid	LTTKIRIFVPATNSP	HLA-DRB1*08:02	100.00% (13/13)	Non-Toxin	0.37	Consensus (smm/nn/sturniolo)
485	nucleocapsid	LVSAVITIEAQIWIL	HLA-DQA1*03:01/DQB1*03:02	100.00% (13/13)	Non-Toxin	0.18	Consensus (comb.lib./smm/nn)
486	nucleocapsid	LWSFAMGVATTIDRS	HLA-DRB1*09:01	100.00% (13/13)	Non-Toxin	0.01	Consensus (comb.lib./smm/nn)
487	nucleocapsid	MAGFFATIRFGLETR	HLA-DPA1*01/DPB1*04:01	100.00% (13/13)	Non-Toxin	0.43	Consensus (comb.lib./smm/nn)
488	nucleocapsid	MSTLVSAVITIEAQI	HLA-DQA1*03:01/DQB1*03:02	100.00% (13/13)	Non-Toxin	0.21	Consensus (comb.lib./smm/nn)
489	nucleocapsid	NEFQSDLNTIKSLML	HLA-DRB1*07:01	100.00% (13/13)	Non-Toxin	0.1	Consensus (comb.lib./smm/nn)
490	nucleocapsid	NEFQSDLNTIKSLML	HLA-DRB1*04:01	100.00% (13/13)	Non-Toxin	0.38	Consensus (smm/nn/sturniolo)
491	nucleocapsid	NSLLNLRSRLAAKAA	HLA-DRB1*15:01	100.00% (13/13)	Non-Toxin	0.51	Consensus (smm/nn/sturniolo)
492	nucleocapsid	PDIEAVIIDVGSMVN	HLA-DRB1*03:01	100.00% (13/13)	Non-Toxin	0.45	Consensus (smm/nn/sturniolo)
493	nucleocapsid	PELRWELTLFALDVI	HLA-DQA1*01:01/DQB1*05:01	100.00% (13/13)	Non-Toxin	0.02	Consensus (comb.lib./smm/nn)
494	nucleocapsid	PELRWELTLFALDVI	HLA-DPA1*01/DPB1*04:01	100.00% (13/13)	Non-Toxin	0.16	Consensus (comb.lib./smm/nn)
495	nucleocapsid	PGALIRSLLNDPDIE	HLA-DRB1*04:05	100.00% (13/13)	Non-Toxin	0.19	Consensus (smm/nn/sturniolo)
496	nucleocapsid	PLLWSFAMGVATTID	HLA-DRB1*09:01	100.00% (13/13)	Non-Toxin	0.01	Consensus (comb.lib./smm/nn)
497	nucleocapsid	QVAELAAAVQETSAG	HLA-DQA1*04:01/DQB1*04:02	100.00% (13/13)	Non-Toxin	0.01	Consensus (comb.lib./smm/nn)
498	nucleocapsid	QVAELAAAVQETSAG	HLA-DQA1*03:01/DQB1*03:02	100.00% (13/13)	Non-Toxin	0.07	Consensus (comb.lib./smm/nn)
499	nucleocapsid	RAVEIISDIGNYVEE	HLA-DRB3*01:01	100.00% (13/13)	Non-Toxin	0.22	Consensus (comb.lib./smm/nn)
500	nucleocapsid	RAVEIISDIGNYVEE	HLA-DRB1*13:02	100.00% (13/13)	Non-Toxin	0.33	Consensus (smm/nn/sturniolo)
501	nucleocapsid	RHHAGGIDQNMANRL	HLA-DRB1*13:02	100.00% (13/13)	Non-Toxin	0.33	Consensus (smm/nn/sturniolo)
502	nucleocapsid	RIFVPATNSPELRWE	HLA-DRB1*04:05	100.00% (13/13)	Non-Toxin	0.27	Consensus (smm/nn/sturniolo)
503	nucleocapsid	RITDMSTLVSAVITI	HLA-DQA1*04:01/DQB1*04:02	100.00% (13/13)	Non-Toxin	0.4	Consensus (comb.lib./smm/nn)
504	nucleocapsid	RLTNSLLNLRSRLAA	HLA-DRB1*15:01	100.00% (13/13)	Non-Toxin	0.49	Consensus (smm/nn/sturniolo)
505	nucleocapsid	RPGALIRSLLNDPDI	HLA-DRB1*04:05	100.00% (13/13)	Non-Toxin	0.19	Consensus (smm/nn/sturniolo)
506	nucleocapsid	RWELTLFALDVIRSP	HLA-DQA1*01:01/DQB1*05:01	100.00% (13/13)	Non-Toxin	0.02	Consensus (comb.lib./smm/nn)
507	nucleocapsid	SDQVAELAAAVQETS	HLA-DQA1*04:01/DQB1*04:02	15.38% (2/13)	Non-Toxin	0.01	Consensus (comb.lib./smm/nn)
508	nucleocapsid	SDQVAELAAAVQETS	HLA-DQA1*03:01/DQB1*03:02	15.38% (2/13)	Non-Toxin	0.03	Consensus (comb.lib./smm/nn)
509	nucleocapsid	SKGKTPFVDSRAYGL	HLA-DRB1*07:01	100.00% (13/13)	Non-Toxin	0.38	Consensus (comb.lib./smm/nn)
510	nucleocapsid	SSDQVAELAAAVQET	HLA-DQA1*04:01/DQB1*04:02	15.38% (2/13)	Non-Toxin	0.01	Consensus (comb.lib./smm/nn)
511	nucleocapsid	SSDQVAELAAAVQET	HLA-DQA1*03:01/DQB1*03:02	15.38% (2/13)	Non-Toxin	0.02	Consensus (comb.lib./smm/nn)
512	nucleocapsid	STLVSAVITIEAQIW	HLA-DQA1*03:01/DQB1*03:02	100.00% (13/13)	Non-Toxin	0.18	Consensus (comb.lib./smm/nn)

#	Protein	Peptide	Allele	Conservancy	Toxicity	Percentile rank	Method used
513	nucleocapsid	SVTFKREMSISSLAN	HLA-DRB1*04:01	0.00% (0/13)	Non-Toxin	0.49	Consensus (smm/nn/sturniolo)
514	nucleocapsid	TDMSTLVSAVITIEA	HLA-DQA1*04:01/DQB1*04:02	100.00% (13/13)	Non-Toxin	0.07	Consensus (comb.lib./smm/nn)
515	nucleocapsid	TEMRNLLSQSLSVRK	HLA-DRB4*01:01	100.00% (13/13)	Non-Toxin	0.32	Consensus (comb.lib./smm/nn)
516	nucleocapsid	TKIRIFVPATNSPEL	HLA-DRB1*04:05	100.00% (13/13)	Non-Toxin	0.16	Consensus (smm/nn/sturniolo)
517	nucleocapsid	TKIRIFVPATNSPEL	HLA-DRB1*08:02	100.00% (13/13)	Non-Toxin	0.35	Consensus (smm/nn/sturniolo)
518	nucleocapsid	TLFALDVIRSPSAAE	HLA-DRB1*11:01	100.00% (13/13)	Non-Toxin	0.45	Consensus (smm/nn/sturniolo)
519	nucleocapsid	TLTTKIRIFVPATNS	HLA-DRB1*08:02	100.00% (13/13)	Non-Toxin	0.37	Consensus (smm/nn/sturniolo)
520	nucleocapsid	TLVSAVITIEAQIWI	HLA-DQA1*03:01/DQB1*03:02	100.00% (13/13)	Non-Toxin	0.19	Consensus (comb.lib./smm/nn)
521	nucleocapsid	TNSLLNLRSRLAAKA	HLA-DRB1*15:01	100.00% (13/13)	Non-Toxin	0.49	Consensus (smm/nn/sturniolo)
522	nucleocapsid	TTKIRIFVPATNSPE	HLA-DRB1*08:02	100.00% (13/13)	Non-Toxin	0.29	Consensus (smm/nn/sturniolo)
523	nucleocapsid	TTKIRIFVPATNSPE	HLA-DRB1*04:05	100.00% (13/13)	Non-Toxin	0.34	Consensus (smm/nn/sturniolo)
524	nucleocapsid	VAELAAAVQETSAGR	HLA-DQA1*04:01/DQB1*04:02	100.00% (13/13)	Non-Toxin	0.12	Consensus (comb.lib./smm/nn)
525	nucleocapsid	VEIISDIGNYVEETG	HLA-DRB1*13:02	100.00% (13/13)	Non-Toxin	0.34	Consensus (smm/nn/sturniolo)
526	nucleocapsid	VIIDVGSMVNGIPVM	HLA-DRB1*03:01	100.00% (13/13)	Non-Toxin	0.45	Consensus (smm/nn/sturniolo)
527	nucleocapsid	WELTLFALDVIRSPS	HLA-DQA1*01:01/DQB1*05:01	100.00% (13/13)	Non-Toxin	0.02	Consensus (comb.lib./smm/nn)
528	nucleocapsid	WSFAMGVATTIDRSM	HLA-DRB1*09:01	100.00% (13/13)	Non-Toxin	0.01	Consensus (comb.lib./smm/nn)
529	nucleocapsid	YPLLWSFAMGVATTI	HLA-DRB1*09:01	100.00% (13/13)	Non-Toxin	0.02	Consensus (comb.lib./smm/nn)
530	Phosphoprotein	AEGSDDIQLDPVVTD	HLA-DRB1*03:01	100.00% (27/27)	Non-Toxin	0.13	Consensus (smm/nn/sturniolo)
531	Phosphoprotein	CLVSDAKMLSYAPEI	HLA-DRB1*03:01	59.26% (16/27)	Non-Toxin	0.02	Consensus (smm/nn/sturniolo)
532	Phosphoprotein	DDIQLDPVVTDVVYH	HLA-DRB1*03:01	100.00% (27/27)	Non-Toxin	0.13	Consensus (smm/nn/sturniolo)
533	Phosphoprotein	DIQLDPVVTDVVYHD	HLA-DRB3*01:01	100.00% (27/27)	Non-Toxin	0.02	Consensus (comb.lib./smm/nn)
534	Phosphoprotein	DIQLDPVVTDVVYHD	HLA-DRB1*03:01	100.00% (27/27)	Non-Toxin	0.27	Consensus (smm/nn/sturniolo)
535	Phosphoprotein	DPVVTDVVYHDHGGE	HLA-DRB3*01:01	100.00% (27/27)	Non-Toxin	0.02	Consensus (comb.lib./smm/nn)
536	Phosphoprotein	DSIKLYTSDDEEADQ	HLA-DRB3*01:01	100.00% (27/27)	Non-Toxin	0.28	Consensus (comb.lib./smm/nn)
537	Phosphoprotein	EGSDDIQLDPVVTDV	HLA-DRB1*03:01	100.00% (27/27)	Non-Toxin	0.13	Consensus (smm/nn/sturniolo)
538	Phosphoprotein	EQQSLFSFDNVKNFR	HLA-DRB3*01:01	100.00% (27/27)	Non-Toxin	0.14	Consensus (comb.lib./smm/nn)
539	Phosphoprotein	FSFDNVKNFRDGSLT	HLA-DRB3*01:01	100.00% (27/27)	Non-Toxin	0.01	Consensus (comb.lib./smm/nn)
540	Phosphoprotein	GHLVSMMIMIPGKGK	HLA-DRB5*01:01	100.00% (27/27)	Non-Toxin	0.27	Consensus (smm/nn/sturniolo)
541	Phosphoprotein	GNVCLVSDAKMLSYA	HLA-DRB1*03:01	59.26% (16/27)	Non-Toxin	0.01	Consensus (smm/nn/sturniolo)
542	Phosphoprotein	GSDDIQLDPVVTDVV	HLA-DRB1*03:01	100.00% (27/27)	Non-Toxin	0.13	Consensus (smm/nn/sturniolo)
543	Phosphoprotein	GVINSIKLINLDMRL	HLA-DRB4*01:01	100.00% (27/27)	Non-Toxin	0.44	Consensus (comb.lib./smm/nn)
544	Phosphoprotein	HLVSMMIMIPGKGKG	HLA-DRB5*01:01	100.00% (27/27)	Non-Toxin	0.28	Consensus (smm/nn/sturniolo)
545	Phosphoprotein	IEGHLVSMMIMIPGK	HLA-DRB4*01:01	100.00% (27/27)	Non-Toxin	0.47	Consensus (comb.lib./smm/nn)
546	Phosphoprotein	IKLINLDMRLNHIEE	HLA-DRB1*03:01	100.00% (27/27)	Non-Toxin	0.1	Consensus (smm/nn/sturniolo)
547	Phosphoprotein	IKLYTSDDEEADQLE	HLA-DRB3*01:01	100.00% (27/27)	Non-Toxin	0.27	Consensus (comb.lib./smm/nn)
548	Phosphoprotein	INLDMRLNHIEEQVK	HLA-DRB1*03:01	100.00% (27/27)	Non-Toxin	0.35	Consensus (smm/nn/sturniolo)
549	Phosphoprotein	INSIKLINLDMRLNH	HLA-DRB1*03:01	100.00% (27/27)	Non-Toxin	0.33	Consensus (smm/nn/sturniolo)
550	Phosphoprotein	IQLDPVVTDVVYHDH	HLA-DRB3*01:01	100.00% (27/27)	Non-Toxin	0.02	Consensus (comb.lib./smm/nn)
551	Phosphoprotein	IQLDPVVTDVVYHDH	HLA-DRB1*03:01	100.00% (27/27)	Non-Toxin	0.26	Consensus (smm/nn/sturniolo)
552	Phosphoprotein	KLINLDMRLNHIEEQ	HLA-DRB1*03:01	100.00% (27/27)	Non-Toxin	0.31	Consensus (smm/nn/sturniolo)
553	Phosphoprotein	KLYTSDDEEADQLEF	HLA-DRB3*01:01	100.00% (27/27)	Non-Toxin	0.28	Consensus (comb.lib./smm/nn)
554	Phosphoprotein	LDPVVTDVVYHDHGG	HLA-DRB3*01:01	100.00% (27/27)	Non-Toxin	0.02	Consensus (comb.lib./smm/nn)
555	Phosphoprotein	LDSIKLYTSDDEEAD	HLA-DRB3*01:01	100.00% (27/27)	Non-Toxin	0.27	Consensus (comb.lib./smm/nn)
556	Phosphoprotein	LEFEDEFAGSSSEVI	HLA-DRB1*07:01	100.00% (27/27)	Non-Toxin	0.45	Consensus (comb.lib./smm/nn)
557	Phosphoprotein	LEQQSLFSFDNVKNF	HLA-DRB3*01:01	100.00% (27/27)	Non-Toxin	0.15	Consensus (comb.lib./smm/nn)
558	Phosphoprotein	LFSFDNVKNFRDGSL	HLA-DRB3*01:01	100.00% (27/27)	Non-Toxin	0.01	Consensus (comb.lib./smm/nn)
559	Phosphoprotein	LINLDMRLNHIEEQV	HLA-DRB1*03:01	100.00% (27/27)	Non-Toxin	0.35	Consensus (smm/nn/sturniolo)

#	Protein	Peptide	Allele	Conservancy	Toxicity	Percentile rank	Method used
560	Phosphoprotein	LTNEPYGAAVQLRED	HLA-DQA1*04:01/DQB1*04:02	62.96% (17/27)	Non-Toxin	0.21	Consensus (comb.lib./smm/nn)
561	Phosphoprotein	LVSDAKMLSYAPEIA	HLA-DRB1*03:01	59.26% (16/27)	Non-Toxin	0.02	Consensus (smm/nn/sturniolo)
562	Phosphoprotein	LVSMMIMIPGKGKGE	HLA-DRB5*01:01	100.00% (27/27)	Non-Toxin	0.28	Consensus (smm/nn/sturniolo)
563	Phosphoprotein	MPSDDFSNTFFPHDT	HLA-DPA1*01/DPB1*04:01	96.30% (26/27)	Non-Toxin	0.49	Consensus (comb.lib./smm/nn)
564	Phosphoprotein	NEPYGAAVQLREDLI	HLA-DQA1*04:01/DQB1*04:02	62.96% (17/27)	Non-Toxin	0.39	Consensus (comb.lib./smm/nn)
565	Phosphoprotein	NGNVCLVSDAKMLSY	HLA-DRB1*03:01	59.26% (16/27)	Non-Toxin	0.01	Consensus (smm/nn/sturniolo)
566	Phosphoprotein	NNGNVCLVSDAKMLS	HLA-DRB1*03:01	62.96% (17/27)	Non-Toxin	0.01	Consensus (smm/nn/sturniolo)
567	Phosphoprotein	NSIKLINLDMRLNHI	HLA-DRB1*03:01	100.00% (27/27)	Non-Toxin	0.16	Consensus (smm/nn/sturniolo)
568	Phosphoprotein	NVCLVSDAKMLSYAP	HLA-DRB1*03:01	59.26% (16/27)	Non-Toxin	0.01	Consensus (smm/nn/sturniolo)
569	Phosphoprotein	PSDDFSNTFFPHDTD	HLA-DPA1*01/DPB1*04:01	96.30% (26/27)	Non-Toxin	0.51	Consensus (comb.lib./smm/nn)
570	Phosphoprotein	PVVTDVVYHDHGGEC	HLA-DRB3*01:01	100.00% (27/27)	Non-Toxin	0.2	Consensus (comb.lib./smm/nn)
571	Phosphoprotein	QLDPVVTDVVYHDHG	HLA-DRB3*01:01	100.00% (27/27)	Non-Toxin	0.02	Consensus (comb.lib./smm/nn)
572	Phosphoprotein	QQSLFSFDNVKNFRD	HLA-DRB3*01:01	100.00% (27/27)	Non-Toxin	0.01	Consensus (comb.lib./smm/nn)
573	Phosphoprotein	QSLFSFDNVKNFRDG	HLA-DRB3*01:01	100.00% (27/27)	Non-Toxin	0.01	Consensus (comb.lib./smm/nn)
574	Phosphoprotein	SDDFSNTFFPHDTDR	HLA-DPA1*01/DPB1*04:01	96.30% (26/27)	Non-Toxin	0.5	Consensus (comb.lib./smm/nn)
575	Phosphoprotein	SDDIQLDPVVTDVVY	HLA-DRB1*03:01	100.00% (27/27)	Non-Toxin	0.13	Consensus (smm/nn/sturniolo)
576	Phosphoprotein	SIKLINLDMRLNHIE	HLA-DRB1*03:01	100.00% (27/27)	Non-Toxin	0.11	Consensus (smm/nn/sturniolo)
577	Phosphoprotein	SIKLYTSDDEEADQL	HLA-DRB3*01:01	100.00% (27/27)	Non-Toxin	0.27	Consensus (comb.lib./smm/nn)
578	Phosphoprotein	SLFSFDNVKNFRDGS	HLA-DRB3*01:01	100.00% (27/27)	Non-Toxin	0.01	Consensus (comb.lib./smm/nn)
579	Phosphoprotein	SLTNEPYGAAVQLRE	HLA-DQA1*04:01/DQB1*04:02	62.96% (17/27)	Non-Toxin	0.19	Consensus (comb.lib./smm/nn)
580	Phosphoprotein	SMMIMIPGKGKGERK	HLA-DRB5*01:01	100.00% (27/27)	Non-Toxin	0.3	Consensus (smm/nn/sturniolo)
581	Phosphoprotein	SSEVIVGISPEDEEP	HLA-DQA1*04:01/DQB1*04:02	62.96% (17/27)	Non-Toxin	0.39	Consensus (comb.lib./smm/nn)
582	Phosphoprotein	SSSEVIVGISPEDEE	HLA-DQA1*04:01/DQB1*04:02	62.96% (17/27)	Non-Toxin	0.29	Consensus (comb.lib./smm/nn)
583	Phosphoprotein	TIEGHLVSMMIMIPG	HLA-DRB4*01:01	100.00% (27/27)	Non-Toxin	0.48	Consensus (comb.lib./smm/nn)
584	Phosphoprotein	TNEPYGAAVQLREDL	HLA-DQA1*04:01/DQB1*04:02	62.96% (17/27)	Non-Toxin	0.25	Consensus (comb.lib./smm/nn)
585	Phosphoprotein	VCLVSDAKMLSYAPE	HLA-DRB1*03:01	59.26% (16/27)	Non-Toxin	0.01	Consensus (smm/nn/sturniolo)
586	Phosphoprotein	VSMMIMIPGKGKGER	HLA-DRB5*01:01	100.00% (27/27)	Non-Toxin	0.27	Consensus (smm/nn/sturniolo)
587	Phosphoprotein	VVTDVVYHDHGGECT	HLA-DRB3*01:01	100.00% (27/27)	Non-Toxin	0.21	Consensus (comb.lib./smm/nn)
588	Polymerase	ASFLMDRRVILPRAA	HLA-DRB3*01:01	100.00% (20/20)	Non-Toxin	0.01	Consensus (comb.lib./smm/nn)
589	Polymerase	ASFLMDRRVILPRAA	HLA-DRB1*03:01	100.00% (20/20)	Non-Toxin	0.01	Consensus (smm/nn/sturniolo)
590	Polymerase	ASQRVNIDLDVLKAI	HLA-DRB3*01:01	100.00% (20/20)	Non-Toxin	0.01	Consensus (comb.lib./smm/nn)
591	Polymerase	CINILKVIQQLLIST	HLA-DRB4*01:01	100.00% (20/20)	Non-Toxin	0.01	Consensus (comb.lib./smm/nn)
592	Polymerase	CLNWRYESMAIFAER	HLA-DRB3*01:01	100.00% (20/20)	Non-Toxin	0.01	Consensus (comb.lib./smm/nn)
593	Polymerase	DDLKKYYQIDQPFFV	HLA-DRB3*01:01	100.00% (20/20)	Non-Toxin	0.01	Consensus (comb.lib./smm/nn)
594	Polymerase	DLELASFLMDRRVIL	HLA-DRB3*01:01	100.00% (20/20)	Non-Toxin	0.01	Consensus (comb.lib./smm/nn)
595	Polymerase	DLELASFLMDRRVIL	HLA-DRB1*03:01	100.00% (20/20)	Non-Toxin	0.02	Consensus (smm/nn/sturniolo)
596	Polymerase	DLKKYYQIDQPFFVP	HLA-DRB3*01:01	100.00% (20/20)	Non-Toxin	0.01	Consensus (comb.lib./smm/nn)
597	Polymerase	DLSMYMKDKALSPIK	HLA-DRB3*01:01	100.00% (20/20)	Non-Toxin	0.01	Consensus (comb.lib./smm/nn)
598	Polymerase	DNIHLLAEFFSFFRT	HLA-DPA1*01:03/DPB1*02:01	100.00% (20/20)	Non-Toxin	0.01	Consensus (comb.lib./smm/nn)
599	Polymerase	DQKIRSMFIDDLLSI	HLA-DRB3*01:01	100.00% (20/20)	Non-Toxin	0.01	Consensus (comb.lib./smm/nn)
600	Polymerase	DSDLSMYMKDKALSP	HLA-DRB3*01:01	100.00% (20/20)	Non-Toxin	0.01	Consensus (comb.lib./smm/nn)
601	Polymerase	DTIIIMLNEAMNYFD	HLA-DRB3*01:01	100.00% (20/20)	Non-Toxin	0.01	Consensus (comb.lib./smm/nn)
602	Polymerase	DYNQFLILNKLLSNR	HLA-DRB1*11:01	100.00% (20/20)	Non-Toxin	0.01	Consensus (smm/nn/sturniolo)
603	Polymerase	ELASFLMDRRVILPR	HLA-DRB3*01:01	100.00% (20/20)	Non-Toxin	0.01	Consensus (comb.lib./smm/nn)
604	Polymerase	ELASFLMDRRVILPR	HLA-DRB1*03:01	100.00% (20/20)	Non-Toxin	0.01	Consensus (smm/nn/sturniolo)
605	Polymerase	ETLTLDVTSPISNNL	HLA-DRB3*01:01	100.00% (20/20)	Non-Toxin	0.01	Consensus (comb.lib./smm/nn)
606	Polymerase	FCLNWRYESMAIFAE	HLA-DRB3*01:01	100.00% (20/20)	Non-Toxin	0.01	Consensus (comb.lib./smm/nn)

#	Protein	Peptide	Allele	Conservancy	Toxicity	Percentile rank	Method used
607	Polymerase	FLILNKLLSNRRQND	HLA-DRB1*11:01	100.00% (20/20)	Non-Toxin	0.01	Consensus (smm/nn/sturniolo)
608	Polymerase	FSINETLTLDVTSPI	HLA-DRB3*01:01	100.00% (20/20)	Non-Toxin	0.01	Consensus (comb.lib./smm/nn)
609	Polymerase	FVIFYASLTYLRRGI	HLA-DPA1*02:01/DPB1*14:01	100.00% (20/20)	Non-Toxin	0.01	NetMHCIIpan
610	Polymerase	GYCINILKVIQQLLI	HLA-DRB4*01:01	100.00% (20/20)	Non-Toxin	0.02	Consensus (comb.lib./smm/nn)
611	Polymerase	HDYNQFLILNKLLSN	HLA-DRB1*11:01	100.00% (20/20)	Non-Toxin	0.01	Consensus (smm/nn/sturniolo)
612	Polymerase	HHDYNQFLILNKLLS	HLA-DRB1*11:01	100.00% (20/20)	Non-Toxin	0.01	Consensus (smm/nn/sturniolo)
613	Polymerase	HHKPPWIIDLNPQEK	HLA-DRB3*01:01	100.00% (20/20)	Non-Toxin	0.02	Consensus (comb.lib./smm/nn)
614	Polymerase	HKPPWIIDLNPQEKI	HLA-DRB3*01:01	100.00% (20/20)	Non-Toxin	0.02	Consensus (comb.lib./smm/nn)
615	Polymerase	IDNIHLLAEFFSFFR	HLA-DPA1*01:03/DPB1*02:01	100.00% (20/20)	Non-Toxin	0.01	Consensus (comb.lib./smm/nn)
616	Polymerase	IHLLAEFFSFFRTFG	HLA-DPA1*01:03/DPB1*02:01	100.00% (20/20)	Non-Toxin	0.01	Consensus (comb.lib./smm/nn)
617	Polymerase	IIIMLNEAMNYFDDN	HLA-DRB3*01:01	100.00% (20/20)	Non-Toxin	0.01	Consensus (comb.lib./smm/nn)
618	Polymerase	ILKVIQQLLISTEFS	HLA-DRB4*01:01	100.00% (20/20)	Non-Toxin	0.01	Consensus (comb.lib./smm/nn)
619	Polymerase	INETLTLDVTSPISN	HLA-DRB3*01:01	100.00% (20/20)	Non-Toxin	0.01	Consensus (comb.lib./smm/nn)
620	Polymerase	INILKVIQQLLISTE	HLA-DRB4*01:01	100.00% (20/20)	Non-Toxin	0.01	Consensus (comb.lib./smm/nn)
621	Polymerase	IRSMFIDDLLSILNI	HLA-DRB3*01:01	100.00% (20/20)	Non-Toxin	0.01	Consensus (comb.lib./smm/nn)
622	Polymerase	KIRSMFIDDLLSILN	HLA-DRB3*01:01	100.00% (20/20)	Non-Toxin	0.01	Consensus (comb.lib./smm/nn)
623	Polymerase	KKYYQIDQPFFVPTK	HLA-DRB3*01:01	100.00% (20/20)	Non-Toxin	0.01	Consensus (comb.lib./smm/nn)
624	Polymerase	KPPWIIDLNPQEKIC	HLA-DRB3*01:01	100.00% (20/20)	Non-Toxin	0.02	Consensus (comb.lib./smm/nn)
625	Polymerase	KYYQIDQPFFVPTKI	HLA-DRB3*01:01	100.00% (20/20)	Non-Toxin	0.01	Consensus (comb.lib./smm/nn)
626	Polymerase	LASFLMDRRVILPRA	HLA-DRB3*01:01	100.00% (20/20)	Non-Toxin	0.01	Consensus (comb.lib./smm/nn)
627	Polymerase	LASFLMDRRVILPRA	HLA-DRB1*03:01	100.00% (20/20)	Non-Toxin	0.01	Consensus (smm/nn/sturniolo)
628	Polymerase	LDFVIFYASLTYLRR	HLA-DPA1*02:01/DPB1*14:01	100.00% (20/20)	Non-Toxin	0.01	NetMHCIIpan
629	Polymerase	LELASFLMDRRVILP	HLA-DRB3*01:01	100.00% (20/20)	Non-Toxin	0.01	Consensus (comb.lib./smm/nn)
630	Polymerase	LELASFLMDRRVILP	HLA-DRB1*03:01	100.00% (20/20)	Non-Toxin	0.01	Consensus (smm/nn/sturniolo)
631	Polymerase	LKKYYQIDQPFFVPT	HLA-DRB3*01:01	100.00% (20/20)	Non-Toxin	0.01	Consensus (comb.lib./smm/nn)
632	Polymerase	LNWRYESMAIFAERL	HLA-DRB3*01:01	100.00% (20/20)	Non-Toxin	0.01	Consensus (comb.lib./smm/nn)
633	Polymerase	LSMYMKDKALSPIKD	HLA-DRB3*01:01	100.00% (20/20)	Non-Toxin	0.01	Consensus (comb.lib./smm/nn)
634	Polymerase	MFIDDLLSILNIDNI	HLA-DRB3*01:01	100.00% (20/20)	Non-Toxin	0.01	Consensus (comb.lib./smm/nn)
635	Polymerase	NETLTLDVTSPISNN	HLA-DRB3*01:01	100.00% (20/20)	Non-Toxin	0.01	Consensus (comb.lib./smm/nn)
636	Polymerase	NHHKPPWIIDLNPQE	HLA-DRB3*01:01	100.00% (20/20)	Non-Toxin	0.02	Consensus (comb.lib./smm/nn)
637	Polymerase	NIDNIHLLAEFFSFF	HLA-DPA1*01:03/DPB1*02:01	100.00% (20/20)	Non-Toxin	0.01	Consensus (comb.lib./smm/nn)
638	Polymerase	NIHLLAEFFSFFRTF	HLA-DPA1*01:03/DPB1*02:01	100.00% (20/20)	Non-Toxin	0.01	Consensus (comb.lib./smm/nn)
639	Polymerase	NILKVIQQLLISTEF	HLA-DRB4*01:01	100.00% (20/20)	Non-Toxin	0.01	Consensus (comb.lib./smm/nn)
640	Polymerase	NQFLILNKLLSNRRQ	HLA-DRB1*11:01	100.00% (20/20)	Non-Toxin	0.01	Consensus (smm/nn/sturniolo)
641	Polymerase	NWRYESMAIFAERLD	HLA-DRB3*01:01	100.00% (20/20)	Non-Toxin	0.01	Consensus (comb.lib./smm/nn)
642	Polymerase	PPWIIDLNPQEKICV	HLA-DRB3*01:01	100.00% (20/20)	Non-Toxin	0.02	Consensus (comb.lib./smm/nn)
643	Polymerase	QFLILNKLLSNRRQN	HLA-DRB1*11:01	100.00% (20/20)	Non-Toxin	0.01	Consensus (smm/nn/sturniolo)
644	Polymerase	QKIRSMFIDDLLSIL	HLA-DRB3*01:01	100.00% (20/20)	Non-Toxin	0.01	Consensus (comb.lib./smm/nn)
645	Polymerase	QRVNIDLDVLKAITP	HLA-DRB3*01:01	100.00% (20/20)	Non-Toxin	0.01	Consensus (comb.lib./smm/nn)
646	Polymerase	RDTIIIMLNEAMNYF	HLA-DRB3*01:01	100.00% (20/20)	Non-Toxin	0.01	Consensus (comb.lib./smm/nn)
647	Polymerase	RKVLILDFRSKLMTK	HLA-DRB1*03:01	100.00% (20/20)	Non-Toxin	0.01	Consensus (smm/nn/sturniolo)
648	Polymerase	RSMFIDDLLSILNID	HLA-DRB3*01:01	100.00% (20/20)	Non-Toxin	0.01	Consensus (comb.lib./smm/nn)
649	Polymerase	RVNIDLDVLKAITPV	HLA-DRB3*01:01	100.00% (20/20)	Non-Toxin	0.01	Consensus (comb.lib./smm/nn)
650	Polymerase	SDLSMYMKDKALSPI	HLA-DRB3*01:01	100.00% (20/20)	Non-Toxin	0.01	Consensus (comb.lib./smm/nn)
651	Polymerase	SFLMDRRVILPRAAH	HLA-DRB3*01:01	100.00% (20/20)	Non-Toxin	0.01	Consensus (comb.lib./smm/nn)
652	Polymerase	SINETLTLDVTSPIS	HLA-DRB3*01:01	100.00% (20/20)	Non-Toxin	0.01	Consensus (comb.lib./smm/nn)
653	Polymerase	SMFIDDLLSILNIDN	HLA-DRB3*01:01	100.00% (20/20)	Non-Toxin	0.01	Consensus (comb.lib./smm/nn)

#	Protein	Peptide	Allele	Conservancy	Toxicity	Percentile rank	Method used
654	Polymerase	SMYMKDKALSPIKDE	HLA-DRB3*01:01	100.00% (20/20)	Non-Toxin	0.01	Consensus (comb.lib./smm/nn)
655	Polymerase	SQRVNIDLDVLKAIT	HLA-DRB3*01:01	100.00% (20/20)	Non-Toxin	0.01	Consensus (comb.lib./smm/nn)
656	Polymerase	TIIIMLNEAMNYFDD	HLA-DRB3*01:01	100.00% (20/20)	Non-Toxin	0.01	Consensus (comb.lib./smm/nn)
657	Polymerase	VNIDLDVLKAITPVS	HLA-DRB3*01:01	100.00% (20/20)	Non-Toxin	0.01	Consensus (comb.lib./smm/nn)
658	Polymerase	WRYESMAIFAERLDE	HLA-DRB3*01:01	100.00% (20/20)	Non-Toxin	0.01	Consensus (comb.lib./smm/nn)
659	Polymerase	YCINILKVIQQLLIS	HLA-DRB4*01:01	100.00% (20/20)	Non-Toxin	0.01	Consensus (comb.lib./smm/nn)
660	Polymerase	YNQFLILNKLLSNRR	HLA-DRB1*11:01	100.00% (20/20)	Non-Toxin	0.01	Consensus (smm/nn/sturniolo)
661	Polymerase	YQIDQPFFVPTKITS	HLA-DRB3*01:01	100.00% (20/20)	Non-Toxin	0.01	Consensus (comb.lib./smm/nn)
662	Polymerase	YYQIDQPFFVPTKIT	HLA-DRB3*01:01	100.00% (20/20)	Non-Toxin	0.01	Consensus (comb.lib./smm/nn)
663	V Protein	AEGSDDIQLDPVVTD	HLA-DRB1*03:01	94.74% (54/57)	Non-Toxin	0.13	Consensus (smm/nn/sturniolo)
664	V Protein	AKMLSYAPEIAVSKE	HLA-DRB1*09:01	19.30% (11/57)	Non-Toxin	0.55	Consensus (comb.lib./smm/nn)
665	V Protein	AQPPYHWSIERSISP	HLA-DRB1*09:01	19.30% (11/57)	Non-Toxin	0.85	Consensus (comb.lib./smm/nn)
666	V Protein	CLVSDAKMLSYAPEI	HLA-DRB1*03:01	19.30% (11/57)	Non-Toxin	0.02	Consensus (smm/nn/sturniolo)
667	V Protein	DAKMLSYAPEIAVSK	HLA-DRB1*09:01	19.30% (11/57)	Non-Toxin	0.54	Consensus (comb.lib./smm/nn)
668	V Protein	DDIQLDPVVTDVVYH	HLA-DRB1*03:01	92.98% (53/57)	Non-Toxin	0.13	Consensus (smm/nn/sturniolo)
669	V Protein	DGKRAWVEEWCNPAC	HLA-DQA1*01:01/DQB1*05:01	87.72% (50/57)	Non-Toxin	0.82	Consensus (comb.lib./smm/nn)
670	V Protein	DIQLDPVVTDVVYHD	HLA-DRB3*01:01	92.98% (53/57)	Non-Toxin	0.02	Consensus (comb.lib./smm/nn)
671	V Protein	DIQLDPVVTDVVYHD	HLA-DRB1*03:01	92.98% (53/57)	Non-Toxin	0.27	Consensus (smm/nn/sturniolo)
672	V Protein	DKLELVNDGLNIIDF	HLA-DRB1*03:01	100.00% (57/57)	Non-Toxin	0.57	Consensus (smm/nn/sturniolo)
673	V Protein	DKLELVNDGLNIIDF	HLA-DRB3*01:01	100.00% (57/57)	Non-Toxin	1.02	Consensus (comb.lib./smm/nn)
674	V Protein	DPVVTDVVYHDHGGE	HLA-DRB3*01:01	98.25% (56/57)	Non-Toxin	0.02	Consensus (comb.lib./smm/nn)
675	V Protein	DPVVTDVVYHDHGGE	HLA-DRB1*03:01	98.25% (56/57)	Non-Toxin	0.6	Consensus (smm/nn/sturniolo)
676	V Protein	DSIKLYTSDDEEADQ	HLA-DRB3*01:01	100.00% (57/57)	Non-Toxin	0.28	Consensus (comb.lib./smm/nn)
677	V Protein	EFEDEFAGSSSEVIV	HLA-DRB1*07:01	98.25% (56/57)	Non-Toxin	0.56	Consensus (comb.lib./smm/nn)
678	V Protein	EGSDDIQLDPVVTDV	HLA-DRB1*03:01	94.74% (54/57)	Non-Toxin	0.13	Consensus (smm/nn/sturniolo)
679	V Protein	GNVCLVSDAKMLSYA	HLA-DRB1*03:01	19.30% (11/57)	Non-Toxin	0.01	Consensus (smm/nn/sturniolo)
680	V Protein	GNVCLVSDAKMLSYA	HLA-DRB1*11:01	19.30% (11/57)	Non-Toxin	0.7	Consensus (smm/nn/sturniolo)
681	V Protein	GSDDIQLDPVVTDVV	HLA-DRB1*03:01	94.74% (54/57)	Non-Toxin	0.13	Consensus (smm/nn/sturniolo)
682	V Protein	GSSSEVIVGISPEDE	HLA-DQA1*04:01/DQB1*04:02	19.30% (11/57)	Non-Toxin	0.83	Consensus (comb.lib./smm/nn)
683	V Protein	IKLYTSDDEEADQLE	HLA-DRB3*01:01	100.00% (57/57)	Non-Toxin	0.27	Consensus (comb.lib./smm/nn)
684	V Protein	IQLDPVVTDVVYHDH	HLA-DRB3*01:01	92.98% (53/57)	Non-Toxin	0.02	Consensus (comb.lib./smm/nn)
685	V Protein	IQLDPVVTDVVYHDH	HLA-DRB1*03:01	92.98% (53/57)	Non-Toxin	0.26	Consensus (smm/nn/sturniolo)
686	V Protein	KLELVNDGLNIIDFI	HLA-DRB1*03:01	100.00% (57/57)	Non-Toxin	0.53	Consensus (smm/nn/sturniolo)
687	V Protein	KLELVNDGLNIIDFI	HLA-DRB3*01:01	100.00% (57/57)	Non-Toxin	1	Consensus (comb.lib./smm/nn)
688	V Protein	KLYTSDDEEADQLEF	HLA-DRB3*01:01	100.00% (57/57)	Non-Toxin	0.28	Consensus (comb.lib./smm/nn)
689	V Protein	KMLSYAPEIAVSKED	HLA-DRB1*09:01	19.30% (11/57)	Non-Toxin	0.67	Consensus (comb.lib./smm/nn)
690	V Protein	LDPVVTDVVYHDHGG	HLA-DRB3*01:01	98.25% (56/57)	Non-Toxin	0.02	Consensus (comb.lib./smm/nn)
691	V Protein	LDPVVTDVVYHDHGG	HLA-DRB1*03:01	98.25% (56/57)	Non-Toxin	0.56	Consensus (smm/nn/sturniolo)
692	V Protein	LDSIKLYTSDDEEAD	HLA-DRB3*01:01	100.00% (57/57)	Non-Toxin	0.27	Consensus (comb.lib./smm/nn)
693	V Protein	LEFEDEFAGSSSEVI	HLA-DRB1*07:01	100.00% (57/57)	Non-Toxin	0.45	Consensus (comb.lib./smm/nn)
694	V Protein	LELVNDGLNIIDFIQ	HLA-DRB1*03:01	100.00% (57/57)	Non-Toxin	0.55	Consensus (smm/nn/sturniolo)
695	V Protein	LVSDAKMLSYAPEIA	HLA-DRB1*03:01	19.30% (11/57)	Non-Toxin	0.02	Consensus (smm/nn/sturniolo)
696	V Protein	LYTSDDEEADQLEFE	HLA-DQA1*03:01/DQB1*03:02	100.00% (57/57)	Non-Toxin	0.79	Consensus (comb.lib./smm/nn)
697	V Protein	MDKLELVNDGLNIID	HLA-DRB1*03:01	100.00% (57/57)	Non-Toxin	0.59	Consensus (smm/nn/sturniolo)
698	V Protein	NGNVCLVSDAKMLSY	HLA-DRB1*03:01	19.30% (11/57)	Non-Toxin	0.01	Consensus (smm/nn/sturniolo)
699	V Protein	NGNVCLVSDAKMLSY	HLA-DRB1*11:01	19.30% (11/57)	Non-Toxin	0.7	Consensus (smm/nn/sturniolo)
700	V Protein	NNGNVCLVSDAKMLS	HLA-DRB1*03:01	19.30% (11/57)	Non-Toxin	0.01	Consensus (smm/nn/sturniolo)

#	Protein	Peptide	Allele	Conservancy	Toxicity	Percentile rank	Method used
701	V Protein	NNGNVCLVSDAKMLS	HLA-DRB1*11:01	19.30% (11/57)	Non-Toxin	0.7	Consensus (smm/nn/sturniolo)
702	V Protein	NVCLVSDAKMLSYAP	HLA-DRB1*03:01	19.30% (11/57)	Non-Toxin	0.01	Consensus (smm/nn/sturniolo)
703	V Protein	NVCLVSDAKMLSYAP	HLA-DRB1*11:01	19.30% (11/57)	Non-Toxin	0.7	Consensus (smm/nn/sturniolo)
704	V Protein	NVNLDSIKLYTSDDE	HLA-DRB1*03:01	100.00% (57/57)	Non-Toxin	0.76	Consensus (smm/nn/sturniolo)
705	V Protein	PPYHWSIERSISPDK	HLA-DRB1*09:01	19.30% (11/57)	Non-Toxin	0.99	Consensus (comb.lib./smm/nn)
706	V Protein	PQTSRNVNLDSIKLY	HLA-DRB1*03:01	19.30% (11/57)	Non-Toxin	0.76	Consensus (smm/nn/sturniolo)
707	V Protein	PVVTDVVYHDHGGEC	HLA-DRB3*01:01	98.25% (56/57)	Non-Toxin	0.2	Consensus (comb.lib./smm/nn)
708	V Protein	QLDPVVTDVVYHDHG	HLA-DRB3*01:01	98.25% (56/57)	Non-Toxin	0.02	Consensus (comb.lib./smm/nn)
709	V Protein	QLDPVVTDVVYHDHG	HLA-DRB1*03:01	98.25% (56/57)	Non-Toxin	0.52	Consensus (smm/nn/sturniolo)
710	V Protein	QPPYHWSIERSISPD	HLA-DRB1*09:01	19.30% (11/57)	Non-Toxin	0.56	Consensus (comb.lib./smm/nn)
711	V Protein	QTSRNVNLDSIKLYT	HLA-DRB1*03:01	19.30% (11/57)	Non-Toxin	0.76	Consensus (smm/nn/sturniolo)
712	V Protein	RNVNLDSIKLYTSDD	HLA-DRB1*03:01	78.95% (45/57)	Non-Toxin	0.76	Consensus (smm/nn/sturniolo)
713	V Protein	SDAKMLSYAPEIAVS	HLA-DRB1*09:01	19.30% (11/57)	Non-Toxin	0.55	Consensus (comb.lib./smm/nn)
714	V Protein	SDDIQLDPVVTDVVY	HLA-DRB1*03:01	92.98% (53/57)	Non-Toxin	0.13	Consensus (smm/nn/sturniolo)
715	V Protein	SEVIVGISPEDEEPS	HLA-DQA1*04:01/DQB1*04:02	19.30% (11/57)	Non-Toxin	0.65	Consensus (comb.lib./smm/nn)
716	V Protein	SIKLYTSDDEEADQL	HLA-DRB3*01:01	100.00% (57/57)	Non-Toxin	0.27	Consensus (comb.lib./smm/nn)
717	V Protein	SRNVNLDSIKLYTSD	HLA-DRB1*03:01	19.30% (11/57)	Non-Toxin	0.76	Consensus (smm/nn/sturniolo)
718	V Protein	SSEVIVGISPEDEEP	HLA-DQA1*04:01/DQB1*04:02	19.30% (11/57)	Non-Toxin	0.39	Consensus (comb.lib./smm/nn)
719	V Protein	SSEVIVGISPEDEEP	HLA-DQA1*03:01/DQB1*03:02	19.30% (11/57)	Non-Toxin	0.98	Consensus (comb.lib./smm/nn)
720	V Protein	SSSEVIVGISPEDEE	HLA-DQA1*04:01/DQB1*04:02	19.30% (11/57)	Non-Toxin	0.29	Consensus (comb.lib./smm/nn)
721	V Protein	SSSEVIVGISPEDEE	HLA-DQA1*03:01/DQB1*03:02	19.30% (11/57)	Non-Toxin	0.82	Consensus (comb.lib./smm/nn)
722	V Protein	TSRNVNLDSIKLYTS	HLA-DRB1*03:01	19.30% (11/57)	Non-Toxin	0.76	Consensus (smm/nn/sturniolo)
723	V Protein	VCLVSDAKMLSYAPE	HLA-DRB1*03:01	19.30% (11/57)	Non-Toxin	0.01	Consensus (smm/nn/sturniolo)
724	V Protein	VCLVSDAKMLSYAPE	HLA-DRB1*11:01	19.30% (11/57)	Non-Toxin	0.7	Consensus (smm/nn/sturniolo)
725	V Protein	VNLDSIKLYTSDDEE	HLA-DRB1*03:01	100.00% (57/57)	Non-Toxin	0.76	Consensus (smm/nn/sturniolo)
726	V Protein	VSDAKMLSYAPEIAV	HLA-DRB1*09:01	19.30% (11/57)	Non-Toxin	0.55	Consensus (comb.lib./smm/nn)
727	V Protein	VVTDVVYHDHGGECT	HLA-DRB3*01:01	98.25% (56/57)	Non-Toxin	0.21	Consensus (comb.lib./smm/nn)
728	V Protein	WDGKRAWVEEWCNPA	HLA-DQA1*01:01/DQB1*05:01	87.72% (50/57)	Non-Toxin	0.86	Consensus (comb.lib./smm/nn)
729	V Protein	YTSDDEEADQLEFED	HLA-DQA1*03:01/DQB1*03:02	100.00% (57/57)	Non-Toxin	0.72	Consensus (comb.lib./smm/nn)
730	W Protein	AEGSDDIQLDPVVTD	HLA-DRB1*03:01	100.00% (39/39)	Non-Toxin	0.13	Consensus (smm/nn/sturniolo)
731	W Protein	AKMLSYAPEIAVSKE	HLA-DRB1*09:01	20.51% (8/39)	Non-Toxin	0.55	Consensus (comb.lib./smm/nn)
732	W Protein	CLVSDAKMLSYAPEI	HLA-DRB1*03:01	20.51% (8/39)	Non-Toxin	0.02	Consensus (smm/nn/sturniolo)
733	W Protein	DAKMLSYAPEIAVSK	HLA-DRB1*09:01	20.51% (8/39)	Non-Toxin	0.54	Consensus (comb.lib./smm/nn)
734	W Protein	DDIQLDPVVTDVVYH	HLA-DRB1*03:01	97.44% (38/39)	Non-Toxin	0.13	Consensus (smm/nn/sturniolo)
735	W Protein	DIQLDPVVTDVVYHD	HLA-DRB3*01:01	97.44% (38/39)	Non-Toxin	0.02	Consensus (comb.lib./smm/nn)
736	W Protein	DIQLDPVVTDVVYHD	HLA-DRB1*03:01	97.44% (38/39)	Non-Toxin	0.27	Consensus (smm/nn/sturniolo)
737	W Protein	DKLELVNDGLNIIDF	HLA-DRB1*03:01	100.00% (39/39)	Non-Toxin	0.57	Consensus (smm/nn/sturniolo)
738	W Protein	DPVVTDVVYHDHGGE	HLA-DRB3*01:01	97.44% (38/39)	Non-Toxin	0.02	Consensus (comb.lib./smm/nn)
739	W Protein	DPVVTDVVYHDHGGE	HLA-DRB1*03:01	97.44% (38/39)	Non-Toxin	0.6	Consensus (smm/nn/sturniolo)
740	W Protein	DSIKLYTSDDEEADQ	HLA-DRB3*01:01	100.00% (39/39)	Non-Toxin	0.28	Consensus (comb.lib./smm/nn)
741	W Protein	EFEDEFAGSSSEVIV	HLA-DRB1*07:01	97.44% (38/39)	Non-Toxin	0.56	Consensus (comb.lib./smm/nn)
742	W Protein	EGSDDIQLDPVVTDV	HLA-DRB1*03:01	100.00% (39/39)	Non-Toxin	0.13	Consensus (smm/nn/sturniolo)
743	W Protein	GIPIKKAQTRNIHLL	HLA-DRB1*07:01	5.13% (2/39)	Non-Toxin	0.53	Consensus (comb.lib./smm/nn)
744	W Protein	GNVCLVSDAKMLSYA	HLA-DRB1*03:01	20.51% (8/39)	Non-Toxin	0.01	Consensus (smm/nn/sturniolo)
745	W Protein	GSDDIQLDPVVTDVV	HLA-DRB1*03:01	100.00% (39/39)	Non-Toxin	0.13	Consensus (smm/nn/sturniolo)
746	W Protein	IKLYTSDDEEADQLE	HLA-DRB3*01:01	100.00% (39/39)	Non-Toxin	0.27	Consensus (comb.lib./smm/nn)
747	W Protein	IQLDPVVTDVVYHDH	HLA-DRB3*01:01	97.44% (38/39)	Non-Toxin	0.02	Consensus (comb.lib./smm/nn)

#	Protein	Peptide	Allele	Conservancy	Toxicity	Percentile rank	Method used
748	W Protein	IQLDPVVTDVVYHDH	HLA-DRB1*03:01	97.44% (38/39)	Non-Toxin	0.26	Consensus (smm/nn/sturniolo)
749	W Protein	KLELVNDGLNIIDFI	HLA-DRB1*03:01	100.00% (39/39)	Non-Toxin	0.53	Consensus (smm/nn/sturniolo)
750	W Protein	KLYTSDDEEADQLEF	HLA-DRB3*01:01	100.00% (39/39)	Non-Toxin	0.28	Consensus (comb.lib./smm/nn)
751	W Protein	LDPVVTDVVYHDHGG	HLA-DRB3*01:01	97.44% (38/39)	Non-Toxin	0.02	Consensus (comb.lib./smm/nn)
752	W Protein	LDPVVTDVVYHDHGG	HLA-DRB1*03:01	97.44% (38/39)	Non-Toxin	0.56	Consensus (smm/nn/sturniolo)
753	W Protein	LDSIKLYTSDDEEAD	HLA-DRB3*01:01	100.00% (39/39)	Non-Toxin	0.27	Consensus (comb.lib./smm/nn)
754	W Protein	LEFEDEFAGSSSEVI	HLA-DRB1*07:01	100.00% (39/39)	Non-Toxin	0.45	Consensus (comb.lib./smm/nn)
755	W Protein	LELVNDGLNIIDFIQ	HLA-DRB1*03:01	100.00% (39/39)	Non-Toxin	0.55	Consensus (smm/nn/sturniolo)
756	W Protein	LVSDAKMLSYAPEIA	HLA-DRB1*03:01	20.51% (8/39)	Non-Toxin	0.02	Consensus (smm/nn/sturniolo)
757	W Protein	MDKLELVNDGLNIID	HLA-DRB1*03:01	100.00% (39/39)	Non-Toxin	0.59	Consensus (smm/nn/sturniolo)
758	W Protein	NGNVCLVSDAKMLSY	HLA-DRB1*03:01	20.51% (8/39)	Non-Toxin	0.01	Consensus (smm/nn/sturniolo)
759	W Protein	NNGNVCLVSDAKMLS	HLA-DRB1*03:01	20.51% (8/39)	Non-Toxin	0.01	Consensus (smm/nn/sturniolo)
760	W Protein	NVCLVSDAKMLSYAP	HLA-DRB1*03:01	20.51% (8/39)	Non-Toxin	0.01	Consensus (smm/nn/sturniolo)
761	W Protein	PVVTDVVYHDHGGEC	HLA-DRB3*01:01	97.44% (38/39)	Non-Toxin	0.2	Consensus (comb.lib./smm/nn)
762	W Protein	QLDPVVTDVVYHDHG	HLA-DRB3*01:01	97.44% (38/39)	Non-Toxin	0.02	Consensus (comb.lib./smm/nn)
763	W Protein	QLDPVVTDVVYHDHG	HLA-DRB1*03:01	97.44% (38/39)	Non-Toxin	0.52	Consensus (smm/nn/sturniolo)
764	W Protein	QPPYHWSIERSISPD	HLA-DRB1*09:01	20.51% (8/39)	Non-Toxin	0.56	Consensus (comb.lib./smm/nn)
765	W Protein	SDAKMLSYAPEIAVS	HLA-DRB1*09:01	20.51% (8/39)	Non-Toxin	0.55	Consensus (comb.lib./smm/nn)
766	W Protein	SDDIQLDPVVTDVVY	HLA-DRB1*03:01	97.44% (38/39)	Non-Toxin	0.13	Consensus (smm/nn/sturniolo)
767	W Protein	SIKLYTSDDEEADQL	HLA-DRB3*01:01	100.00% (39/39)	Non-Toxin	0.27	Consensus (comb.lib./smm/nn)
768	W Protein	SSEVIVGISPEDEEP	HLA-DQA1*04:01/DQB1*04:02	20.51% (8/39)	Non-Toxin	0.39	Consensus (comb.lib./smm/nn)
769	W Protein	SSSEVIVGISPEDEE	HLA-DQA1*04:01/DQB1*04:02	20.51% (8/39)	Non-Toxin	0.29	Consensus (comb.lib./smm/nn)
770	W Protein	TRNIHLLGRKTCLGR	HLA-DRB1*11:01	92.31% (36/39)	Non-Toxin	0.44	Consensus (smm/nn/sturniolo)
771	W Protein	VCLVSDAKMLSYAPE	HLA-DRB1*03:01	20.51% (8/39)	Non-Toxin	0.01	Consensus (smm/nn/sturniolo)
772	W Protein	VSDAKMLSYAPEIAV	HLA-DRB1*09:01	20.51% (8/39)	Non-Toxin	0.55	Consensus (comb.lib./smm/nn)
773	W Protein	VVTDVVYHDHGGECT	HLA-DRB3*01:01	97.44% (38/39)	Non-Toxin	0.21	Consensus (comb.lib./smm/nn)

Table S4. Homology models of full length protein tertiary structure of NIPAH virus. Nipah protein sequences were retrieved from NCBI. The homology modeling tools SwissModel and I-Tasser were used.

#	NIPAH-CoV Protein	Templet used	Protein length	Tools used	QMEAN	C-Score	Estimated TM-score	Estimated RMSD	Iden1
1	C Protein	3dyjA	Full length	I-TASSER	-	-2.88	0.39±0.13	11.5±4.5Å	0.14
2	Fusion Protein	5evmA	Full length	I-TASSER	-	-0.53	0.65±0.13	8.7±4.6Å	1
3	Glycoprotein	3d11A	Full length	I-TASSER	-	-0.83	0.61±0.14	9.7±4.6Å	0.95
4	Matrix Protein	4g1gA	Full length	I-TASSER	-	0.18	0.74±0.11	6.1±3.8Å	0.2
5	Nucleocapsid	6jc3A	Full length	I-TASSER	-	-0.88	0.60±0.14	9.5±4.6Å	0.31
6	Phosphoprotein	4n5bA	Full length	I-TASSER	-	-1.29	0.55±0.15	11.2±4.6Å	1
7	Polymerase	6v85.1.A	Full length	SwissModel	-6.06	-	-	-	-
8	V protein	2b5lA	Full length	I-TASSER	-	-1.09	0.58±0.14	9.6±4.6Å	0.26
9	W protein	4co6A	Full length	I-TASSER	-	-0.75	0.62±0.14	8.8±4.6Å	1

Table S5: HLA alleles covered by the overlapping CTL and HTL epitopes.

#	HLA alleles	Class of HLA alleles
1	HLA-A*01:01	HLA Class I alleles
2	HLA-A*02:01	
3	HLA-A*02:03	
4	HLA-A*02:06	
5	HLA-A*03:01	
6	HLA-A*11:01	
7	HLA-A*23:01	
8	HLA-A*24:02	
9	HLA-A*26:01	
10	HLA-A*30:01	
11	HLA-A*30:02	
12	HLA-A*31:01	
13	HLA-A*32:01	
14	HLA-A*33:01	
15	HLA-A*68:01	
16	HLA-A*68:02	
17	HLA-B*07:02	
18	HLA-B*08:01	
19	HLA-B*15:01	
20	HLA-B*35:01	
21	HLA-B*40:01	
22	HLA-B*44:02	
23	HLA-B*44:03	
24	HLA-B*51:01	
25	HLA-B*53:01	
26	HLA-B*57:01	
27	HLA-B*58:01	

#	HLA alleles	Class of HLA alleles
28	HLA-DPA1*01/DPB1*04:01	HLA Class II alleles
29	HLA-DPA1*01:03/DPB1*02:01	
30	HLA-DPA1*02:01/DPB1*01:01	
31	HLA-DPA1*02:01/DPB1*14:01	
32	HLA-DPA1*03:01/DPB1*04:02	
33	HLA-DQA1*01:01/DQB1*05:01	
34	HLA-DQA1*01:02/DQB1*06:02	
35	HLA-DQA1*03:01/DQB1*03:02	
36	HLA-DQA1*04:01/DQB1*04:02	
37	HLA-DQA1*05:01/DQB1*02:01	
38	HLA-DQA1*05:01/DQB1*03:01	
39	HLA-DRB1*01:01	
40	HLA-DRB1*03:01	
41	HLA-DRB1*04:01	
42	HLA-DRB1*04:05	
43	HLA-DRB1*07:01	
44	HLA-DRB1*08:02	
45	HLA-DRB1*09:01	
46	HLA-DRB1*11:01	
47	HLA-DRB1*13:02	
48	HLA-DRB1*15:01	
49	HLA-DRB3*01:01	
50	HLA-DRB3*02:02	
51	HLA-DRB4*01:01	
52	HLA-DRB5*01:01	

Table S6: Population coverage by all the overlapping CTL and HTL epitopes forming epitope clusters.

Population/area	Class combined		
	coverage[a]	average_hit[b]	pc90[c]
Algeria	75.83%	49.09	2.07
American Samoa	98.75%	123.14	58.71
Argentina	99.04%	148.28	63.93
Australia	95.84%	131.68	58.36
Austria	99.99%	249.29	148.97
Belarus	43.81%	19.06	1.07
Belgium	99.87%	197.29	103.64
Bolivia	77.82%	27.34	7.66
Borneo	42.09%	16.89	3.11
Brazil	98.55%	164.55	73.95
Bulgaria	99.75%	193.33	104.4
Burkina Faso	67.18%	58.94	14.32
Cameroon	93.95%	149.53	54.78
Canada	27.22%	12.32	0.69
Cape Verde	99.83%	232.79	125.86
Central Africa	94.14%	140.31	34.19
Central African Republic	77.51%	57.41	2.22
Central America	53.29%	24.9	1.07
Chile	98.57%	158.04	67.81
China	97.61%	146.36	59.33
Colombia	56.35%	28.97	1.15
Congo	63.54%	31.88	1.37
Cook Islands	62.91%	30	1.35
Costa Rica	24.31%	7.86	2.38
Croatia	99.93%	224.31	126
Cuba	99.84%	207.24	108.55
Czech Republic	99.84%	218.88	117.13
Denmark	87.68%	38.39	4.06
East Africa	96.51%	156.14	63.27
East Asia	99.61%	186.19	81.45
Ecuador	89.16%	67.36	19.38
England	100.00%	259.61	159.85
Equatorial Guinea	43.90%	24.85	0.89
Ethiopia	82.77%	40.17	3.48
Europe	99.95%	234.55	135.3
Fiji	75.74%	45.68	2.06
Finland	100.00%	245.18	142.03
France	99.97%	239.02	139.04
Gabon	41.78%	20.76	0.86
Georgia	99.57%	206.5	104.32
Germany	99.99%	258.01	156.1
Greece	65.84%	36.83	1.46
Guatemala	52.58%	25.98	1.05
Guinea-Bissau	98.93%	188.56	82.62
Hong Kong	96.05%	120.67	58.65

Population/area	Class combined		
	coverage[a]	average_hit[b]	pc90[c]
India	97.17%	146.09	58.74
Indonesia	92.82%	111.45	25.26
Iran	99.10%	186.13	78.38
Ireland Northern	100.00%	264.94	165.96
Ireland South	99.99%	261.39	160.81
Israel	96.51%	145.07	58.8
Italy	99.85%	257.55	141.32
Ivory Coast	67.75%	55.21	13.33
Jamaica	27.41%	10.06	0.83
Japan	99.61%	180.01	79.85
Jordan	95.62%	117.51	50.03
Kenya	89.56%	111.28	33.52
Korea; South	99.68%	193.37	89.99
Lebanon	69.40%	40.22	1.63
Macedonia	74.69%	53.35	1.98
Malaysia	91.87%	105.12	19.88
Mali	96.02%	147.68	63.78
Martinique	77.11%	54.36	2.18
Mexico	99.08%	171.17	75.24
Mongolia	98.98%	141.56	64.56
Morocco	99.77%	212.65	108.95
Nauru	37.40%	14.64	0.96
Netherlands	82.53%	48.92	2.86
New Caledonia	99.66%	145.08	64.83
New Zealand	63.88%	32.43	1.38
Niue	40.56%	17.37	3.53
North Africa	98.98%	182.84	80.96
North America	99.87%	218.51	116.1
Northeast Asia	97.67%	146.82	59.36
Norway	93.75%	54.36	5.81
Oceania	97.51%	128.14	58.58
Oman	99.69%	171.64	102.28
Pakistan	97.13%	105.39	59.06
Papua New Guinea	99.30%	117.19	58.78
Peru	100.00%	161.92	75.69
Philippines	96.36%	110.68	58.28
Poland	99.96%	231.67	135.44
Portugal	99.70%	204.4	102.59
Romania	99.67%	178	98.67
Russia	99.79%	188.78	96.93
Rwanda	70.09%	49.73	1.67
Samoa	71.25%	28.82	1.74
Sao Tome and Principe	98.89%	194.11	82.59
Saudi Arabia	99.65%	186.61	95.11
Scotland	96.52%	95.32	23.08
Senegal	96.92%	163.73	65.82
Serbia	73.37%	61.35	13.15
Singapore	97.24%	140.39	58.99

Population/area	Class combined		
	coverage[a]	average_hit[b]	pc90[c]
Slovenia	83.68%	47.94	3.06
South Africa	95.27%	151.25	58.34
South America	95.02%	123.47	43.49
South Asia	98.61%	162.24	66.72
Southeast Asia	97.51%	140.12	58.91
Southwest Asia	95.73%	138.47	58.57
Spain	97.47%	141.95	48.85
Sri Lanka	52.39%	43.15	12.39
Sudan	97.47%	166.24	63.94
Sweden	100.00%	260.91	157.95
Taiwan	99.22%	163.27	75.28
Thailand	96.75%	137.1	59.11
Tokelau	20.79%	4.08	2.27
Tonga	62.79%	28.86	4.84
Tunisia	99.33%	181.42	82.85
Turkey	85.87%	78.2	3.54
Uganda	94.87%	135.67	58.56
Ukraine	50.64%	23.43	1.22
United Arab Emirates	35.19%	41.36	7.56
United States	99.88%	219.7	117.86
Venezuela	90.04%	87.35	47.01
Vietnam	96.12%	133.53	58.42
West Africa	98.37%	177.28	75.18
West Indies	99.65%	201.68	105.88
Zambia	98.10%	159.01	73.61
Zimbabwe	97.65%	168.43	66.45
**World**	**99.71%**	**201.5**	**98.56**
**Average**	**85.79**	**127.56**	**56.91**
**Standard deviation**	**20.73**	**74.82**	**47.14**

Note: Following allele(s) were not available at IEDB "Population coverage" tool database, and therefore not included in the calculation, please note that allele names are case sensitive:DPA1*01, DRB5*01:01, DRB3*02:02, DRB3*01:01, DRB4*01:01.
a: projected population coverage; **b:** average number of epitope hits / HLA combinations recognized by the population; **c:** minimum number of epitope hits / HLA combinations recognized by 90% of the population

Table S7: Construct of CTL-MPV-1, CTL-MPV-2, CTL-MPV-3, HTL-MPV-1, and HTL-MPV-2. Physicochemical property analysis based on the amino acid sequences of all the designed three CTL and two HTL Multi-Patch Vaccines. The adjuvants human β Defensin 2 and β Defensin 3 are shown in green and magenta respectively; CTL GaEI Ag-Patches are shown in red; HTL GaEI Ag-Patches are shown in blue; linkers and His tag are shown in black.

S.No.	Vaccine	Vaccine constructs
1	**CTL-MPV-1 construct**<br><br>(Comprising of identified CTL Ag-PATCHES from c Protein, Fusion Protein, Glycoprotein, Matrix Protein, Nucleoprotein, Phosphoprotein of NiV):	GIGDPVTCLKSGAICHPVFCPRRYKQIGTCGLPGTKCCKKPEAAAKRMMEVLKEEAWRIYP<br>VLLPQMELLGGGGSITHQEQTMGMYVLYLMQRCCPMLPKLQFLKKIGKLGGGGSISECSV<br>GILHYEKLSKIGLVKGVTRKYKIKSNPLTKDIVIKMIPNVSNMGGGGSESDSITGQIIYVDLSS<br>YYIIVRVYFPILTEIQQAYIQELLPVSFNNDNSEWISIVPNFILVRNTLGGGGSASDKGKNPSK<br>VIKSYYGTMDIKKINEGLLDSKILSAFNTVGGGGSEVLDRGDEVPSLFMTNVWTPSNPNTV<br>YHCSAVYNNEFYYVGGGGSEENSKIVFIEISDQRLSIGSPSKIYDSLGQPVFYQASFSWDT<br>MIKFGDVQTVNPLVVNWRGGGGSSESMEGVSDFSPSSWEHGGYLDKVEPEIDENGSMIP<br>KYKIYTPGANERKYNNYMYLICYGFGGGGSIQLDKHQALRIFFLSITKLNDSGIYMIPRTMLE<br>FGGGGSAESMKVGAAFTLISMYSERPGALIRSLLNDPDIEAVGGGGSEEMEGLMRILKTAR<br>DSSKGKTPFVDSRAYGLGGGGSITIEAQIWILIAKAVTAPDTAEESETRRWAKYVQQKRVN<br>PFFALTQQWGGGGSNTIKSLMLLYREIGPRAPYMVLLEESIQTKFAPGGYPLLWSFAMGV<br>ATTIDRSMGALNINRGYLEPMYFRLGGGGSKYIMPSDDFSNTFFPHDTDRLNYGGGGSSV<br>LMGVINSIKLINLDMRLNHIEEQVKEIPKIINKLESIDRVLAKTNTALSTIEGHLVSMEAAAKGII<br>NTLQKYYCRVRGGRCAVLSCLPKEEQIGKCSTRGRKCCRRKKHHHHHH
2	**CTL-MPV-2 construct**<br><br>(Comprising of identified CTL Ag-PATCHES from Polymerase, V Protein,W Protein of NiV):	GIGDPVTCLKSGAICHPVFCPRRYKQIGTCGLPGTKCCKKPEAAAKKSDKTGKKCYYLTPE<br>MVLMYCDVLEGRMMMETTVKSDIKYQPLISRSNALWGLIDPLFPVMGNRIYNIVSMIEPLVL<br>ALGGGGSFTDQKIRSMFIDDLLSILNIDNIHLLAEFFSFFRTFGHPILEAKGGGGSLTIDDCVK<br>NWESFCGIQFDCFMELKLDSDLSMYMKDKALSPIKDEWDSVYPREVLSYGGGGSEALIAS<br>GVGKYFKENGMVKDEHELLKTLFGGGGSKTPEDDIFIHYPKGGIEGYSQKTWTIATIPFLFL<br>SAYETNTRIAAIGGGGSTQKVHPNLPYKVKKEICAKQAQLYFERLRMNLRALGGGGSDIKL<br>GNVKRPTKALRSAIRIATVYTWAYGDNEECWGGGGSFLIVDPELFALYLGQSISIKWAFEIH<br>HRRPRGRHTMVDLLSDLVSNTSKHTYKVLSNALSHPRVFKRGGGGSFEKLSQNLLVTSYM<br>IYLMNWCDFGGGGSHVDTSSRSWNTSDLDFVIFYASLTYLRRGIIKQLGGGGSRPSVENH<br>KYRRIGLNSSSCYKALNLSPLIQRYLPSGAQRLFIGEGSGSMMLLYQSTLGQSISFYGGGG<br>SVMMEDGLLVSKIAYTPGFPISRLFNMYRSYFGLVLVCFPVYSNPDSTEVYLGGGGSAEG<br>SDDIQLDPVVTDVVYGGGGSCLVSDAKVLSYAPEIGGGGSQQGKDAQPPYHWSIEAAAK<br>GIINTLQKYYCRVRGGRCAVLSCLPKEEQIGKCSTRGRKCCRRKKHHHHHH

S.No.	Vaccine	Vaccine constructs
**3**	**HTL-MPV-1 construct**<br><br>(Comprising of identified HTL Ag-PATCHES from C Protein, Fusion Protein, Glycoprotein of NiV):	GIGDPVTCLKSGAICHPVFCPRRYKQIGTCGLPGTKCCKKPEAAAKMMASILLTLFRRTKKKYRRHTDDQAFNNPASKTEQKHGGGGSCLRMMEVLKEEAWRIYPVLLPQMELLEKEGGGGSQKVQMTYNWTQWLQTLYTMIMEENVPDMDGGGGSMVVILDKRCYCNLLILILMISECSVGIGGGGSKIKSNPLTKDIVIKMIPNVSNMSQCTGGGGGSKNNTHDLVGDVRLAGVIMAGVAIGIATAAQITAGGGGGSKQTELSLDLALSKYLSDLLFVFGPNLQDPVSNSMTIQAISQAFGGNYETLLRTLGYATEDFDDLLEGGGGSSITGQIIYVDLSSYYIIVRVYFPILTEIQQAYIQELLPVSFNNDNSEWISIVPNFILVRNTLISNIEIGFCLITKRSVICNGGGGSDTVNPSLISMLSMIILYVLSIASLCIGLITFISFIIVEKKRNTYSRLEDRRVGGGGSDSKILSAFNTVIALLGSIVIIVMNIMIIQNYTRSTDNQAMIKDALQSIQQQIKGLADGGGGSAMDEGYFAYSHLEKIGSCSRGVSGGGGSPEVCWEGVYNDAFLIDRINWISAGVFLEAAAKGIINTLQKYYCRVRGGRCAVLSCLPKEEQIGKCSTRGRKCCRRKKHHHHHH
**4**	**HTL-MPV-2 construct**<br><br>(Comprising of identified HTL Ag-PATCHES from Matrix protein, Nucleoprotein, protein, Phosphoprotein, Polymerase, V Protein of NiV):	GIGDPVTCLKSGAICHPVFCPRRYKQIGTCGLPGTKCCKKPEAAAKQLDKHQALRIFFLSITKLNDSGIYMIPRTMLEFRRNNAIAFNLLVYGGGGSPSIPREFMIYDDVFIDNTGRILKGGGGGSTLTTKIRIFVPATNSPELRWELTLFALDVIRSPSAAEGGGGSERPGALIRSLLNDPDIEAVIIDVGSMVNGIPVMGGGGSSKGKTPFVDSRAYGLRITDMSTLVSAVITIEAQIWILGGGGSRHHAGGIDQNMANRLGLSSNQVAELAAAVQETSAGRQGGGGSAEGSDDIQLDPVVTDVVYHDHGGECTGGGGSLEQQSLFSFDNVKNFRDGSLTNEPYGAAVQLREDLIGGGGSDQKIRSMFIDDLLSILNIDNIHLLAEFFSFFRTFGGGGGSGYCINILKVIQQLLISTEFSINETLTLDVTSPISNNLGGGGSHHDYNQFLILNKLLSNRRQNDGGGGSDDLKKYYQIDQPFFVPTKITSGGGGSAEGSDDIQLDPVVTDVVYHDHGGECTGGGGSNNGNVCLVSDAKMLSYAPEIAVSKEDGGGGSPQTSRNVNLDSIKLYTSDDEEADQLEFEDEFAGSSSEVIVGISPEDEEPSEAAAKGIINTLQKYYCRVRGGRCAVLSCLPKEEQIGKCSTRGRKCCRRKKHHHHHH

Table S8: INF-γ inducing POSITIVE epitopes with a score of 1 or more than 1, screened from the CTL MPVs.

#	CTL-MPV	Sequence	Method	Result	Score
1	CTL-MPV-1	QQQSYLDSGIHFGAT	SVM	POSITIVE	0.014601
2	CTL-MPV-1	HWQQQSYLDSGIHFG	SVM	POSITIVE	0.021451
3	CTL-MPV-1	HFGATTTAPSLSGKG	SVM	POSITIVE	0.006054
4	CTL-MPV-1	EVDSAVVGIFMPAFT	SVM	POSITIVE	0.363695
5	CTL-MPV-1	FARAFVEVYLFFYFW	SVM	POSITIVE	0.349729
6	CTL-MPV-1	GSSVLFVTNPFYQSV	SVM	POSITIVE	0.3411
7	CTL-MPV-1	GYTGYIDWFQPNISG	SVM	POSITIVE	0.085113
8	CTL-MPV-1	HEEHVLQTVIPAPWY	SVM	POSITIVE	0.559065
9	CTL-MPV-1	KISSLILCNFQFQCC	SVM	POSITIVE	0.408967
10	CTL-MPV-1	RWNRSLFERVLSVAL	SVM	POSITIVE	0.444948
11	CTL-MPV-1	CGSYDYRDYYENESF	SVM	POSITIVE	0.243738
12	CTL-MPV-1	TVYSYIRTPVDSYLK	SVM	POSITIVE	0.314022
13	CTL-MPV-1	VFKDELFTLAVNRMS	SVM	POSITIVE	0.012104
14	CTL-MPV-1	VYSYIRTPVDSYLKF	SVM	POSITIVE	0.061491
15	CTL-MPV-1	DSGIHFGATTTAPSL	SVM	NEGATIVE	-0.0848
16	CTL-MPV-1	GIHFGATTTAPSLSG	SVM	NEGATIVE	-0.06577
17	CTL-MPV-1	IHFGATTTAPSLSGK	SVM	NEGATIVE	-0.04903
18	CTL-MPV-1	LDSGIHFGATTTAPS	SVM	NEGATIVE	-0.13812
19	CTL-MPV-1	SGIHFGATTTAPSLS	SVM	NEGATIVE	-0.18675
20	CTL-MPV-1	DSGIHFGATTTAPSL	SVM	NEGATIVE	-0.0848
21	CTL-MPV-1	LDSGIHFGATTTAPS	SVM	NEGATIVE	-0.13812
22	CTL-MPV-1	WQQQSYLDSGIHFGA	SVM	NEGATIVE	-0.03573
23	CTL-MPV-1	SGIHFGATTTAPSLS	SVM	NEGATIVE	-0.18675
24	CTL-MPV-1	FGATTTAPSLSGKGN	SVM	NEGATIVE	-0.22921
25	CTL-MPV-1	GIHFGATTTAPSLSG	SVM	NEGATIVE	-0.06577
26	CTL-MPV-1	QQSYLDSGIHFGATT	SVM	NEGATIVE	-0.12639
27	CTL-MPV-1	SHWQQQSYLDSGIHF	SVM	NEGATIVE	-0.16041
28	CTL-MPV-1	IHFGATTTAPSLSGK	SVM	NEGATIVE	-0.04903
29	CTL-MPV-1	YLDSGIHFGATTTAP	SVM	NEGATIVE	-0.24225
30	CTL-MPV-1	SGIHFGATTTAPSLS	SVM	NEGATIVE	-0.18675
31	CTL-MPV-1	QSYLDSGIHFGATTT	SVM	NEGATIVE	-0.03631
32	CTL-MPV-1	SYLDSGIHFGATTTA	SVM	NEGATIVE	-0.22194
33	CTL-MPV-1	EDQKYSEFLGNMVNE	SVM	NEGATIVE	-0.88771

#	CTL-MPV	Sequence	Method	Result	Score
34	CTL-MPV-2	SYLGSGIHSGATTTA	SVM	POSITIVE	0.037787
35	CTL-MPV-2	QQQSYLGSGIHSGAT	SVM	POSITIVE	0.145343
36	CTL-MPV-2	FWSAGVTLYNITTGL	SVM	POSITIVE	0.465067
37	CTL-MPV-2	ANGLDTFSGFKVDFW	SVM	POSITIVE	0.260875
38	CTL-MPV-2	YLGSGIHSGATTTAP	SVM	NEGATIVE	-0.25657
39	CTL-MPV-2	LGSGIHSGATTTAPS	SVM	NEGATIVE	-0.03696
40	CTL-MPV-2	GSGIHSGATTTAPSL	SVM	NEGATIVE	-0.20969
41	CTL-MPV-2	QSYLGSGIHSGATTT	SVM	NEGATIVE	-0.00588
42	CTL-MPV-2	QQSYLGSGIHSGATT	SVM	NEGATIVE	-0.0339
43	CTL-MPV-2	WQQQSYLGSGIHSGA	SVM	NEGATIVE	-0.06562
44	CTL-MPV-2	NGLDTFSGFKVDFWS	SVM	NEGATIVE	-0.4901

Table S9: INF-γ inducing POSITIVE epitopes with a score of 1 or more than 1, screened from the HTL MPVs.

#	HTL-MPV	Sequence	Method	Result	Score
1	HTL-MPV-1	FGLARYIKNDSNYVVK	SVM	POSITIVE	0.152406
2	HTL-MPV-1	AAIAFNVYVNTKPEILDF	SVM	POSITIVE	0.038756
3	HTL-MPV-1	AIAFNVYVNTKPEILDFD	SVM	POSITIVE	0.024703
4	HTL-MPV-1	GLARYIKNDSNYVVKG	SVM	NEGATIVE	-0.04135
5	HTL-MPV-1	LARYIKNDSNYVVKGN	SVM	NEGATIVE	-0.05923
6	HTL-MPV-1	ARYIKNDSNYVVKGNA	SVM	NEGATIVE	-0.0928
7	HTL-MPV-1	DFGLARYIKNDSNYVV	SVM	NEGATIVE	-0.08624
8	HTL-MPV-1	AFNVYVNTKPEILDF	SVM	NEGATIVE	-0.78488
9	HTL-MPV-1	FNVYVNTKPEILDFD	SVM	NEGATIVE	-0.70084
10	HTL-MPV-1	ILDFDRLVNGMLQCV	SVM	NEGATIVE	-0.502
11	HTL-MPV-1	KPEILDFDRLVNGML	SVM	NEGATIVE	-0.44773
12	HTL-MPV-1	AFNVYVNTKPEILDFD	SVM	NEGATIVE	-0.40182
13	HTL-MPV-1	IAFNVYVNTKPEILDF	SVM	NEGATIVE	-0.5766
14	HTL-MPV-1	AIAFNVYVNTKPEILDF	SVM	NEGATIVE	-0.41184
15	HTL-MPV-1	IAFNVYVNTKPEILDFD	SVM	NEGATIVE	-0.17287
#	HTL-MPV	Sequence	Method	Result	Score
16	HTL-MPV-2	GIVLKDIKLGNLLLT	SVM	POSITIVE	0.006928
17	HTL-MPV-2	QGIVLKDIKLGNLLL	SVM	POSITIVE	0.076582
18	HTL-MPV-2	SQGIVLKDIKLGNLL	SVM	POSITIVE	0.10677
19	HTL-MPV-2	HSQGIVLKDIKLGNL	SVM	POSITIVE	0.042983
20	HTL-MPV-2	LHSQGIVLKDIKLGN	SVM	POSITIVE	0.011449
21	HTL-MPV-2	YLHSQGIVLKDIKLG	SVM	POSITIVE	2.047511
22	HTL-MPV-2	FKVYFWSAGVTLYNI	SVM	POSITIVE	0.175602
23	HTL-MPV-2	VYFWSAGVTLYNITT	SVM	POSITIVE	0.215957
24	HTL-MPV-2	KVYFWSAGVTLYNIT	SVM	POSITIVE	0.131178
25	HTL-MPV-2	FSGFKVYFWSAGVTL	SVM	POSITIVE	0.037046
26	HTL-MPV-2	SGFKVYFWSAGVTLY	SVM	POSITIVE	1.969099
27	HTL-MPV-2	IVLKDIKLGNLLLTT	SVM	NEGATIVE	-0.37097
28	HTL-MPV-2	EYLHSQGIVLKDIKL	SVM	NEGATIVE	-0.23876
29	HTL-MPV-2	VLKDIKLGNLLLTTG	SVM	NEGATIVE	-0.61579
30	HTL-MPV-2	ANGLDTFSGFKVYFW	SVM	NEGATIVE	-0.55794
31	HTL-MPV-2	NGLDTFSGFKVYFWS	SVM	NEGATIVE	-0.51605

Table S10: AllergenFP and Vaxijen analysis of MPVs. For the Vaxijen the default threshold is 0.4, and here all the MPVs have scored above 0.4, indicating potential antigenic nature.

S.No.	MPVs	AllergenFP v.1.0	Vaxijen	
1	CTL-MPV-1	NON-ALLERGEN	ANTIGENS	0.5571
2	CTL-MPV-2	NON-ALLERGEN	ANTIGENS	0.5360
3	HTL-MPV-1	NON-ALLERGEN	ANTIGENS	0.5455
4	HTL-MPV-2	NON-ALLERGEN	ANTIGENS	0.5201

Table S11: Parameters for the tertiary structure homology modeling of all the CTL and HTL MPVs by the I-TASSER tool.

S.No.	MPVs	PDB hit	C-Score	TM-Score	RMSD (Å)
1	CTL-MPV-1	7abhu	-0.42	0.66 ± 0.13	9.4 ± 4.6
2	CTL-MPV-2	5a22A	-2.01	0.47 ± 0.15	13.3 ± 4.1
3	HTL-MPV-1	2b9bB	-3.41	0.34 ± 0.11	16.6 ±2.9
4	HTL-MPV-2	7wooF	-2.00	0.48 ± 0.15	12.8 ± 4.2

Table S12: Refinement parameter values for CTL and HTL MPV models after refinement by GalaxyRefine tool. RMSD value in Å indicated deviation from initial model. GDT-HA (global distance test-High Accuracy): backbone structure accuracy measured by GDT-HA.

S.No.	MPV Models	GDT-HA	RMSD	MolProbity	Clash score	Poor rotamers	Rama favored
1	CTL-MPV-1	0.9461	0.429	2.235	17.6	0.7	91.9
2	CTL-MPV-2	0.9668	0.370	2.274	15.4	0.6	88.7
3	HTL-MPV-1	0.9555	0.398	2.709	33.2	1.1	84.5
4	HTL-MPV-2	0.9834	0.330	2.465	18.4	0.4	82.3

Table S13: B cell linear epitopes screened from CTL & HTL MPVs.

No.	CTL-MPV-1	Chain	Start	End	Peptide	Number of residues	Score
1	CTL-MPV-1	A	795	830	GRCAVLSCLPKEEQIGKCSTRGRKCCRRKKHHHHHH	36	0.909
2	CTL-MPV-1	A	1	41	GIGDPVTCLKSGAICHPVFCPRRYKQIGTCGLPGTKCCKKP	41	0.849
3	CTL-MPV-1	A	226	249	KNPSKVIKSYYGTMDIKKINEGLL	24	0.762
4	CTL-MPV-1	A	761	786	NTALSTIEGHLVSMEAAAKGIINTLQ	26	0.76
5	CTL-MPV-1	A	572	585	VTAPDTAEESETRR	14	0.752
6	CTL-MPV-1	A	158	170	PGANERKYNNYMY	13	0.749
7	CTL-MPV-1	A	262	287	GGGSEVLDRGDEVPSLFMTNVWTPSN	26	0.747
8	CTL-MPV-1	A	478	498	GGSAESMKVGAAFTLISMYSE	21	0.701
9	CTL-MPV-1	A	719	738	GVINSIKLINLDMRLNHIEE	20	0.694
10	CTL-MPV-1	A	658	675	TTIDRSMGALNINRGYLE	18	0.69
11	CTL-MPV-1	A	190	211	LRIFFLSITKLNDSGIYMIPRT	22	0.673
12	CTL-MPV-1	A	437	460	GGGGSIQLDKHQALRIFFLSITKL	24	0.67
13	CTL-MPV-1	A	309	329	GSEENSKIVFIEISDQRLSIG	21	0.669
14	CTL-MPV-1	A	694	711	DFSNTFFPHDTDRLNYGG	18	0.664
15	CTL-MPV-1	A	510	529	DPDIEAVGGGGSEEMEGLMR	20	0.66
16	CTL-MPV-1	A	396	410	LDKVEPEIDENGSMI	15	0.643
17	CTL-MPV-1	A	55	75	EAWRIYPVLLPQMELLGGGGS	21	0.633
18	CTL-MPV-1	A	135	150	YLDKVEPEIDENGSMI	16	0.624
19	CTL-MPV-1	A	622	637	EIGPRAPYMVLLEESI	16	0.574
20	CTL-MPV-1	A	367	374	VNWRGGGG	8	0.534
21	CTL-MPV-1	A	95	102	CPMLPKLQ	8	0.531
**No.**	CTL-MPV-2	**Chain**	**Start**	**End**	**Peptide**	**Number of residues**	**Score**
1	CTL-MPV-2	A	5	59	PVTCLKSGAICHPVFCPRRYKQIGTCGLPGTKCCKKPEAAAKKSDKTGKKCYYLT	55	0.801
2	CTL-MPV-2	A	722	752	PYHWSIEAAAKGIINTLQKYYCRVRGGRCAV	31	0.78
3	CTL-MPV-2	A	332	366	PNLPYKVKKEICAKQAQLYFERLRMNLRALGGGGS	35	0.773
4	CTL-MPV-2	A	228	241	VYPREVLSYGGGGS	14	0.77
5	CTL-MPV-2	A	488	534	VTSYMIYLMNWCDFGGGGSHVDTSSRSWNTSDLDFVIFYASLTYLRR	47	0.729
6	CTL-MPV-2	A	259	289	VKDEHELLKTLFGGGGSKTPEDDIFIHYPKG	31	0.714
7	CTL-MPV-2	A	754	767	SCLPKEEQIGKCST	14	0.713
8	CTL-MPV-2	A	574	597	IQRYLPSGAQRLFIGEGSGSMMLL	24	0.71
9	CTL-MPV-2	A	309	317	LSAYETNTR	9	0.691
10	CTL-MPV-2	A	185	201	CVKNWESFCGIQFDCFM	17	0.682
11	CTL-MPV-2	A	655	704	YSNPDSTEVYLGGGGSAEGSDDIQLDPVVTDVVYGGGGSCLVSDAKVLSY	50	0.678
12	CTL-MPV-2	A	127	152	GGGSFTDQKIRSMFIDDLLSILNIDN	26	0.654
13	CTL-MPV-2	A	600	609	STLGQSISFY	10	0.649
14	CTL-MPV-2	A	220	226	PIKDEWD	7	0.618
15	CTL-MPV-2	A	83	95	DIKYQPLISRSNA	13	0.604
16	CTL-MPV-2	A	434	437	RRPR	4	0.603
17	CTL-MPV-2	A	630	642	PGFPISRLFNMYR	13	0.599
18	CTL-MPV-2	A	473	478	RGGGGS	6	0.597

No.	HTL-MPV-1	Chain	Start	End	Peptide	Number of residues	Score
1	HTL-MPV-1	A	505	587	TRSTDNQAMIKDALQSIQQQIKGLADGGGGSAMDEGYFAYSHLEKIGSCSRGVSGGGG SPEVCWEGVYNDAFLIDRINWISAG	83	0.839
2	HTL-MPV-1	A	426	489	MLSMIILYVLSIASLCIGLITFISFIIVEKKRNTYSRLEDRRVGGGGSDSKILSAFNTVIALLG	64	0.815
3	HTL-MPV-1	A	241	272	VAIGIATAAQITAGGGGGSKQTELSLDLALSK	32	0.722
4	HTL-MPV-1	A	53	102	LTLFRRTKKKYRRHTDDQAFNNPASKTEQKHGGGGSCLRMMEVLKEEAWR	50	0.71
5	HTL-MPV-1	A	369	379	VSFNNDNSEWI	11	0.641
6	HTL-MPV-1	A	115	152	EKEGGGGSQKVQMTYNWTQWLQTLYTMIMEENVPDMDG	38	0.638
7	HTL-MPV-1	A	173	185	ILMISECSVGIGG	13	0.612
8	HTL-MPV-1	A	225	233	HDLVGDVRL	9	0.607
9	HTL-MPV-1	A	212	222	QCTGGGGGSKN	11	0.538
10	HTL-MPV-1	A	20	32	CPRRYKQIGTCGL	13	0.521
**No.**	HTL-MPV-1	**Chain**	**Start**	**End**	**Peptide**	**Number of residues**	**Score**
1	HTL-MPV-2	A	623	663	CRVRGGRCAVLSCLPKEEQIGKCSTRGRKCCRRKKHHHHHH	41	0.832
2	HTL-MPV-2	A	1	43	GIGDPVTCLKSGAICHPVFCPRRYKQIGTCGLPGTKCCKKPEA	43	0.808
3	HTL-MPV-2	A	542	578	YAPEIAVSKEDGGGGSPQTSRNVNLDSIKLYTSDDEE	37	0.755
4	HTL-MPV-2	A	582	607	LEFEDEFAGSSSEVIVGISPEDEEPS	26	0.705
5	HTL-MPV-2	A	154	176	DVIRSPSAAEGGGGSERPGALIR	23	0.697
6	HTL-MPV-2	A	85	116	IAFNLLVYGGGGSPSIPREFMIYDDVFIDNTG	32	0.695
7	HTL-MPV-2	A	306	329	VVYHDHGGECTGGGGSLEQQSLFS	24	0.674
8	HTL-MPV-2	A	416	438	LISTEFSINETLTLDVTSPISNN	23	0.667
9	HTL-MPV-2	A	463	481	NDGGGGSDDLKKYYQIDQP	19	0.638
10	HTL-MPV-2	A	485	502	PTKITSGGGGSAEGSDDI	18	0.611
11	HTL-MPV-2	A	137	144	PATNSPEL	8	0.61
12	HTL-MPV-2	A	179	209	LNDPDIEAVIIDVGSMVNGIPVMGGGGSSKG	31	0.602
13	HTL-MPV-2	A	281	287	SAGRQGG	7	0.578
14	HTL-MPV-2	A	290	303	SAEGSDDIQLDPVV	14	0.578
15	HTL-MPV-2	A	119	126	LKGGGGGS	8	0.562
16	HTL-MPV-2	A	336	343	FRDGSLTN	8	0.508

Table S14: B cell Discontinuous epitopes screened from CTL MPVs.

No.		Residues	Number of residues	Score
1	CTL-MPV-1	A:G1, A:I2, A:G3, A:D4, A:P5, A:V6, A:T7, A:C8, A:L9, A:K10, A:S11, A:G12, A:A13, A:I14, A:C15, A:H16, A:P17, A:V18, A:F19, A:C20, A:P21, A:R22, A:R23, A:K25, A:Q26, A:I27, A:G28, A:T29, A:C30, A:G31, A:L32, A:P33, A:G34, A:T35, A:K36, A:C37, A:K39, A:K40, A:P41, A:V51, A:E55, A:A56, A:W57, A:R58, A:I59, A:Y60, A:P61, A:V62, A:L63, A:L64, A:P65, A:M67, A:E68, A:L69, A:C94, A:C95, A:P96, A:M97, A:L98, A:P99, A:L101, A:Q102, A:L136, A:D137, A:K138, A:V139, A:E140, A:P141, A:E142, A:I143, A:D144, A:E145, A:N146, A:G147, A:S148, A:I150, A:K154, A:P158, A:G159, A:A160, A:N161, A:E162, A:K164, A:Y165, A:N166, A:N167, A:Y168, A:M169, A:Y170, A:R191, A:F193, A:L195, A:S196, A:I197, A:T198, A:K199, A:L200, A:N201, A:D202, A:S203, A:G204, A:I205, A:Y206, A:M207, A:I208, A:P209, A:T211, A:F215, A:G225, A:K226, A:N227, A:P228, A:S229, A:K230, A:V231, A:I232, A:K233, A:S234, A:Y235, A:Y236, A:G237, A:T238, A:M239, A:D240, A:I241, A:K242, A:K243, A:I244, A:N245, A:E246, A:G247, A:L248, A:L249, A:K252, A:G261, A:G262, A:G263, A:G264, A:S265, A:E266, A:V267, A:L268, A:D269, A:R270, A:G271, A:D272, A:E273, A:V274, A:P275, A:S276, A:L277, A:F278, A:M279, A:T280, A:N281, A:V282, A:W283, A:T284, A:P285, A:S286, A:N287, A:E301, A:F302, A:Y303, A:G309, A:S310, A:E311, A:E312, A:N313, A:S314, A:K315, A:I316, A:V317, A:F318, A:I319, A:E320, A:I321, A:S322, A:D323, A:Q324, A:L326, A:S327, A:I328, A:G329, A:S330, A:F356, A:G357, A:V359, A:Q360, A:N363, A:P364, A:R370, A:G394, A:L396, A:D397, A:K398, A:V399, A:E400, A:P401, A:E402, A:I403, A:D404, A:E405, A:N406, A:G407, A:M409, A:I410, A:Y413, A:K414, A:T417, A:P418, A:G437, A:G438, A:G439, A:G440, A:S441, A:I442, A:Q443, A:L444, A:D445, A:H447, A:Q448, A:A449, A:L450, A:R451, A:I452, A:F453, A:F454, A:L455, A:S456, A:I457, A:T458, A:K459, A:L460, A:N461, A:D462, A:S463, A:G478, A:G479, A:S480, A:A481, A:E482, A:S483, A:M484, A:K485, A:V486, A:G487, A:A488, A:A489, A:F490, A:T491, A:L492, A:I493, A:S494, A:M495, A:Y496, A:S497, A:E498, A:P500, A:D510, A:P511, A:D512, A:I513, A:E514, A:A515, A:V516, A:G517, A:G518, A:G519, A:G520, A:S521, A:E522, A:E523, A:M524, A:E525, A:G526, A:M528, A:R529, A:V572, A:T573, A:A574, A:P575, A:D576, A:T577, A:A578, A:E579, A:E580, A:S581, A:E582, A:T583, A:R584, A:R585, A:K588, A:Y589, A:Q591, A:Q592, A:K593, A:R594, A:E622, A:I623, A:G624, A:P625, A:R626, A:A627, A:P628, A:M630, A:V631, A:L633, A:E634, A:E635, A:S636, A:I637, A:Q638, A:T658, A:T659, A:I660, A:D661, A:R662, A:S663, A:M664, A:G665, A:A666, A:L667, A:N668, A:I669, A:N670, A:R671, A:G672, A:Y673, A:L674, A:E675, A:Y678, A:D694, A:F695, A:S696, A:N697, A:T698, A:F699, A:F700, A:P701, A:H702, A:D703, A:T704, A:D705, A:R706, A:L707, A:N708, A:Y709, A:G710, A:G711, A:G712, A:G713, A:G719, A:V720, A:I721, A:N722, A:S723, A:I724, A:K725, A:L726, A:I727, A:N728, A:L729, A:D730, A:M731, A:R732, A:L733, A:N734, A:E737, A:N761, A:T762, A:A763, A:L764, A:S765, A:T766, A:I767, A:E768, A:G769, A:H770, A:L771, A:V772, A:S773, A:M774, A:E775, A:A776, A:A777, A:A778, A:K779, A:G780, A:I781, A:I782, A:N783, A:T784, A:L785, A:Q786, A:G795, A:R796, A:C797, A:A798, A:V799, A:L800, A:S801, A:C802, A:L803, A:P804, A:K805, A:E806, A:Q808, A:I809, A:G810, A:K811, A:C812, A:S813, A:T814, A:R815, A:G816, A:R817, A:K818, A:C819, A:C820, A:R821, A:K823, A:K824, A:H825, A:H826, A:H827, A:H828, A:H829, A:H830	429	0.71
2	CTL-MPV-1	A:G371, A:G372, A:G373, A:G374, A:S375	5	0.511
3	CTL-MPV-1	A:G71, A:G72, A:G73, A:G74, A:S75, A:I76	6	0.51
**No.**		**Residues**	**Number of residues**	**Score**
1	CTL-MPV-2	A:Y723, A:H724, A:E728, A:A729, A:A730, A:A731, A:K732, A:G733, A:I734, A:I735, A:N736, A:T737, A:L738, A:Q739, A:K740, A:Y741, A:Y742, A:C743, A:R744, A:V745, A:R746, A:G747, A:G748, A:R749, A:C750, A:A751, A:V752	27	0.824
2	CTL-MPV-2	A:G1, A:I2, A:G3, A:P5, A:V6, A:T7, A:C8, A:L9, A:K10, A:S11, A:G12, A:A13, A:I14, A:C15, A:H16, A:P17, A:V18, A:F19, A:C20, A:P21, A:R22, A:R23, A:Y24, A:K25, A:Q26, A:I27, A:G28, A:T29, A:C30, A:G31, A:L32, A:P33, A:G34, A:T35, A:K36, A:C37, A:C38, A:K39, A:K40, A:P41, A:E42, A:A43, A:A44, A:A45, A:K46, A:K47, A:S48, A:D49, A:K50, A:T51, A:G52, A:K53, A:K54, A:C55, A:Y56, A:Y57, A:L58, A:T59	58	0.784
3	CTL-MPV-2	A:Y491, A:Y494, A:L495, A:M496	4	0.763
4	CTL-MPV-2	A:E172, A:K174, A:G178, A:T181, A:I182, A:C185, A:V186, A:K187, A:N188, A:W189, A:E190, A:S191, A:F192, A:C193, A:G194, A:I195, A:Q196, A:P220, A:I221, A:K222, A:D223, A:E224, A:W225, A:D226, A:V228, A:Y229, A:P230, A:R231, A:E232, A:V233, A:L234, A:S235, A:Y236, A:G237, A:G238, A:G239, A:G240, A:S241, A:E242, A:D261, A:H263, A:E264, A:L265, A:L266, A:K267, A:T268, A:L269, A:F270, A:G271, A:G272, A:G273, A:G274, A:S275, A:K276, A:T277, A:P278, A:E279, A:D280, A:D281, A:I282, A:F283, A:I284, A:H285, A:Y286, A:P287, A:K288, A:L309, A:S310, A:A311, A:Y312, A:E313, A:T314, A:N315, A:T316, A:H331, A:P332, A:N333, A:L334, A:P335, A:Y336, A:K337, A:V338, A:K339, A:K340, A:E341, A:I342, A:C343, A:A344, A:K345, A:Q346, A:A347, A:Q348, A:L349, A:Y350, A:F351, A:E352, A:R353, A:L354, A:R355, A:M356, A:L358, A:R359, A:A360, A:G362, A:G363, A:G364, A:G365, A:S366, A:D367, A:I368	110	0.733
5	CTL-MPV-2	A:R434, A:R435, A:P436, A:R437, A:G438, A:W498, A:C499, A:D500, A:F501, A:G502, A:G503, A:G504, A:G505, A:S506, A:H507, A:V508, A:D509, A:T510, A:S511, A:S512, A:R513, A:S514, A:W515, A:N516, A:T517, A:S518, A:D519, A:L520, A:D521, A:F522, A:V523, A:I524, A:Y526, A:A527, A:S528, A:L529, A:T530, A:Y531, A:L532, A:R533, A:I536, A:I574, A:Q575, A:L578, A:P579, A:S580, A:G581, A:A582, A:Q583, A:R584, A:L585, A:F586, A:I587, A:G588, A:E589, A:G590, A:S591, A:G592, A:S593, A:M594, A:L596, A:L597, A:S600, A:T601, A:L602, A:G603, A:Q604, A:S605, A:I606, A:S607, A:F608, A:Y609, A:Y628, A:T629, A:P630, A:G631, A:F632, A:P633, A:I634, A:S635, A:R636, A:L637, A:F638, A:N639, A:M640, A:Y641, A:R642, A:S643, A:F652, A:S656, A:N657, A:P658, A:D659, A:S660, A:T661, A:E662, A:V663, A:Y664, A:L665, A:G666, A:G667, A:G668, A:G669, A:S670, A:A671, A:E672, A:G673, A:S674, A:D675, A:D676, A:I677, A:Q678, A:L679, A:D680, A:P681, A:V682, A:V683, A:T684, A:D685,	159	0.679

		A:V686, A:V687, A:Y688, A:G689, A:G690, A:G691, A:G692, A:S693, A:C694, A:L695, A:V696, A:S697, A:D698, A:A699, A:K700, A:V701, A:L702, A:S703, A:Y704, A:L753, A:S754, A:C755, A:L756, A:P757, A:K758, A:E759, A:E760, A:Q761, A:I762, A:G763, A:K764, A:C765, A:S766, A:T767, A:R768, A:G769, A:R770, A:C772, A:H778, A:H779		
6	CTL-MPV-2	A:Q484, A:N485, A:V488, A:T489, A:M492, A:I493	6	0.657
7	CTL-MPV-2	A:D83, A:I84, A:K85, A:Y86, A:Q87, A:P88, A:L89, A:I90, A:S91, A:R92, A:N94, A:A95, A:G98, A:L99, A:P102, A:L103, A:P105, A:V106, A:M107, A:G108, A:R110, A:L125, A:G127, A:G128, A:G129, A:S130, A:F131, A:T132, A:D133, A:Q134, A:K135, A:I136, A:R137, A:S138, A:M139, A:F140, A:I141, A:D142, A:D143, A:L144, A:L145, A:S146, A:I147, A:L148, A:N149, A:I150, A:D151, A:N152, A:K369, A:L370, A:G371	51	0.628
8	CTL-MPV-2	A:G474, A:G475, A:G476, A:G477, A:S478, A:K481	6	0.626
9	CTL-MPV-2	A:F197, A:D198, A:C199, A:F200	4	0.614
10	CTL-MPV-2	A:S726, A:I727, A:K771	3	0.509
11	CTL-MPV-2	A:E255, A:N256, A:G257, A:V259, A:K260	5	0.507
**No.**		**Residues**	**Number of residues**	**Score**
1	HTL-MPV-1	A:T505, A:T508, A:D509, A:N510, A:Q511, A:A512, A:M513, A:I514, A:K515, A:D516, A:A517, A:L518, A:Q519, A:S520, A:I521, A:Q522, A:Q523, A:Q524, A:I525, A:K526, A:G527, A:L528, A:A529, A:D530, A:G531, A:G532, A:G533, A:G534, A:S535, A:A536, A:M537, A:D538, A:E539, A:G540, A:Y541, A:F542, A:A543, A:Y544, A:S545, A:H546, A:L547, A:E548, A:K549, A:I550, A:G551, A:S552, A:C553, A:S554, A:R555, A:G556, A:V557, A:S558, A:G559, A:G560, A:G561, A:G562, A:S563, A:P564, A:E565, A:V566, A:C567, A:W568	62	0.878
2	HTL-MPV-1	A:C20, A:P21, A:R22, A:R23, A:K25, A:Q26, A:G28, A:C30, A:L32, A:V369, A:S370, A:F371, A:N372, A:N373, A:D374, A:N375, A:S376, A:E377, A:W378, A:I379, A:S425, A:M426, A:L427, A:S428, A:M429, A:I430, A:I431, A:L432, A:Y433, A:V434, A:L435, A:S436, A:I437, A:A438, A:S439, A:L440, A:C441, A:I442, A:G443, A:L444, A:I445, A:T446, A:F447, A:I448, A:S449, A:F450, A:I451, A:I452, A:V453, A:E454, A:K455, A:K456, A:R457, A:N458, A:T459, A:Y460, A:S461, A:R462, A:L463, A:E464, A:D465, A:R466, A:R467, A:V468, A:G469, A:G470, A:G471, A:G472, A:S473, A:D474, A:S475, A:K476, A:I477, A:L478, A:S479, A:A480, A:F481, A:N482, A:T483, A:V484, A:I485, A:A486, A:L487, A:L488, A:G489, A:V492	86	0.758
3	HTL-MPV-1	A:E569, A:G570, A:V571, A:Y572, A:N573, A:D574, A:A575, A:F576, A:L577, A:I578, A:D579, A:R580, A:I581, A:N582, A:W583, A:I584, A:S585, A:A586, A:G587, A:V588	20	0.727
4	HTL-MPV-1	A:L53, A:T54, A:F56, A:R57, A:R58, A:T59, A:K60, A:K61, A:K62, A:Y63, A:R64, A:R65, A:H66, A:T67, A:D68, A:D69, A:Q70, A:A71, A:F72, A:N73, A:N74, A:P75, A:A76, A:S77, A:K78, A:T79, A:E80, A:Q81, A:K82, A:H83, A:G84, A:G85, A:G86, A:G87, A:S88, A:C89, A:L90, A:R91, A:M92, A:M93, A:E94, A:V95, A:L96, A:K97, A:E98, A:E99, A:W101, A:R102, A:G215, A:G216, A:G218, A:G219, A:S220, A:K221, A:N222, A:N223, A:G240, A:V241, A:A242, A:I243, A:G244, A:I245, A:A246, A:T247, A:A248, A:A249, A:Q250, A:I251, A:T252, A:A253, A:G254, A:G255, A:G256, A:G257, A:G258, A:S259, A:K260, A:Q261, A:T262, A:E263, A:L264, A:S265, A:L266, A:D267, A:L268, A:A269, A:L270, A:S271, A:K272, A:L341, A:S342, A:S343, A:Y344	93	0.69
5	HTL-MPV-1	A:H225, A:D226, A:L227, A:V228, A:G229, A:D230, A:V231, A:R232	8	0.609
6	HTL-MPV-1	A:P109, A:E112, A:L113, A:E115, A:K116, A:E117, A:G118, A:G119, A:G120, A:G121, A:S122, A:Q123, A:K124, A:V125, A:Q126, A:M127, A:N130, A:W131, A:T132, A:Q133, A:W134, A:L135, A:Q136, A:T137, A:L138, A:Y139, A:T140, A:M141, A:I142, A:M143, A:E144, A:E145, A:N146, A:V147, A:P148, A:D149, A:M150, A:D151, A:G152, A:G155, A:M157, A:L170, A:I171, A:I173, A:L174, A:I176, A:S177, A:E178, A:C179, A:S180, A:V181, A:G182, A:I183, A:G184, A:G185, A:G187	56	0.608
7	HTL-MPV-1	A:P205, A:N206, A:S208, A:N209, A:Q212, A:C213	6	0.556
**No.**		**Residues**	**Number of residues**	**Score**
1	HTL-MPV-2	A:A589, A:G590, A:S591, A:S592, A:S593, A:E594, A:V595, A:I596, A:V597, A:G598, A:I599, A:S600, A:P601, A:E602, A:D603, A:E604	16	0.799
2	HTL-MPV-2	A:N463, A:D464, A:G465, A:G466, A:G467, A:G468, A:S469, A:D470, A:D471, A:L472, A:K473, A:K474, A:Y475, A:Y476, A:Q477, A:I478, A:D479, A:Q480, A:P481, A:F482, A:L540, A:S541, A:Y542, A:A543, A:P544, A:E545, A:I546, A:A547, A:V548, A:S549, A:K550, A:D552, A:G553, A:G554, A:G555, A:G556, A:S557, A:P558, A:Q559, A:T560, A:S561, A:R562, A:N563, A:V564, A:N565, A:L566, A:D567, A:S568, A:I569, A:L571, A:T573, A:S574, A:D575, A:D576, A:E577, A:E578, A:C623, A:V625, A:R626, A:G627, A:G628, A:R629, A:C630, A:A631, A:V632, A:L633, A:S634, A:C635, A:L636, A:P637, A:K638, A:E639, A:E640, A:Q641, A:I642, A:G643, A:K644, A:C645, A:S646, A:T647, A:R648	81	0.761
3	HTL-MPV-2	A:L416, A:I417, A:S418, A:T419, A:F421, A:S422, A:I423, A:N424, A:E425, A:T426, A:L427, A:T428, A:L429, A:D430, A:V431, A:T432, A:S433, A:P434, A:S436, A:N437, A:N438, A:T489, A:S490, A:G491, A:G492, A:G493, A:G494, A:S495, A:A496, A:E497, A:G498, A:S499, A:D500, A:D501, A:I502, A:Q503, A:L504, A:G649, A:R650, A:K651, A:C652, A:C653, A:R654, A:R655, A:K656, A:K657, A:H658, A:H659, A:H660, A:H661, A:H662, A:H663	52	0.682
4	HTL-MPV-2	A:G1, A:I2, A:G3, A:D4, A:P5, A:V6, A:T7, A:C8, A:L9, A:S11, A:G12, A:A13, A:I14, A:C15, A:H16, A:P17, A:V18, A:F19, A:C20, A:P21, A:R22, A:Y24, A:K25, A:Q26, A:I27, A:G28, A:T29, A:C30, A:G31, A:L32, A:P33, A:G34, A:T35, A:K36, A:C37, A:C38, A:K39, A:K40, A:P41, A:E42, A:A43, A:K46, A:Q47, A:L48, A:D49, A:L150, A:F151, A:D154, A:V155, A:I156, A:S158, A:P159, A:S160, A:A161, A:A162, A:E163, A:G164, A:G165, A:G166, A:G167, A:S168, A:E169, A:R170, A:P171, A:G172, A:A173, A:L174, A:R176, A:L179, A:N180, A:D181, A:P182, A:D183, A:I184, A:E185, A:A186, A:V187, A:I188, A:I189, A:D190, A:V191, A:G192, A:S193, A:M194, A:V195, A:N196, A:G197, A:I198, A:P199, A:V200, A:M201, A:G202, A:G203,	157	0.666

		A:G204, A:G205, A:S206, A:S207, A:K208, A:G209, A:K210, A:V214, A:D215, A:G244, A:G245, A:G246, A:R249, A:H250, A:A252, A:G253, A:S281, A:A282, A:G283, A:R284, A:Q285, A:G286, A:G287, A:G288, A:G289, A:S290, A:A291, A:E292, A:G293, A:S294, A:D295, A:D296, A:I297, A:Q298, A:L299, A:D300, A:P301, A:V302, A:V303, A:V306, A:V307, A:Y308, A:H309, A:D310, A:H311, A:G312, A:G313, A:E314, A:C315, A:T316, A:G317, A:G318, A:G319, A:G320, A:S321, A:L322, A:E323, A:Q324, A:G339, A:S340, A:L341, A:T342, A:N343, A:D372		
5	HTL-MPV-2	A:P137, A:A138, A:T139, A:N140, A:S141, A:E143, A:L144, A:E147	8	0.639
6	HTL-MPV-2	A:R80, A:N83, A:I85, A:A86, A:F87, A:N88, A:L89, A:L90, A:V91, A:Y92, A:G93, A:G94, A:G95, A:G96, A:S97, A:P98, A:S99, A:I100, A:P101, A:R102, A:E103, A:F104, A:M105, A:I106, A:Y107, A:D108, A:D109, A:V110, A:F111, A:I112, A:D113, A:N114, A:T115, A:G116, A:R117, A:L119, A:K120, A:G121, A:G122, A:G123, A:G124, A:G125, A:S126, A:T127, A:V136, A:N335, A:R337	47	0.62
7	HTL-MPV-2	A:A579, A:L582, A:E583, A:E585, A:N616, A:Q619	6	0.558
8	HTL-MPV-2	A:E605, A:P606, A:S607	3	0.55

Table S15: Analysis of codon-optimized cDNA of all the MPVs.

S.No.	MPVs	GC content	CAI (Codon Adaptation Index) score	Tandem rare codons
1	CTL-MPV-1	64.74%	1	0%
2	CTL-MPV-2	66.06%	1	0%
3	HTL-MPV-1	64.86%	1	0%
4	HTL-MPV-2	67.96%	1	0%
	Ideal values	30-70%	0.8-1.0	<30%

SUPPLEMENTARY txt 1: CTL 'GaEl Ag-Patches' from NIPAH Proteome:

```
C Protein

9 overlapping epitopes
--MEMLKEETW-------------
-MMEMLKEETW-------------
RMMEMLKEETW-------------
----MLKEETWRIY----------
--MEVLKEEAW-------------
RMMEVLKEEAW-------------
------------IYPVLLPQM---
-------------YPVLLPQM---
---------------VLLPQMELL
58-81
RMMEVLKEEAWRIYPVLLPQMELL


11 overlapping epitopes
---------------------------FLKKIGKL
------------------------KLQFLKKIGK-
--------------------CPMLPKLQF-------
-----------------RCCPMLPKLQF-------
```

```
-------------YLMQRCCPML------------
---------MYVLYLMQR-----------------
----EQTMGMYVLY---------------------
---QEQTMGMYVLY---------------------
-----QTMGMYVLY---------------------
--HQEQTMGMY------------------------
ITHQEQTMGMY------------------------
131-165
ITHQEQTMGMYVLYLMQRCCPMLPKLQFLKKIGKL
```

Fusion Protein

12 overlapping epitopes

```
----------------------------------------KMIPNVSNM
-------------------------------------IVIKMIPNV---
---------------------------------LTKDIVIKM-------
-------------------------------NPLTKDIVI---------
---------------------------KIKSNPLTK-------------
-------------------------KYKIKSNPLTK-------------
------------------LVKGVTRKY----------------------
-----------------GLVKGVTRK-----------------------
------------KLSKIGLVK----------------------------
----------YEKLSKIGL------------------------------
ISECSVGILHY--------------------------------------
-SECSVGILHY--------------------------------------
>gi|13559813|ref|NP_112026.1|/20-68
ISECSVGILHYEKLSKIGLVKGVTRKYKIKSNPLTKDIVIKMIPNVSNM
```

25 overlapping epitopes

```
---------------------------------------------------------FILVRNTL
------------------------------------------------------VPNFILVRNTL
----------------------------------------------------SIVPNFILV----
-------------------------------------------------EWISIVPNF-------
------------------------------------------------SEWISIVPNF-------
-----------------------------------------VSFNNDNSEW--------------
---------------------------------AYIQELLPVSF---------------------
------------------------------IQQAYIQEL--------------------------
--------------------------ILTEIQQAY------------------------------
---------------------------LTEIQQAY------------------------------
------------------------FPILTEIQQAY------------------------------
----------------------VYFPILTEI----------------------------------
----------------YYIIVRVYF----------------------------------------
---------------SYYIIVRVY-----------------------------------------
---------------SYYIIVRVYF----------------------------------------
--------------SSYYIIVRV------------------------------------------
----------YVDLSSYY-----------------------------------------------
---------IYVDLSSYY-----------------------------------------------
--------IIYVDLSSYY-----------------------------------------------
--------IIYVDLSSY------------------------------------------------
------GQIIYVDLSSY------------------------------------------------
---SITGQIIYV-----------------------------------------------------
--DSITGQIIY------------------------------------------------------
--DSITGQIIYV-----------------------------------------------------
ESDSITGQIIY------------------------------------------------------
>gi|13559813|ref|NP_112026.1|/258-322
ESDSITGQIIYVDLSSYYIIVRVYFPILTEIQQAYIQELLPVSFNNDNSEWISIVPNFILVRNTL
```

Glycoprotein

```
10 overlapping epitopes
------IPSKVIKSY-------------------------
ASDKGKNPSK------------------------------
---KGKNPSKVIK---------------------------
------NPSKVIKSY-------------------------
------------KSYYGTMDIK------------------
------------KSYYGTMDIKK-----------------
-------------------------------KILSAFNTV
----------------------KINEGLLDSK--------
--------------------------GLLDSKILSA----
---------------------------LLDSKILSA----
----------------------------------------
----------------------------------------
14-53
ASDKGKNPSKVIKSYYGTMDIKKINEGLLDSKILSAFNTV
```

```
12 overlapping epitopes
-------------------------------VYNNEFYYV
------------------------------AVYNNEFYY-
-----------------------------SAVYNNEFY--
-----------------------------SAVYNNEFYY-
------------------------TVYHCSAVY-------
------------------TPSNPNTVY-------------
-----------------WTPSNPNTVY-------------
----------------VWTPSNPNTVY-------------
--------VPSLFMTNVW----------------------
--------VPSLFMTNV-----------------------
-------EVPSLFMTNV-----------------------
EVLDRGDEV-------------------------------
254-293
EVLDRGDEVPSLFMTNVWTPSNPNTVYHCSAVYNNEFYYV
```

```
19 overlapping epitopes
--------------------------------------------------TVNPLVVNWR
--------------------------------------------------TVNPLVVNW-
-------------------------------------------------LTVNPLVVNW-
-------------------------------------------------QTVNPLVVNW-
-----------------------------------------------DVQTVNPLV----
-------------------------------------FSWDTMIKF--------------
-----------------------------------ASFSWDTMIK---------------
-------------------------------VFYQASFSW--------------------
-----------------------------QPVFYQASF----------------------
-----------------------------QPVFYQASFSW--------------------
-------------------------DSLGQPVFY--------------------------
-----------------------IYDSLGQPVF---------------------------
----------------------KIYDSLGQPV----------------------------
----------------------KIYDSLGQPVF---------------------------
-------------------SPSKIYDSL--------------------------------
----------------SIGSPSKIY-----------------------------------
--------------RLSIGSPSK-------------------------------------
-------EISDQRLSI--------------------------------------------
EENSKIVFI---------------------------------------------------
421-480
EENSKIVFIEISDQRLSIGSPSKIYDSLGQPVFYQASFSWDTMIKFGDVQTVNPLVVNWR
```

Matrix Protein

11 overlapping epitopes

```
---------------------------------------------------NYMYLICYGF
------------------------------------------------KYNNYMYLI----
--------------------------------------KIYTPGANER-------------
--------------------------------------KIYTPGANERK------------
--------------------------------SMIPKYKIY--------------------
----------------------------DENGSMIPKY-----------------------
-------------------YLDKVEPEI---------------------------------
----------SPSSWEHGGYL----------------------------------------
------VSDFSPSSW----------------------------------------------
-ESMEGVSDF---------------------------------------------------
SESMEGVSDF---------------------------------------------------
10-70
SESMEGVSDFSPSSWEHGGYLDKVEPEIDENGSMIPKYKIYTPGANERKYNNYMYLICYGF
```

8 overlapping epitopes

```
------------------------YMIPRTML--
------------------------YMIPRTMLEF
-----------------------IYMIPRTML--
-----------------------IYMIPRTMLEF
-----------------KLNDSGIYM--------
---------RIFFLSITK----------------
-QLDKHQAL-------------------------
IQLDKHQAL-------------------------
163-196
IQLDKHQALRIFFLSITKLNDSGIYMIPRTMLEF
```

Nucleoprotein

9 overlapping epitopes

```
-------------------------SLLNDPDIEAV
----------------------LTNSLLNLR-----
---------------------RLTNSLLNLR-----
------------------RPGALIRSL---------
------------------RPGALIRSLL--------
---------FTLISMYSER-----------------
-------AAFTLISMY--------------------
-ESMKVGAAF--------------------------
AESMKVGAAF--------------------------
65-100
AESMKVGAAFTLISMYSERPGALIRSLLNDPDIEAV
```

9 overlapping epitopes

```
----------------------FVDSRAYGL
--------------------TPFVDSRAY--
-------------------KTPFVDSRAY--
-----------------KGKTPFVDSR----
---------------SSKGKTPFV-------
----------KTARDSSKGK-----------
----GLMRILKTA------------------
EEMEGLMRI----------------------
EEMEGLMRIL---------------------
123-153
EEMEGLMRILKTARDSSKGKTPFVDSRAYGL
```

15 overlapping epitopes

```
---------------------------------------PFFALTQQW
--------------------------------------NPFFALTQQW
---------------------------------QQKRVNPFF------
--------------------------------VQQKRVNPF-------
--------------------------------VQQKRVNPFF------
-------------------------------YVQQKRVNPF-------
-----------------------SETRRWAKY----------------
---------------------EESETRRWAKY----------------
---------------------EESETRRW-------------------
--------------------AEESETRRW-------------------
-------------------TAEESETRRW-------------------
------------------DTAEESETR---------------------
------------------DTAEESETRR--------------------
--------ILIAKAVTA-------------------------------
ITIEAQIW----------------------------------------
166-213
ITIEAQIWILIAKAVTAPDTAEESETRRWAKYVQQKRVNPFFALTQQW
```

25 overlapping epitopes

```
--------------------------------------------------------------YLEPMYFRL
-------------------------------------------------------------GYLEPMYFR-
-------------------------------------------------------------GYLEPMYFRL
------------------------------------------------------------RGYLEPMYFR-
-----------------------------------------------------------NRGYLEPMYFR-
---------------------------------------------------RSMGALNINR----------
-----------------------------------------------TTIDRSMGA---------------
-----------------------------------------FAMGVATTI---------------------
-------------------------------------LLWSFAMGV-------------------------
-----------------------------------YPLLWSFAM---------------------------
----------------------------------GYPLLWSF-----------------------------
-------------------------------APGGYPLLW-------------------------------
------------------------------FAPGGYPLLW-------------------------------
-----------------------------KFAPGGYPLLW-------------------------------
---------------------------QTKFAPGGY-----------------------------------
--------------------------IQTKFAPGGY-----------------------------------
----------------------LEESIQTKF----------------------------------------
------------IGPRAPYMVL-------------------------------------------------
-------------GPRAPYMVL-------------------------------------------------
-------------GPRAPYMVLL------------------------------------------------
----------REIGPRAPY----------------------------------------------------
-TIKSLMLLYR------------------------------------------------------------
-TIKSLMLLY-------------------------------------------------------------
NTIKSLMLL--------------------------------------------------------------
NTIKSLMLLY-------------------------------------------------------------
292-362
NTIKSLMLLYREIGPRAPYMVLLEESIQTKFAPGGYPLLWSFAMGVATTIDRSMGALNINRGYLEPMYFRL
```

Phosphoprotein

7 overlapping epitopes

```
-------------FPHDTDRLNY
-----------TFFPHDTDR---
----------NTFFPHDTDR---
---MPSDDFSNTF----------
```

```
---MPSDDFSNTFF---------
--IMPSDDFSNTF----------
KYIMPSDDF--------------
476-498
KYIMPSDDFSNTFFPHDTDRLNY
```

14 overlapping epitopes

```
--------------------------------------------------STIEGHLVSM
--------------------------------------------KTNTALSTI-------
-----------------------------------------VLAKTNTAL----------
----------------------------------------RVLAKTNTA-----------
------------------------------------ESIDRVLAK---------------
----------------------------------KLESIDRVLAK---------------
------------------------------KIINKLESI---------------------
----------------------------IPKIINKL------------------------
---------------------------EIPKIINKL------------------------
--------------------------KEIPKIINKL------------------------
-----------------RLNHIEEQV----------------------------------
----------KLINLDMRL-----------------------------------------
-VLMGVINSI--------------------------------------------------
SVLMGVINSI--------------------------------------------------
515-574
SVLMGVINSIKLINLDMRLNHIEEQVKEIPKIINKLESIDRVLAKTNTALSTIEGHLVSM
```

Polymerase

22 overlapping epitopes

```
---------------------------------------------------------------------SMIEPLVLA-
---------------------------------------------------------------------SMIEPLVLAL
---------------------------------------------------------------RIYNIVSMI-------
---------------------------------------------------------FPVMGNRI--------------
---------------------------------------------------------FPVMGNRIY-------------
--------------------------------------------------------LFPVMGNRIY-------------
---------------------------------------------------GLIDPLFPV-------------------
-------------------------------------------------LWGLIDPLF---------------------
------------------------------------------------ALWGLIDPL----------------------
------------------------------------------LISRSNALW----------------------------
----------------------------------------QPLISRSNAL-----------------------------
------------------------------------DIKYQPLISR---------------------------------
-------------------------------TTVKSDIKY---------------------------------------
------------------------------ETTVKSDIKY---------------------------------------
--------------------------RMMMETTVK--------------------------------------------
------------------MYCDVLEGR----------------------------------------------------
-----------LTPEMVLMY-----------------------------------------------------------
----------YLTPEMVLM------------------------------------------------------------
----------YLTPEMVLMY-----------------------------------------------------------
---------YYLTPEMVL-------------------------------------------------------------
KSDKTGKKCY---------------------------------------------------------------------
KSDKTGKKCYY--------------------------------------------------------------------
219-297
KSDKTGKKCYYLTPEMVLMYCDVLEGRMMMETTVKSDIKYQPLISRSNALWGLIDPLFPVMGNRIYNIVSMIEPLVLAL
```

11 overlapping epitopes

```
---------------------------------RTFGHPILEAK
---------------------------EFFSFFRTF--------
--------------------------AEFFSFFRTF--------
```

```
-----------------------HLLAEFFSF------------
-----------------------HLLAEFFSFF-----------
----------------------IHLLAEFFSF------------
----------------ILNIDNIHLL------------------
---------FIDDLLSIL--------------------------
-------SMFIDDLLSI---------------------------
------RSMFIDDLL-----------------------------
FTDQKIRSM-----------------------------------
328-371
FTDQKIRSMFIDDLLSILNIDNIHLLAEFFSFFRTFGHPILEAK
```

```
16 overlapping epitopes
-------------------------------------------------YPREVLSY
------------------------------------------------VYPREVLSY
-----------------------------------------------SVYPREVLSY
---------------------------------------SPIKDEWDSVY-------
-------------------------------------ALSPIKDEW-----------
------------------------------------KALSPIKDEW-----------
--------------------------------YMKDKALSPI---------------
------------------------------SMYMKDKAL------------------
------------------------KLDSDLSMY------------------------
-----------------------LKLDSDLSMY------------------------
----------------------ELKLDSDLSMY------------------------
---------------IQFDCFMEL---------------------------------
--------------GIQFDCFMEL---------------------------------
-----------SFCGIQFDCF------------------------------------
---------WESFCGIQF---------------------------------------
LTIDDCVKNW-----------------------------------------------
438-494
LTIDDCVKNWESFCGIQFDCFMELKLDSDLSMYMKDKALSPIKDEWDSVYPREVLSY
```

```
10 overlapping epitopes
-------------------DEHELLKTL-
-------------------DEHELLKTLF
---------KYFKENGMVK----------
--LIASGVGKY------------------
--LIASGVGKYF-----------------
-ALIASGVGK-------------------
-ALIASGVGKY------------------
-ALSHPRVFK-------------------
-ALSHPRVFKR------------------
EALIASGVGKY------------------
568-596
EALIASGVGKYFKENGMVKDEHELLKTLF
```

```
16 overlapping epitopes
-------------------------------------ETNTRIAAI
----------------------------------SAYETNTRI---
----------------------------IPFLFLSAY---------
---------------------------TIPFLFLSAY---------
--------------------------ATIPFLFLSAY---------
------------------------TIATIPFLF-------------
-----------------------WTIATIPFL--------------
-----------------------WTIATIPFLF-------------
----------------------TWTIATIPFLF-------------
```

```
---------------------KTWTIATIPF---------------
-----------------GYSQKTWTI--------------------
---------------IEGYSQKTW----------------------
--------------GIEGYSQKTW----------------------
----------YPKGGIEGY---------------------------
-TPEDDIFIHY-----------------------------------
KTPEDDIFIHY-----------------------------------
783-828
KTPEDDIFIHYPKGGIEGYSQKTWTIATIPFLFLSAYETNTRIAAI
```

12 overlapping epitopes

```
--------------------------RLRMNLRAL
------------------KQAQLYFER--------
--------------EICAKQAQLY-----------
-------------KEICAKQAQLY-----------
----------KVKKEICAK----------------
-------LPYKVKKEI-------------------
----HPNLPYKV-----------------------
--ILNPNLICI------------------------
--ILNPNLICIF-----------------------
--KVHPNLPYK------------------------
--KVHPNLPYKVK----------------------
TQKVHPNLPY-------------------------
839-873
TQKVHPNLPYKVKKEICAKQAQLYFERLRMNLRAL
```

15 overlapping epitopes

```
-------------------------WAYGDNEECW
-------------------IATVYTWAY-------
------------------RIATVYTW---------
------------------RIATVYTWAY-------
-----------------IRIATVYTWAY-------
----------------AIRIATVYTW---------
---------------SAIRIATV------------
---------------SAIRIATVY-----------
---------------SAIRIATVYTW---------
--------------RSAIRIATVY-----------
--------RPTKALRSA------------------
--------RPTKALRSAI-----------------
-----NVKRPTKAL---------------------
-----NVKRPTKALR--------------------
DIKLGNVKR--------------------------
1281-1315
DIKLGNVKRPTKALRSAIRIATVYTWAYGDNEECW
```

24 overlapping epitopes

```
----------------------------------------------------------LSHPRVFKR
-------------------------------------------------HTYKVLSNA---------
---------------------------------------------NTSKHTYKV-------------
-------------------------------------------VSNTSKHTY---------------
------------------------------------------LVSNTSKHTY---------------
----------------------------------TMVDLLSDL------------------------
----------------------------RPRGRHTM-------------------------------
----------------------------RPRGRHTMV------------------------------
----------------------------RPRGRHTMVDL----------------------------
----------------------------RLFAKM-TY------------------------------
```

----------------------------RLFAKM-TYK-----------------------------
----------------------------RLFAKM-TYKMR---------------------------
----------------------------RLRDKSTQF------------------------------
----------------------------RLRDKSTQFK-----------------------------
--------------------WAFEIHHRR--------------------------------------
-------------------KWAFEIHHRR--------------------------------------
--------------QSISIKWAF--
-------------GQSISIKWAF--
------------LGQSISIKW--
----------LYLGQSISI--
---------ALYLGQSISIK---
--------FALYLGQSI--
--IVDPELFALY---
FLIVDPELFAL--
1534-1600
FLIVDPELFALYLGQSISIKWAFEIHHRRPRGRHTMVDLLSDLVSNTSKHTYKVLSNALSHPRVFKR

10 overlapping epitopes
--------------IYLMNWCDF
-------------MIYLMNWCDF
-----------SYMIYLMNW---
----------TSYMIYLMNW---
---------VTSYMIYLMNW---
-------LLVTSYMIY-------
-------LLVTSYMIYL------
----SQNLLVTSY----------
---LSQNLLVTSY----------
FEKLSQNLL--------------
1620-1642
FEKLSQNLLVTSYMIYLMNWCDF

12 overlapping epitopes
------------------------YLRRGIIKQL
-------------------YASLTYLRR------
------------------FYASLTYLR-------
------------------FYASLTYLRR------
-----------------IFYASLTYLR-------
-----------------IFYASLTYLRR------
----------------VIFYASLTY---------
---------------FVIFYASLTY---------
----------TSDLDFVIFY--------------
---------NTSDLDFVIFY--------------
------RSWNTSDLDF------------------
HVDTSSRSW-------------------------
1706-1739
HVDTSSRSWNTSDLDFVIFYASLTYLRRGIIKQL

17 overlapping epitopes
---TLGQSISFY
--STLGQSISF-
--STLGQSISFY
--YQSTLGQSISF-
--MMLLYQSTL-------
--GSGSMMLLY-----------
--GEGSGSMML-------------
--------------------------------LPSGAQRLF-----------------------

```
-------------------------------YLPSGAQRL------------------------
-------------------------------YLPSGAQRLF-----------------------
------------------------------RYLPSGAQR-------------------------
------------------------------RYLPSGAQRL------------------------
------------------------------RYLPSGAQRLF-----------------------
-----------------------NLSPLIQRY--------------------------------
--------------------KALNLSPLIQR---------------------------------
----------RIGLNSSSCY--------------------------------------------
RPSVENHKY-------------------------------------------------------
1801-1864
RPSVENHKYRRIGLNSSSCYKALNLSPLIQRYLPSGAQRLFIGEGSGSMMLLYQSTLGQSISFY
```

```
17 overlapping epitopes
------------------------------------------NPDSTEVYL
----------------------------------------YSNPDSTEVY-
--------------------------------LVLVCFPVY----------
----------------------------SYFGLVLVCF-------------
---------------------------RSYFGLVLVCF-------------
---------------------RLFNMYRSY---------------------
---------------------RLFNMYRSYF--------------------
--------------------SRLFNMYRSY---------------------
-------------------ISRLFNMYR-----------------------
-----------------FPISRLFNM-------------------------
-----------------FPISRLFNMY------------------------
--------------TPGFPISRL----------------------------
--------------TPGFPISRLF---------------------------
-------------YTPGFPISRLF---------------------------
-------------YTPGFPISR-----------------------------
----DGLLVSKI---------------------------------------
VMMEDGLLV------------------------------------------
1966-2016
VMMEDGLLVSKIAYTPGFPISRLFNMYRSYFGLVLVCFPVYSNPDSTEVYL
```

V Protein

```
7 overlapping epitopes
---------DPVVTDVVY
--------LDPVVTDVVY
-------QLDPVVTDVVY
-------QLDPVVTDV--
------IQLDPVVTDV--
------IQLDPVVTDVV-
AEGSDDIQL---------
------------------
------------------
99-116
AEGSDDIQLDPVVTDVVY
```

```
5 overlapping epitopes
------KMLSYAPEI
---SDAKMLSY----
--VSDAKMLSY----
-LVSDAKMLSY----
CLVSDAKMLSY----
149-163
CLVSDAKMLSYAPEI
```

```
6 overlapping epitopes
------QPPYHWSI
-----AQPPYHWSI
----DAQPPYHW--
----DAQPPYHWSI
---KDAQPPYHW--
QQGKDAQPPY----
355-368
QQGKDAQPPYHWSI
```

W Protein

```
9 overlapping epitopes
------KMLSYAPEI
------KVLSYAPEI
---SDAKVLSY----
--VSDAKVLSY----
-LVSDAKVLSY----
--VSDAKMLSY----
-LVSDAKMLSY----
CLVSDAKVLSY----
CLVSDAKMLSY----
149-163
CLVSDAKVLSYAPEI
```

SUPPLEMENTARY txt 2: HTL 'GaEI Ag-Patches' from NIPAH Proteome:

C Protein

```
15 overlapping epitopes
----------------------DQVFNNPASKIKQKP
---------------------DDQVFNNPASKIKQK-
--------------------TDDQVFNNPASKIKQ--
-------------------HTDDQVFNNPASKIK---
------------------RHTDDQVFNNPASKI----
---------FRRTKKKYRRHTDDQ-------------
--------LFRRTKKKYRRHTDD--------------
-------TLFRRTKKKYRRHTD---------------
------LTLFRRTKKKYRRHT----------------
-----LLTLFRRTKKKYRRH-----------------
----ILLTLFRRTKKKYRR------------------
---SILLTLFRRTKKKYR-------------------
--ASILLTLFRRTKKKY--------------------
-MASILLTLFRRTKKK---------------------
MMASILLTLFRRTKK----------------------
>gi|1859635642|gb|QKV44002.1|/1-37
MMASILLTLFRRTKKKYRRHTDDQAFNNPASKTEQKH
```

```
13 overlapping epitopes
--------------IYPVLLPQMELLERE
-------------WRIYPVLLPQMELLE--
-----------TWRIYPVLLPQMELL---
----------ETWRIYPVLLPQMEL----
---------EETWRIYPVLLPQME-----
--------KEETWRIYPVLLPQM------
------MLKEETWRIYPVLLP--------
-----EMLKEETWRIYPVLL---------
----MEMLKEETWRIYPVL----------
---MMEMLKEETWRIYPV-----------
--RMMEMLKEETWRIYP------------
-LRMMEMLKEETWRIY-------------
CLRMMEMLKEETWRI--------------
>gi|1859635642|gb|QKV44002.1|/56-84
CLRMMEVLKEEAWRIYPVLLPQMELLEKE
```

```
13 overlapping epitopes
--------------TLYTMIMEENVPDMD
-------------QTLYTMIMEENVPDM-
------------LQTLYTMIMEENVPD--
-----------WLQTLYTMIMEENVP---
----------QWLQTLYTMIMEENV----
---------TQWLQTLYTMIMEEN-----
--------WTQWLQTLYTMIMEE------
-----TYNWTQWLQTLYTMI---------
----MTYNWTQWLQTLYTM----------
---QMTYNWTQWLQTLYT-----------
--VQMTYNWTQWLQTLY------------
-KVQMTYNWTQWLQTL-------------
QKVQMTYNWTQWLQT--------------
>gi|1859635642|gb|QKV44002.1|/92-120
QKVQMTYNWTQWLQTLYTMIMEENVPDMD
```

Fusion Protein

```
11 overlapping epitopes
------------LLILILMISECSVGI
-----------NLLILILMISECSVG-
----------CNLLILILMISECSV--
---------YCNLLILILMISECS---
--------CYCNLLILILMISEC----
-------RCYCNLLILILMISE-----
------KRCYCNLLILILMIS------
-----DKRCYCNLLILILMI-------
----LDKRCYCNLLILILM--------
---ILDKRCYCNLLILIL---------
MVVILDKRCYCNLLI------------
>gi|13559813|ref|NP_112026.1|/1-27
MVVILDKRCYCNLLILILMISECSVGI
```

```
13 overlapping epitopes
------------IKMIPNVSNMSQCTG
-----------VIKMIPNVSNMSQCT-
----------IVIKMIPNVSNMSQC--
```

```
---------DIVIKMIPNVSNMSQ---
--------KDIVIKMIPNVSNMS----
-------TKDIVIKMIPNVSNM-----
------LTKDIVIKMIPNVSN------
-----PLTKDIVIKMIPNVS-------
----NPLTKDIVIKMIPNV--------
---SNPLTKDIVIKMIPN---------
--KSNPLTKDIVIKMIP----------
-IKSNPLTKDIVIKMI-----------
KIKSNPLTKDIVIKM------------
>gi|13559813|ref|NP_112026.1|/47-73
KIKSNPLTKDIVIKMIPNVSNMSQCTG
```

14 overlapping epitopes

```
-------------------GVAIGIATAAQITAG
------------------AGVAIGIATAAQITA-
--------------GVIMAGVAIGIATAA-----
-------------AGVIMAGVAIGIATA------
------------LAGVIMAGVAIGIAT-------
-----------RLAGVIMAGVAIGIA--------
----------VRLAGVIMAGVAIGI---------
------LVGDVRLAGVIMAGV-------------
-----DLVGDVRLAGVIMAG--------------
----HDLVGDVRLAGVIMA---------------
---THDLVGDVRLAGVIM----------------
--NTHDLVGDVRLAGVI-----------------
-NNTHDLVGDVRLAGV------------------
KNNTHDLVGDVRLAG-------------------
>gi|13559813|ref|NP_112026.1|/98-131
KNNTHDLVGDVRLAGVIMAGVAIGIATAAQITAG
```

23 overlapping epitopes

```
---------------------------------------------------RTLGYATEDFDDLLE
--------------------------------------------------LRTLGYATEDFDDLL-
--------------------------------------------GNYETLLRTLGYATE-------
-------------------------------------------GGNYETLLRTLGYAT--------
------------------------------------------FGGNYETLLRTLGYA---------
-----------------------------------------AFGGNYETLLRTLGY----------
--------------------------------SMTIQAISQAFGGNY-------------------
-------------------------------NSMTIQAISQAFGGN--------------------
-----------------------------VSNSMTIQAISQAFG----------------------
----------------------------PVSNSMTIQAISQAF-----------------------
---------------------------DPVSNSMTIQAISQA------------------------
--------------------------QDPVSNSMTIQAISQ-------------------------
-------------------------LQDPVSNSMTIQAIS--------------------------
------------------------NLQDPVSNSMTIQAI---------------------------
-------------YLSDLLFVFGPNLQD--------------------------------------
------------KYLSDLLFVFGPNLQ---------------------------------------
-----------SKYLSDLLFVFGPNL----------------------------------------
----------LSKYLSDLLFVFGPN-----------------------------------------
---------ALSKYLSDLLFVFGP------------------------------------------
--------LALSKYLSDLLFVFG-------------------------------------------
-------DLALSKYLSDLLFVF--------------------------------------------
-QTELSLDLALSKYLS--------------------------------------------------
KQTELSLDLALSKYL---------------------------------------------------
>gi|13559813|ref|NP_112026.1|/193-258
KQTELSLDLALSKYLSDLLFVFGPNLQDPVSNSMTIQAISQAFGGNYETLLRTLGYATEDFDDLLE
```

```
29 overlapping epitopes
------------------------------------------------------------------EIGFCLITKRSVICN
-----------------------------------------------------NFILVRNTLISNIEI-------------
----------------------------------------------------PNFILVRNTLISNIE--------------
---------------------------------------------------VPNFILVRNTLISNI---------------
--------------------------------------------------IVPNFILVRNTLISN----------------
-------------------------------------------------SIVPNFILVRNTLIS-----------------
------------------------------------------------ISIVPNFILVRNTLI------------------
---------------------------------------------SEWISIVPNFILVRN---------------------
--------------------------------------------NSEWISIVPNFILVR----------------------
-------------------------------------------DNSEWISIVPNFILV-----------------------
------------------------------------------NDNSEWISIVPNFIL------------------------
-----------------------------------------NNDNSEWISIVPNFI-------------------------
-------------------------------------PVSFNNDNSEWISIV-----------------------------
------------------------------------LPVSFNNDNSEWISI------------------------------
-----------------------------------LLPVSFNNDNSEWIS-------------------------------
------------------------LTEIQQAYIQELLPV------------------------------------------
----------------IVRVYFPILTEIQQA--------------------------------------------------
---------------IIVRVYFPILTEIQQ---------------------------------------------------
-------------YYIIVRVYFPILTEI-----------------------------------------------------
--------------YIIVRVYFPILTEIQ----------------------------------------------------
------------SYYIIVRVYFPILTE------------------------------------------------------
-----------SSYYIIVRVYFPILT-------------------------------------------------------
----------LSSYYIIVRVYFPIL--------------------------------------------------------
---------DLSSYYIIVRVYFPI---------------------------------------------------------
----QIIYVDLSSYYIIVR--------------------------------------------------------------
---GQIIYVDLSSYYIIV---------------------------------------------------------------
--TGQIIYVDLSSYYII----------------------------------------------------------------
-ITGQIIYVDLSSYYI-----------------------------------------------------------------
SITGQIIYVDLSSYY------------------------------------------------------------------
>gi|13559813|ref|NP_112026.1|/261-341
SITGQIIYVDLSSYYIIVRVYFPILTEIQQAYIQELLPVSFNNDNSEWISIVPNFILVRNTLISNIEIGFCLITKRSVICN
```

```
23 overlapping epitopes
-------------------------------------EKKRNTYSRLEDRRV
-------------------------------ISFIIVEKKRNTYSR------
------------------------------FISFIIVEKKRNTYS-------
---------------------------LITFISFIIVEKKRN----------
--------------------------GLITFISFIIVEKKR-----------
-------------------------IGLITFISFIIVEKK------------
------------------------CIGLITFISFIIVEK-------------
-----------------------LCIGLITFISFIIVE--------------
----------------------SLCIGLITFISFIIV---------------
---------------------ASLCIGLITFISFII----------------
------------MIILYVLSIASLCIG-------------------------
-----------SMIILYVLSIASLCI--------------------------
----------LSMIILYVLSIASLC---------------------------
---------MLSMIILYVLSIASL----------------------------
--------SMLSMIILYVLSIAS-----------------------------
-------ISMLSMIILYVLSIA------------------------------
------LISMLSMIILYVLSI-------------------------------
-----SLISMLSMIILYVLS--------------------------------
----PSLISMLSMIILYVL---------------------------------
---NPSLISMLSMIILYV----------------------------------
--VNPSLISMLSMIILY-----------------------------------
-TVNPSLISMLSMIIL------------------------------------
```

```
DTVNPSLISMLSMII-------------------------------------
>gi|13559813|ref|NP_112026.1|/482-533
DTVNPSLISMLSMIILYVLSIASLCIGLITFISFIIVEKKRNTYSRLEDRRV
```

Glycoprotein

23 overlapping epitopes

```
------------------------------------------DALQGIQQQIKGLAD
-----------------------------------------KDALQGIQQQIKGLA-
----------------------------------------IKDALQGIQQQIKGL--
---------------------------------------VIKDALQGIQQQIKG---
--------------------------------------AVIKDALQGIQQQIK----
----------------------MNIMIIQNYTRSTDN--------------------
---------------------VMNIMIIQNYTRSTD---------------------
--------------------IVMNIMIIQNYTRST----------------------
-------------------IIVMNIMIIQNYTRS-----------------------
------------------VIIVMNIMIIQNYTR------------------------
-----------------IVIIVMNIMIIQNYT-------------------------
----------------SIVIIVMNIMIIQNY--------------------------
---------------GSIVIIVMNIMIIQN---------------------------
--------------LGSIVIIVMNIMIIQ----------------------------
-------------LLGSIVIIVMNIMII-----------------------------
------------ALLGSIVIIVMNIMI------------------------------
-----------IALLGSIVIIVMNIM-------------------------------
-----SAFNTVIALLGSIVI-------------------------------------
----LSAFNTVIALLGSIV--------------------------------------
---ILSAFNTVIALLGSI---------------------------------------
--KILSAFNTVIALLGS----------------------------------------
-SKILSAFNTVIALLG-----------------------------------------
DSKILSAFNTVIALL------------------------------------------
>gi|253559848|gb|ACT32615.1|/43-99
DSKILSAFNTVIALLGSIVIIVMNIMIIQNYTRSTDNQAMIKDALQSIQQQIKGLAD
```

9 overlapping epitopes

```
--------YSHLERIGSCSRGVS
-------AYSHLERIGSCSRGV-
------FAYSHLERIGSCSRG--
-----YFAYSHLERIGSCSR---
----GYFAYSHLERIGSCS----
---EGYFAYSHLERIGSC-----
--DEGYFAYSHLERIGS------
-MDEGYFAYSHLERIG-------
AMDEGYFAYSHLERI--------
>gi|253559848|gb|ACT32615.1|/223-245
AMDEGYFAYSHLEKIGSCSRGVS
```

13 overlapping epitopes

```
------------FLIDRINWISAGVFL
-----------AFLIDRINWISAGVF-
----------DAFLIDRINWISAGV--
---------NDAFLIDRINWISAG---
--------YNDAFLIDRINWISA----
-------VYNDAFLIDRINWIS-----
------GVYNDAFLIDRINWI------
-----EGVYNDAFLIDRINW-------
```

```
----WEGVYNDAFLIDRIN--------
---CWEGVYNDAFLIDRI---------
--ICWEGVYNDAFLIDR----------
-EICWEGVYNDAFLID-----------
PEICWEGVYNDAFLI------------
>gi|253559848|gb|ACT32615.1|/500-526
PEVCWEGVYNDAFLIDRINWISAGVFL
```

Matrix Protein

15 overlapping epitopes

```
-------------------------------EFRRNNAIAFNLLVY
------------------------------LEFRRNNAIAFNLLV-
-----------------------------MLEFRRNNAIAFNLL--
----------------------------TMLEFRRNNAIAFNL---
---------------------------RTMLEFRRNNAIAFN----
--------------------------PRTMLEFRRNNAIAF-----
-------------------DSGIYMIPRTMLEFR------------
-------LRIFFLSITKLNDSG------------------------
------ALRIFFLSITKLNDS-------------------------
-----QALRIFFLSITKLND--------------------------
----HQALRIFFLSITKLN---------------------------
---KHQALRIFFLSITKL----------------------------
--DKHQALRIFFLSITK-----------------------------
-LDKHQALRIFFLSIT------------------------------
QLDKHQALRIFFLSI-------------------------------
>gi|13559811|ref|NP_112025.1|/164-209
QLDKHQALRIFFLSITKLNDSGIYMIPRTMLEFRRNNAIAFNLLVY
```

10 overlapping epitopes

```
---------YDDVFIDNTGRILKG
--------IYDDVFIDNTGRILK-
-------MIYDDVFIDNTGRIL--
------FMIYDDVFIDNTGRI---
-----EFMIYDDVFIDNTGR----
----REFMIYDDVFIDNTG-----
---PREFMIYDDVFIDNT------
--IPREFMIYDDVFIDN-------
-SIPREFMIYDDVFID--------
PSIPREFMIYDDVFI---------
>gi|13559811|ref|NP_112025.1|/329-352
PSIPREFMIYDDVFIDNTGRILKG
```

Nucleoprotein

15 overlapping epitopes

```
----------------------TLFALDVIRSPSAAE
---------------------LTLFALDVIRSPSAA-
--------------------ELTLFALDVIRSPSA--
-------------------WELTLFALDVIRSPS---
------------------RWELTLFALDVIRSP----
-----------------LRWELTLFALDVIRS-----
----------------ELRWELTLFALDVIR------
---------------PELRWELTLFALDVI-------
```

```
------RIFVPATNSPELRWE----------------
-----IRIFVPATNSPELRW-----------------
----KIRIFVPATNSPELR------------------
---TKIRIFVPATNSPEL-------------------
--TTKIRIFVPATNSPE--------------------
-LTTKIRIFVPATNSP---------------------
TLTTKIRIFVPATNS----------------------
>gi|1679387250|gb|QCY59029.1|/30-66
TLTTKIRIFVPATNSPELRWELTLFALDVIRSPSAAE
```

14 overlapping epitopes

```
------------------VIIDVGSMVNGIPVM
-----------------AVIIDVGSMVNGIPV-
----------------EAVIIDVGSMVNGIP--
---------------IEAVIIDVGSMVNGI---
--------------DIEAVIIDVGSMVNG----
-------------PDIEAVIIDVGSMVN-----
------------DPDIEAVIIDVGSMV------
------IRSLLNDPDIEAVII------------
-----LIRSLLNDPDIEAVI-------------
----ALIRSLLNDPDIEAV--------------
---GALIRSLLNDPDIEA---------------
--PGALIRSLLNDPDIE----------------
-RPGALIRSLLNDPDI-----------------
ERPGALIRSLLNDPD------------------
>gi|1679387250|gb|QCY59029.1|/82-114
ERPGALIRSLLNDPDIEAVIIDVGSMVNGIPVM
```

11 overlapping epitopes

```
----------------------LVSAVITIEAQIWIL
---------------------TLVSAVITIEAQIWI-
--------------------STLVSAVITIEAQIW--
-------------------MSTLVSAVITIEAQI---
------------------DMSTLVSAVITIEAQ----
-----------------TDMSTLVSAVITIEA-----
----------------ITDMSTLVSAVITIE------
---------------RITDMSTLVSAVITI-------
--------------LRITDMSTLVSAVIT--------
-------------GLRITDMSTLVSAVI---------
SKGKTPFVDSRAYGL----------------------
>gi|1679387250|gb|QCY59029.1|/139-175
SKGKTPFVDSRAYGLRITDMSTLVSAVITIEAQIWIL
```

14 overlapping epitopes

```
----------------------AELAAAVQETSAGRQ
---------------------VAELAAAVQETSAGR-
--------------------QVAELAAAVQETSAG--
-------------------DQVAELAAAVQETSA---
------------------SDQVAELAAAVQETS----
-----------------SSDQVAELAAAVQET-----
----------------LSSDQVAELAAAVQE------
---------------GLSSDQVAELAAAVQ-------
--------------LGLSSDQVAELAAAV--------
----GGIDQNMANRLGLSS------------------
---AGGIDQNMANRLGLS-------------------
--HAGGIDQNMANRLGL--------------------
```

```
-HHAGGIDQNMANRLG---------------------
RHHAGGIDQNMANRL----------------------
>gi|1679387250|gb|QCY59029.1|/368-404
RHHAGGIDQNMANRLGLSSNQVAELAAAVQETSAGRQ
```

Phosphoprotein

12 overlapping epitopes

```
-----------VVTDVVYHDHGGECT
----------PVVTDVVYHDHGGEC-
---------DPVVTDVVYHDHGGE--
--------LDPVVTDVVYHDHGG---
-------QLDPVVTDVVYHDHG----
------IQLDPVVTDVVYHDH-----
-----DIQLDPVVTDVVYHD------
----DDIQLDPVVTDVVYH-------
---SDDIQLDPVVTDVVY--------
--GSDDIQLDPVVTDVV---------
-EGSDDIQLDPVVTDV----------
AEGSDDIQLDPVVTD-----------
>gi|1802790259|gb|QHR78950.1|/99-124
AEGSDDIQLDPVVTDVVYHDHGGECT
```

11 overlapping epitopes

```
---------------------NEPYGAAVQLREDLI
--------------------TNEPYGAAVQLREDL-
-------------------LTNEPYGAAVQLRED--
------------------SLTNEPYGAAVQLRE---
------FSFDNVKNFRDGSLT---------------
-----LFSFDNVKNFRDGSL----------------
----SLFSFDNVKNFRDGS-----------------
---QSLFSFDNVKNFRDG------------------
--QQSLFSFDNVKNFRD-------------------
-EQQSLFSFDNVKNFR--------------------
LEQQSLFSFDNVKNF---------------------
>gi|1802790259|gb|QHR78950.1|/603-638
LEQQSLFSFDNVKNFRDGSLTNEPYGAAVQLREDLI
```

Polymerase

12 overlapping epitopes

```
--------------------IHLLAEFFSFFRTFG
-------------------NIHLLAEFFSFFRTF-
------------------DNIHLLAEFFSFFRT--
-----------------IDNIHLLAEFFSFFR---
----------------NIDNIHLLAEFFSFF----
------MFIDDLLSILNIDNI--------------
-----SMFIDDLLSILNIDN---------------
----RSMFIDDLLSILNID----------------
---IRSMFIDDLLSILNI-----------------
--KIRSMFIDDLLSILN------------------
-QKIRSMFIDDLLSIL-------------------
DQKIRSMFIDDLLSI--------------------
>gi|15487370|gb|AAK50546.1|/330-364
```

DQKIRSMFIDDLLSILNIDNIHLLAEFFSFFRTFG

11 overlapping epitopes
----------------------ETLTLDVTSPISNNL
---------------------NETLTLDVTSPISNN-
--------------------INETLTLDVTSPISN--
-------------------SINETLTLDVTSPIS---
------------------FSINETLTLDVTSPI----
-----ILKVIQQLLISTEFS-----------------
----NILKVIQQLLISTEF------------------
---INILKVIQQLLISTE-------------------
--CINILKVIQQLLIST--------------------
-YCINILKVIQQLLIS---------------------
GYCINILKVIQQLLI----------------------
>gi|15487370|gb|AAK50546.1|/947-983
GYCINILKVIQQLLISTEFSINETLTLDVTSPISNNL

7 overlapping epitopes
------FLILNKLLSNRRQND
-----QFLILNKLLSNRRQN-
----NQFLILNKLLSNRRQ--
---YNQFLILNKLLSNRR---
--DYNQFLILNKLLSNR----
-HDYNQFLILNKLLSN-----
HHDYNQFLILNKLLS------
>gi|15487370|gb|AAK50546.1|/1164-1184
HHDYNQFLILNKLLSNRRQND

7 overlapping epitopes
------YQIDQPFFVPTKITS
-----YYQIDQPFFVPTKIT-
----KYYQIDQPFFVPTKI--
---KKYYQIDQPFFVPTK---
--LKKYYQIDQPFFVPT----
-DLKKYYQIDQPFFVP-----
DDLKKYYQIDQPFFV------
>gi|15487370|gb|AAK50546.1|/2064-2084
DDLKKYYQIDQPFFVPTKITS

V Protein

12 overlapping epitopes
-----------VVTDVVYHDHGGECT
----------PVVTDVVYHDHGGEC-
---------DPVVTDVVYHDHGGE--
--------LDPVVTDVVYHDHGG---
-------QLDPVVTDVVYHDHG----
------IQLDPVVTDVVYHDH-----
-----DIQLDPVVTDVVYHD------
----DDIQLDPVVTDVVYH-------
---SDDIQLDPVVTDVVY--------
--GSDDIQLDPVVTDVV---------
-EGSDDIQLDPVVTDV----------
AEGSDDIQLDPVVTD-----------

```
>gi|1802790260|gb|QHR78951.1|/99-124
AEGSDDIQLDPVVTDVVYHDHGGECT


12 overlapping epitopes
-----------KMLSYAPEIAVSKED
----------AKMLSYAPEIAVSKE-
---------DAKMLSYAPEIAVSK--
--------SDAKMLSYAPEIAVS---
-------VSDAKMLSYAPEIAV----
------LVSDAKMLSYAPEIA-----
-----CLVSDAKMLSYAPEI------
----VCLVSDAKMLSYAPE-------
---NVCLVSDAKMLSYAP--------
--GNVCLVSDAKMLSYA---------
-NGNVCLVSDAKMLSY----------
NNGNVCLVSDAKMLS-----------
>gi|1802790260|gb|QHR78951.1|/144-169
NNGNVCLVSDAKMLSYAPEIAVSKED


20 overlapping epitopes
-----------------------------------SEVIVGISPEDEEPS
----------------------------------SSEVIVGISPEDEEP-
---------------------------------SSSEVIVGISPEDEE--
--------------------------------GSSSEVIVGISPEDE---
-------------------------EFEDEFAGSSSEVIV----------
------------------------LEFEDEFAGSSSEVI-----------
--------------YTSDDEEADQLEFED---------------------
-------------LYTSDDEEADQLEFE----------------------
------------KLYTSDDEEADQLEF-----------------------
-----------IKLYTSDDEEADQLE------------------------
----------SIKLYTSDDEEADQL-------------------------
---------DSIKLYTSDDEEADQ--------------------------
--------LDSIKLYTSDDEEAD---------------------------
------VNLDSIKLYTSDDEE-----------------------------
-----NVNLDSIKLYTSDDE------------------------------
----RNVNLDSIKLYTSDD-------------------------------
---SRNVNLDSIKLYTSD--------------------------------
--TSRNVNLDSIKLYTS---------------------------------
-QTSRNVNLDSIKLYT----------------------------------
PQTSRNVNLDSIKLY-----------------------------------
>gi|1802790260|gb|QHR78951.1|/224-273
PQTSRNVNLDSIKLYTSDDEEADQLEFEDEFAGSSSEVIVGISPEDEEPS


W Protein


12 overlapping epitopes
-----------VVTDVVYHDHGGECT
----------PVVTDVVYHDHGGEC-
---------DPVVTDVVYHDHGGE--
--------LDPVVTDVVYHDHGG---
-------QLDPVVTDVVYHDHG----
------IQLDPVVTDVVYHDH-----
-----DIQLDPVVTDVVYHD------
----DDIQLDPVVTDVVYH-------
---SDDIQLDPVVTDVVY--------
```

```
--GSDDIQLDPVVTDVV---------
-EGSDDIQLDPVVTDV----------
AEGSDDIQLDPVVTD-----------
>gi|374256971|gb|AEZ01401.1|/99-124
AEGSDDIQLDPVVTDVVYHDHGGECT
```

```
11 overlapping epitopes
-----------KMLSYAPEIAVSKED
----------AKMLSYAPEIAVSKE
---------DAKMLSYAPEIAVSK-
--------SDAKMLSYAPEIAVS--
-------VSDAKMLSYAPEIAV---
------LVSDAKMLSYAPEIA----
-----CLVSDAKMLSYAPEI-----
----VCLVSDAKMLSYAPE------
---NVCLVSDAKMLSYAP-------
--GNVCLVSDAKMLSYA--------
-NGNVCLVSDAKMLSY---------
NNGNVCLVSDAKMLS----------
>gi|374256971|gb|AEZ01401.1|/144-169
NNGDVCLVSDAKVLSYAPEIAVSKED
```

SUPPLEMENTARY txt 3: Overlapping epitopes and HLA allele pairs:

```
AAFTLISMY       HLA-A*30:02
AAFTLISMY       HLA-B*15:01
AAFTLISMY       HLA-B*35:01
AAFTLISMY       HLA-A*30:02
AAFTLISMY       HLA-B*35:01
AATATLTTK       HLA-A*11:01
AAYPLGVGK       HLA-A*11:01
AAYPLGVGK       HLA-A*03:01
AAYPLGVGK       HLA-A*30:01
AEESETRRW       HLA-B*44:02
AEESETRRW       HLA-B*44:03
AEFFSFFRTF      HLA-B*44:03
AEFFSFFRTF      HLA-B*44:02
AEFFSFFRTF      HLA-A*23:01
AEFFSFFRTF      HLA-B*40:01
AEFFSFFRTF      HLA-A*24:02
AEFFSFFRTF      HLA-B*44:03
AEFFSFFRTF      HLA-B*44:02
AEGSDDIQL       HLA-B*40:01
AEGSDDIQL       HLA-B*40:01
AEGSDDIQL       HLA-B*40:01
AELPIPEY        HLA-B*44:03
AELPIPEY        HLA-B*44:02
AENDEEIQEI      HLA-B*44:02
AENPVFTVF       HLA-B*44:03
AENPVFTVF       HLA-B*44:02
AENPVFTVF       HLA-B*40:01
AENPVFTVF       HLA-B*44:03
AENPVFTVF       HLA-B*44:02
AENPVFTVF       HLA-B*40:01
```

AEQDETVISL	HLA-B*40:01
AESMKVGAAF	HLA-B*44:02
AESMKVGAAF	HLA-B*44:03
AESMKVGAAF	HLA-B*15:01
AESMKVGAAF	HLA-B*44:02
AESMKVGAAF	HLA-B*44:03
AGRLFAKMTYK	HLA-A*03:01
AIGPPVFTDK	HLA-A*11:01
AIGPPVFTDK	HLA-A*03:01
AIRIATVYTW	HLA-B*58:01
AISQAFGGNY	HLA-A*30:02
ALIASGVGK	HLA-A*03:01
ALIASGVGKY	HLA-B*15:01
ALIASGVGKY	HLA-B*15:01
ALIPAPIGGF	HLA-B*15:01
ALKSMSRCCF	HLA-B*15:01
ALQSIQQQI	HLA-A*02:03
ALQSIQQQI	HLA-A*02:01
ALSHPRVFK	HLA-A*03:01
ALSHPRVFK	HLA-A*11:01
ALSHPRVFK	HLA-A*30:01
ALSHPRVFKR	HLA-A*31:01
ALSPIKDEW	HLA-A*32:01
ALSPIKDEW	HLA-B*58:01
ALWGLIDPL	HLA-A*02:01
ALWGLIDPL	HLA-A*02:06
ALYEAMKNA	HLA-A*02:03
ALYEAMKNA	HLA-A*02:01
ALYEAMKNA	HLA-A*02:06
ALYLGQSISIK	HLA-A*03:01
AMDEGYFAY	HLA-A*01:01
AMDEGYFAY	HLA-A*01:01
AMDEGYFAY	HLA-A*30:02
AMIKDALQSI	HLA-A*02:03
APGGYPLLW	HLA-B*53:01
APIGGFNYL	HLA-B*07:02
APIGGFNYL	HLA-B*07:02
APIMKAHAI	HLA-B*08:01
APIMKAHAI	HLA-B*07:02
APIMKAHAIF	HLA-B*07:02
APIMKAHAIF	HLA-B*15:01
APVENLKKL	HLA-B*07:02
AQITAGVAL	HLA-B*15:01
AQITAGVALY	HLA-B*15:01
AQITAGVALY	HLA-B*15:01
AQITAGVALY	HLA-A*30:02
AQKTITNCF	HLA-B*15:01
AQPPYHWSI	HLA-A*02:06
AQPPYHWSI	HLA-A*02:06
ASDKGKNPSK	HLA-A*30:01
ASFRSYQSK	HLA-A*30:01
ASFRSYQSK	HLA-A*11:01
ASFRSYQSK	HLA-A*03:01
ASFSWDTMIK	HLA-A*11:01
ASILLTLFR	HLA-A*11:01
ASILLTLFR	HLA-A*68:01
ASILLTLFR	HLA-A*31:01
ASILLTLFR	HLA-A*31:01
ASILLTLFR	HLA-A*11:01

ASINENVNEK	HLA-A*11:01
ASKHIIRLK	HLA-A*30:01
ATDDPAISNK	HLA-A*11:01
ATIPFLFLSAY	HLA-A*11:01
ATIPFLFLSAY	HLA-A*30:02
ATNSPELRW	HLA-B*58:01
ATNSPELRW	HLA-B*57:01
ATNSPELRW	HLA-B*58:01
ATNSPELRW	HLA-A*32:01
AVKEEPPQK	HLA-A*30:01
AVKEEPPQK	HLA-A*11:01
AVKEEPPQK	HLA-A*03:01
AVKEEPPQK	HLA-A*30:01
AVKEEPPQK	HLA-A*11:01
AVKEEPPQK	HLA-A*03:01
AVKEKSPQK	HLA-A*30:01
AVKEKSPQK	HLA-A*03:01
AVKEKSPQK	HLA-A*11:01
AVKPKNNGESY	HLA-B*15:01
AVKPKNNGESY	HLA-A*30:02
AVLQPSIPR	HLA-A*11:01
AVLQPSIPR	HLA-A*31:01
AVYNNEFYY	HLA-A*11:01
AVYNNEFYY	HLA-A*30:02
AVYNNEFYY	HLA-A*30:02
AVYNNEFYY	HLA-A*11:01
AYIQELLPVSF	HLA-A*23:01
AYIQELLPVSF	HLA-A*24:02
AYPECNNILF	HLA-A*24:02
AYPECNNILF	HLA-A*23:01
AYPECNNILF	HLA-A*24:02
AYPECNNILF	HLA-A*23:01
CLVSDAKMLSY	HLA-A*01:01
CLVSDAKMLSY	HLA-A*01:01
CLVSDAKMLSY	HLA-A*01:01
CLVSDAKMLSY	HLA-A*01:01
CLVSDAKMLSY	HLA-A*01:01
CLVSDAKVLSY	HLA-A*01:01
CPMLPKLQF	HLA-B*53:01
CPMLPKLQF	HLA-B*35:01
CPMLPKLQF	HLA-B*07:02
CPNPLPFREY	HLA-B*53:01
CSVDLARALR	HLA-A*68:01
DAELPIPEY	HLA-B*35:01
DAFLIDRI	HLA-B*51:01
DALEAMVGR	HLA-A*68:01
DALEAMVGR	HLA-A*33:01
DAQPPYHW	HLA-B*53:01
DAQPPYHW	HLA-B*53:01
DAQPPYHWSI	HLA-B*51:01
DAQPPYHWSI	HLA-B*51:01
DEHELLKTL	HLA-B*44:03
DEHELLKTLF	HLA-B*44:03
DENGSMIPKY	HLA-B*44:03
DENGSMIPKY	HLA-B*44:02
DGLLVSKI	HLA-B*51:01
DIFEEAASF	HLA-B*35:01
DIFEEAASF	HLA-A*26:01
DIFEEAASFR	HLA-A*68:01

DIGSDINTLR	HLA-A*68:01
DIKLGNVKR	HLA-A*33:01
DIKYQPLISR	HLA-A*33:01
DIKYQPLISR	HLA-A*68:01
DILEQQSLF	HLA-A*26:01
DISSQISSM	HLA-A*26:01
DLKKYYQI	HLA-B*08:01
DPAKDSPVI	HLA-B*51:01
DPAKDSPVI	HLA-B*51:01
DPVLNSTYW	HLA-B*53:01
DPVSNSMTI	HLA-B*51:01
DPVVTDVVY	HLA-B*35:01
DPVVTDVVY	HLA-B*35:01
DPVVTDVVY	HLA-B*35:01
DPVVTDVVY	HLA-B*35:01
DPVVTDVVY	HLA-B*35:01
DPVVTDVVY	HLA-B*35:01
DPYNMLEYV	HLA-B*51:01
DSITGQIIY	HLA-A*26:01
DSITGQIIY	HLA-B*35:01
DSITGQIIYV	HLA-A*68:02
DSLGQPVFY	HLA-A*26:01
DSQSITKTI	HLA-B*51:01
DSYEYIINR	HLA-A*68:01
DSYEYIINR	HLA-A*68:01
DSYEYIINR	HLA-A*33:01
DTAEESETR	HLA-A*68:01
DTAEESETRR	HLA-A*68:01
DTVNPSLISM	HLA-A*26:01
DVFIDNTGR	HLA-A*68:01
DVFIDNTGR	HLA-A*33:01
DVLKAITPV	HLA-A*68:02
DVQTVNPLV	HLA-A*68:02
DYATPMTNNMR	HLA-A*33:01
EALIASGVGKY	HLA-A*26:01
EAMVGRYI	HLA-B*51:01
EAWYLASQR	HLA-A*68:01
EAWYLASQR	HLA-A*68:01
EAWYLASQR	HLA-A*33:01
EEAASFRSY	HLA-B*44:03
EEAASFRSY	HLA-B*44:02
EEAASFRSY	HLA-B*44:03
EEAASFRSY	HLA-B*44:02
EEMEGLMRI	HLA-B*44:03
EEMEGLMRI	HLA-B*44:02
EEMEGLMRIL	HLA-B*44:03
EENSKIVFI	HLA-B*44:02
EENSKIVFI	HLA-B*44:03
EESETRRW	HLA-B*44:02
EESETRRW	HLA-B*44:03
EESETRRWAKY	HLA-B*44:02
EESETRRWAKY	HLA-B*44:03
EFFSFFRTF	HLA-A*23:01
EFFSFFRTF	HLA-A*24:02
EFFSFFRTF	HLA-A*23:01
EFFSFFRTF	HLA-A*24:02
EFNPHNHYK	HLA-A*33:01
EICAKQAQLY	HLA-A*26:01
EIGPKVSL	HLA-B*08:01

EIINIHECR	HLA-A*68:01
EIINIHECR	HLA-A*33:01
EIINIHECR	HLA-A*68:01
EIINIHECR	HLA-A*33:01
EIISDIGNY	HLA-A*26:01
EIISDIGNY	HLA-A*26:01
EIISDIGNYV	HLA-A*68:02
EILKSEISY	HLA-B*35:01
EIPKIINKL	HLA-A*68:02
EIPKIINKL	HLA-A*26:01
EIQEIANTV	HLA-A*68:02
EISDQRLSI	HLA-A*68:02
EIYDTGDNV	HLA-A*68:02
EIYDTGDNVIR	HLA-A*68:01
EIYGLPGFF	HLA-A*26:01
EIYGLPGFF	HLA-A*26:01
ELKLDSDLSMY	HLA-A*01:01
ELKLFPSEY	HLA-A*26:01
ELRSELIGY	HLA-A*26:01
ELRSELIGY	HLA-A*26:01
ELVVSSHVPR	HLA-A*68:01
EPYGAAVQL	HLA-B*51:01
EPYGAAVQL	HLA-B*35:01
EPYGAAVQL	HLA-B*53:01
EQTMGMYVLY	HLA-A*26:01
EQTMGMYVLY	HLA-A*68:01
EQTMGMYVLY	HLA-A*26:01
ESDSITGQIIY	HLA-A*01:01
ESIDRVLAK	HLA-A*68:01
ESMAIFAER	HLA-A*68:01
ESMAIFAER	HLA-A*33:01
ESMAIFAER	HLA-A*68:01
ESMAIFAER	HLA-A*33:01
ESMAIFAERL	HLA-A*68:02
ESMEGVSDF	HLA-A*26:01
ESMKVGAAF	HLA-B*35:01
ESMKVGAAF	HLA-A*26:01
ESMKVGAAF	HLA-A*26:01
ESTNEAVVKL	HLA-A*68:02
ETAEKTVY	HLA-A*26:01
ETAEKTVYV	HLA-A*68:02
ETAEKTVYV	HLA-A*26:01
ETAEKTVYVL	HLA-A*68:02
ETDDYNGIY	HLA-A*01:01
ETDDYNGIY	HLA-A*01:01
ETDDYNGIY	HLA-A*26:01
ETDDYNGIYHL	HLA-A*68:02
ETIISTHLF	HLA-A*26:01
ETIISTHLF	HLA-A*26:01
ETIISTHLFIY	HLA-A*26:01
ETKQAGRLF	HLA-A*26:01
ETLLRTLGY	HLA-A*26:01
ETLLRTLGY	HLA-A*26:01
ETNTRIAAI	HLA-A*68:02
ETTVKSDIKY	HLA-A*26:01
ETTWIGNLDSY	HLA-A*26:01
ETTWIGNLDSY	HLA-A*26:01
EVDNNHLIY	HLA-A*01:01
EVDNNHLIY	HLA-B*35:01

EVDNNHLIY	HLA-A*01:01
EVDNNHLIY	HLA-A*26:01
EVIDTTTML	HLA-A*68:02
EVIDTTTML	HLA-A*26:01
EVIDTTTMLR	HLA-A*68:01
EVLDRGDEV	HLA-A*68:02
EVNDQGITSV	HLA-A*68:02
EVPDALEAM	HLA-A*26:01
EVPDALEAMV	HLA-A*68:02
EVPSLFMTNV	HLA-A*68:02
EWISIVPNF	HLA-A*23:01
EWISIVPNF	HLA-A*24:02
EYIINRTAGR	HLA-A*33:01
EYIINRTAGR	HLA-A*33:01
FAERLDEIY	HLA-B*35:01
FAERLDEIY	HLA-A*01:01
FALSNGVLF	HLA-B*35:01
FALSNGVLF	HLA-B*53:01
FALSNGVLF	HLA-B*35:01
FALSNGVLF	HLA-B*53:01
FALYLGQSI	HLA-B*51:01
FAMGVATTI	HLA-B*51:01
FAPGGYPLLW	HLA-B*53:01
FAPGGYPLLW	HLA-B*58:01
FAPGGYPLLW	HLA-B*53:01
FAYSHLEKI	HLA-B*51:01
FAYSHLEKI	HLA-A*02:06
FEKLSQNLL	HLA-B*40:01
FIDDLLSIL	HLA-A*02:06
FIDDLLSIL	HLA-A*02:01
FIDDLLSIL	HLA-A*02:06
FIDDLLSIL	HLA-A*02:01
FILVRNTL	HLA-B*08:01
FIYSKKIHY	HLA-B*15:01
FIYSKKIHY	HLA-B*15:01
FIYSKKIHY	HLA-A*26:01
FIYSKKIHY	HLA-A*30:02
FLDWASDPY	HLA-B*35:01
FLDWASDPY	HLA-A*01:01
FLIDRINWI	HLA-A*02:03
FLIDRINWI	HLA-A*02:01
FLIDRINWI	HLA-A*02:06
FLIDRINWI	HLA-A*02:03
FLIDRINWI	HLA-A*02:01
FLIDRINWI	HLA-A*02:06
FLIDRINWISA	HLA-A*02:03
FLIDRINWISA	HLA-A*02:01
FLIVDPELFAL	HLA-A*02:01
FLIVDPELFAL	HLA-A*02:06
FLKKIGKL	HLA-B*08:01
FLMDRRVIL	HLA-B*08:01
FLMDRRVIL	HLA-A*02:03
FLMDRRVIL	HLA-A*02:01
FLMDRRVIL	HLA-B*08:01
FLMDRRVIL	HLA-A*02:03
FLMDRRVIL	HLA-A*02:01
FMVEILIEV	HLA-A*02:06
FMVEILIEV	HLA-A*02:01
FMVEILIEV	HLA-A*02:03

FMVEILIEV	HLA-A*02:06
FMVEILIEV	HLA-A*02:01
FMVEILIEV	HLA-A*02:03
FNKVKSAL	HLA-B*08:01
FNYLNLSRIF	HLA-A*23:01
FPAVGFLV	HLA-B*51:01
FPHDTDRLNY	HLA-B*35:01
FPHDTDRLNY	HLA-B*53:01
FPHDTDRLNY	HLA-B*35:01
FPHDTDRLNY	HLA-B*53:01
FPILTEIQQAY	HLA-B*35:01
FPILTEIQQAY	HLA-B*35:01
FPILTEIQQAY	HLA-B*53:01
FPISRLFNM	HLA-B*35:01
FPISRLFNM	HLA-B*51:01
FPISRLFNM	HLA-B*08:01
FPISRLFNM	HLA-B*53:01
FPISRLFNMY	HLA-B*35:01
FPISRLFNMY	HLA-B*53:01
FPISRLFNMY	HLA-B*35:01
FPLWSTEEL	HLA-B*35:01
FPLWSTEEL	HLA-B*53:01
FPLWSTEEL	HLA-B*35:01
FPLWSTEEL	HLA-B*53:01
FPLWSTEEL	HLA-B*51:01
FPVMGNRI	HLA-B*51:01
FPVMGNRIY	HLA-B*35:01
FPVMGNRIY	HLA-B*53:01
FPVMGNRIY	HLA-B*35:01
FPVMGNRIY	HLA-B*53:01
FSFDNVKNF	HLA-B*35:01
FSFDNVKNF	HLA-B*15:01
FSFDNVKNF	HLA-A*26:01
FSFDNVKNFR	HLA-A*68:01
FSFDNVKNFR	HLA-A*31:01
FSFDNVKNFR	HLA-A*68:01
FSLMDINPW	HLA-B*58:01
FSLMDINPWL	HLA-A*02:01
FSLMDINPWL	HLA-A*02:06
FSWDTMIKF	HLA-B*58:01
FTDQKIRSM	HLA-B*08:01
FTLISMYSER	HLA-A*68:01
FTSSPERGW	HLA-B*58:01
FTSSPERGW	HLA-B*57:01
FTSSPERGW	HLA-B*58:01
FTSSPERGW	HLA-B*57:01
FTSSPERGW	HLA-B*58:01
FTSSPERGW	HLA-B*57:01
FVDSRAYGL	HLA-A*02:06
FVIFYASLTY	HLA-B*15:01
FVIFYASLTY	HLA-B*35:01
FVNDENFDPY	HLA-B*35:01
FVNDENFDPY	HLA-A*01:01
FYASLTYLR	HLA-A*33:01
FYASLTYLR	HLA-A*33:01
FYASLTYLRR	HLA-A*33:01
GEGSGSMML	HLA-B*40:01
GEKVDTNLIY	HLA-B*44:03
GESYNQHQF	HLA-B*44:02

GESYNQHQF	HLA-B*44:03
GESYNQHQF	HLA-B*40:01
GFFNWMHKR	HLA-A*31:01
GFFNWMHKR	HLA-A*33:01
GIEGYSQKTW	HLA-B*44:02
GIKQGDTLY	HLA-B*15:01
GIQFDCFMEL	HLA-A*02:06
GLADKIGTEI	HLA-A*02:03
GLIDPLFPV	HLA-A*02:06
GLIDPLFPV	HLA-A*02:01
GLIDPLFPV	HLA-A*02:03
GLIDPLFPV	HLA-A*02:01
GLIDPLFPV	HLA-A*02:06
GLIDPLFPV	HLA-A*02:03
GLIRSGLRK	HLA-A*03:01
GLLDSKILSA	HLA-A*02:01
GLLDSKILSA	HLA-A*02:03
GLLDTTKGL	HLA-A*02:01
GLLDTTKGL	HLA-A*02:03
GLMRILKTA	HLA-A*02:03
GLNPTAIPF	HLA-B*15:01
GLNPTAVPF	HLA-B*15:01
GLNPTAVPF	HLA-B*15:01
GLNPTAVPF	HLA-B*15:01
GLNPTAVPF	HLA-B*15:01
GLNPTAVPF	HLA-B*15:01
GLQPKLVSR	HLA-A*31:01
GLSSDQVAEL	HLA-A*02:03
GLVKGVTRK	HLA-A*03:01
GMAGFFATI	HLA-A*02:03
GMAGFFATI	HLA-A*02:03
GMFEDHPPTK	HLA-A*03:01
GMFEDHPPTKK	HLA-A*03:01
GPLNHLVPW	HLA-B*53:01
GPQTSRNVNL	HLA-B*07:02
GPQTSRNVNL	HLA-B*07:02
GPRAPYMVL	HLA-B*07:02
GPRAPYMVL	HLA-B*07:02
GPRAPYMVLL	HLA-B*07:02
GQIIYVDLSSY	HLA-B*15:01
GQKVQMTY	HLA-B*15:01
GQSISIKWAF	HLA-B*15:01
GRLFAKMTYK	HLA-A*03:01
GSEDPIIQEL	HLA-B*40:01
GSEDPIIREL	HLA-B*40:01
GSEDPIIREL	HLA-B*40:01
GSGSMMLLY	HLA-A*30:02
GSGSMMLLY	HLA-A*30:02
GTEIGPKVSL	HLA-B*40:01
GTIGKRVSNTR	HLA-A*31:01
GTIGKRVSNTR	HLA-A*31:01
GTIGKRVSNTR	HLA-A*31:01
GTKFDTVSAF	HLA-B*15:01
GYLEPMYFR	HLA-A*31:01
GYLEPMYFR	HLA-A*33:01
GYLEPMYFRL	HLA-A*23:01
GYPLLWSF	HLA-A*23:01
GYPLLWSF	HLA-A*24:02
GYPLLWSF	HLA-A*23:01

GYSQKTWTI	HLA-A*24:02
GYSQKTWTI	HLA-A*23:01
HIIEGQSTR	HLA-A*68:01
HIKNNIRRK	HLA-A*30:01
HLDSPIVSGK	HLA-A*03:01
HLLAEFFSF	HLA-A*23:01
HLLAEFFSF	HLA-A*32:01
HLLAEFFSF	HLA-B*15:01
HLLAEFFSF	HLA-A*32:01
HLLAEFFSFF	HLA-B*15:01
HLLGRKTCL	HLA-B*08:01
HMLADKVLEY	HLA-B*15:01
HMLADKVLEY	HLA-A*30:02
HMLADKVLEY	HLA-B*15:01
HMRDKSTEI	HLA-B*08:01
HPNLPYKV	HLA-B*51:01
HPPTKKARVSM	HLA-B*07:02
HPQDLLEEL	HLA-B*35:01
HPQDLLEEL	HLA-B*53:01
HPQDLLEEL	HLA-B*07:02
HQEQTMGMY	HLA-A*30:02
HTEFNPHNHY	HLA-A*30:02
HTEFNPHNHY	HLA-A*01:01
HTEFNPHNHY	HLA-B*44:03
HTEFNPHNHY	HLA-A*30:02
HTEFNPHNHY	HLA-B*44:02
HTEFNPHNHY	HLA-A*26:01
HTYKVLSNA	HLA-A*68:02
HVDTSSRSW	HLA-B*58:01
HVDTSSRSW	HLA-B*53:01
IATVYTWAY	HLA-B*35:01
IATVYTWAY	HLA-B*58:01
IATVYTWAY	HLA-B*53:01
IATVYTWAY	HLA-A*30:02
IATVYTWAY	HLA-B*35:01
IAYPECNNI	HLA-B*51:01
IEGYSQKTW	HLA-B*44:02
IEGYSQKTW	HLA-B*44:03
IFNAVKVCR	HLA-A*33:01
IFNAVKVCR	HLA-A*31:01
IFYASLTYLR	HLA-A*31:01
IFYASLTYLR	HLA-A*33:01
IFYASLTYLRR	HLA-A*31:01
IGPRAPYMVL	HLA-B*07:02
IGTEIGPKVSL	HLA-B*40:01
IHLLAEFFSF	HLA-A*23:01
IIMLNEAMNY	HLA-B*15:01
IIMLNEAMNY	HLA-A*30:02
IISDIGNYV	HLA-A*02:03
IISDIGNYV	HLA-A*02:06
IISDIGNYV	HLA-A*68:02
IISTHLFIY	HLA-A*30:02
IIYVDLSSY	HLA-B*15:01
IIYVDLSSY	HLA-A*30:02
IIYVDLSSY	HLA-B*15:01
IIYVDLSSY	HLA-A*30:02
IIYVDLSSYY	HLA-A*30:02
IIYVDLSSYY	HLA-A*30:02
ILIAKAVTA	HLA-A*02:03

ILKPKLISY	HLA-B*15:01
ILKPKLISY	HLA-B*15:01
ILKPKLISY	HLA-A*30:02
ILKPKLISY	HLA-A*32:01
ILKPKLISYTL	HLA-B*08:01
ILNIDNIHLL	HLA-A*02:03
ILNIDNIHLL	HLA-A*02:01
ILNIDNIHLL	HLA-A*02:01
ILNPNLICI	HLA-A*02:01
ILNPNLICIF	HLA-B*15:01
ILRNSPNPMLK	HLA-A*03:01
ILRSGLLKY	HLA-B*15:01
ILRSGLLKY	HLA-B*15:01
ILRSGLLKY	HLA-A*30:02
ILTEIQQAY	HLA-B*15:01
ILTEIQQAY	HLA-A*30:02
ILTEIQQAY	HLA-B*35:01
ILVEHSHLI	HLA-A*02:03
ILVEHSHLI	HLA-A*02:03
ILVEHSHLI	HLA-A*02:01
ILVEHSHLI	HLA-A*02:06
IMKKSFKAY	HLA-B*15:01
IMKKSFKAY	HLA-A*30:02
IMKKSFKAY	HLA-B*15:01
IMLNEAMNY	HLA-A*30:02
IMLNEAMNY	HLA-B*15:01
IMLNEAMNY	HLA-A*03:01
IMLNEAMNY	HLA-A*30:02
IMLNEAMNYF	HLA-B*15:01
IMPSDDFSNTF	HLA-B*35:01
IMTESVLQK	HLA-A*03:01
INPWLNRLTW	HLA-B*53:01
IPAPIGGFNY	HLA-B*35:01
IPAPIGGFNY	HLA-B*35:01
IPAPIGGFNY	HLA-B*53:01
IPFLFLSAY	HLA-B*35:01
IPFLFLSAY	HLA-B*53:01
IPFLFLSAY	HLA-B*35:01
IPFTPKNL	HLA-B*51:01
IPFTPKNLSV	HLA-B*51:01
IPGQRELKL	HLA-B*08:01
IPGQRELKL	HLA-B*07:02
IPIKKGTDAKY	HLA-B*35:01
IPKIINKL	HLA-B*51:01
IPSKVIKSY	HLA-B*35:01
IQFDCFMEL	HLA-A*02:06
IQFDCFMEL	HLA-A*02:01
IQFDCFMEL	HLA-A*02:06
IQLDKHQAL	HLA-B*08:01
IQLDKHQAL	HLA-A*02:06
IQLDPVVTDV	HLA-A*02:06
IQLDPVVTDV	HLA-A*02:06
IQLDPVVTDV	HLA-A*02:06
IQLDPVVTDVV	HLA-A*02:06
IQLDPVVTDVV	HLA-A*02:06
IQLDPVVTDVV	HLA-A*02:06
IQQAYIQEL	HLA-A*02:06
IQQLLISTEF	HLA-B*15:01
IQTKFAPGGY	HLA-A*30:02

IRIATVYTWAY	HLA-A*30:02
ISECSVGILHY	HLA-A*01:01
ISICWDGKRAW	HLA-B*57:01
ISICWDGKRAW	HLA-B*58:01
ISQAFGGNY	HLA-A*30:02
ISRLFNMYR	HLA-A*31:01
ISRLFNMYR	HLA-A*31:01
ITHQEQTMGMY	HLA-A*01:01
ITIEAQIW	HLA-B*58:01
ITSQGMTSK	HLA-A*11:01
ITSQGMTSK	HLA-A*03:01
IVDPELFALY	HLA-A*01:01
IVIKMIPNV	HLA-A*02:06
IYDSLGQPVF	HLA-A*24:02
IYDSLGQPVF	HLA-A*23:01
IYGLPGFFNW	HLA-A*23:01
IYGLPGFFNW	HLA-A*24:02
IYGLPGFFNW	HLA-A*23:01
IYGLPGFFNW	HLA-A*24:02
IYKNNTHDL	HLA-A*24:02
IYLMNWCDF	HLA-A*23:01
IYLMNWCDF	HLA-A*24:02
IYMIPRTML	HLA-A*24:02
IYMIPRTML	HLA-A*23:01
IYMIPRTMLEF	HLA-A*23:01
IYMIPRTMLEF	HLA-A*24:02
IYMIPRTMLEF	HLA-A*24:02
IYMIPRTMLEF	HLA-A*23:01
IYPVLLPQM	HLA-A*24:02
IYPVLLPQM	HLA-A*23:01
IYVDLSSYY	HLA-A*30:02
KALNLSPLIQR	HLA-A*31:01
KALSPIKDEW	HLA-B*58:01
KALSPIKDEW	HLA-B*57:01
KALSPIKDEW	HLA-B*58:01
KAYNIISKK	HLA-A*03:01
KAYNIISKK	HLA-A*11:01
KAYNIISKK	HLA-A*30:01
KDAQPPYHW	HLA-B*44:02
KDAQPPYHW	HLA-B*44:02
KEEPPQKRL	HLA-B*40:01
KEEPPQKRL	HLA-B*44:02
KEEPPQKRL	HLA-B*44:03
KEEPPQKRL	HLA-B*40:01
KEEPPQKRL	HLA-B*44:02
KEEPPQKRL	HLA-B*44:03
KEICAKQAQLY	HLA-B*44:03
KEICAKQAQLY	HLA-B*44:03
KEICAKQAQLY	HLA-B*44:02
KEIPKIINKL	HLA-B*40:01
KEKSPQKRL	HLA-B*44:02
KETKQAGRLF	HLA-B*44:02
KFAGSVLNR	HLA-A*31:01
KFAPGGYPLLW	HLA-A*23:01
KFAPGGYPLLW	HLA-A*24:02
KFKDTKSSELY	HLA-A*30:02
KFMVEILIEV	HLA-A*02:03
KFMVEILIEV	HLA-A*02:01
KGKNPSKVIK	HLA-A*30:01

KGKTPFVDSR	HLA-A*31:01
KGLIRSGLRK	HLA-A*03:01
KIDRMKLQF	HLA-A*32:01
KIINKLESI	HLA-A*02:03
KIINKLESI	HLA-A*32:01
KIINKLESI	HLA-A*02:06
KIKSNPLTK	HLA-A*03:01
KIKSNPLTK	HLA-A*30:01
KIKSNPLTK	HLA-A*11:01
KILSAFNTV	HLA-A*02:06
KINEGLLDSK	HLA-A*03:01
KINEGLLDSK	HLA-A*11:01
KINGVISKR	HLA-A*31:01
KINGVISKR	HLA-A*03:01
KIWWKIIGY	HLA-A*32:01
KIWWKIIGY	HLA-A*32:01
KIWWKIIGY	HLA-A*30:02
KIYDSLGQPV	HLA-A*02:03
KIYDSLGQPVF	HLA-A*32:01
KIYTPGANER	HLA-A*31:01
KIYTPGANER	HLA-A*03:01
KIYTPGANERK	HLA-A*03:01
KKYYQIDQPF	HLA-A*23:01
KKYYQIDQPF	HLA-A*23:01
KLDSDLSMY	HLA-A*01:01
KLDSDLSMY	HLA-A*30:02
KLESIDRVLAK	HLA-A*03:01
KLFPSEYSI	HLA-A*32:01
KLFPSEYSI	HLA-A*02:01
KLFPSEYSI	HLA-A*32:01
KLFPSEYSI	HLA-A*02:01
KLFPSEYSI	HLA-A*02:06
KLFPSEYSI	HLA-A*02:03
KLINLDMRL	HLA-A*02:01
KLINLDMRL	HLA-A*02:03
KLINLDMRL	HLA-A*02:01
KLINLDMRL	HLA-A*02:06
KLISAIEYA	HLA-A*02:01
KLISAIEYA	HLA-A*02:06
KLISAIEYA	HLA-A*02:03
KLISYTLPV	HLA-A*02:01
KLISYTLPV	HLA-A*02:03
KLISYTLPV	HLA-A*02:06
KLKGLVVPL	HLA-A*02:03
KLKGLVVPL	HLA-A*02:03
KLKGLVVPLF	HLA-B*15:01
KLKGLVVPLF	HLA-A*32:01
KLMTKTLPK	HLA-A*03:01
KLMTKTLPK	HLA-A*30:01
KLNDSGIYM	HLA-A*02:01
KLNDSGIYM	HLA-A*02:03
KLQFLKKIGK	HLA-A*03:01
KLSKIGLVK	HLA-A*03:01
KLSKIGLVK	HLA-A*30:01
KMIPNVSNM	HLA-B*15:01
KMIPNVSNM	HLA-A*02:03
KMIPNVSNM	HLA-A*32:01
KMLSYAPEI	HLA-A*02:01
KMLSYAPEI	HLA-A*02:06

KMLSYAPEI	HLA-A*32:01
KMLSYAPEI	HLA-A*02:01
KMLSYAPEI	HLA-A*02:06
KMLSYAPEI	HLA-A*32:01
KMLSYAPEI	HLA-A*02:01
KMLSYAPEI	HLA-A*02:06
KMLSYAPEI	HLA-A*32:01
KMLSYAPEI	HLA-A*02:01
KMLSYAPEI	HLA-A*32:01
KMLSYAPEI	HLA-A*02:01
KMLSYAPEI	HLA-A*32:01
KNQKEIQKTY	HLA-B*15:01
KNQKEIQKTY	HLA-A*30:02
KNQKEIQKTY	HLA-B*15:01
KNQKEIQKTY	HLA-A*30:02
KNQKEIQKTY	HLA-B*15:01
KNQKEIQKTY	HLA-A*30:02
KPENCRLSM	HLA-B*07:02
KPKLISYTL	HLA-B*07:02
KPKLISYTL	HLA-B*08:01
KPKNNGESY	HLA-B*35:01
KPNESIGRTI	HLA-B*07:02
KPNESIGRTI	HLA-B*07:02
KPQTEGVSNL	HLA-B*07:02
KPVIGRDIL	HLA-B*07:02
KQAGRLFAK	HLA-A*30:01
KQAGRLFAK	HLA-A*03:01
KQAQLYFER	HLA-A*31:01
KQFHDDLKKY	HLA-A*30:02
KQFHDDLKKY	HLA-B*15:01
KQFHDDLKKY	HLA-B*15:01
KQFHDDLKKY	HLA-A*30:02
KQFHDDLKKYY	HLA-A*30:02
KQFHDDLKKYY	HLA-B*15:01
KQRPGTPMPK	HLA-A*30:01
KQRPGTPMPK	HLA-A*03:01
KSDKTGKKCY	HLA-A*01:01
KSDKTGKKCYY	HLA-A*01:01
KSFLDYHTEF	HLA-B*58:01
KSFLDYHTEF	HLA-B*57:01
KSFLDYHTEF	HLA-A*32:01
KSFLDYHTEF	HLA-A*23:01
KSFLDYHTEF	HLA-B*57:01
KSFLDYHTEF	HLA-B*58:01
KSGLQPKLVSR	HLA-A*31:01
KSMSRCCFW	HLA-B*58:01
KSMSRCCFW	HLA-B*57:01
KSRELDFPLW	HLA-B*58:01
KSRELDFPLW	HLA-B*57:01
KSRELDFPLW	HLA-B*57:01
KSRELDFPLW	HLA-B*58:01
KSRGIPIKK	HLA-A*30:01
KSRGIPIKK	HLA-A*30:01
KSRGIPIKK	HLA-A*30:01
KSRGIPIKK	HLA-A*30:01
KSRGIPIKK	HLA-A*03:01
KSRGIPIKK	HLA-A*31:01
KSRGIPIKK	HLA-A*30:01
KSRGIPIKK	HLA-A*03:01

KSRGIPIKK	HLA-A*31:01
KSRGIPIKK	HLA-A*30:01
KSRGIPIKK	HLA-A*03:01
KSRGIPIKK	HLA-A*31:01
KSSELYHIK	HLA-A*30:01
KSSELYHIK	HLA-A*11:01
KSYYGTMDIK	HLA-A*03:01
KSYYGTMDIKK	HLA-A*03:01
KTARDSSKGK	HLA-A*30:01
KTEQKHGRTF	HLA-B*44:02
KTIKNITAR	HLA-A*31:01
KTIKNITAR	HLA-A*31:01
KTIKNITAR	HLA-A*11:01
KTITNCFLLK	HLA-A*11:01
KTLIRTHIK	HLA-A*30:01
KTLPKGMQER	HLA-A*31:01
KTLPKGMQERR	HLA-A*31:01
KTNTALSTI	HLA-A*32:01
KTPEDDIFIHY	HLA-A*01:01
KTPFVDSRAY	HLA-A*30:02
KTPFVDSRAY	HLA-A*30:02
KTSNQILKPK	HLA-A*03:01
KTSNQILKPK	HLA-A*11:01
KTWTIATIPF	HLA-A*32:01
KTWTIATIPF	HLA-B*58:01
KVASFMLHL	HLA-A*32:01
KVASFMLHL	HLA-A*02:06
KVHPNLPYK	HLA-A*03:01
KVHPNLPYK	HLA-A*30:01
KVHPNLPYK	HLA-A*11:01
KVHPNLPYK	HLA-A*31:01
KVHPNLPYKVK	HLA-A*30:01
KVHPNLPYKVK	HLA-A*03:01
KVIGMLQNI	HLA-A*32:01
KVIGMLQNI	HLA-A*02:06
KVIGMLQNITR	HLA-A*31:01
KVIVYSLIKF	HLA-A*32:01
KVIVYSLIKF	HLA-B*15:01
KVKKEICAK	HLA-A*30:01
KVKKEICAK	HLA-A*03:01
KVLEYAPIMK	HLA-A*03:01
KVLEYAPIMK	HLA-A*11:01
KVLSYAPEI	HLA-A*32:01
KVLSYAPEI	HLA-A*02:06
KVMPYGPSGIK	HLA-A*03:01
KVMPYGPSGIK	HLA-A*30:01
KVMPYGPSGIK	HLA-A*11:01
KVREHMLADK	HLA-A*30:01
KVREHMLADK	HLA-A*03:01
KWAFEIHHRR	HLA-A*31:01
KWYECFLF	HLA-A*23:01
KWYECFLF	HLA-A*24:02
KWYECFLFW	HLA-A*23:01
KWYECFLFW	HLA-A*24:02
KWYECFLFW	HLA-A*23:01
KWYECFLFWF	HLA-A*23:01
KWYECFLFWF	HLA-A*24:02
KYFKENGMVK	HLA-A*30:01
KYIMPSDDF	HLA-A*24:02

KYIMPSDDF	HLA-A*23:01
KYKIKSNPLTK	HLA-A*30:01
KYKIKSNPLTK	HLA-A*03:01
KYLSDLLF	HLA-A*23:01
KYLSDLLFVF	HLA-A*23:01
KYLSDLLFVF	HLA-A*24:02
KYLSDLLFVF	HLA-A*23:01
KYLSDLLFVF	HLA-A*24:02
KYNNYMYLI	HLA-A*24:02
KYNNYMYLI	HLA-A*23:01
KYYQIDQPF	HLA-A*23:01
KYYQIDQPF	HLA-A*24:02
KYYQIDQPF	HLA-A*23:01
KYYQIDQPF	HLA-A*24:02
KYYQIDQPFF	HLA-A*23:01
KYYQIDQPFF	HLA-A*24:02
KYYQIDQPFF	HLA-A*23:01
KYYQIDQPFF	HLA-A*24:02
KYYQIDQPFFV	HLA-A*02:06
KYYSVDYCR	HLA-A*31:01
KYYSVDYCR	HLA-A*31:01
KYYSVDYCRR	HLA-A*31:01
KYYSVDYCRR	HLA-A*31:01
LADKVLEY	HLA-A*01:01
LAEFFSFFR	HLA-A*68:01
LAKTVAQTV	HLA-B*51:01
LAMDEGYFAY	HLA-B*35:01
LAMDEGYFAY	HLA-B*15:01
LAMDEGYFAY	HLA-A*01:01
LAMDEGYFAY	HLA-A*01:01
LAMDEGYFAY	HLA-B*35:01
LARALRSHMW	HLA-B*58:01
LARALRSHMW	HLA-B*57:01
LARALRSHMW	HLA-B*57:01
LDPVVTDVVY	HLA-B*35:01
LDPVVTDVVY	HLA-B*35:01
LDPVVTDVVY	HLA-B*35:01
LDPVVTDVVY	HLA-B*35:01
LDPVVTDVVY	HLA-B*35:01
LDPVVTDVVY	HLA-B*35:01
LEESIQTKF	HLA-B*44:03
LEESIQTKF	HLA-B*44:02
LEQQSLFSF	HLA-B*44:03
LEQQSLFSF	HLA-B*44:02
LETDDYNGIY	HLA-A*01:01
LETDDYNGIY	HLA-A*01:01
LFPVMGNRIY	HLA-B*35:01
LFSFDNVKNF	HLA-A*23:01
LGQSISIKW	HLA-B*58:01
LGQSISIKW	HLA-B*57:01
LIASGVGKY	HLA-A*30:02
LIASGVGKY	HLA-B*15:01
LIASGVGKY	HLA-A*26:01
LIASGVGKYF	HLA-B*15:01
LISRSNALW	HLA-B*58:01
LISRSNALW	HLA-B*58:01
LKLDSDLSMY	HLA-A*01:01
LKSMSRCCFW	HLA-B*58:01
LLAEFFSFF	HLA-B*15:01

LLAEFFSFFR	HLA-A*68:01
LLDSKILSA	HLA-A*02:01
LLDTVNPSL	HLA-A*02:01
LLDTVNPSL	HLA-A*02:06
LLKNKIWCI	HLA-B*08:01
LLLPTQGPY	HLA-B*15:01
LLSQSLSVRKF	HLA-B*15:01
LLVTSYMIY	HLA-B*15:01
LLVTSYMIYL	HLA-A*02:01
LLWSFAMGV	HLA-A*02:01
LPAHASKHI	HLA-B*51:01
LPIPEYTEV	HLA-B*51:01
LPIPEYTEV	HLA-B*53:01
LPRAAHEIL	HLA-B*07:02
LPSGAQRLF	HLA-B*35:01
LPSGAQRLF	HLA-B*53:01
LPSGAQRLF	HLA-B*35:01
LPYKVKKEI	HLA-B*51:01
LPYKVKKEI	HLA-B*07:02
LQDPVSNSM	HLA-A*02:06
LQPSIPREF	HLA-B*15:01
LSHPRVFKR	HLA-A*31:01
LSHPRVFKR	HLA-A*31:01
LSHPRVFKR	HLA-A*33:01
LSLDLALSK	HLA-A*11:01
LSLDLALSKY	HLA-A*01:01
LSNREVKIW	HLA-B*57:01
LSNREVKIW	HLA-B*58:01
LSNREVKIWW	HLA-B*58:01
LSNREVKIWW	HLA-B*57:01
LSQNLLVTSY	HLA-B*15:01
LSQNLLVTSY	HLA-A*30:02
LTEIQQAY	HLA-A*01:01
LTIDDCVKNW	HLA-B*58:01
LTIDDCVKNW	HLA-B*57:01
LTIDDCVKNW	HLA-B*58:01
LTKDIVIKM	HLA-A*26:01
LTNSLLNLR	HLA-A*68:01
LTPEMVLMY	HLA-A*01:01
LTPEMVLMY	HLA-A*01:01
LTPEMVLMY	HLA-A*26:01
LTVNPLVVNW	HLA-B*58:01
LVKGVTRKY	HLA-A*26:01
LVKGVTRKY	HLA-B*15:01
LVKGVTRKY	HLA-A*30:02
LVLVCFPVY	HLA-B*35:01
LVSDAKMLSY	HLA-A*01:01
LVSDAKMLSY	HLA-B*15:01
LVSDAKMLSY	HLA-A*01:01
LVSDAKMLSY	HLA-B*15:01
LVSDAKMLSY	HLA-A*01:01
LVSDAKMLSY	HLA-B*15:01
LVSDAKMLSY	HLA-A*01:01
LVSDAKMLSY	HLA-A*01:01
LVSDAKVLSY	HLA-A*01:01
LVSNTSKHTY	HLA-B*15:01
LVSNTSKHTY	HLA-A*30:02
LVVSSHVPRF	HLA-B*15:01
LWGLIDPLF	HLA-A*23:01

LWGLIDPLF	HLA-A*24:02
LWGLIDPLF	HLA-A*23:01
LYFPAVGFL	HLA-A*23:01
LYFPAVGFL	HLA-A*24:02
LYLGQSISI	HLA-A*24:02
MAGFFATIR	HLA-A*68:01
MAGFFATIRF	HLA-B*58:01
MAGFFATIRF	HLA-B*15:01
MAGFFATIRF	HLA-B*35:01
MASILLTLF	HLA-B*58:01
MASILLTLF	HLA-B*35:01
MASILLTLF	HLA-B*53:01
MASILLTLF	HLA-B*15:01
MASILLTLF	HLA-B*53:01
MASILLTLF	HLA-B*58:01
MASILLTLFR	HLA-A*68:01
MASILLTLFR	HLA-A*31:01
MEMLKEETW	HLA-B*44:02
MEMLKEETW	HLA-B*44:03
MEVLKEEAW	HLA-B*44:02
MEVLKEEAW	HLA-B*44:03
MIKDALQSI	HLA-A*02:03
MIYLMNWCDF	HLA-A*23:01
MIYLMNWCDF	HLA-A*24:02
MLADKVLEY	HLA-B*15:01
MLADKVLEY	HLA-B*35:01
MLADKVLEY	HLA-A*03:01
MLADKVLEY	HLA-A*30:02
MLADKVLEY	HLA-A*01:01
MLADKVLEY	HLA-B*15:01
MLADKVLEY	HLA-A*30:02
MLADKVLEY	HLA-A*01:01
MLADKVLEY	HLA-A*26:01
MLADKVLEY	HLA-B*35:01
MLADKVLEYA	HLA-A*02:03
MLEYVLSGAY	HLA-B*15:01
MLEYVLSGAY	HLA-A*30:02
MLKEETWRIY	HLA-B*15:01
MLNEAMNYF	HLA-B*15:01
MLNEAMNYF	HLA-B*35:01
MMASILLTL	HLA-A*02:03
MMASILLTL	HLA-A*02:01
MMASILLTL	HLA-A*02:06
MMASILLTL	HLA-A*32:01
MMASILLTL	HLA-B*15:01
MMASILLTL	HLA-A*32:01
MMASILLTL	HLA-A*02:01
MMASILLTL	HLA-A*02:03
MMASILLTLF	HLA-B*15:01
MMASILLTLF	HLA-A*02:03
MMASILLTLF	HLA-B*58:01
MMASILLTLF	HLA-A*02:01
MMASILLTLF	HLA-A*32:01
MMASILLTLF	HLA-A*23:01
MMASILLTLFR	HLA-A*68:01
MMEMLKEETW	HLA-B*58:01
MMLLYQSTL	HLA-A*02:01
MMLLYQSTL	HLA-A*02:03
MPKSRGIPI	HLA-B*08:01

MPKSRGIPI	HLA-B*07:02
MPKSRGIPI	HLA-B*08:01
MPKSRGIPI	HLA-B*07:02
MPKSRGIPI	HLA-B*08:01
MPKSRGIPI	HLA-B*07:02
MPSDDFSNTF	HLA-B*35:01
MPSDDFSNTF	HLA-B*53:01
MPSDDFSNTF	HLA-B*35:01
MPSDDFSNTF	HLA-B*53:01
MPSDDFSNTFF	HLA-B*53:01
MPSDDFSNTFF	HLA-B*53:01
MPSDDFSNTFF	HLA-B*35:01
MPTESKKV	HLA-B*51:01
MPTESKKVRF	HLA-B*53:01
MPYGPSGI	HLA-B*51:01
MTESVLQKV	HLA-A*68:02
MTIQAISQA	HLA-A*68:02
MTIQAISQAF	HLA-B*15:01
MTIQAISQAF	HLA-A*26:01
MTIQAISQAF	HLA-A*26:01
MTRLAVKPK	HLA-A*30:01
MTYNWTQW	HLA-B*58:01
MTYNWTQW	HLA-B*57:01
MTYNWTQW	HLA-B*58:01
MTYNWTQWL	HLA-A*68:02
MTYNWTQWL	HLA-A*32:01
MTYNWTQWL	HLA-A*68:02
MVNGIPVMER	HLA-A*68:01
MVNGIPVMER	HLA-A*68:01
MVNGIPVMER	HLA-A*31:01
MVNGIPVMER	HLA-A*33:01
MVNGIPVMERR	HLA-A*68:01
MVNGIPVMERR	HLA-A*68:01
MYCDVLEGR	HLA-A*33:01
MYCDVLEGR	HLA-A*33:01
MYGWFFVPR	HLA-A*33:01
MYVLYLMQR	HLA-A*33:01
MYVLYLMQR	HLA-A*33:01
NAIAFNLLV	HLA-B*51:01
NAIAFNLLVY	HLA-B*35:01
NEPYGAAVQL	HLA-B*40:01
NIMIIQNYTR	HLA-A*68:01
NIMKKSFKAY	HLA-B*15:01
NIRRKVLIL	HLA-B*08:01
NIRRKVLIL	HLA-B*08:01
NITARTILR	HLA-A*33:01
NLHGKRKSL	HLA-B*08:01
NLHGKRKSL	HLA-B*08:01
NLLSQSLSV	HLA-A*02:01
NLSPLIQRY	HLA-A*30:02
NLSPLIQRY	HLA-A*26:01
NPDSTEVYL	HLA-B*53:01
NPFFALTQQW	HLA-B*53:01
NPLTKDIVI	HLA-B*51:01
NPPIKTGVL	HLA-B*08:01
NPQEKICVL	HLA-B*08:01
NPSKVIKSY	HLA-B*35:01
NPSKVIKSY	HLA-B*53:01
NPTAVPFTL	HLA-B*53:01

NPTAVPFTL	HLA-B*35:01
NPTAVPFTL	HLA-B*51:01
NPTAVPFTL	HLA-B*07:02
NPTAVPFTL	HLA-B*53:01
NPTAVPFTL	HLA-B*35:01
NPTAVPFTL	HLA-B*51:01
NPTAVPFTL	HLA-B*07:02
NPTAVPFTLR	HLA-A*68:01
NPTAVPFTLR	HLA-A*68:01
NPTAVPFTLR	HLA-A*68:01
NPTAVPFTLR	HLA-A*68:01
NPTAVPFTLR	HLA-A*68:01
NPWLNRLTW	HLA-B*53:01
NQILKPKLISY	HLA-B*15:01
NQKEIQKTY	HLA-B*15:01
NQKEIQKTY	HLA-B*15:01
NQKEIQKTY	HLA-B*15:01
NRGYLEPMYFR	HLA-A*33:01
NTCSVDLAR	HLA-A*68:01
NTEAAVLSRY	HLA-A*26:01
NTEAAVLSRY	HLA-A*01:01
NTFFPHDTDR	HLA-A*68:01
NTFFPHDTDR	HLA-A*68:01
NTIKSLMLL	HLA-A*68:02
NTIKSLMLLY	HLA-A*26:01
NTIKSLMLLY	HLA-A*68:01
NTIKSLMLLY	HLA-A*11:01
NTIKSLMLLY	HLA-A*26:01
NTMYGWFFV	HLA-A*68:02
NTMYGWFFV	HLA-A*68:02
NTSDLDFVIFY	HLA-A*01:01
NTSDLDFVIFY	HLA-A*01:01
NTSKHTYKV	HLA-A*68:02
NTSKSFLDY	HLA-A*01:01
NTSKSFLDY	HLA-A*01:01
NTYSRLEDR	HLA-A*33:01
NVIISLGKY	HLA-A*26:01
NVKRPTKAL	HLA-B*08:01
NVKRPTKALR	HLA-A*33:01
NVNLDSIKLY	HLA-A*26:01
NVNLDSIKLY	HLA-A*26:01
NVNLDSIKLY	HLA-A*26:01
NWTQWLQTLY	HLA-A*01:01
NYLNLSRIF	HLA-A*23:01
NYLNLSRIF	HLA-A*23:01
NYLNLSRIF	HLA-A*24:02
NYMYLICYGF	HLA-A*23:01
NYMYLICYGF	HLA-A*24:02
PFFALTQQW	HLA-A*23:01
PTSSGDLYY	HLA-A*01:01
QAFNNPASK	HLA-A*11:01
QAFNNPASK	HLA-A*30:01
QELLKENSF	HLA-B*44:02
QELLKENSF	HLA-B*44:03
QEQTMGMYVLY	HLA-B*44:03
QEQTMGMYVLY	HLA-B*44:03
QEQTMGMYVLY	HLA-B*44:02
QESKSRELDF	HLA-B*44:02
QETAEKTVY	HLA-B*44:02

QETAEKTVY	HLA-B*44:03
QETAEKTVYV	HLA-A*68:02
QGKDAQPLYY	HLA-A*01:01
QITAGVALY	HLA-A*26:01
QKNQKEIQKTY	HLA-B*15:01
QKNQKEIQKTY	HLA-B*15:01
QKNQKEIQKTY	HLA-B*15:01
QLDKHQAL	HLA-B*08:01
QLDPVVTDV	HLA-A*02:01
QLDPVVTDV	HLA-A*02:06
QLDPVVTDV	HLA-A*02:01
QLDPVVTDV	HLA-A*02:06
QLDPVVTDV	HLA-A*02:01
QLDPVVTDV	HLA-A*02:06
QLDPVVTDVVY	HLA-A*01:01
QLDPVVTDVVY	HLA-A*01:01
QLDPVVTDVVY	HLA-A*01:01
QPFFVPTKI	HLA-B*51:01
QPFFVPTKI	HLA-B*53:01
QPLISRSNAL	HLA-B*07:02
QPPYHWSI	HLA-B*51:01
QPPYHWSI	HLA-B*51:01
QPSIKDQTKAW	HLA-B*53:01
QPSIKDQTKAW	HLA-B*53:01
QPSIPREFM	HLA-B*07:02
QPVFYQASF	HLA-B*35:01
QPVFYQASF	HLA-B*53:01
QPVFYQASFSW	HLA-B*53:01
QQGKDAQPLY	HLA-B*15:01
QQGKDAQPPY	HLA-B*15:01
QQGKDAQPPY	HLA-B*15:01
QQKRVNPFF	HLA-B*15:01
QQKRVNPFF	HLA-B*15:01
QQLLISTEF	HLA-B*15:01
QQLLISTEF	HLA-B*15:01
QSISIKWAF	HLA-B*58:01
QSISIKWAF	HLA-A*32:01
QSISIKWAF	HLA-A*32:01
QSISIKWAF	HLA-B*57:01
QSISIKWAF	HLA-B*58:01
QTAENPVFTV	HLA-A*68:02
QTKFAPGGY	HLA-A*30:02
QTKFAPGGY	HLA-A*26:01
QTMGMYVLY	HLA-A*11:01
QTMGMYVLY	HLA-A*68:01
QTMGMYVLY	HLA-A*30:02
QTMGMYVLY	HLA-A*26:01
QTMGMYVLY	HLA-A*32:01
QTMGMYVLY	HLA-A*26:01
QTMGMYVLY	HLA-A*30:02
QTMGMYVLY	HLA-A*32:01
QTMGMYVLY	HLA-A*01:01
QTRNIHLLGR	HLA-A*31:01
QTVLEIITK	HLA-A*11:01
QTVNPLVVNW	HLA-B*57:01
QTVNPLVVNW	HLA-B*58:01
QVAELAAAV	HLA-A*68:02
QVDAELPIPEY	HLA-A*01:01
RALRSHMW	HLA-B*58:01

RALRSHMW	HLA-B*57:01
RALRSHMW	HLA-B*58:01
RALRSHMWR	HLA-A*31:01
RALRSHMWR	HLA-A*31:01
RAVIKNSQK	HLA-A*30:01
RCCPMLPKLQF	HLA-B*07:02
REAKFAAGGVL	HLA-B*40:01
REDLILPEL	HLA-B*40:01
REDLILPEL	HLA-B*40:01
REHMLADKVL	HLA-B*40:01
REHSSIRVPY	HLA-B*44:02
REHSSIRVPY	HLA-B*44:03
REIGPRAPY	HLA-B*44:02
REIGPRAPY	HLA-B*44:03
REKNGFKEVW	HLA-B*44:02
REKNGFKEVW	HLA-B*44:03
RELALGRVI	HLA-B*40:01
RELALGRVIY	HLA-B*44:03
RELALGRVIY	HLA-B*44:02
RELALGRVIY	HLA-B*15:01
RELKLFPSEY	HLA-B*44:03
RELKLFPSEY	HLA-B*44:02
RELKLFPSEY	HLA-A*30:02
RELKLFPSEY	HLA-B*15:01
RELKLFPSEY	HLA-B*44:03
RELKLFPSEY	HLA-B*44:02
RELLKENSL	HLA-B*40:01
RELLKENSL	HLA-B*40:01
REMSISSL	HLA-B*40:01
RETDLVHL	HLA-B*40:01
RETDLVHL	HLA-B*40:01
RETDLVHL	HLA-B*40:01
RFALSNGVLF	HLA-A*23:01
RFALSNGVLF	HLA-A*24:02
RFALSNGVLF	HLA-A*23:01
RFALSNGVLF	HLA-A*24:02
RGYLEPMYFR	HLA-A*31:01
RGYLEPMYFR	HLA-A*31:01
RIATVYTW	HLA-B*58:01
RIATVYTW	HLA-A*32:01
RIATVYTWAY	HLA-A*30:02
RIATVYTWAY	HLA-B*15:01
RIATVYTWAY	HLA-B*58:01
RIATVYTWAY	HLA-A*32:01
RIATVYTWAY	HLA-A*03:01
RIFFLSITK	HLA-A*03:01
RIFFLSITK	HLA-A*11:01
RIFFLSITK	HLA-A*30:01
RIGLNSSSCY	HLA-A*30:02
RIYNIVSMI	HLA-A*32:01
RLAGVIMAGV	HLA-A*02:03
RLETDDYNGIY	HLA-A*01:01
RLETDDYNGIY	HLA-A*01:01
RLFAKMTY	HLA-A*03:01
RLFAKMTYK	HLA-A*03:01
RLFAKMTYK	HLA-A*03:01
RLFAKMTYK	HLA-A*30:01
RLFAKMTYK	HLA-A*11:01
RLFAKMTYK	HLA-A*31:01

RLFAKMTYKMR	HLA-A*31:01
RLFAQMGFQK	HLA-A*03:01
RLFNMYRSY	HLA-A*32:01
RLFNMYRSY	HLA-B*15:01
RLFNMYRSY	HLA-A*30:02
RLFNMYRSY	HLA-A*03:01
RLFNMYRSY	HLA-A*30:01
RLFNMYRSY	HLA-A*32:01
RLFNMYRSY	HLA-A*30:02
RLFNMYRSY	HLA-B*15:01
RLFNMYRSYF	HLA-A*32:01
RLFNMYRSYF	HLA-B*15:01
RLFNMYRSYF	HLA-A*32:01
RLLDTVNPSL	HLA-A*02:01
RLNGILTPI	HLA-A*02:03
RLNGILTPI	HLA-A*32:01
RLNGILTPIK	HLA-A*03:01
RLNHIEEQV	HLA-A*02:03
RLNHIEEQV	HLA-A*02:01
RLNLHGKRK	HLA-A*03:01
RLRDKSTQF	HLA-B*15:01
RLRDKSTQF	HLA-B*15:01
RLRDKSTQF	HLA-A*32:01
RLRDKSTQF	HLA-B*08:01
RLRDKSTQFK	HLA-A*03:01
RLRDKSTQFK	HLA-A*30:01
RLRLETDDY	HLA-A*30:02
RLRMNLRAL	HLA-B*07:02
RLSHHDYNQF	HLA-B*15:01
RLSIGSPSK	HLA-A*03:01
RLSIGSPSK	HLA-A*30:01
RLTNSLLNLR	HLA-A*31:01
RMMEMLKEETW	HLA-B*58:01
RMMEVLKEEAW	HLA-A*32:01
RMMMETTVK	HLA-A*03:01
RPGALIRSL	HLA-B*07:02
RPGALIRSL	HLA-B*07:02
RPGALIRSLL	HLA-B*07:02
RPGALIRSLL	HLA-B*07:02
RPKLFAVKI	HLA-B*07:02
RPNSHYIL	HLA-B*07:02
RPRGRHTM	HLA-B*07:02
RPRGRHTM	HLA-B*07:02
RPRGRHTM	HLA-B*08:01
RPRGRHTMV	HLA-B*07:02
RPRGRHTMV	HLA-B*07:02
RPRGRHTMV	HLA-B*08:01
RPRGRHTMVDL	HLA-B*07:02
RPSVENHKY	HLA-B*35:01
RPTKALRSA	HLA-B*07:02
RPTKALRSAI	HLA-B*07:02
RPTKALRSAI	HLA-B*07:02
RPTSSGDLYY	HLA-A*01:01
RQRPGTPMPK	HLA-A*30:01
RQRPGTPMPK	HLA-A*03:01
RQRPGTPMPK	HLA-A*30:01
RQRPGTPMPK	HLA-A*03:01
RSAIRIATVY	HLA-B*58:01
RSAIRIATVY	HLA-A*30:02

RSAIRIATVY	HLA-B*15:01
RSAIRIATVY	HLA-B*57:01
RSKLMTKTLPK	HLA-A*30:01
RSMFIDDLL	HLA-B*58:01
RSMGALNINR	HLA-A*31:01
RSMGALNINR	HLA-A*31:01
RSPSHHLEPY	HLA-A*30:02
RSRLAAKAAK	HLA-A*30:01
RSWNTSDLDF	HLA-B*58:01
RSWNTSDLDF	HLA-B*57:01
RSYFGLVLVCF	HLA-A*23:01
RSYQSKLGR	HLA-A*31:01
RSYQSKLGR	HLA-A*31:01
RSYQSKLGR	HLA-A*30:01
RSYQSKLGR	HLA-A*03:01
RTFGHPILEAK	HLA-A*03:01
RTFGHPILEAK	HLA-A*11:01
RTFGHPILEAK	HLA-A*30:01
RTIEGQSIR	HLA-A*31:01
RTIEGQSIR	HLA-A*31:01
RVLAKTNTA	HLA-A*30:01
RVRPTSSGDLY	HLA-A*30:02
RVRPTSSGDLY	HLA-A*30:02
RVRPTSSGDLY	HLA-A*30:01
RYEDNTGTKF	HLA-A*24:02
RYESMAIFAER	HLA-A*31:01
RYKCSNTSK	HLA-A*30:01
RYKCSNTSK	HLA-A*30:01
RYLPSGAQR	HLA-A*31:01
RYLPSGAQRL	HLA-A*24:02
RYLPSGAQRL	HLA-A*23:01
RYLPSGAQRLF	HLA-A*23:01
RYLPSGAQRLF	HLA-A*24:02
RYLPSGAQRLF	HLA-A*24:02
RYLPSGAQRLF	HLA-A*23:01
RYPALALNEF	HLA-A*24:02
RYPALALNEF	HLA-A*23:01
RYPALALNEF	HLA-B*07:02
RYPALALNEF	HLA-B*35:01
RYPALALNEF	HLA-A*24:02
RYPALALNEF	HLA-A*23:01
SAIEYAQLR	HLA-A*68:01
SAIEYAQLR	HLA-A*68:01
SAIRIATV	HLA-B*51:01
SAIRIATVY	HLA-B*35:01
SAIRIATVY	HLA-B*15:01
SAIRIATVY	HLA-A*30:02
SAIRIATVY	HLA-B*35:01
SAIRIATVY	HLA-B*15:01
SAIRIATVY	HLA-A*30:02
SAIRIATVYTW	HLA-B*58:01
SAIRIATVYTW	HLA-B*57:01
SAIRIATVYTW	HLA-B*57:01
SAIRIATVYTW	HLA-B*58:01
SAKGRAVEI	HLA-B*08:01
SASKKIFNL	HLA-B*08:01
SAVKEKSPQK	HLA-A*30:01
SAVYNNEFY	HLA-B*35:01
SAVYNNEFYY	HLA-A*11:01

SAVYNNEFYY	HLA-A*30:02
SAVYNNEFYY	HLA-A*30:02
SAYETNTRI	HLA-B*51:01
SDAKMLSY	HLA-A*01:01
SDAKMLSY	HLA-A*01:01
SDAKVLSY	HLA-A*01:01
SECSVGILHY	HLA-B*44:03
SECSVGILHY	HLA-B*44:02
SEDPIIQEL	HLA-B*40:01
SEDPIIQEL	HLA-B*44:03
SEDPIIQEL	HLA-B*44:02
SEDPIIQELL	HLA-B*40:01
SEDPIIREL	HLA-B*40:01
SEDPIIREL	HLA-B*44:03
SEDPIIREL	HLA-B*44:02
SEDPIIREL	HLA-B*40:01
SEDPIIREL	HLA-B*44:03
SEDPIIREL	HLA-B*44:02
SEDPIIRELL	HLA-B*40:01
SEDPIIRELL	HLA-B*44:02
SEDPIIRELL	HLA-B*44:03
SEDPIIRELL	HLA-B*40:01
SEDPIIRELL	HLA-B*44:02
SEDPIIRELL	HLA-B*44:03
SESMEGVSDF	HLA-B*44:02
SESMEGVSDF	HLA-B*44:03
SETRRWAKY	HLA-B*44:02
SETRRWAKY	HLA-B*44:03
SEWISIVPNF	HLA-B*44:03
SEWISIVPNF	HLA-A*23:01
SEWISIVPNF	HLA-B*44:02
SEWISIVPNF	HLA-B*44:03
SFCGIQFDCF	HLA-A*23:01
SFDNVKNFR	HLA-A*33:01
SFLDYHTEF	HLA-A*23:01
SFLDYHTEF	HLA-A*24:02
SFLDYHTEF	HLA-A*23:01
SFLDYHTEF	HLA-A*24:02
SGLQPKLVSR	HLA-A*31:01
SGRQSVTFK	HLA-A*30:01
SGSEDPIIQEL	HLA-B*40:01
SIGSPSKIY	HLA-A*30:02
SILLTLFRR	HLA-A*31:01
SIMTESVLQK	HLA-A*11:01
SIMTESVLQK	HLA-A*03:01
SINENVNEK	HLA-A*11:01
SIRDNLQAK	HLA-A*30:01
SIRDNLQAK	HLA-A*30:01
SITGQIIYV	HLA-A*02:06
SITGQIIYV	HLA-A*02:01
SITGQIIYV	HLA-A*02:03
SITGQIIYV	HLA-A*68:02
SITSQGMTSK	HLA-A*11:01
SIVPNFILV	HLA-A*02:06
SIVPNFILV	HLA-A*68:02
SIVPNFILV	HLA-A*02:03
SKTEQKHGRTF	HLA-B*44:02
SKTEQKHGRTF	HLA-B*44:03
SKYLSDLLFVF	HLA-A*23:01

SLADSVPSSSV	HLA-A*02:03
SLADSVPSSSV	HLA-A*02:01
SLADSVPSSSV	HLA-A*02:06
SLDKDGFKV	HLA-A*02:01
SLDLALSKY	HLA-A*01:01
SLGKYLGSV	HLA-A*02:03
SLHIKINGV	HLA-A*02:03
SLIDTSSTI	HLA-A*02:03
SLIDTSSTI	HLA-A*02:01
SLLNDPDIEAV	HLA-A*02:01
SLMDINPWL	HLA-A*02:01
SLMDINPWL	HLA-A*02:06
SLMDINPWL	HLA-A*02:03
SLMDINPWL	HLA-A*02:01
SLMDINPWL	HLA-A*02:06
SLMDINPWL	HLA-A*02:03
SLREDIITSK	HLA-A*03:01
SLYILRQSK	HLA-A*03:01
SLYILRQSK	HLA-A*30:01
SMFIDDLLSI	HLA-A*02:03
SMFIDDLLSI	HLA-A*02:03
SMGIRPNSHY	HLA-B*15:01
SMGIRPNSHY	HLA-B*15:01
SMIEPLVLA	HLA-A*02:03
SMIEPLVLA	HLA-A*02:06
SMIEPLVLA	HLA-A*02:01
SMIEPLVLAL	HLA-A*02:03
SMIEPLVLAL	HLA-A*02:01
SMIEPLVLAL	HLA-A*02:06
SMIPKYKIY	HLA-B*15:01
SMIPKYKIY	HLA-B*15:01
SMIPKYKIY	HLA-A*30:02
SMLSMIILY	HLA-A*30:02
SMNTKSASK	HLA-A*03:01
SMNTKSASK	HLA-A*30:01
SMYMKDKAL	HLA-B*08:01
SPELRWEL	HLA-B*08:01
SPIKDEWDSVY	HLA-B*35:01
SPIVSGKLI	HLA-B*51:01
SPNPMLKGL	HLA-B*07:02
SPQKRLPM	HLA-B*07:02
SPQKRLPM	HLA-B*08:01
SPQKRLPML	HLA-B*07:02
SPQKRLPML	HLA-B*08:01
SPSHHLEPY	HLA-B*35:01
SPSHHLEPY	HLA-B*35:01
SPSKIYDSL	HLA-B*07:02
SPSSWEHGGYL	HLA-B*07:02
SPVIAEHYY	HLA-B*35:01
SPVIAEHYY	HLA-B*35:01
SPVIAEHYY	HLA-B*35:01
SPVIAEHYY	HLA-B*35:01
SPVIAEHYY	HLA-B*53:01
SPVIAEHYY	HLA-B*35:01
SPVIAEHYY	HLA-B*53:01
SPVIAEHYY	HLA-B*35:01
SPVIAEHYY	HLA-B*53:01
SQNLLVTSY	HLA-B*15:01
SQNLLVTSY	HLA-A*30:02

SQNLLVTSY	HLA-B*15:01
SQNLLVTSY	HLA-A*30:02
SQQGKDAQPLY	HLA-B*15:01
SQQGKDAQPLY	HLA-A*30:02
SQSKWYECF	HLA-B*15:01
SQSLSVRKF	HLA-B*15:01
SQSLSVRKF	HLA-B*15:01
SRLFNMYRSY	HLA-B*15:01
SRLFNMYRSY	HLA-A*30:02
SRLFNMYRSY	HLA-A*03:01
SSGPLNHLVPW	HLA-B*57:01
SSGPLNHLVPW	HLA-B*58:01
SSIQQPSIK	HLA-A*11:01
SSIQQPSIK	HLA-A*11:01
SSIQQPSIK	HLA-A*11:01
SSKGKTPFV	HLA-A*30:01
SSMNQSLQQSK	HLA-A*11:01
SSRDVIKTL	HLA-A*30:01
SSSEVIVGI	HLA-A*68:02
SSSEVIVGI	HLA-A*68:02
SSSEVIVGI	HLA-A*68:02
SSYYIIVRV	HLA-A*68:02
STASINENV	HLA-A*68:02
STDNQAMIK	HLA-A*11:01
STEFSINETL	HLA-B*40:01
STHLFIYSK	HLA-A*11:01
STHLFIYSK	HLA-A*11:01
STHLFIYSK	HLA-A*30:01
STIEGHLVSM	HLA-A*26:01
STITIPANI	HLA-A*68:02
STLGQSISF	HLA-A*32:01
STLGQSISF	HLA-A*32:01
STLGQSISFY	HLA-A*30:02
STLGQSISFY	HLA-A*11:01
STLGQSISFY	HLA-A*30:02
STNEAVVKL	HLA-A*68:02
STRDSLQIK	HLA-A*30:01
STRDSLQIK	HLA-A*11:01
STSNNLSHR	HLA-A*68:01
STSPTDGTIGK	HLA-A*11:01
STSPTDGTIGK	HLA-A*11:01
STSPTDGTIGK	HLA-A*11:01
SVLEGKFRL	HLA-A*02:06
SVLEGKFRLR	HLA-A*31:01
SVLMGVINSI	HLA-A*02:03
SVLNRVSRY	HLA-A*30:02
SVLNRVSRY	HLA-A*30:02
SVLNRVSRY	HLA-A*26:01
SVMENYKTR	HLA-A*68:01
SVMENYKTR	HLA-A*31:01
SVMENYKTR	HLA-A*33:01
SVRKFMVEI	HLA-A*30:01
SVYPREVLSY	HLA-A*03:01
SVYPREVLSY	HLA-A*11:01
SVYPREVLSY	HLA-B*15:01
SVYPREVLSY	HLA-A*30:02
SVYPREVLSY	HLA-A*30:02
SVYPREVLSY	HLA-B*15:01
SVYPREVLSY	HLA-A*03:01

SVYPREVLSY	HLA-A*26:01
SVYPREVLSY	HLA-A*11:01
SVYPREVLSY	HLA-A*32:01
SYFGLVLVCF	HLA-A*23:01
SYFGLVLVCF	HLA-A*24:02
SYFGLVLVCF	HLA-A*23:01
SYMIYLMNW	HLA-A*23:01
SYMIYLMNW	HLA-A*24:02
SYMIYLMNW	HLA-A*23:01
SYMIYLMNW	HLA-A*24:02
SYNQHQFAL	HLA-A*24:02
SYNQHQFAL	HLA-A*23:01
SYYIIVRVY	HLA-A*30:02
SYYIIVRVYF	HLA-A*23:01
SYYIIVRVYF	HLA-A*23:01
TAAQITAGV	HLA-A*68:02
TAEESETRRW	HLA-B*44:02
TAEESETRRW	HLA-B*44:03
TEAAVLSRY	HLA-B*44:03
TEAAVLSRY	HLA-B*44:02
TEFNPHNHY	HLA-B*44:03
TEFNPHNHY	HLA-B*44:03
TEFNPHNHY	HLA-B*44:02
TEFSINETL	HLA-B*40:01
TEFSINETL	HLA-B*40:01
TEFSINETL	HLA-B*44:03
TEFSINETLTL	HLA-B*40:01
TEIGPKVSL	HLA-B*40:01
TEIGPKVSL	HLA-B*44:03
TEIGPKVSL	HLA-B*44:02
TEIILNPNL	HLA-B*40:01
TELSLDLAL	HLA-B*40:01
TEQKHGRTF	HLA-B*44:03
TEQKHGRTF	HLA-B*44:02
TESVLQKVM	HLA-B*44:03
TESVLQKVM	HLA-B*44:02
TETIISTHL	HLA-B*40:01
TETIISTHLF	HLA-B*44:03
TETIISTHLF	HLA-B*44:02
TEVDNNHLI	HLA-B*40:01
TEVDNNHLI	HLA-B*44:03
TEVDNNHLIY	HLA-A*01:01
TEVDNNHLIY	HLA-B*44:03
TEVDNNHLIY	HLA-A*01:01
TEVDNNHLIY	HLA-B*44:02
TFFPHDTDR	HLA-A*33:01
TGKLKGLVV	HLA-B*08:01
TGQKVQMTY	HLA-A*30:02
TIATIPFLF	HLA-A*23:01
TIATIPFLF	HLA-A*32:01
TIISTHLFIY	HLA-A*11:01
TIKSLMLLY	HLA-A*30:02
TIKSLMLLY	HLA-A*30:02
TIKSLMLLY	HLA-A*26:01
TIKSLMLLYR	HLA-A*31:01
TIMNCVTKK	HLA-A*11:01
TIPFLFLSAY	HLA-B*35:01
TIRFGLETR	HLA-A*33:01
TKTIKNITAR	HLA-A*68:01

TLFALDVIR	HLA-A*68:01
TLFRRTKKK	HLA-A*03:01
TLGQSISFY	HLA-A*30:02
TLVDETRSA	HLA-A*02:03
TMVDLLSDL	HLA-A*02:03
TMYGWFFVPR	HLA-A*31:01
TMYGWFFVPR	HLA-A*33:01
TPEDDIFIHY	HLA-B*35:01
TPEDDIFIHY	HLA-B*53:01
TPEDDIFIHY	HLA-B*44:03
TPFVDSRAY	HLA-B*35:01
TPFVDSRAY	HLA-B*35:01
TPFVDSRAY	HLA-B*53:01
TPGFPISRL	HLA-B*07:02
TPGFPISRLF	HLA-B*53:01
TPIKGALEI	HLA-B*51:01
TPIKGALEI	HLA-B*53:01
TPIKGALEIY	HLA-B*35:01
TPMPKSRGI	HLA-B*07:02
TPMPKSRGI	HLA-B*51:01
TPMPKSRGI	HLA-B*08:01
TPMPKSRGI	HLA-B*07:02
TPMPKSRGI	HLA-B*51:01
TPMPKSRGI	HLA-B*08:01
TPMPKSRGI	HLA-B*07:02
TPMPKSRGI	HLA-B*51:01
TPSNPNTVY	HLA-B*35:01
TPSNPNTVY	HLA-B*53:01
TPVSTSNNL	HLA-B*07:02
TQKVHPNLPY	HLA-B*15:01
TSDLDFVIFY	HLA-A*01:01
TSDLDFVIFY	HLA-A*11:01
TSDLDFVIFY	HLA-A*01:01
TSNQILKPK	HLA-A*11:01
TSNQILKPK	HLA-A*30:01
TSYMIYLMNW	HLA-B*58:01
TTIDRSMGA	HLA-A*68:02
TTVKSDIKY	HLA-A*26:01
TTVKSDIKY	HLA-A*01:01
TTWIGNLDSY	HLA-A*26:01
TTWIGNLDSY	HLA-A*26:01
TVAQTVLEI	HLA-A*68:02
TVFKDNEVLY	HLA-A*26:01
TVFKDNEVLY	HLA-A*30:02
TVFKDNEVLYR	HLA-A*68:01
TVLEIITKA	HLA-A*02:06
TVLEIITKA	HLA-A*68:02
TVNPLVVNW	HLA-B*57:01
TVNPLVVNW	HLA-B*58:01
TVNPLVVNW	HLA-A*32:01
TVNPLVVNW	HLA-B*53:01
TVNPLVVNWR	HLA-A*68:01
TVNPSLISM	HLA-A*26:01
TVNPSLISM	HLA-A*68:02
TVYHCSAVY	HLA-A*30:02
TVYHCSAVY	HLA-B*35:01
TVYHCSAVY	HLA-B*15:01
TVYHCSAVY	HLA-A*30:02
TVYHCSAVY	HLA-A*26:01

TWTIATIPFLF	HLA-A*23:01
TYWSGSLMM	HLA-A*23:01
TYWSGSLMMTR	HLA-A*33:01
VAAVLQPSI	HLA-B*51:01
VEIISDIGNY	HLA-A*26:01
VFKDNEVLY	HLA-A*30:02
VFYQASFSW	HLA-A*23:01
VFYQASFSW	HLA-A*24:02
VFYQASFSW	HLA-A*32:01
VIFYASLTY	HLA-B*15:01
VIFYASLTY	HLA-B*35:01
VIFYASLTY	HLA-A*30:02
VIFYASLTY	HLA-A*03:01
VIFYASLTY	HLA-A*11:01
VIFYASLTY	HLA-A*32:01
VIFYASLTY	HLA-A*30:02
VIFYASLTY	HLA-B*15:01
VIKNSQKPK	HLA-A*30:01
VIRPKLFAV	HLA-B*08:01
VIRPKLFAV	HLA-A*02:03
VLAKTNTAL	HLA-A*02:03
VLGNVIISL	HLA-A*02:01
VLLPQMELL	HLA-A*02:01
VLLPQMELL	HLA-A*02:06
VLMGVINSI	HLA-A*02:03
VLMGVINSI	HLA-A*02:01
VLMGVINSI	HLA-A*02:06
VLMGVINSI	HLA-A*02:03
VLMGVINSI	HLA-A*02:01
VLMGVINSI	HLA-A*02:06
VLMGVINSI	HLA-A*32:01
VMMEDGLLV	HLA-A*02:01
VMMEDGLLV	HLA-A*02:01
VMMEDGLLV	HLA-A*02:06
VMMEDGLLV	HLA-A*02:03
VPAKDSPVI	HLA-B*51:01
VPATNSPEL	HLA-B*07:02
VPATNSPEL	HLA-B*35:01
VPATNSPEL	HLA-B*53:01
VPATNSPEL	HLA-B*51:01
VPATNSPELRW	HLA-B*53:01
VPATNSPELRW	HLA-B*57:01
VPATNSPELRW	HLA-B*58:01
VPDMDLLQAL	HLA-B*07:02
VPNFILVRNTL	HLA-B*07:02
VPRFALSNGVL	HLA-B*07:02
VPSLFMTNV	HLA-B*51:01
VPSLFMTNVW	HLA-B*53:01
VQQKRVNPF	HLA-B*15:01
VQQKRVNPF	HLA-B*15:01
VQQKRVNPFF	HLA-B*15:01
VRPTSSGDLYY	HLA-A*01:01
VSDAKMLSY	HLA-A*01:01
VSDAKMLSY	HLA-A*01:01
VSDAKMLSY	HLA-A*01:01
VSDAKMLSY	HLA-A*01:01
VSDAKMLSY	HLA-A*30:02
VSDAKMLSY	HLA-A*01:01
VSDAKMLSY	HLA-A*30:02

VSDAKVLSY	HLA-A*01:01
VSDAKVLSY	HLA-A*30:02
VSDFSPSSW	HLA-B*58:01
VSDFSPSSW	HLA-B*57:01
VSFNNDNSEW	HLA-B*58:01
VSFNNDNSEW	HLA-B*57:01
VSNTSKHTY	HLA-A*30:02
VSNTSKHTY	HLA-A*30:02
VSNTSKHTY	HLA-A*01:01
VSRLSHHDY	HLA-A*30:02
VTFKREMSI	HLA-A*32:01
VTGQKVQMTY	HLA-A*30:02
VTSYMIYLMNW	HLA-B*58:01
VVSSHVPRF	HLA-A*32:01
VWTPSNPNTVY	HLA-B*35:01
VYFPILTEI	HLA-A*24:02
VYFPILTEI	HLA-A*23:01
VYNNEFYYV	HLA-A*24:02
VYNNEFYYV	HLA-A*23:01
VYPREVLSY	HLA-A*24:02
VYPREVLSY	HLA-A*23:01
WAFEIHHRR	HLA-A*68:01
WAFEIHHRR	HLA-A*33:01
WAFEIHHRR	HLA-A*68:01
WAYGDNEECW	HLA-B*58:01
WESFCGIQF	HLA-B*40:01
WESFCGIQF	HLA-B*44:03
WTIATIPFL	HLA-A*02:06
WTIATIPFL	HLA-A*68:02
WTIATIPFL	HLA-A*02:01
WTIATIPFLF	HLA-B*58:01
WTPSNPNTVY	HLA-B*35:01
WTPSNPNTVY	HLA-A*01:01
WTQWLQTLY	HLA-A*01:01
WTQWLQTLY	HLA-A*30:02
WTQWLQTLY	HLA-A*01:01
YASLTYLRR	HLA-A*68:01
YATPMTNNM	HLA-B*35:01
YEDNTGTKF	HLA-B*44:02
YEDNTGTKF	HLA-B*40:01
YEDNTGTKF	HLA-B*44:03
YEKLSKIGL	HLA-B*40:01
YESMAIFAER	HLA-A*68:01
YGFTSSPERGW	HLA-B*57:01
YGFTSSPERGW	HLA-B*57:01
YGFTSSPERGW	HLA-B*57:01
YGLPGFFNW	HLA-B*58:01
YGLPGFFNW	HLA-B*58:01
YGLPGFFNW	HLA-B*57:01
YHTEFNPHNHY	HLA-B*44:03
YIINRTAGR	HLA-A*68:01
YILRSGLLK	HLA-A*03:01
YLASQRVNI	HLA-A*02:03
YLDKVEPEI	HLA-A*02:01
YLDKVEPEI	HLA-A*02:06
YLEDEQFNV	HLA-A*02:01
YLEDEQFNV	HLA-A*02:06
YLEDEQFNVSY	HLA-A*01:01
YLEPMYFRL	HLA-A*02:01

YLEPMYFRL	HLA-A*02:06
YLEPMYFRL	HLA-A*02:01
YLEPMYFRL	HLA-A*02:06
YLMQRCCPML	HLA-B*08:01
YLMQRCCPML	HLA-A*02:03
YLMQRCCPML	HLA-A*02:01
YLPSGAQRL	HLA-A*02:03
YLPSGAQRL	HLA-A*02:03
YLPSGAQRLF	HLA-B*53:01
YLRRGIIKQL	HLA-A*02:03
YLSDLLFVF	HLA-A*02:01
YLSDLLFVF	HLA-A*02:06
YLSDLLFVF	HLA-B*15:01
YLTPEMVLM	HLA-A*02:01
YLTPEMVLMY	HLA-B*15:01
YMIPRTML	HLA-B*08:01
YMIPRTMLEF	HLA-B*15:01
YMIPRTMLEF	HLA-A*23:01
YMIPRTMLEF	HLA-B*15:01
YMKDKALSPI	HLA-A*02:03
YPALALNEF	HLA-B*35:01
YPALALNEF	HLA-B*53:01
YPALALNEF	HLA-B*35:01
YPALALNEF	HLA-B*53:01
YPALALNEF	HLA-B*07:02
YPECNNILF	HLA-B*35:01
YPECNNILF	HLA-B*53:01
YPECNNILF	HLA-B*53:01
YPECNNILF	HLA-B*35:01
YPKGGIEGY	HLA-B*35:01
YPKGGIEGY	HLA-B*35:01
YPKGGIEGY	HLA-B*53:01
YPKGGIEGY	HLA-A*26:01
YPLLWSFAM	HLA-B*35:01
YPLLWSFAM	HLA-B*35:01
YPREVLSY	HLA-B*35:01
YPREVLSY	HLA-B*35:01
YPSAGTENV	HLA-B*51:01
YPVLERTRI	HLA-B*51:01
YPVLLPQM	HLA-B*51:01
YPVLLPQM	HLA-B*35:01
YQIDQPFFV	HLA-A*02:06
YQIDQPFFV	HLA-A*02:01
YQIDQPFFV	HLA-A*02:06
YQIDQPFFV	HLA-A*02:01
YQIDQPFFV	HLA-A*02:03
YQQAMLLGL	HLA-A*02:06
YQSTLGQSISF	HLA-B*15:01
YSNPDSTEVY	HLA-A*01:01
YSNPDSTEVY	HLA-B*15:01
YSNPDSTEVY	HLA-B*35:01
YSNPDSTEVY	HLA-A*01:01
YTEVDNNHLIY	HLA-A*01:01
YTEVDNNHLIY	HLA-A*01:01
YTPGFPISR	HLA-A*68:01
YTPGFPISR	HLA-A*68:01
YTPGFPISRLF	HLA-B*57:01
YVDLSSYY	HLA-A*01:01
YVQQKRVNPF	HLA-B*15:01

YYIIVRVYF HLA-A*23:01
YYIIVRVYF HLA-A*23:01
YYIIVRVYF HLA-A*24:02
YYLTPEMVL HLA-A*24:02
YYLTPEMVL HLA-A*23:01
YYQIDQPFF HLA-A*23:01
YYQIDQPFF HLA-A*24:02
YYQIDQPFF HLA-A*24:02
YYQIDQPFF HLA-A*23:01
YYQIDQPFFV HLA-A*02:06
YYQIDQPFFV HLA-A*02:06

AEGSDDIQLDPVVTD HLA-DRB1*03:01
AEGSDDIQLDPVVTD HLA-DRB1*03:01
AEGSDDIQLDPVVTD HLA-DRB1*03:01
AEKTVYVLTALQDYI HLA-DQA1*05:01/DQB1*02:01
AELAAAVQETSAGRQ HLA-DQA1*04:01/DQB1*04:02
AFGGNYETLLRTLGY HLA-DRB1*11:01
AFLIDRINWISAGVF HLA-DRB3*01:01
AGGIDQNMANRLGLS HLA-DRB1*13:02
AGVAIGIATAAQITA HLA-DRB1*08:02
AGVIMAGVAIGIATA HLA-DQA1*05:01/DQB1*03:01
AKMLSYAPEIAVSKE HLA-DRB1*09:01
AKMLSYAPEIAVSKE HLA-DRB1*09:01
ALIRSLLNDPDIEAV HLA-DRB3*01:01
ALIRSLLNDPDIEAV HLA-DRB1*04:05
ALLGSIVIIVMNIMI HLA-DRB4*01:01
ALLGSIVIIVMNIMI HLA-DRB1*15:01
ALRIFFLSITKLNDS HLA-DPA1*01/DPB1*04:01
ALRIFFLSITKLNDS HLA-DPA1*02:01/DPB1*14:01
ALSKYLSDLLFVFGP HLA-DRB3*01:01
AMDEGYFAYSHLERI HLA-DPA1*03:01/DPB1*04:02
AMDEGYFAYSHLERI HLA-DPA1*02:01/DPB1*01:01
AMDEGYFAYSHLERI HLA-DPA1*01:03/DPB1*02:01
AQPPYHWSIERSISP HLA-DRB1*09:01
ASFLMDRRVILPRAA HLA-DRB3*01:01
ASFLMDRRVILPRAA HLA-DRB1*03:01
ASFSWDTMIKFGDVL HLA-DRB3*01:01
ASILLTLFRRTKKKY HLA-DPA1*03:01/DPB1*04:02
ASILLTLFRRTKKKY HLA-DRB1*11:01
ASLCIGLITFISFII HLA-DRB1*15:01
ASLCIGLITFISFII HLA-DPA1*01:03/DPB1*02:01
ASQRVNIDLDVLKAI HLA-DRB3*01:01
AVEIISDIGNYVEET HLA-DRB3*01:01
AVEIISDIGNYVEET HLA-DRB1*13:02
AVIIDVGSMVNGIPV HLA-DRB1*03:01
AVIKDALQGIQQQIK HLA-DRB4*01:01
AYSHLERIGSCSRGV HLA-DRB1*11:01
CFSLMDINPWLNRLT HLA-DRB3*01:01
CIGLITFISFIIVEK HLA-DPA1*01:03/DPB1*02:01
CIGLITFISFIIVEK HLA-DRB1*15:01
CINILKVIQQLLIST HLA-DRB4*01:01
CLNWRYESMAIFAER HLA-DRB3*01:01
CLRMMEMLKEETWRI HLA-DRB3*01:01
CLVSDAKMLSYAPEI HLA-DRB1*03:01
CLVSDAKMLSYAPEI HLA-DRB1*03:01
CLVSDAKMLSYAPEI HLA-DRB1*03:01

CNLLILILMISECSV HLA-DRB1*11:01
CNLLILILMISECSV HLA-DRB1*04:05
CPMLPKLQFLKKIGK HLA-DRB1*11:01
CPMLPKLQFLKKIGK HLA-DRB5*01:01
CPRELVVSSHVPRFA HLA-DRB1*03:01
CSAVYNNEFYYVLCA HLA-DPA1*01/DPB1*04:01
CWEGVYNDAFLIDRI HLA-DRB3*01:01
CWEGVYNDAFLIDRI HLA-DPA1*01:03/DPB1*02:01
CYCNLLILILMISEC HLA-DPA1*03:01/DPB1*04:02
CYCNLLILILMISEC HLA-DRB1*11:01
DAFLIDRINWISAGV HLA-DRB3*01:01
DAFLIDRINWISAGV HLA-DRB1*03:01
DAKMLSYAPEIAVSK HLA-DRB1*09:01
DAKMLSYAPEIAVSK HLA-DRB1*09:01
DALQGIQQQIKGLAD HLA-DRB4*01:01
DDIQLDPVVTDVVYH HLA-DRB1*03:01
DDIQLDPVVTDVVYH HLA-DRB1*03:01
DDIQLDPVVTDVVYH HLA-DRB1*03:01
DDLKKYYQIDQPFFV HLA-DRB3*01:01
DDQVFNNPASKIKQK HLA-DRB1*13:02
DEGYFAYSHLERIGS HLA-DPA1*03:01/DPB1*04:02
DEGYFAYSHLERIGS HLA-DPA1*01:03/DPB1*02:01
DEGYFAYSHLERIGS HLA-DPA1*02:01/DPB1*01:01
DEGYFAYSHLERIGS HLA-DRB1*11:01
DEGYFAYSHLERIGS HLA-DPA1*02:01/DPB1*14:01
DEGYFAYSHLERIGS HLA-DPA1*01/DPB1*04:01
DGKRAWVEEWCNPAC HLA-DQA1*01:01/DQB1*05:01
DIEAVIIDVGSMVNG HLA-DRB1*03:01
DIQLDPVVTDVVYHD HLA-DRB3*01:01
DIQLDPVVTDVVYHD HLA-DRB1*03:01
DIQLDPVVTDVVYHD HLA-DRB3*01:01
DIQLDPVVTDVVYHD HLA-DRB1*03:01
DIQLDPVVTDVVYHD HLA-DRB3*01:01
DIQLDPVVTDVVYHD HLA-DRB1*03:01
DIVIKMIPNVSNMSQ HLA-DRB1*08:02
DKHQALRIFFLSITK HLA-DPA1*01/DPB1*04:01
DKHQALRIFFLSITK HLA-DPA1*01:03/DPB1*02:01
DKLELVNDGLNIIDF HLA-DRB1*03:01
DKLELVNDGLNIIDF HLA-DRB3*01:01
DKLELVNDGLNIIDF HLA-DRB1*03:01
DKRCYCNLLILILMI HLA-DPA1*03:01/DPB1*04:02
DLALSKYLSDLLFVF HLA-DRB3*01:01
DLELASFLMDRRVIL HLA-DRB3*01:01
DLELASFLMDRRVIL HLA-DRB1*03:01
DLKKYYQIDQPFFVP HLA-DRB3*01:01
DLSMYMKDKALSPIK HLA-DRB3*01:01
DLSSYYIIVRVYFPI HLA-DRB3*01:01
DLSSYYIIVRVYFPI HLA-DPA1*02:01/DPB1*01:01
DLVGDVRLAGVIMAG HLA-DRB1*03:01
DMSTLVSAVITIEAQ HLA-DQA1*04:01/DQB1*04:02
DNIHLLAEFFSFFRT HLA-DPA1*01:03/DPB1*02:01
DNSEWISIVPNFILV HLA-DRB5*01:01
DPDIEAVIIDVGSMV HLA-DRB1*03:01
DPVSNSMTIQAISQA HLA-DQA1*01:02/DQB1*06:02
DPVVTDVVYHDHGGE HLA-DRB3*01:01
DPVVTDVVYHDHGGE HLA-DRB3*01:01
DPVVTDVVYHDHGGE HLA-DRB1*03:01
DPVVTDVVYHDHGGE HLA-DRB3*01:01
DPVVTDVVYHDHGGE HLA-DRB1*03:01

DQKIRSMFIDDLLSI HLA-DRB3*01:01
DQVAELAAAVQETSA HLA-DQA1*04:01/DQB1*04:02
DQVAELAAAVQETSA HLA-DQA1*03:01/DQB1*03:02
DQVFNNPASKIKQKP HLA-DRB1*13:02
DSDLSMYMKDKALSP HLA-DRB3*01:01
DSGIYMIPRTMLEFR HLA-DRB1*11:01
DSIKLYTSDDEEADQ HLA-DRB3*01:01
DSIKLYTSDDEEADQ HLA-DRB3*01:01
DSIKLYTSDDEEADQ HLA-DRB3*01:01
DSKILSAFNTVIALL HLA-DPA1*03:01/DPB1*04:02
DTIIIMLNEAMNYFD HLA-DRB3*01:01
DTVNPSLISMLSMII HLA-DRB1*15:01
DYNQFLILNKLLSNR HLA-DRB1*11:01
EAVIIDVGSMVNGIP HLA-DRB1*03:01
EETWRIYPVLLPQME HLA-DRB1*08:02
EFEDEFAGSSSEVIV HLA-DRB1*07:01
EFEDEFAGSSSEVIV HLA-DRB1*07:01
EFMIYDDVFIDNTGR HLA-DRB3*01:01
EFQSDLNTIKSLMLL HLA-DRB1*07:01
EFRRNNAIAFNLLVY HLA-DRB3*02:02
EGSDDIQLDPVVTDV HLA-DRB1*03:01
EGSDDIQLDPVVTDV HLA-DRB1*03:01
EGSDDIQLDPVVTDV HLA-DRB1*03:01
EGVYNDAFLIDRINW HLA-DPA1*01:03/DPB1*02:01
EGYFAYSHLERIGSC HLA-DPA1*03:01/DPB1*04:02
EGYFAYSHLERIGSC HLA-DPA1*01:03/DPB1*02:01
EGYFAYSHLERIGSC HLA-DRB1*11:01
EGYFAYSHLERIGSC HLA-DPA1*02:01/DPB1*01:01
EGYFAYSHLERIGSC HLA-DPA1*01/DPB1*04:01
EGYFAYSHLERIGSC HLA-DPA1*02:01/DPB1*14:01
EICWEGVYNDAFLID HLA-DRB3*01:01
EICWEGVYNDAFLID HLA-DPA1*01:03/DPB1*02:01
EIGFCLITKRSVICN HLA-DRB1*11:01
EKKRNTYSRLEDRRV HLA-DRB1*09:01
EKTVYVLTALQDYIN HLA-DQA1*05:01/DQB1*02:01
ELASFLMDRRVILPR HLA-DRB3*01:01
ELASFLMDRRVILPR HLA-DRB1*03:01
ELRWELTLFALDVIR HLA-DQA1*01:01/DQB1*05:01
ELRWELTLFALDVIR HLA-DPA1*01/DPB1*04:01
ELTLFALDVIRSPSA HLA-DQA1*01:01/DQB1*05:01
ELTLFALDVIRSPSA HLA-DRB1*11:01
ELVVSSHVPRFALSN HLA-DRB1*03:01
EMLKEETWRIYPVLL HLA-DRB3*01:01
EQQSLFSFDNVKNFR HLA-DRB3*01:01
ERPGALIRSLLNDPD HLA-DRB1*04:05
ETLTLDVTSPISNNL HLA-DRB3*01:01
ETWRIYPVLLPQMEL HLA-DRB1*08:02
FAYSHLERIGSCSRG HLA-DRB1*11:01
FCLNWRYESMAIFAE HLA-DRB3*01:01
FGGNYETLLRTLGYA HLA-DRB1*11:01
FISFIIVEKKRNTYS HLA-DRB1*11:01
FLIDRINWISAGVFL HLA-DRB3*01:01
FLILNKLLSNRRQND HLA-DRB1*11:01
FMIYDDVFIDNTGRI HLA-DRB3*01:01
FMIYDDVFIDNTGRI HLA-DRB1*03:01
FQSDLNTIKSLMLLY HLA-DRB1*07:01
FRRTKKKYRRHTDDQ HLA-DRB5*01:01
FSFDNVKNFRDGSLT HLA-DRB3*01:01
FSINETLTLDVTSPI HLA-DRB3*01:01

FSWDTMIKFGDVLTV HLA-DRB3*01:01
FVIFYASLTYLRRGI HLA-DPA1*02:01/DPB1*14:01
FYQASFSWDTMIKFG HLA-DRB3*01:01
GALIRSLLNDPDIEA HLA-DRB3*01:01
GALIRSLLNDPDIEA HLA-DRB1*04:05
GGIDQNMANRLGLSS HLA-DRB1*13:02
GGNYETLLRTLGYAT HLA-DRB1*11:01
GGYPLLWSFAMGVAT HLA-DRB1*09:01
GHLVSMMIMIPGKGK HLA-DRB5*01:01
GIPIKKAQTRNIHLL HLA-DRB1*07:01
GKRKKIRTIAAYPLG HLA-DRB1*08:02
GLITFISFIIVEKKR HLA-DPA1*01:03/DPB1*02:01
GLRITDMSTLVSAVI HLA-DQA1*04:01/DQB1*04:02
GLSSDQVAELAAAVQ HLA-DQA1*04:01/DQB1*04:02
GMAGFFATIRFGLET HLA-DPA1*01/DPB1*04:01
GNFVRRAGKYYSVDY HLA-DRB1*11:01
GNVCLVSDAKMLSYA HLA-DRB1*03:01
GNVCLVSDAKMLSYA HLA-DRB1*03:01
GNVCLVSDAKMLSYA HLA-DRB1*11:01
GNVCLVSDAKMLSYA HLA-DRB1*03:01
GNYETLLRTLGYATE HLA-DRB1*11:01
GQIIYVDLSSYYIIV HLA-DRB1*15:01
GQIIYVDLSSYYIIV HLA-DRB3*01:01
GRAVEIISDIGNYVE HLA-DRB3*01:01
GSDDIQLDPVVTDVV HLA-DRB1*03:01
GSDDIQLDPVVTDVV HLA-DRB1*03:01
GSDDIQLDPVVTDVV HLA-DRB1*03:01
GSIVIIVMNIMIIQN HLA-DRB4*01:01
GSIVIIVMNIMIIQN HLA-DRB1*15:01
GSSSEVIVGISPEDE HLA-DQA1*04:01/DQB1*04:02
GVAIGIATAAQITAG HLA-DRB1*08:02
GVIMAGVAIGIATAA HLA-DQA1*05:01/DQB1*03:01
GVINSIKLINLDMRL HLA-DRB4*01:01
GVYNDAFLIDRINWI HLA-DRB3*01:01
GVYNDAFLIDRINWI HLA-DRB1*03:01
GYCINILKVIQQLLI HLA-DRB4*01:01
GYFAYSHLERIGSCS HLA-DPA1*03:01/DPB1*04:02
GYFAYSHLERIGSCS HLA-DPA1*01:03/DPB1*02:01
GYFAYSHLERIGSCS HLA-DRB1*11:01
GYPLLWSFAMGVATT HLA-DRB1*09:01
HAGGIDQNMANRLGL HLA-DRB1*13:02
HCSAVYNNEFYYVLC HLA-DPA1*01/DPB1*04:01
HDLVGDVRLAGVIMA HLA-DRB1*03:01
HDYNQFLILNKLLSN HLA-DRB1*11:01
HHAGGIDQNMANRLG HLA-DRB1*13:02
HHDYNQFLILNKLLS HLA-DRB1*11:01
HHKPPWIIDLNPQEK HLA-DRB3*01:01
HIKINGVISKRLFAQ HLA-DRB1*11:01
HKPPWIIDLNPQEKI HLA-DRB3*01:01
HLGNFVRRAGKYYSV HLA-DRB1*11:01
HLVPWKKVLTSGSIF HLA-DRB1*08:02
HLVSMMIMIPGKGKG HLA-DRB5*01:01
HQALRIFFLSITKLN HLA-DPA1*01/DPB1*04:01
HQALRIFFLSITKLN HLA-DPA1*01:03/DPB1*02:01
HQALRIFFLSITKLN HLA-DPA1*02:01/DPB1*14:01
HTDDQVFNNPASKIK HLA-DRB1*13:02
IALLGSIVIIVMNIM HLA-DRB4*01:01
IALLGSIVIIVMNIM HLA-DRB1*15:01
ICWEGVYNDAFLIDR HLA-DRB3*01:01

ICWEGVYNDAFLIDR HLA-DPA1*01:03/DPB1*02:01
IDNIHLLAEFFSFFR HLA-DPA1*01:03/DPB1*02:01
IEAVIIDVGSMVNGI HLA-DRB1*03:01
IEGHLVSMMIMIPGK HLA-DRB4*01:01
IGLITFISFIIVEKK HLA-DPA1*01:03/DPB1*02:01
IGLITFISFIIVEKK HLA-DRB1*15:01
IHLLAEFFSFFRTFG HLA-DPA1*01:03/DPB1*02:01
IIIMLNEAMNYFDDN HLA-DRB3*01:01
IIVMNIMIIQNYTRS HLA-DRB4*01:01
IIVMNIMIIQNYTRS HLA-DRB5*01:01
IIVMNIMIIQNYTRS HLA-DRB1*15:01
IIVRVYFPILTEIQQ HLA-DPA1*02:01/DPB1*01:01
IIVRVYFPILTEIQQ HLA-DPA1*01:03/DPB1*02:01
IIVRVYFPILTEIQQ HLA-DQA1*01:01/DQB1*05:01
IKDALQGIQQQIKGL HLA-DRB4*01:01
IKINGVISKRLFAQM HLA-DRB1*11:01
IKLINLDMRLNHIEE HLA-DRB1*03:01
IKLYTSDDEEADQLE HLA-DRB3*01:01
IKLYTSDDEEADQLE HLA-DRB3*01:01
IKLYTSDDEEADQLE HLA-DRB3*01:01
IKMIPNVSNMSQCTG HLA-DRB1*08:02
IKSNPLTKDIVIKMI HLA-DRB1*03:01
ILDKRCYCNLLILIL HLA-DPA1*03:01/DPB1*04:02
ILKVIQQLLISTEFS HLA-DRB4*01:01
ILLTLFRRTKKKYRR HLA-DRB5*01:01
ILLTLFRRTKKKYRR HLA-DRB1*11:01
ILSAFNTVIALLGSI HLA-DRB1*07:01
ILSAFNTVIALLGSI HLA-DPA1*03:01/DPB1*04:02
INETLTLDVTSPISN HLA-DRB3*01:01
INILKVIQQLLISTE HLA-DRB4*01:01
INLDMRLNHIEEQVK HLA-DRB1*03:01
INSIKLINLDMRLNH HLA-DRB1*03:01
IPREFMIYDDVFIDN HLA-DRB3*01:01
IPREFMIYDDVFIDN HLA-DQA1*05:01/DQB1*02:01
IPREFMIYDDVFIDN HLA-DQA1*01:01/DQB1*05:01
IQLDPVVTDVVYHDH HLA-DRB3*01:01
IQLDPVVTDVVYHDH HLA-DRB1*03:01
IQLDPVVTDVVYHDH HLA-DRB3*01:01
IQLDPVVTDVVYHDH HLA-DRB1*03:01
IQLDPVVTDVVYHDH HLA-DRB3*01:01
IQLDPVVTDVVYHDH HLA-DRB1*03:01
IRIFVPATNSPELRW HLA-DRB1*04:05
IRSLLNDPDIEAVII HLA-DRB3*01:01
IRSMFIDDLLSILNI HLA-DRB3*01:01
ISFIIVEKKRNTYSR HLA-DRB1*11:01
ISIVPNFILVRNTLI HLA-DRB1*07:01
ISIVPNFILVRNTLI HLA-DRB1*01:01
ISIVPNFILVRNTLI HLA-DRB1*04:05
ISMLSMIILYVLSIA HLA-DRB4*01:01
ISMLSMIILYVLSIA HLA-DRB1*11:01
ITDMSTLVSAVITIE HLA-DQA1*04:01/DQB1*04:02
ITGQIIYVDLSSYYI HLA-DRB3*01:01
ITNCFLLKNKIWCIS HLA-DRB1*11:01
IVIIVMNIMIIQNYT HLA-DRB4*01:01
IVIKMIPNVSNMSQC HLA-DRB1*08:02
IVMNIMIIQNYTRST HLA-DRB4*01:01
IVMNIMIIQNYTRST HLA-DRB5*01:01
IVMNIMIIQNYTRST HLA-DRB1*15:01
IVPNFILVRNTLISN HLA-DRB1*01:01

IVPNFILVRNTLISN HLA-DRB1*07:01
IVPNFILVRNTLISN HLA-DRB1*04:05
IVPNFILVRNTLISN HLA-DRB1*08:02
IVRVYFPILTEIQQA HLA-DPA1*02:01/DPB1*01:01
IVRVYFPILTEIQQA HLA-DQA1*01:01/DQB1*05:01
IVRVYFPILTEIQQA HLA-DPA1*01:03/DPB1*02:01
IYDDVFIDNTGRILK HLA-DRB3*01:01
IYDDVFIDNTGRILK HLA-DRB1*03:01
IYPVLLPQMELLERE HLA-DPA1*03:01/DPB1*04:02
KCPRELVVSSHVPRF HLA-DRB1*03:01
KDALQGIQQQIKGLA HLA-DRB4*01:01
KDIVIKMIPNVSNMS HLA-DRB1*08:02
KEETWRIYPVLLPQM HLA-DRB1*08:02
KGRAVEIISDIGNYV HLA-DRB3*01:01
KHQALRIFFLSITKL HLA-DPA1*01/DPB1*04:01
KHQALRIFFLSITKL HLA-DPA1*01:03/DPB1*02:01
KIKSNPLTKDIVIKM HLA-DRB1*03:01
KILSAFNTVIALLGS HLA-DPA1*03:01/DPB1*04:02
KILSAFNTVIALLGS HLA-DRB1*07:01
KIRIFVPATNSPELR HLA-DRB1*04:05
KIRSMFIDDLLSILN HLA-DRB3*01:01
KKIRTIAAYPLGVGK HLA-DRB1*08:02
KKYYQIDQPFFVPTK HLA-DRB3*01:01
KLELVNDGLNIIDFI HLA-DRB1*03:01
KLELVNDGLNIIDFI HLA-DRB3*01:01
KLELVNDGLNIIDFI HLA-DRB1*03:01
KLINLDMRLNHIEEQ HLA-DRB1*03:01
KLYTSDDEEADQLEF HLA-DRB3*01:01
KLYTSDDEEADQLEF HLA-DRB3*01:01
KLYTSDDEEADQLEF HLA-DRB3*01:01
KMLSYAPEIAVSKED HLA-DRB1*09:01
KNLCFSLMDINPWLN HLA-DRB3*01:01
KNNTHDLVGDVRLAG HLA-DRB1*03:01
KPPWIIDLNPQEKIC HLA-DRB3*01:01
KQTELSLDLALSKYL HLA-DRB1*03:01
KRCYCNLLILILMIS HLA-DPA1*03:01/DPB1*04:02
KRCYCNLLILILMIS HLA-DRB1*11:01
KRKKIRTIAAYPLGV HLA-DRB1*08:02
KRKKIRTIAAYPLGV HLA-DRB5*01:01
KSNPLTKDIVIKMIP HLA-DRB1*03:01
KTITNCFLLKNKIWC HLA-DRB1*11:01
KTVYVLTALQDYINT HLA-DQA1*05:01/DQB1*02:01
KVQMTYNWTQWLQTL HLA-DPA1*01:03/DPB1*02:01
KVQMTYNWTQWLQTL HLA-DPA1*01/DPB1*04:01
KYLSDLLFVFGPNLQ HLA-DRB3*01:01
KYYQIDQPFFVPTKI HLA-DRB3*01:01
LAGVIMAGVAIGIAT HLA-DQA1*05:01/DQB1*03:01
LALSKYLSDLLFVFG HLA-DRB3*01:01
LASFLMDRRVILPRA HLA-DRB3*01:01
LASFLMDRRVILPRA HLA-DRB1*03:01
LCFSLMDINPWLNRL HLA-DRB3*01:01
LCIGLITFISFIIVE HLA-DPA1*01:03/DPB1*02:01
LCIGLITFISFIIVE HLA-DRB1*15:01
LDFVIFYASLTYLRR HLA-DPA1*02:01/DPB1*14:01
LDKHQALRIFFLSIT HLA-DPA1*01/DPB1*04:01
LDKHQALRIFFLSIT HLA-DPA1*01:03/DPB1*02:01
LDKRCYCNLLILILM HLA-DPA1*03:01/DPB1*04:02
LDPVVTDVVYHDHGG HLA-DRB3*01:01
LDPVVTDVVYHDHGG HLA-DRB3*01:01

LDPVVTDVVYHDHGG HLA-DRB1*03:01
LDPVVTDVVYHDHGG HLA-DRB3*01:01
LDPVVTDVVYHDHGG HLA-DRB1*03:01
LDSIKLYTSDDEEAD HLA-DRB3*01:01
LDSIKLYTSDDEEAD HLA-DRB3*01:01
LDSIKLYTSDDEEAD HLA-DRB3*01:01
LEFEDEFAGSSSEVI HLA-DRB1*07:01
LEFEDEFAGSSSEVI HLA-DRB1*07:01
LEFEDEFAGSSSEVI HLA-DRB1*07:01
LEFRRNNAIAFNLLV HLA-DRB3*02:02
LEFRRNNAIAFNLLV HLA-DRB3*01:01
LEFRRNNAIAFNLLV HLA-DRB1*13:02
LELASFLMDRRVILP HLA-DRB3*01:01
LELASFLMDRRVILP HLA-DRB1*03:01
LELVNDGLNIIDFIQ HLA-DRB1*03:01
LELVNDGLNIIDFIQ HLA-DRB1*03:01
LEQQSLFSFDNVKNF HLA-DRB3*01:01
LFRRTKKKYRRHTDD HLA-DRB5*01:01
LFSFDNVKNFRDGSL HLA-DRB3*01:01
LGLSSDQVAELAAAV HLA-DQA1*04:01/DQB1*04:02
LGNFVRRAGKYYSVD HLA-DRB1*11:01
LGSIVIIVMNIMIIQ HLA-DRB4*01:01
LGSIVIIVMNIMIIQ HLA-DRB1*15:01
LHIKINGVISKRLFA HLA-DRB1*07:01
LHIKINGVISKRLFA HLA-DRB1*11:01
LHIKINGVISKRLFA HLA-DRB3*02:02
LHLGNFVRRAGKYYS HLA-DRB1*11:01
LINLDMRLNHIEEQV HLA-DRB1*03:01
LIRSLLNDPDIEAVI HLA-DRB3*01:01
LISMLSMIILYVLSI HLA-DRB4*01:01
LISMLSMIILYVLSI HLA-DRB1*11:01
LITFISFIIVEKKRN HLA-DPA1*01:03/DPB1*02:01
LKKYYQIDQPFFVPT HLA-DRB3*01:01
LLGSIVIIVMNIMII HLA-DRB4*01:01
LLGSIVIIVMNIMII HLA-DRB1*15:01
LLILILMISECSVGI HLA-DRB1*11:01
LLPVSFNNDNSEWIS HLA-DRB3*01:01
LLTLFRRTKKKYRRH HLA-DRB5*01:01
LLTLFRRTKKKYRRH HLA-DRB1*11:01
LLWSFAMGVATTIDR HLA-DRB1*09:01
LNHLVPWKKVLTSGS HLA-DRB1*08:02
LNWRYESMAIFAERL HLA-DRB3*01:01
LPNNICLQKTSNQIL HLA-DRB1*07:01
LPVSFNNDNSEWISI HLA-DRB3*01:01
LQDPVSNSMTIQAIS HLA-DQA1*01:02/DQB1*06:02
LQTLYTMIMEENVPD HLA-DQA1*04:01/DQB1*04:02
LRIFFLSITKLNDSG HLA-DPA1*02:01/DPB1*14:01
LRITDMSTLVSAVIT HLA-DQA1*04:01/DQB1*04:02
LRMMEMLKEETWRIY HLA-DRB3*01:01
LRTLGYATEDFDDLL HLA-DQA1*05:01/DQB1*02:01
LRWELTLFALDVIRS HLA-DQA1*01:01/DQB1*05:01
LRWELTLFALDVIRS HLA-DPA1*01/DPB1*04:01
LSAFNTVIALLGSIV HLA-DRB1*07:01
LSAFNTVIALLGSIV HLA-DPA1*03:01/DPB1*04:02
LSKYLSDLLFVFGPN HLA-DRB3*01:01
LSLHIKINGVISKRL HLA-DRB1*07:01
LSMIILYVLSIASLC HLA-DRB1*11:01
LSMIILYVLSIASLC HLA-DRB1*15:01
LSMIILYVLSIASLC HLA-DRB1*04:01

LSMYMKDKALSPIKD HLA-DRB3*01:01
LSSDQVAELAAAVQE HLA-DQA1*04:01/DQB1*04:02
LSSDQVAELAAAVQE HLA-DQA1*03:01/DQB1*03:02
LSSYYIIVRVYFPIL HLA-DPA1*01:03/DPB1*02:01
LSSYYIIVRVYFPIL HLA-DRB3*01:01
LSSYYIIVRVYFPIL HLA-DPA1*01/DPB1*04:01
LSSYYIIVRVYFPIL HLA-DPA1*02:01/DPB1*01:01
LTEIQQAYIQELLPV HLA-DQA1*05:01/DQB1*02:01
LTEMRNLLSQSLSVR HLA-DRB4*01:01
LTKDIVIKMIPNVSN HLA-DRB1*08:02
LTKDIVIKMIPNVSN HLA-DRB1*03:01
LTLFALDVIRSPSAA HLA-DQA1*01:01/DQB1*05:01
LTLFALDVIRSPSAA HLA-DRB1*11:01
LTLFRRTKKKYRRHT HLA-DRB5*01:01
LTLFRRTKKKYRRHT HLA-DRB1*11:01
LTNEPYGAAVQLRED HLA-DQA1*04:01/DQB1*04:02
LTNSLLNLRSRLAAK HLA-DRB1*15:01
LTTKIRIFVPATNSP HLA-DRB1*08:02
LVGDVRLAGVIMAGV HLA-DRB1*03:01
LVSAVITIEAQIWIL HLA-DQA1*03:01/DQB1*03:02
LVSDAKMLSYAPEIA HLA-DRB1*03:01
LVSDAKMLSYAPEIA HLA-DRB1*03:01
LVSDAKMLSYAPEIA HLA-DRB1*03:01
LVSMMIMIPGKGKGE HLA-DRB5*01:01
LVVSSHVPRFALSNG HLA-DRB1*03:01
LWSFAMGVATTIDRS HLA-DRB1*09:01
LYTSDDEEADQLEFE HLA-DQA1*03:01/DQB1*03:02
MAGFFATIRFGLETR HLA-DPA1*01/DPB1*04:01
MASILLTLFRRTKKK HLA-DPA1*03:01/DPB1*04:02
MASILLTLFRRTKKK HLA-DPA1*01:03/DPB1*02:01
MDEGYFAYSHLERIG HLA-DPA1*03:01/DPB1*04:02
MDEGYFAYSHLERIG HLA-DPA1*01:03/DPB1*02:01
MDEGYFAYSHLERIG HLA-DPA1*02:01/DPB1*01:01
MDKLELVNDGLNIID HLA-DRB1*03:01
MDKLELVNDGLNIID HLA-DRB1*03:01
MEMLKEETWRIYPVL HLA-DRB3*01:01
MFIDDLLSILNIDNI HLA-DRB3*01:01
MIILYVLSIASLCIG HLA-DRB1*11:01
MIILYVLSIASLCIG HLA-DRB1*15:01
MIILYVLSIASLCIG HLA-DRB1*04:01
MIYDDVFIDNTGRIL HLA-DRB3*01:01
MIYDDVFIDNTGRIL HLA-DRB1*03:01
MLEFRRNNAIAFNLL HLA-DRB3*02:02
MLEFRRNNAIAFNLL HLA-DRB3*01:01
MLEFRRNNAIAFNLL HLA-DRB1*13:02
MLKEETWRIYPVLLP HLA-DRB3*01:01
MLPKLQFLKKIGKLI HLA-DRB1*11:01
MLPKLQFLKKIGKLI HLA-DRB5*01:01
MLSMIILYVLSIASL HLA-DRB1*11:01
MLSMIILYVLSIASL HLA-DRB1*15:01
MLSMIILYVLSIASL HLA-DRB1*04:01
MLSMIILYVLSIASL HLA-DPA1*02:01/DPB1*01:01
MMASILLTLFRRTKK HLA-DPA1*03:01/DPB1*04:02
MMASILLTLFRRTKK HLA-DPA1*01:03/DPB1*02:01
MMEMLKEETWRIYPV HLA-DRB3*01:01
MNIMIIQNYTRSTDN HLA-DRB5*01:01
MPSDDFSNTFFPHDT HLA-DPA1*01/DPB1*04:01
MSTLVSAVITIEAQI HLA-DQA1*03:01/DQB1*03:02
MTYNWTQWLQTLYTM HLA-DPA1*01:03/DPB1*02:01

MVVILDKRCYCNLLI HLA-DRB1*03:01
MVVILDKRCYCNLLI HLA-DRB1*11:01
MYLICYGFVEDVERT HLA-DQA1*01:01/DQB1*05:01
NDAFLIDRINWISAG HLA-DRB3*01:01
NDAFLIDRINWISAG HLA-DRB1*03:01
NDNSEWISIVPNFIL HLA-DRB5*01:01
NEFQSDLNTIKSLML HLA-DRB1*07:01
NEFQSDLNTIKSLML HLA-DRB1*04:01
NEPYGAAVQLREDLI HLA-DQA1*04:01/DQB1*04:02
NETLTLDVTSPISNN HLA-DRB3*01:01
NFILVRNTLISNIEI HLA-DRB1*08:02
NFVRRAGKYYSVDYC HLA-DRB1*11:01
NGNVCLVSDAKMLSY HLA-DRB1*03:01
NGNVCLVSDAKMLSY HLA-DRB1*03:01
NGNVCLVSDAKMLSY HLA-DRB1*11:01
NGNVCLVSDAKMLSY HLA-DRB1*03:01
NHHKPPWIIDLNPQE HLA-DRB3*01:01
NHLVPWKKVLTSGSI HLA-DRB1*08:02
NIDNIHLLAEFFSFF HLA-DPA1*01:03/DPB1*02:01
NIHLLAEFFSFFRTF HLA-DPA1*01:03/DPB1*02:01
NILKVIQQLLISTEF HLA-DRB4*01:01
NLCFSLMDINPWLNR HLA-DRB3*01:01
NLLILILMISECSVG HLA-DRB1*11:01
NLLILILMISECSVG HLA-DRB1*04:05
NLQDPVSNSMTIQAI HLA-DQA1*01:02/DQB1*06:02
NNDNSEWISIVPNFI HLA-DRB5*01:01
NNGNVCLVSDAKMLS HLA-DRB1*03:01
NNGNVCLVSDAKMLS HLA-DRB1*03:01
NNGNVCLVSDAKMLS HLA-DRB1*11:01
NNGNVCLVSDAKMLS HLA-DRB1*03:01
NNTHDLVGDVRLAGV HLA-DRB1*03:01
NPLTKDIVIKMIPNV HLA-DRB1*03:01
NPSLISMLSMIILYV HLA-DRB4*01:01
NPSLISMLSMIILYV HLA-DRB1*15:01
NQFLILNKLLSNRRQ HLA-DRB1*11:01
NSEWISIVPNFILVR HLA-DRB5*01:01
NSIKLINLDMRLNHI HLA-DRB1*03:01
NSLLNLRSRLAAKAA HLA-DRB1*15:01
NSMTIQAISQAFGGN HLA-DRB1*08:02
NTHDLVGDVRLAGVI HLA-DRB1*03:01
NVCLVSDAKMLSYAP HLA-DRB1*03:01
NVCLVSDAKMLSYAP HLA-DRB1*03:01
NVCLVSDAKMLSYAP HLA-DRB1*11:01
NVCLVSDAKMLSYAP HLA-DRB1*03:01
NVNLDSIKLYTSDDE HLA-DRB1*03:01
NWRYESMAIFAERLD HLA-DRB3*01:01
NYMYLICYGFVEDVE HLA-DQA1*01:01/DQB1*05:01
PDIEAVIIDVGSMVN HLA-DRB1*03:01
PEICWEGVYNDAFLI HLA-DRB3*01:01
PELRWELTLFALDVI HLA-DQA1*01:01/DQB1*05:01
PELRWELTLFALDVI HLA-DPA1*01/DPB1*04:01
PGALIRSLLNDPDIE HLA-DRB1*04:05
PLLWSFAMGVATTID HLA-DRB1*09:01
PLTKDIVIKMIPNVS HLA-DRB1*03:01
PMLPKLQFLKKIGKL HLA-DRB1*11:01
PMLPKLQFLKKIGKL HLA-DRB5*01:01
PNFILVRNTLISNIE HLA-DRB1*01:01
PNFILVRNTLISNIE HLA-DRB1*08:02
PNFILVRNTLISNIE HLA-DRB1*04:05

PPWIIDLNPQEKICV HLA-DRB3*01:01
PPYHWSIERSISPDK HLA-DRB1*09:01
PQTSRNVNLDSIKLY HLA-DRB1*03:01
PREFMIYDDVFIDNT HLA-DRB3*01:01
PREFMIYDDVFIDNT HLA-DQA1*01:01/DQB1*05:01
PREFMIYDDVFIDNT HLA-DQA1*05:01/DQB1*02:01
PRELVVSSHVPRFAL HLA-DRB1*03:01
PRTMLEFRRNNAIAF HLA-DRB3*01:01
PRTMLEFRRNNAIAF HLA-DRB1*13:02
PSDDFSNTFFPHDTD HLA-DPA1*01/DPB1*04:01
PSIPREFMIYDDVFI HLA-DRB3*01:01
PSIPREFMIYDDVFI HLA-DQA1*05:01/DQB1*02:01
PSLISMLSMIILYVL HLA-DRB4*01:01
PSLISMLSMIILYVL HLA-DRB1*15:01
PVSFNNDNSEWISIV HLA-DRB3*01:01
PVSNSMTIQAISQAF HLA-DQA1*01:02/DQB1*06:02
PVVTDVVYHDHGGEC HLA-DRB3*01:01
PVVTDVVYHDHGGEC HLA-DRB3*01:01
PVVTDVVYHDHGGEC HLA-DRB3*01:01
QALRIFFLSITKLND HLA-DPA1*01/DPB1*04:01
QALRIFFLSITKLND HLA-DPA1*02:01/DPB1*14:01
QASFSWDTMIKFGDV HLA-DRB3*01:01
QDPVSNSMTIQAISQ HLA-DQA1*01:02/DQB1*06:02
QFLILNKLLSNRRQN HLA-DRB1*11:01
QIIYVDLSSYYIIVR HLA-DRB1*15:01
QKIRSMFIDDLLSIL HLA-DRB3*01:01
QKNLCFSLMDINPWL HLA-DRB3*01:01
QKVQMTYNWTQWLQT HLA-DPA1*01:03/DPB1*02:01
QLDKHQALRIFFLSI HLA-DPA1*01/DPB1*04:01
QLDKHQALRIFFLSI HLA-DPA1*01:03/DPB1*02:01
QLDPVVTDVVYHDHG HLA-DRB3*01:01
QLDPVVTDVVYHDHG HLA-DRB3*01:01
QLDPVVTDVVYHDHG HLA-DRB1*03:01
QLDPVVTDVVYHDHG HLA-DRB3*01:01
QLDPVVTDVVYHDHG HLA-DRB1*03:01
QMTYNWTQWLQTLYT HLA-DPA1*01:03/DPB1*02:01
QMTYNWTQWLQTLYT HLA-DPA1*01/DPB1*04:01
QMTYNWTQWLQTLYT HLA-DPA1*02:01/DPB1*01:01
QMTYNWTQWLQTLYT HLA-DPA1*03:01/DPB1*04:02
QPPYHWSIERSISPD HLA-DRB1*09:01
QPPYHWSIERSISPD HLA-DRB1*09:01
QQSLFSFDNVKNFRD HLA-DRB3*01:01
QRVNIDLDVLKAITP HLA-DRB3*01:01
QSLFSFDNVKNFRDG HLA-DRB3*01:01
QTELSLDLALSKYLS HLA-DRB1*03:01
QTLYTMIMEENVPDM HLA-DQA1*04:01/DQB1*04:02
QTSRNVNLDSIKLYT HLA-DRB1*03:01
QVAELAAAVQETSAG HLA-DQA1*04:01/DQB1*04:02
QVAELAAAVQETSAG HLA-DQA1*03:01/DQB1*03:02
QWLQTLYTMIMEENV HLA-DQA1*04:01/DQB1*04:02
RAVEIISDIGNYVEE HLA-DRB3*01:01
RAVEIISDIGNYVEE HLA-DRB1*13:02
RCYCNLLILILMISE HLA-DPA1*03:01/DPB1*04:02
RCYCNLLILILMISE HLA-DRB1*11:01
RDTIIIMLNEAMNYF HLA-DRB3*01:01
REFMIYDDVFIDNTG HLA-DRB3*01:01
REFMIYDDVFIDNTG HLA-DQA1*01:01/DQB1*05:01
RELVVSSHVPRFALS HLA-DRB1*03:01
RHHAGGIDQNMANRL HLA-DRB1*13:02

RHTDDQVFNNPASKI HLA-DRB1*13:02
RIFVPATNSPELRWE HLA-DRB1*04:05
RITDMSTLVSAVITI HLA-DQA1*04:01/DQB1*04:02
RKKIRTIAAYPLGVG HLA-DRB1*08:02
RKVLILDFRSKLMTK HLA-DRB1*03:01
RLAGVIMAGVAIGIA HLA-DQA1*05:01/DQB1*03:01
RLTNSLLNLRSRLAA HLA-DRB1*15:01
RMMEMLKEETWRIYP HLA-DRB3*01:01
RNVNLDSIKLYTSDD HLA-DRB1*03:01
RPGALIRSLLNDPDI HLA-DRB1*04:05
RSMFIDDLLSILNID HLA-DRB3*01:01
RTLGYATEDFDDLLE HLA-DQA1*05:01/DQB1*02:01
RTMLEFRRNNAIAFN HLA-DRB3*02:02
RTMLEFRRNNAIAFN HLA-DRB3*01:01
RVNIDLDVLKAITPV HLA-DRB3*01:01
RWELTLFALDVIRSP HLA-DQA1*01:01/DQB1*05:01
SAFNTVIALLGSIVI HLA-DRB1*07:01
SAVYNNEFYYVLCAV HLA-DPA1*01/DPB1*04:01
SDAKMLSYAPEIAVS HLA-DRB1*09:01
SDAKMLSYAPEIAVS HLA-DRB1*09:01
SDDFSNTFFPHDTDR HLA-DPA1*01/DPB1*04:01
SDDIQLDPVVTDVVY HLA-DRB1*03:01
SDDIQLDPVVTDVVY HLA-DRB1*03:01
SDDIQLDPVVTDVVY HLA-DRB1*03:01
SDLSMYMKDKALSPI HLA-DRB3*01:01
SDQVAELAAAVQETS HLA-DQA1*04:01/DQB1*04:02
SDQVAELAAAVQETS HLA-DQA1*03:01/DQB1*03:02
SEVIVGISPEDEEPS HLA-DQA1*04:01/DQB1*04:02
SEWISIVPNFILVRN HLA-DRB5*01:01
SFLMDRRVILPRAAH HLA-DRB3*01:01
SFSWDTMIKFGDVLT HLA-DRB3*01:01
SIKLINLDMRLNHIE HLA-DRB1*03:01
SIKLYTSDDEEADQL HLA-DRB3*01:01
SIKLYTSDDEEADQL HLA-DRB3*01:01
SIKLYTSDDEEADQL HLA-DRB3*01:01
SILLTLFRRTKKKYR HLA-DRB5*01:01
SILLTLFRRTKKKYR HLA-DRB1*11:01
SINETLTLDVTSPIS HLA-DRB3*01:01
SIPREFMIYDDVFID HLA-DRB3*01:01
SIPREFMIYDDVFID HLA-DQA1*05:01/DQB1*02:01
SIPREFMIYDDVFID HLA-DQA1*01:01/DQB1*05:01
SITGQIIYVDLSSYY HLA-DRB3*01:01
SIVIIVMNIMIIQNY HLA-DRB4*01:01
SIVIIVMNIMIIQNY HLA-DRB1*15:01
SIVPNFILVRNTLIS HLA-DRB1*07:01
SIVPNFILVRNTLIS HLA-DRB1*01:01
SIVPNFILVRNTLIS HLA-DRB1*04:05
SKGKTPFVDSRAYGL HLA-DRB1*07:01
SKILSAFNTVIALLG HLA-DRB1*07:01
SKILSAFNTVIALLG HLA-DPA1*03:01/DPB1*04:02
SKYLSDLLFVFGPNL HLA-DRB3*01:01
SLCIGLITFISFIIV HLA-DPA1*01:03/DPB1*02:01
SLCIGLITFISFIIV HLA-DRB1*15:01
SLFSFDNVKNFRDGS HLA-DRB3*01:01
SLHIKINGVISKRLF HLA-DRB1*07:01
SLISMLSMIILYVLS HLA-DRB4*01:01
SLTNEPYGAAVQLRE HLA-DQA1*04:01/DQB1*04:02
SMFIDDLLSILNIDN HLA-DRB3*01:01
SMIILYVLSIASLCI HLA-DRB1*11:01

SMIILYVLSIASLCI HLA-DRB1*15:01
SMIILYVLSIASLCI HLA-DRB1*04:01
SMLSMIILYVLSIAS HLA-DRB1*11:01
SMLSMIILYVLSIAS HLA-DRB1*15:01
SMMIMIPGKGKGERK HLA-DRB5*01:01
SMTIQAISQAFGGNY HLA-DRB1*08:02
SMYMKDKALSPIKDE HLA-DRB3*01:01
SNPLTKDIVIKMIPN HLA-DRB1*03:01
SQRVNIDLDVLKAIT HLA-DRB3*01:01
SRNVNLDSIKLYTSD HLA-DRB1*03:01
SSDQVAELAAAVQET HLA-DQA1*04:01/DQB1*04:02
SSDQVAELAAAVQET HLA-DQA1*03:01/DQB1*03:02
SSEVIVGISPEDEEP HLA-DQA1*04:01/DQB1*04:02
SSEVIVGISPEDEEP HLA-DQA1*04:01/DQB1*04:02
SSEVIVGISPEDEEP HLA-DQA1*03:01/DQB1*03:02
SSEVIVGISPEDEEP HLA-DQA1*04:01/DQB1*04:02
SSSEVIVGISPEDEE HLA-DQA1*04:01/DQB1*04:02
SSSEVIVGISPEDEE HLA-DQA1*04:01/DQB1*04:02
SSSEVIVGISPEDEE HLA-DQA1*03:01/DQB1*03:02
SSSEVIVGISPEDEE HLA-DQA1*04:01/DQB1*04:02
SSYYIIVRVYFPILT HLA-DPA1*01:03/DPB1*02:01
SSYYIIVRVYFPILT HLA-DRB3*01:01
SSYYIIVRVYFPILT HLA-DPA1*01/DPB1*04:01
SSYYIIVRVYFPILT HLA-DPA1*02:01/DPB1*01:01
STLVSAVITIEAQIW HLA-DQA1*03:01/DQB1*03:02
SVTFKREMSISSLAN HLA-DRB1*04:01
SYYIIVRVYFPILTE HLA-DPA1*01:03/DPB1*02:01
SYYIIVRVYFPILTE HLA-DPA1*01/DPB1*04:01
SYYIIVRVYFPILTE HLA-DRB3*01:01
SYYIIVRVYFPILTE HLA-DPA1*02:01/DPB1*01:01
SYYIIVRVYFPILTE HLA-DQA1*01:01/DQB1*05:01
TDDQVFNNPASKIKQ HLA-DRB1*13:02
TDMSTLVSAVITIEA HLA-DQA1*04:01/DQB1*04:02
TEMRNLLSQSLSVRK HLA-DRB4*01:01
TGQIIYVDLSSYYII HLA-DRB1*15:01
TGQIIYVDLSSYYII HLA-DRB3*01:01
THDLVGDVRLAGVIM HLA-DRB1*03:01
TIEGHLVSMMIMIPG HLA-DRB4*01:01
TIIIMLNEAMNYFDD HLA-DRB3*01:01
TITNCFLLKNKIWCI HLA-DRB1*11:01
TKDIVIKMIPNVSNM HLA-DRB1*08:02
TKIRIFVPATNSPEL HLA-DRB1*04:05
TKIRIFVPATNSPEL HLA-DRB1*08:02
TLFALDVIRSPSAAE HLA-DRB1*11:01
TLFRRTKKKYRRHTD HLA-DRB5*01:01
TLFRRTKKKYRRHTD HLA-DRB1*11:01
TLTTKIRIFVPATNS HLA-DRB1*08:02
TLVSAVITIEAQIWI HLA-DQA1*03:01/DQB1*03:02
TLYTMIMEENVPDMD HLA-DQA1*04:01/DQB1*04:02
TMLEFRRNNAIAFNL HLA-DRB3*02:02
TMLEFRRNNAIAFNL HLA-DRB3*01:01
TMLEFRRNNAIAFNL HLA-DRB1*13:02
TNEPYGAAVQLREDL HLA-DQA1*04:01/DQB1*04:02
TNSLLNLRSRLAAKA HLA-DRB1*15:01
TQWLQTLYTMIMEEN HLA-DQA1*04:01/DQB1*04:02
TRNIHLLGRKTCLGR HLA-DRB1*11:01
TSRNVNLDSIKLYTS HLA-DRB1*03:01
TTKIRIFVPATNSPE HLA-DRB1*08:02
TTKIRIFVPATNSPE HLA-DRB1*04:05

TVNPSLISMLSMIIL HLA-DRB4*01:01
TVNPSLISMLSMIIL HLA-DRB1*15:01
TWRIYPVLLPQMELL HLA-DRB1*08:02
TYNWTQWLQTLYTMI HLA-DPA1*01:03/DPB1*02:01
TYNWTQWLQTLYTMI HLA-DPA1*02:01/DPB1*01:01
VAELAAAVQETSAGR HLA-DQA1*04:01/DQB1*04:02
VCLVSDAKMLSYAPE HLA-DRB1*03:01
VCLVSDAKMLSYAPE HLA-DRB1*03:01
VCLVSDAKMLSYAPE HLA-DRB1*11:01
VCLVSDAKMLSYAPE HLA-DRB1*03:01
VEIISDIGNYVEETG HLA-DRB1*13:02
VFYQASFSWDTMIKF HLA-DRB3*01:01
VIIDVGSMVNGIPVM HLA-DRB1*03:01
VIIVMNIMIIQNYTR HLA-DRB4*01:01
VIIVMNIMIIQNYTR HLA-DRB5*01:01
VIIVMNIMIIQNYTR HLA-DRB1*15:01
VIKDALQGIQQQIKG HLA-DRB4*01:01
VIKMIPNVSNMSQCT HLA-DRB1*08:02
VMNIMIIQNYTRSTD HLA-DRB5*01:01
VNIDLDVLKAITPVS HLA-DRB3*01:01
VNLDSIKLYTSDDEE HLA-DRB1*03:01
VNPSLISMLSMIILY HLA-DRB4*01:01
VNPSLISMLSMIILY HLA-DRB1*15:01
VPNFILVRNTLISNI HLA-DRB1*01:01
VPNFILVRNTLISNI HLA-DRB1*08:02
VPNFILVRNTLISNI HLA-DRB1*04:05
VPNFILVRNTLISNI HLA-DRB1*07:01
VQMTYNWTQWLQTLY HLA-DPA1*01:03/DPB1*02:01
VQMTYNWTQWLQTLY HLA-DPA1*01/DPB1*04:01
VQMTYNWTQWLQTLY HLA-DPA1*02:01/DPB1*01:01
VRLAGVIMAGVAIGI HLA-DQA1*05:01/DQB1*03:01
VSDAKMLSYAPEIAV HLA-DRB1*09:01
VSDAKMLSYAPEIAV HLA-DRB1*09:01
VSMMIMIPGKGKGER HLA-DRB5*01:01
VSNSMTIQAISQAFG HLA-DQA1*01:02/DQB1*06:02
VVTDVVYHDHGGECT HLA-DRB3*01:01
VVTDVVYHDHGGECT HLA-DRB3*01:01
VVTDVVYHDHGGECT HLA-DRB3*01:01
VYNDAFLIDRINWIS HLA-DRB3*01:01
VYNDAFLIDRINWIS HLA-DRB1*03:01
WDGKRAWVEEWCNPA HLA-DQA1*01:01/DQB1*05:01
WEGVYNDAFLIDRIN HLA-DRB3*01:01
WEGVYNDAFLIDRIN HLA-DPA1*01:03/DPB1*02:01
WELTLFALDVIRSPS HLA-DQA1*01:01/DQB1*05:01
WLQTLYTMIMEENVP HLA-DQA1*04:01/DQB1*04:02
WRIYPVLLPQMELLE HLA-DRB1*08:02
WRYESMAIFAERLDE HLA-DRB3*01:01
WSFAMGVATTIDRSM HLA-DRB1*09:01
WTQWLQTLYTMIMEE HLA-DQA1*04:01/DQB1*04:02
YCINILKVIQQLLIS HLA-DRB4*01:01
YCNLLILILMISECS HLA-DPA1*03:01/DPB1*04:02
YCNLLILILMISECS HLA-DRB1*11:01
YCNLLILILMISECS HLA-DRB1*04:05
YDDVFIDNTGRILKG HLA-DRB3*01:01
YDDVFIDNTGRILKG HLA-DRB1*03:01
YFAYSHLERIGSCSR HLA-DPA1*03:01/DPB1*04:02
YFAYSHLERIGSCSR HLA-DRB1*11:01
YHCSAVYNNEFYYVL HLA-DPA1*01/DPB1*04:01
YIIVRVYFPILTEIQ HLA-DPA1*01:03/DPB1*02:01

YIIVRVYFPILTEIQ HLA-DPA1*01/DPB1*04:01
YIIVRVYFPILTEIQ HLA-DPA1*02:01/DPB1*01:01
YIIVRVYFPILTEIQ HLA-DQA1*01:01/DQB1*05:01
YLICYGFVEDVERTP HLA-DQA1*01:01/DQB1*05:01
YLSDLLFVFGPNLQD HLA-DRB3*01:01
YMYLICYGFVEDVER HLA-DQA1*01:01/DQB1*05:01
YNDAFLIDRINWISA HLA-DRB3*01:01
YNDAFLIDRINWISA HLA-DRB1*03:01
YNQFLILNKLLSNRR HLA-DRB1*11:01
YPLLWSFAMGVATTI HLA-DRB1*09:01
YQASFSWDTMIKFGD HLA-DRB3*01:01
YQIDQPFFVPTKITS HLA-DRB3*01:01
YSHLERIGSCSRGVS HLA-DRB1*11:01
YTSDDEEADQLEFED HLA-DQA1*03:01/DQB1*03:02
YYIIVRVYFPILTEI HLA-DPA1*01:03/DPB1*02:01
YYIIVRVYFPILTEI HLA-DPA1*01/DPB1*04:01
YYIIVRVYFPILTEI HLA-DPA1*02:01/DPB1*01:01
YYIIVRVYFPILTEI HLA-DQA1*01:01/DQB1*05:01
YYIIVRVYFPILTEI HLA-DRB3*01:01
YYQIDQPFFVPTKIT HLA-DRB3*01:01
